# Message in a Bottle—Metabarcoding enables biodiversity comparisons across ecoregions

**DOI:** 10.1093/gigascience/giac040

**Published:** 2022-04-28

**Authors:** D Steinke, S L deWaard, J E Sones, N V Ivanova, S W J Prosser, K Perez, T W A Braukmann, M Milton, E V Zakharov, J R deWaard, S Ratnasingham, P D N Hebert

**Affiliations:** Centre for Biodiversity Genomics, University of Guelph, 50 Stone Road East, Guelph, ONT N1G 2W1, Canada; Department of Integrative Biology, University of Guelph, 50 Stone Road East, Guelph, ONT N1G 2W1, Canada; Centre for Biodiversity Genomics, University of Guelph, 50 Stone Road East, Guelph, ONT N1G 2W1, Canada; Centre for Biodiversity Genomics, University of Guelph, 50 Stone Road East, Guelph, ONT N1G 2W1, Canada; Centre for Biodiversity Genomics, University of Guelph, 50 Stone Road East, Guelph, ONT N1G 2W1, Canada; Department of Integrative Biology, University of Guelph, 50 Stone Road East, Guelph, ONT N1G 2W1, Canada; Centre for Biodiversity Genomics, University of Guelph, 50 Stone Road East, Guelph, ONT N1G 2W1, Canada; Centre for Biodiversity Genomics, University of Guelph, 50 Stone Road East, Guelph, ONT N1G 2W1, Canada; Centre for Biodiversity Genomics, University of Guelph, 50 Stone Road East, Guelph, ONT N1G 2W1, Canada; Centre for Biodiversity Genomics, University of Guelph, 50 Stone Road East, Guelph, ONT N1G 2W1, Canada; Centre for Biodiversity Genomics, University of Guelph, 50 Stone Road East, Guelph, ONT N1G 2W1, Canada; Department of Integrative Biology, University of Guelph, 50 Stone Road East, Guelph, ONT N1G 2W1, Canada; Centre for Biodiversity Genomics, University of Guelph, 50 Stone Road East, Guelph, ONT N1G 2W1, Canada; School of Environmental Sciences, University of Guelph, 50 Stone Road East, Guelph, ONT N1G 2W1, Canada; Centre for Biodiversity Genomics, University of Guelph, 50 Stone Road East, Guelph, ONT N1G 2W1, Canada; Department of Integrative Biology, University of Guelph, 50 Stone Road East, Guelph, ONT N1G 2W1, Canada; Centre for Biodiversity Genomics, University of Guelph, 50 Stone Road East, Guelph, ONT N1G 2W1, Canada; Department of Integrative Biology, University of Guelph, 50 Stone Road East, Guelph, ONT N1G 2W1, Canada

## Abstract

**Background:**

Traditional biomonitoring approaches have delivered a basic understanding of biodiversity, but they cannot support the large-scale assessments required to manage and protect entire ecosystems. This study used DNA metabarcoding to assess spatial and temporal variation in species richness and diversity in arthropod communities from 52 protected areas spanning 3 Canadian ecoregions.

**Results:**

This study revealed the presence of 26,263 arthropod species in the 3 ecoregions and indicated that at least another 3,000–5,000 await detection. Results further demonstrate that communities are more similar within than between ecoregions, even after controlling for geographical distance. Overall α-diversity declined from east to west, reflecting a gradient in habitat disturbance. Shifts in species composition were high at every site, with turnover greater than nestedness, suggesting the presence of many transient species.

**Conclusions:**

Differences in species composition among their arthropod communities confirm that ecoregions are a useful synoptic for biogeographic patterns and for structuring conservation efforts. The present results also demonstrate that metabarcoding enables large-scale monitoring of shifts in species composition, making it possible to move beyond the biomass measurements that have been the key metric used in prior efforts to track change in arthropod communities.

## Background

Terrestrial organisms are exposed to diverse anthropogenic stressors, including climate change, resource extraction, and agriculture. Habitat degradation, pesticide use, invasive species, and associated shifts in food webs have provoked major reductions in the diversity and abundance of terrestrial arthropods [1–4]. These declines have led to calls for more comprehensive biosurveillance to inform environmental management and conservation. Long-term monitoring of species composition is essential to quantify biological change, but efforts using morphological diagnostics have targeted a small set of indicator species [5] because of the need for taxonomic experts for each group. As a consequence, they cannot support the broad assessments needed to manage and protect ecosystems, let alone forecast human impacts on them by integrating statistical modelling. The latter methods demand comprehensive data on species distributions and abundance [6], information that is currently unavailable because of the prior focus on selected biotic compartments at limited geographic scale.

Two methodological advances promise to meet the need for comprehensive biodiversity data. First, identification systems based on the analysis of sequence variation in short, standardized gene regions (i.e., DNA barcodes) enable species discrimination [7]. Second, high-throughput sequencers (HTS) permit the inexpensive acquisition of millions of DNA barcode records [8]. These advances now enable biodiversity surveys at speeds and scales that were previously inconceivable. In particular, the coupling of HTS with DNA barcoding, known as metabarcoding [9], has a compelling advantage over traditional approaches for tracking shifts in species presence. It can generate georeferenced occurrence data from bulk samples at low cost, and a single instrument can process hundreds of bulk samples each week. Because the sequencing output of HTS is doubling every 9 months [10,11], analytical costs are certain to sharply decline, allowing production to soar. This augmented capacity for data generation has already enabled large-scale biotic surveys of aquatic and terrestrial arthropods [12–15], vertebrates [16], pollen [17], diatoms [18], and fungi [19–21].

Access to large collections of specimens is essential to capitalize on the analytical capacity provided by DNA metabarcoding. Among the many approaches used to sample terrestrial arthropods, Malaise traps [22] have gained wide adoption because they collect large, diverse samples with little effort [23]. Although most effective for sampling flying insects, they also collect ground-active arthropods. By coupling DNA barcoding with Malaise trapping [24,25], high-resolution monitoring networks for arthropods are within reach, but there are challenges. Data interpretation requires a well-parameterized DNA barcode reference library for the region under investigation, creating the need for a system to aid site selection. Ecoregions are designed to serve as spatial framework for the research, assessment, and monitoring of ecosystems and therefore represent a good candidate [26–29], although their boundaries are rarely sharply defined and they are based on distributional data for a narrow range of taxa. Despite these limitations, ecoregions have been widely and successfully used to guide management decisions and to explore species and community diversity patterns [30,31]. As a result, they are a good candidate to serve as the backbone for a large-scale monitoring network. The most widely adopted schema partitions the world's 14 terrestrial biomes into 846 ecoregions [31].

**Figure 1:**
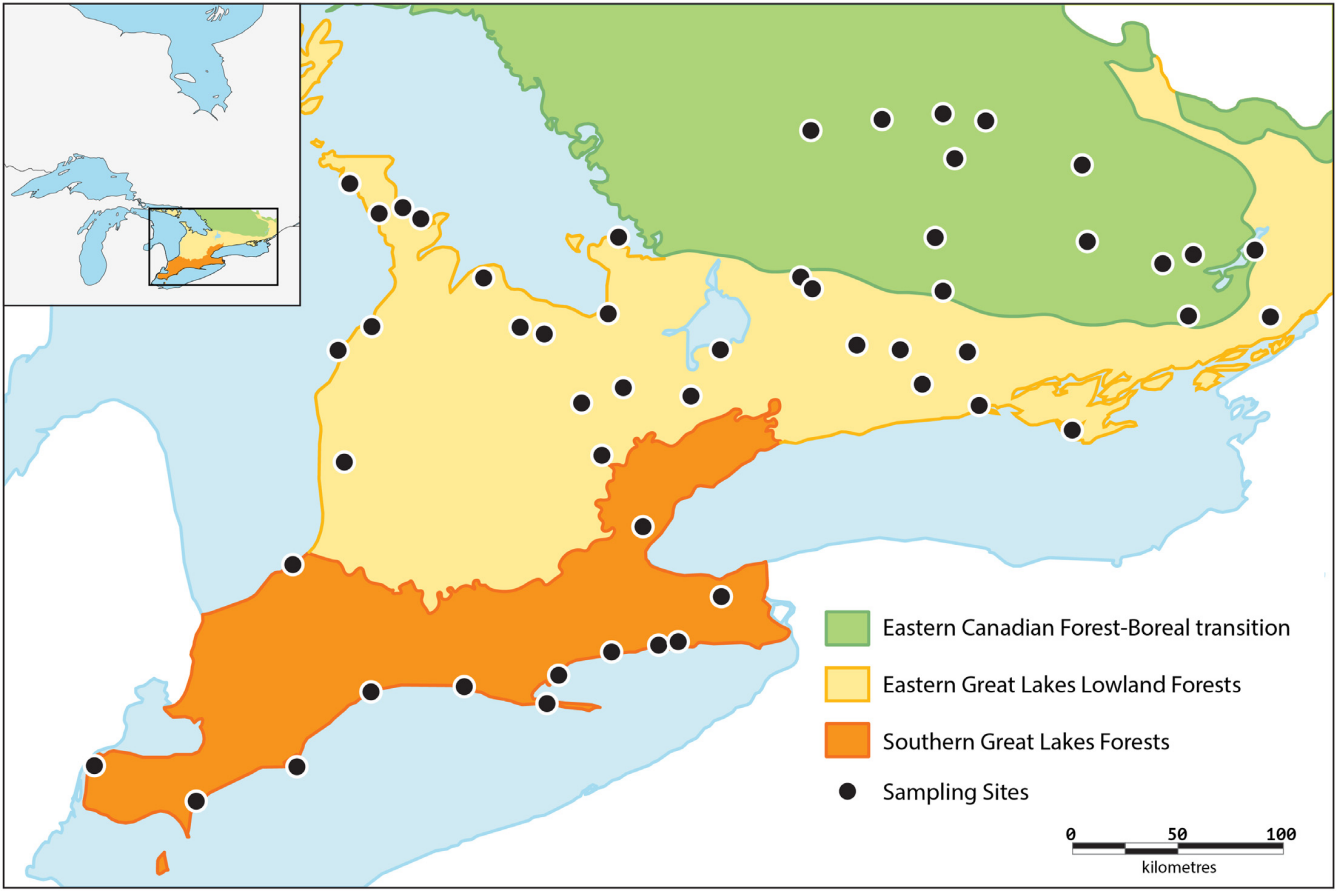
Map of sampling locations and ecoregion boundaries in Southern Ontario, Canada.

This study demonstrates the feasibility of using metabarcoding for the comparison of temporal and spatial patterns of arthropod communities in 3 of Canada's 47 terrestrial ecoregions: the Eastern Canadian Forest–Boreal Transition (ECF; 75,000 km^2^), the Eastern Great Lakes Lowland Forests (EGL; 63,000 km^2^), and the Southern Great Lakes Forests (SGL; 22,000 km^2^) (Fig. 1). Forest cover declines from 77.7% in the ECF to 30.1% in the EGL and just 12.1% in the SGL, while cropland/pastures cover 78% of the SGL, 57% of the EGL, and 3% of the ECF [31]. The EGL and SGL are the most populated ecoregions in Ontario, with developed land (e.g., urban, road networks) encompassing >7% of the SGL [32]. As such, these ecoregions provide a good basis for assessing the impacts of varied disturbance regimes on biodiversity.

### Data description

Collections were made by deploying a Malaise trap at 52 sites in these 3 ecoregions, and samples were metabarcoded to examine variation in their species richness (α-diversity), community composition (β-diversity), and phylogenetic diversity. Malaise traps were deployed for 20 weeks at 15 sites in the ECF, 24 sites in the EGL, and 13 sites in the SGL. Catches were harvested at 2-week intervals, and 410 of the resultant 520 samples were designated for metabarcoding (the others were reserved for single-specimen barcoding). Analysis began with non-destructive lysis of the specimens in each bi-weekly sample, followed by DNA extraction using a membrane-based protocol [33]. A 463-bp amplicon of cytochrome *c* oxidase I (COI) was then PCR amplified, and the amplicon pools from each set of 10 samples were sequenced on an Ion Torrent S5 using a 530 chip with a maximum read length output of 600 bp. This chipset usually produces 9–12 million reads of varying length at a 1–2% error rate. The sequences were subsequently analysed using mBRAVE [34]. All raw HTS datasets were deposited in the SRA [35] under the BioProject accession No. PRJNA629553.

## Results

**Figure 2:**
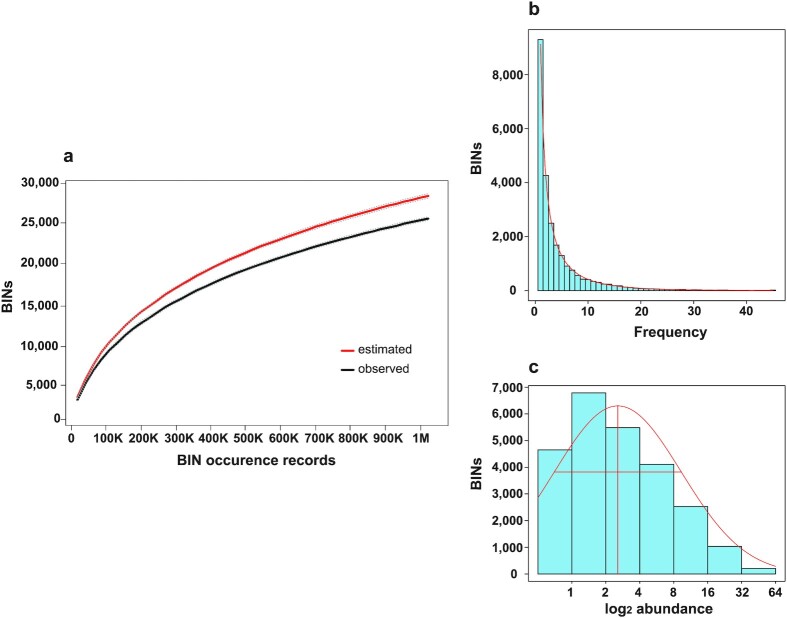
(a) BIN accumulation curve for the 410 Malaise trap samples collected in 51 Ontario provincial parks. (b) Fisher log series fit to the number of sites where each BIN was observed. (c) Preston lognormal species abundance curve showing the total BINs within each log_2_ abundance interval.

Sequence analysis of the 410 samples produced 367,823,207 reads across 41 S5 runs (mean reads per run = 8.97 million, see Supplementary Table S1). Two-thirds were filtered, leaving 126,253,260 reads that could be assigned to a BIN (Barcode Index Number [36]) on BOLD [37] (Supplementary Fig. S1). Nearly all reads (99.3%) found a BIN match on BOLD, but those that failed were *de novo* clustered using mBRAVE with a 99% similarity threshold. The latter analysis recognized an average of 28 additional operational taxonomic units (OTUs) per sample, but >96% of them reflected sequencing/PCR errors (e.g., chimeras, sequences with multiple indels) or NUMTs so they were excluded from further analysis. Consideration of the assigned reads revealed 26,263 BINs among the 52 sites, with more than one-third (9,301) found at only 1 site, respectively (Fig. 2b).

The Chao 1 [38] estimate for the total number of BINs present at the 52 sites was 29,640 (Fig. 2a), while species richness extrapolation based on the lognormal distribution (Fig. 2c [39]) suggested the presence of 31,516 BINs. On average, 0.3 million sequences were recovered per sample, and they revealed the presence of a mean of 2,352 BINs per site (range 996–4,581 BINs, Supplementary Table S2), with bi-weekly samples containing a mean of 619 (SE 14.3) BINs (range 60–1,666, Supplementary Table S3). Most low BIN counts occurred in spring (May) or fall (September), with diversity peaking in mid-summer (June/July) (Supplementary Fig. S2). Taxonomic composition at an ordinal level was similar among samples, with more than one-half of the BINs being flies (Diptera), followed by Hymenoptera, Lepidoptera, Hemiptera, and Coleoptera (Supplementary Fig. S3).

**Figure 3:**
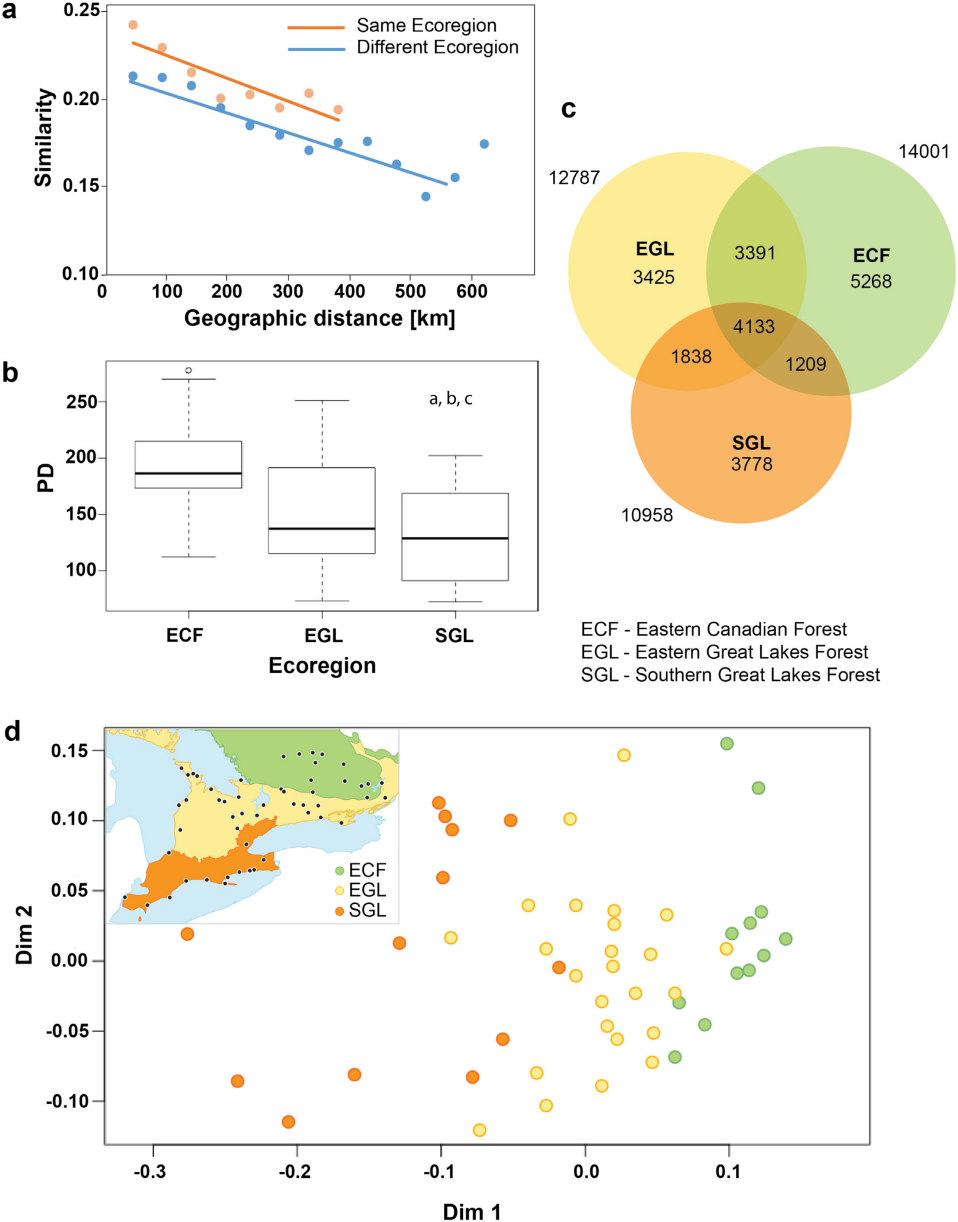
BIN compositional differences among 3 Ontario ecoregions: (a) Relationship between geographical distance and mean community similarity (Sørensen similarity coefficient) within and between ecoregions. (b) Box plots comparing Faith Phylogenetic Diversity for the 3 ecoregions. Significant differences between pairs are indicated with different lowercase letters (a: ECF/EGL; b: ECF/SGL; c: EGL/SGL). (c) Venn diagram depicting BIN overlap among ecoregions. (d) Non-metric multidimensional scaling (NMDS) plot using Bray-Curtis index coefficient. Colour coding is based on ecoregion.

Overlap in BIN composition was higher among parks in an ecoregion than among those in different ecoregions, even after geographical distance was considered (Fig. 3a). Sites in the ECF had the highest mean phylogenetic diversity followed by EGL and finally SGL (Fig. 3b), differences that were significant for all pairwise comparisons (Kruskal-Wallis and Dunn post hoc *P* < 0.005 for ECF/EGL, *P* < 0.003 for ECF/SGL, *P* < 0.05 for EGL/SGL). More BINs were collected in the ECF (14,001) than in the EGL (12,787) or SGL (10,958) (Fig. 3c). The Chao 1 estimates for the number of BINs present in each ecoregion were 15,401 for ECF, 14,577 for EGL, and 12,602 for SGL. The 3 ecoregions shared 4,133 BINs, while roughly one-third of those in each region were not collected elsewhere. A 2D non-metric multidimensional scaling (NMDS) ordination plot revealed that BIN assemblages for sites in each ecoregion formed cohesive groupings (Fig. 3d). Permutational multivariate analysis of variance (PERMANOVA) analysis also suggested that community structure varied between ecoregions (*R*^2^  =  0.141, *P*  <  0.001) and minimally with decreasing site elevation (*R*^2^  =  0.035, *P*  =  0.03) (Supplementary Table S4).

**Figure 4:**
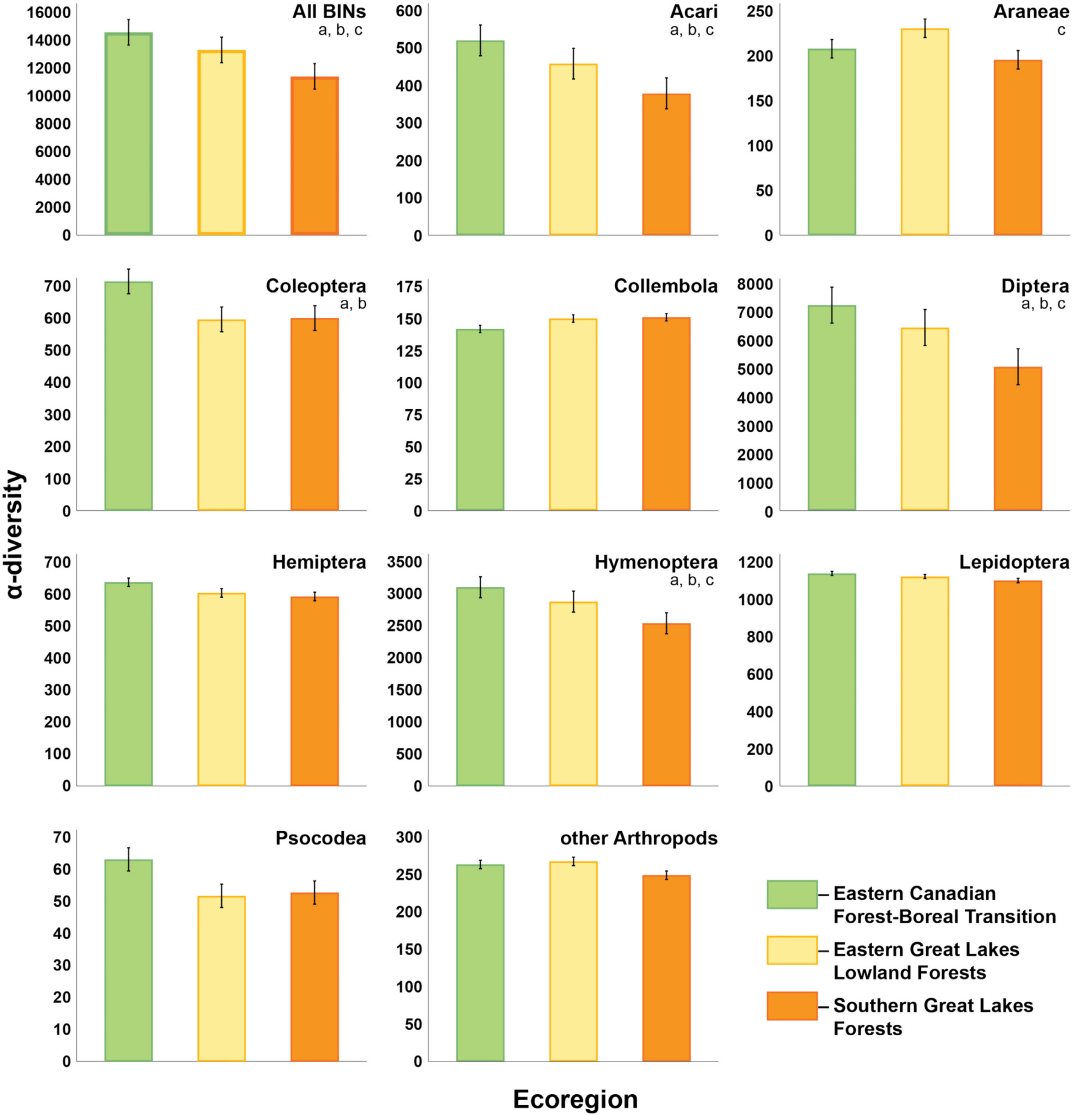
Comparison of α-diversity (± SE) in 3 Ontario ecoregions for all BINs and for 10 arthropod taxa using 12 random sites from the total sites for each ecoregion. Statistical tests are based on Kruskal–Wallis followed by Mann–Whitney post hoc comparisons with Bonferroni correction. Significant differences between pairs are indicated with different lowercase letters (a: ECF/EGL, b: ECF/SGL, c: EGL/SGL).

Overall, α-diversity was highest in the ECF, intermediate in the EGL, and lowest in SGL (Fig. 4). The α-diversity patterns for the varied insect orders followed the overall trend, but BIN richness for Collembola showed the opposite trend as it peaked in the SGL, while spider α-diversity was highest in the EGL.

**Figure 5:**
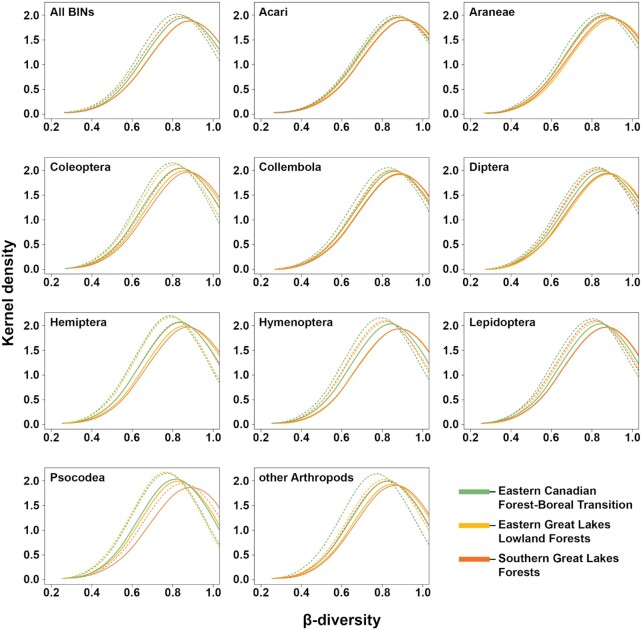
Total β-diversity (solid lines) and turnover (dotted lines) for 3 Ontario ecoregions. Values were computed using 1,000 bootstrap samples of 12 random sites from each ecoregion. Significant differences between ecoregions are detected when the peaks of the density plots do not overlap.

Levels of turnover (Fig. 5) were generally high among sites (species replacement by new species not found elsewhere) as well as high nestedness levels (gain and loss of species also found elsewhere). Lower levels of both turnover and nestedness were observed for most taxa at sites in the ECF, while the highest values were found in the SGL.

## Discussion

This study used metabarcoding to examine the species represented in 410 Malaise trap samples derived from 52 protected sites in 3 juxtaposed Canadian ecoregions. Metabarcoding revealed 26,263 species of arthropods, while Chao 1 and Preston lognormal extrapolations indicated that another 3,000–5,000 species await detection. Because just 52 sites were surveyed, a more comprehensive sampling program in these ecoregions might reveal as many as 50,000 species of arthropods. Nearly 5-fold variation (996–4,581) in BIN counts was detected among sites; counts showed a similar range for the 30 sites where all samples were analysed (996–4,508) and the 22 where just half were metabarcoded (1,312–4,581). On average, 619 BINs were recovered from each metabarcoded sample, a count that was 52.5% higher than the mean BIN count (406) for samples that were barcoded using the Pacific Biosciences Sequel platform (D. Steinke et al., in preparation). This difference suggests that more than half the BINs recovered from metabarcoded samples derive from environmental DNA attached to specimens in the sample, from their gut contents, or from sequence errors that escaped the stringent filtering conditions.

The 3 ecoregions examined in this study collectively span 160,000 km^2^, just 1.6% of Canada's land surface, but 2 (SGL, EGL) are among the most heavily populated areas in the country [32]. The ecoregions showed considerable overlap in species composition; 33.1% of the BINs recorded from ≥3 sites were shared by the 3 ecoregions. BIN richness was lowest in the southernmost ecoregion (SGL) and highest in the most northerly (ECF). This difference coincided with a disturbance gradient—from forested regions with low human density in the ECF (78% forest cover) to disturbed landscapes dominated by farmland/cities in the SGL (12% forest cover). The decline in species richness in response to disturbance is consistent with earlier studies [40–42], even though our collections all derived from protected areas. Gray et al. [43] reported that protected sites contain significantly higher species counts than adjacent disturbed areas, perhaps because communities in protected areas include representatives of original habitats and generalists from adjacent disturbed landscapes [44]. However, protected areas in the SGL were small islands of remnant forest in a landscape dominated by agricultural activity, so they were undoubtedly heavily exposed to pesticides, with agricultural fields creating dispersal barriers that further reduced diversity.

Our results indicate that α-diversity for major insect orders of flying insects (Diptera, Hymenoptera, Hemiptera, Lepidoptera) peaked in the least disturbed ecoregion (ECF). By contrast, 2 groups of arthropods (Araneae, Collembola) lacking flight showed a different trend, with their diversity peaking in other ecoregions. Aside from potential random sampling effects this difference might also reflect the fact that Malaise traps only sample flightless taxa with resident populations near the trap but capture flying insects from distant habitats. As such, biodiversity patterns for flying insects provide a regional perspective while those for taxa without flight provide a local perspective [25,45]. If so, the reduction in diversity of Collembola from the most southerly (SGL) to northerly (ECF) ecoregion might reflect the expected latitudinal gradient in biodiversity, undisrupted by disturbance because of the local source of specimens in each sample.

The present study establishes the feasibility of monitoring changes in species composition of arthropod communities [46, 47]. For all 3 ecoregions, temporal turnover was high, reflecting the seasonal succession of species. Species richness was lower at the beginning and end of the season and peaked in the summer months (Supplementary Fig. S2). β-diversity was lowest for most taxonomic groups at sites in the ECF and highest in the SGL. Species turnover was generally higher than nestedness, suggesting the presence of many transient species [48]. As many species were only collected at 1 or 2 sites (Fig. 2b), many samples likely included transients passively transported by the wind [49]. Wingless and small insects generally depend on air currents to carry them to new sites, and the Malaise trap can function as a windbreak.

Metabarcoding can already provide cost-effective biosurveillance as the present study analysed ∼856,000 specimens and generated 223,860 species occurrence records for ${\$}$82,000, an analytical cost of <$0.50 per record. By adopting simpler analytical protocols (e.g., destructive processing of samples) with ongoing reductions in sequencing costs [11], costs can be reduced by an order of magnitude, delivering species occurrence records for $0.04 apiece in the ecoregions targeted in this study. In settings with higher α-diversity, the cost could be halved. Aside from its cost-effectiveness for data acquisition, the digital format of metabarcoding results aids their curation, validation, and preservation. Current metabarcoding protocols cannot estimate the total abundance of each species in a sample. However, they have been used to provide relative abundance [50,51] or relative biomass [52,53]. This situation shifts when multiple samples are analysed because the abundance of a species can then be estimated from its frequency of occurrence in these samples (rare species will be recovered less frequently than abundant taxa).

Because the 846 currently recognized ecoregions [31] were largely delineated on the basis of distributional data for vascular plants and vertebrates, there remains a need to ascertain how well they represent diversity patterns in other taxa. Smith et al. [54] found that arthropods showed weak adherence to ecoregion boundaries and proposed this might reflect dispersal limitations linked to their small body size or to the biased assemblage of arthropod species with data. Our much larger dataset shows evidence of structuring by ecoregion as both phylogenetic diversity and BIN composition were significantly different among ecoregions, even when comparisons extended to widely separated sites. This result suggests that ecoregions do provide a useful structural framework, reinforcing results from earlier studies [55,56]. However, a third of species in this study crossed ecoregion boundaries and more extensive sampling would raise the incidence of shared species. The latter results make it clear that high sampling effort is required to better understand species distributions. In looking to the future, it is apparent that there is an immediate need for a more detailed understanding of the levels of species overlap between adjacent ecoregions. Is, for example, the pattern of high overlap in species composition among neighbouring ecoregions detected in this study a general pattern or are some ecoregion boundaries sharply delineated? Such information is critical in designing an effective global biomonitoring network to inform conservation efforts [57,58].

### Potential implications

Past monitoring programs have provided limited insights into the shifting distributions and abundances of arthropod species [59]. By coupling the use of an efficient collection method with the capacity of DNA metabarcoding to determine the species composition of bulk samples, this study confirms that compositional shifts in arthropod communities can be tracked using DNA metabarcoding [60]. The present results also indicate that the ecoregion concept not only furthers understanding of foundational biogeographic principles and improves their potential application to conservation efforts, but also provides a logical scaffold for large-scale monitoring networks.

## Methods

### Sample collection

An ez-Malaise trap (BioQuip Products, Compton, California, USA) was deployed to collect arthropods at 1 site in each of 50 provincial parks, while 2 sites were sampled in the final park (Algonquin) because of its large size. Trap catches were harvested every second week from early May through September, producing 10 samples per site, for a total of 520 samples. These samples were preserved in 95% ethanol and held at −20°C until DNA extraction. Five samples (weeks 1+2, 5+6, 9+10, 13+14, 17+18) from each of 22 sites were used for single-specimen barcoding (D. Steinke et al., in preparation), while the other 410 samples were analysed in this study. A direct count indicated that 230,000 specimens were present in the 21.2% of the samples that were barcoded. On this basis, the remaining samples (78.8%), those examined in this study, included ∼856,000 specimens.

### DNA extraction and PCR

DNA extraction used a membrane-based protocol [33] modified for bulk samples. Specimens were removed from ethanol by filtration through a sterile Microfunnel 0.45 µM Supor Membrane Filter (Pall Laboratory, Port Washington, New York, USA) using a 6-Funnel Manifold (Pall Laboratory, Port Washington, New York, USA). The wet weight of each sample was then ascertained to allow volume adjustment (Supplementary Table S5) of the lysis buffer [33]. Each sample was then incubated overnight at 56°C while gently mixed on a shaker. Eight 50-μL aliquots (technical replicates) from each of the 410 lysates were then transferred into 3,280 separate wells in 96-well microplates and DNA extracts were generated using Acroprep 3.0-µm glass fiber/0.2-µm Bio-Inert membrane plates (Pall Laboratory, Port Washington, New York, USA). Each plate contained 80 lysate samples, 8 technical replicates of a positive control (lysate from a bulk sample whose component specimens were individually Sanger sequenced; public BOLD dataset [77]) and 8 negative controls. Each lysate was mixed with 100 μL of binding mix, transferred to a column plate, and centrifuged at 5,000*g* for 5 min. DNA was then purified with 3 washes; the first used 180 μL of protein wash buffer centrifuged at 5,000*g* for 5 min. Each column was then washed twice with 600 μL of wash buffer centrifuged at 5,000*g* for 5 min. Columns were transferred to clean tubes and spun dry at 5,000*g* for 5 min to remove residual buffer before their transfer to clean collection tubes followed by incubation for 30 min at 56°C to dry the membrane. DNA was subsequently eluted by adding 60 μL of 10 mM Tris-HCl pH 8.0 followed by centrifugation at 5,000*g* for 5 min.

PCR reactions used a standard protocol [62]. Briefly, each reaction included 5% trehalose (Honeywell, Charlotte, North Carolina, USA), 1× Platinum Taq reaction buffer (Invitrogen), 2.5 mM magnesium chloride (Invitrogen, Waltham, Massachusetts, USA), 0.1 μM of each primer (Integrated DNA Technologies, Coralville, Iowa, USA), 50 μM of each dNTP (KAPA Biosystems), 0.3 units of Platinum Taq (Invitrogen, Waltham, Massachusetts, USA), 2 μL of DNA extract, and Hyclone ultra-pure water (Thermo Fisher Scientific, Waltham, Massachusetts, USA) for a final volume of 12.5 μL. Two-stage PCR was used to generate amplicon libraries for sequencing on an Ion Torrent S5 platform. The first round of PCR used the primer combination AncientLepF3 [63] and LepR1 [64] to amplify a 463-bp fragment of COI. Prior to the second PCR, first-round products were diluted 2× with ddH_2_O. Fusion primers were then used to attach platform-specific unique molecular identifiers (UMIs) along with the sequencing adaptors required for Ion Torrent S5 libraries. Both rounds of PCR used the same thermocycling conditions: initial denaturation at 94°C for 2 min, followed by 20 cycles of denaturation at 94°C for 40 sec, annealing at 51°C for 1 min, and extension at 72°C for 1 min, with a final extension at 72°C of 5 min.

### HTS library construction

For each plate, labelled products were pooled prior to sequencing. In total, 41 libraries were assembled. Each included 8 technical replicates of 10 samples plus 8 technical replicates of an extraction negative and a positive control, respectively (i.e., 96 samples). The 10 samples from each of the 30 sites that were only metabarcoded, together with positive and negative controls, were pooled after UMI tagging to create a library that was analysed on a 530 chip (30 chips in total). Five samples were available from each of the other 22 sites (where half the samples were retained for barcoding). The UMI-tagged amplicons from 5 samples from each of 2 sites were pooled with positive and negative controls to produce a single library. Amplicon libraries were prepared on an Ion Chef (Thermo Fisher Scientific, Waltham, Massachusetts, USA) and sequenced on an Ion Torrent S5 platform at the Centre for Biodiversity Genomics following the manufacturer's instructions (Thermo Fisher Scientific, Waltham, Massachusetts, USA).

### Sequence analysis

Reads from the 8 replicates for each sample were concatenated using a bash script and uploaded to mBRAVE [34] for quality filtering and subsequent queries using several reference libraries in an open reference approach. All reads were queried against 5 system libraries on mBRAVE: bacteria (SYS-CRLBACTERIA) to screen for potential contamination, e.g., by endosymbionts such as *Wolbachia*, chordates (SYS-CRLCHORDATA), insects (SYS-CRLINSECTA), non-insect arthropods (SYS-CRLNONINSECTARTH), and non-arthropod invertebrates (SYS-CRLNONARTHINVERT). All non-arthropod reads were discarded from further analysis. Sequences were only included in this analysis if they possessed a minimum length >350 bp and met the following 3 quality criteria (mean QV > 20; <25% positions with QV < 20; <5% positions with QV < 10). Reads were trimmed 30 bp from their 5′ terminus with a set trim length filter of 450 bp. Reads were matched to the sequences in each reference library with an ID distance threshold of 3% but were only retained for further analysis when ≥5 reads matched an OTU in the reference database. This number is based on earlier benchmarking of the assignment algorithm on mBRAVE, and IonTorrent-generated sequences provided the best compromise between removing error and retaining real matches. All reads failing to match any sequence in the 5 reference libraries were clustered at an OTU threshold of 1% with a minimum of 5 reads per cluster, again a value based on initial benchmarking. All raw data are available in the NCBI SRA (BioProject accession No. PRJNA629553).

Using mBRAVE, we generated BIN (and OTU) tables including all library queries for each individual plate/run (10 samples, plus a negative and positive control [61] for each run). Read counts for any BINs recovered from the negative control on a plate were subtracted from the counts for the same BIN in the 80 non-control wells in the run. When this subtraction reduced the read count for a BIN to zero, its occurrence was removed. This step reduced the effects of rare tag switching on data integrity [65] and reduced background contamination.

### Ecoregion analysis

OTU tables were converted to presence/absence matrices. To determine the completeness of sampling, we calculated accumulation curves and the Chao 1 estimator for total diversity [38] using the vegan package [66]. For further extrapolation of species richness, we used the lognormal species abundance distribution [39]. The fit of the Fisher Logseries [67] was used to determine relative BIN abundance. Both methods are implemented in vegan (fisherfit, prestonfit) [66]. We calculated the Sørensen similarity coefficient to ascertain whether differences in species assemblages were greater between or across ecoregion borders. Differences in BIN composition among the 3 ecoregions were examined using NMDS with the Bray-Curtis index coefficient as implemented in vegan [66]. The adonis function of the vegan package was used to conduct a PERMANOVA to partition distance matrices among sources of variation (factors such as elevation and ecoregion).

A maximum likelihood phylogeny was inferred for a BIN sequence alignment using RAxML Black box (RAxML, RRID:SCR_006086) [68] on XCEDE via the CIPRES portal (CIPRES Science Gateway, RRID:SCR_008439) [69]. This system uses a GTRCAT model, which is recommended for larger datasets. The resulting phylogeny comprising 26,263 BIN sequences was used to calculate the Faith phylogenetic distance (PD) [70] using the picante package [71]. Because this measure is influenced by polytomies in a phylogeny [72], only 1 representative was included per BIN to avoid bias introduced by variation in the number of records for each BIN. A Kruskal–Wallis test followed by a Dunn post hoc analysis was used to determine whether significant PD differences existed between ecoregions.

The α-diversity was quantified as the number of BINs observed at a site. It was calculated using 12 random sites from the total sites for each ecoregion. Pairwise BIN diversity among ecoregions was evaluated using the nonparametric multiple comparison function implemented in the R package dunn.test 1.2.4 [73]. dunn.test is equivalent to the Kruskall–Wallis and pairwise Mann–Whitney post hoc tests with Bonferroni correction. The β-diversity was computed as multi-site Sorensen and Simpson indices using the betapart 1.3. package [74]. β-diversity calculations between pairs of ecoregions were computed using 12 random sites from the total pool of sites for each ecoregion, and resampled 1,000 times. We then split among-site β-diversity into turnover and nestedness.

All analyses were performed in R v.3.4.4 [75].

## Data Availability

All raw HTS datasets underlying this article are available in the SRA [76] and can be accessed with BioProject accession No. PRJNA629553. Additional supporting data and materials are available on the *GigaScience* database [77].

## Additional Files

**Supplementary Figure S1**: Relationship between filtered read count and number of BINs for 410 metabarcoded samples from 3 ecoregions

**Supplementary Figure S2**: Bar plot showing α-diversity per month for all 52 sites

**Supplementary Figure S3**: Patterns of α-diversity and read abundance per major arthropod group and site

**Supplementary Table S1:** mBRAVE project codes as well as samples analyzed and read coverage for each 530 chip analyzed on the Ion Torrent S5

**Supplementary Table S2:** GPS coordinates, elevation (m), and ecoregion assignment for the 52 sampling sites and the number of BINs recovered from each site

**Supplementary Table S3:** Sampling dates, pre- and post-filtering read counts, BIN and OTU counts for the 410 samplesResults of PERMANOVA to partition distance matrices among sources of variation

**Supplementary Table S4:** Results of PERMANOVA to partition distance matrices among sources of variation

**Supplementary Table S5:** Wet weight (g) to insect lysis buffer volume (mL) ratios for Malaise trap bulk samples.

giac040_GIGA-D-21-00198_Original_Submission

giac040_GIGA-D-21-00198_Revision_1

giac040_GIGA-D-21-00198_Revision_2

giac040_Response_to_Reviewer_Comments_Original_Submission

giac040_Response_to_Reviewer_Comments_Revision_1

giac040_Reviewer_1_Report_Original_Submission Camila Duarte Ritter -- 9/13/2021 Reviewed

giac040_Reviewer_1_Report_Revision_1 Camila Duarte Ritter -- 3/1/2022 Reviewed

giac040_Reviewer_2_Report_Original_Submission Shanlin Liu -- 9/15/2021 Reviewed

giac040_Reviewer_3_Report_Original_Submission Kingsly Beng -- 9/21/2021 Reviewed

giac040_Reviewer_4_Report_Original_Submission Christina Lynggaard -- 9/24/2021 Reviewed

giac040_Supplemental_Figures_and_Tables

## Abbreviations

BIN: Barcode Index Number; bp: base pairs; COI: cytochrome *c* oxidase I; ECF: Eastern Canadian Forest–Boreal Transition; EGL: Eastern Great Lakes Lowland Forests; HTS: high-throughput sequencers; mBRAVE: Multiplex Barcode Research And Visualization Environment; NCBI: National Center for Biotechnology Information; SGL: Southern Great Lakes Forests; NMDS: non-metric multidimensional scaling; NUMT: nuclear mitochondrial DNA segment; OTU: operational taxonomic unit; PERMANOVA: permutational multivariate analysis of variance; SRA: Sequence Read Archive; UMI: unique molecular identifier.

## Funding

This study was enabled by awards to P.D.N.H. from the Ontario Ministry of Research, Innovation and Science (RE07-002), the Canada Foundation for Innovation, and by a grant from the Canada First Research Excellence Fund to the University of Guelph's “Food From Thought” research program.

## Authors' Contributions

D.S., E.V.Z., J.R.D.W., and P.D.N.H. designed the study. D.S., J.R.D.W., J.E.S., and K.P. coordinated the study. S.L.D.W., N.V.I., S.W.J.P., and T.W.A.B. did the bench work and contributed to analyses. S.R. and M.M. oversaw database organization. D.S. did the analyses and wrote the manuscript. P.D.N.H., J.R.D.W., E.V.Z., and T.W.A.B. revised the manuscript.
