# Supplementary material for: Message in a Bottle—Metabarcoding enables biodiversity comparisons across ecoregions
Source: Gigascience. 2022 Apr 28;11:giac040. doi: 10.1093/gigascience/giac040 (PMC9049109; doi:10.1093/gigascience/giac040)
Supplement: giac040_GIGA-D-21-00198_Original_Submission [file giac040_giga-d-21-00198_original_submission.pdf]

GigaScience

Message in a Bottle – Metabarcoding Enables Biodiversity Comparisons Across Ecoregions

--Manuscript Draft--

**Manuscript Number:**	GIGA-D-21-00198
**Full Title:**	Message in a Bottle – Metabarcoding Enables Biodiversity Comparisons Across Ecoregions
**Article Type:**	Research
**Funding Information:**	ontario ministry of research, innovation and science — Dr Paul DN Hebert<br>canada foundation for innovation — Dr Paul DN Hebert<br>canada first research excellence fund — Dr Paul DN Hebert
**Abstract:**	Background<br><br>Traditional biomonitoring approaches have delivered a basic understanding of biodiversity, but they cannot support the large-scale assessments required to manage and protect entire ecosystems. This study employed DNA metabarcoding to assess spatial and temporal variation in species richness and diversity in arthropod communities from 52 protected areas spanning three Canadian ecoregions.<br><br>Results<br><br>This study revealed the presence of 26,263 arthropod species in the three ecoregions and indicated that at least another 3,000–5,000 await detection. Results further demonstrate that communities are more similar within than between ecoregions, even after controlling for geographical distance. Overall α-diversity declined from east to west, reflecting a gradient in habitat disturbance. Shifts in species composition were high at every site with turnover greater than nestedness, suggesting the presence of many transient species.<br><br>Conclusions<br><br>Differences in species composition among their arthropod communities confirm that ecoregions are a useful synoptic for biogeographic patterns and for structuring conservation efforts. The present results also demonstrate that metabarcoding enables large-scale monitoring of shifts in species composition, making it possible to move beyond the biomass measurements that have been the key metric employed in prior efforts to track change in arthropod communities.
**Corresponding Author:**	Dirk Steinke, Dr. rer. nat.<br>University of Guelph<br>Guelph, Ontario CANADA
**Corresponding Author Secondary Information:**	
**Corresponding Author's Institution:**	University of Guelph
**Corresponding Author's Secondary Institution:**	
**First Author:**	Dirk Steinke, Dr. rer. nat.
**First Author Secondary Information:**	
**Order of Authors:**	Dirk Steinke, Dr. rer. nat.<br>Stephanie L deWaard<br>Jayme E Sones<br>Natalia V Ivanova<br>Sean SW Prosser

Powered by Editorial Manager® and ProduXion Manager® from Aries Systems Corporation

	Kate Perez
	Thomas Wa Braukmann
	Megan Milton
	Evgeny V Zakharov
	Jeremy R deWaard
	Sujeevan Ratnasingham
	Paul DN Hebert
Order of Authors Secondary Information:	
Additional Information:	
**Question**	**Response**
Are you submitting this manuscript to a special series or article collection?	No
**Experimental design and statistics**<br><br>Full details of the experimental design and statistical methods used should be given in the Methods section, as detailed in our Minimum Standards Reporting Checklist. Information essential to interpreting the data presented should be made available in the figure legends.<br><br>Have you included all the information requested in your manuscript?	Yes
**Resources**<br><br>A description of all resources used, including antibodies, cell lines, animals and software tools, with enough information to allow them to be uniquely identified, should be included in the Methods section. Authors are strongly encouraged to cite Research Resource Identifiers (RRIDs) for antibodies, model organisms and tools, where possible.<br><br>Have you included the information requested as detailed in our Minimum Standards Reporting Checklist?	Yes
**Availability of data and materials**	Yes

All datasets and code on which the conclusions of the paper rely must be either included in your submission or deposited in publicly available repositories (where available and ethically appropriate), referencing such data using a unique identifier in the references and in the "Availability of Data and Materials" section of your manuscript.<br><br>Have you have met the above requirement as detailed in our Minimum Standards Reporting Checklist?	

Manuscript
Click here to access/download;Manuscript;OPP_ecoregions_final_Gigascienc

For submission to *GigaScience*

Message in a Bottle – Metabarcoding Enables Biodiversity Comparisons Across Ecoregions

Steinke D[1,2*], deWaard SL[1], Sones, JE[1], Ivanova NV[1,2], Prosser SWJ[1], Perez K[1], Braukmann TWA[1], Milton M[1], Zakharov EV[1,2], deWaard JR[1,3], Ratnasingham S [1,2] Hebert PDN[1,2]

Affiliations:
[1]Centre for Biodiversity Genomics, University of Guelph, 50 Stone Road East, Guelph, Ontario, N1G 2W1, Canada
[2]Department of Integrative Biology, University of Guelph, 50 Stone Road East, Guelph, Ontario, N1G 2W1, Canada
[3]School of Environmental Sciences, University of Guelph, 50 Stone Road East, Guelph, Ontario, N1G 2W1, Canada

*Corresponding author: Dirk Steinke (dsteinke@uoguelph.ca)

Abstract

Background

Traditional biomonitoring approaches have delivered a basic understanding of biodiversity, but they cannot support the large-scale assessments required to manage and protect entire ecosystems. This study employed DNA metabarcoding to assess spatial and temporal variation in species richness and diversity in arthropod communities from 52 protected areas spanning three Canadian ecoregions.

Results

This study revealed the presence of 26,263 arthropod species in the three ecoregions and indicated that at least another 3,000–5,000 await detection. Results further demonstrate that communities are more similar within than between ecoregions, even after controlling for geographical distance. Overall α-diversity declined from east to west, reflecting a gradient in habitat disturbance. Shifts in species composition were high at every site with turnover greater than nestedness, suggesting the presence of many transient species.

Conclusions

Differences in species composition among their arthropod communities confirm that ecoregions are a useful synoptic for biogeographic patterns and for structuring conservation efforts. The present results also demonstrate that metabarcoding enables large-scale monitoring of shifts in species composition, making it possible to move beyond the biomass measurements that have been the key metric employed in prior efforts to track change in arthropod communities.

Background

Terrestrial organisms are exposed to diverse anthropogenic stressors, including climate change, resource extraction, and agriculture. Habitat degradation, pesticide usage, invasive species, and associated shifts in food webs have provoked major reductions in the abundance of terrestrial arthropods [1-4]. These declines have led to calls for more comprehensive biosurveillance to inform environmental management and conservation. Long-term monitoring of species composition is essential to quantify biological change, but efforts employing morphological diagnostics have targeted a small set of indicator species because of the need for taxonomic experts for each group. As a consequence, they cannot support the broad assessments needed to manage and protect ecosystems, let alone forecast human impacts on them by integrating statistical modelling. The latter methods demand comprehensive data on species distributions and abundance [5], information that is currently unavailable because of the prior focus on selected biotic compartments at limited geographic scale.

Two methodological advances promise to meet the need for comprehensive biodiversity data. Firstly, identification systems based on the analysis of sequence variation in short, standardized gene regions (i.e., DNA barcodes) enable species discrimination [6]. Secondly, high-throughput sequencers (HTS) permit the inexpensive acquisition of millions of DNA barcode records [7]. These advances now enable biodiversity surveys at speeds and scales that were previously inconceivable. In particular, the coupling of HTS with DNA barcoding, known as metabarcoding [8], has a compelling advantage over traditional approaches for tracking shifts in species presence. It can generate georeferenced occurrence data from bulk samples at low cost, and a single instrument can process hundreds of bulk samples each week. Because the sequencing output of HTS is doubling every nine months [9,10], analytical costs are certain to sharply decline, allowing production to soar. This augmented capacity for data generation has already enabled large-scale biotic surveys of aquatic and terrestrial arthropods [11-14], vertebrates [15], pollen [16], diatoms [17], and fungi [18-20].

Access to large collections of specimens is essential to capitalize on the analytical capacity provided by DNA metabarcoding. Among the many approaches used to sample terrestrial arthropods, Malaise traps [21] have gained wide adoption because they collect large, diverse samples with little effort [22]. Although most-effective for sampling flying insects, they also collect ground-active arthropods. By coupling DNA barcoding with Malaise trapping [23,24], high-resolution monitoring networks for arthropods are within reach, but there are challenges. Data interpretation requires a well-parameterized DNA barcode reference library for the region under investigation, creating the need for a system to aid site selection. Ecoregions represent an obvious candidate [25-28] although their boundaries are rarely sharply defined, and they are based on distributional data for a narrow range of taxa. Despite these limitations, ecoregions have been widely used to guide management decisions and to explore species and community diversity patterns [29,30]. As a result, they are a good candidate to serve as the backbone for a large-scale monitoring network. The most widely adopted schema partitions the world's 14 terrestrial biomes into 846 ecoregions [30].

This study demonstrates the feasibility of employing metabarcoding for large-scale bio-surveillance by comparing the temporal and spatial patterning of arthropod communities in three of Canada's 47 terrestrial ecoregions: the Eastern Canadian Forest – Boreal Transition (ECF – 75,000 km^2), the Eastern Great Lakes Lowland Forests (EGL – 63,000 km^2), and the Southern Great Lakes Forests (SGL – 22,000 km^2) (Figure 1). Forest cover declines from 77.7% in the ECF to 30.1% in the EGL and just 12.1% in the SGL while cropland/pastures cover 78% of the SGL, 57% of the EGL, and 3% of the ECF [31]. The EGL and SGL are the most populated ecoregions in Ontario with developed land (e.g., urban, road networks) encompassing more than 7% of the SGL [31]. As such, these ecoregions provide a good basis for assessing the impacts of varied disturbance regimes on biodiversity.

Data Description

Collections were made by deploying a Malaise trap at 52 sites in these three ecoregions and samples were metabarcoded to examine variation in their species richness, community

composition, phylogenetic diversity, as well as alpha (α) and beta (β)-diversity. Malaise traps were deployed for 20 weeks at 15 sites in the ECF, 24 sites in the EGL, and 13 sites in the SGL. Catches were harvested at two-week intervals and 410 of the resultant 520 samples were designated for metabarcoding (the others were reserved for single specimen barcoding). Analysis began with non-destructive lysis of the specimens in each bi-weekly sample, followed by DNA extraction using a membrane-based protocol [32]. A 463 bp amplicon of cytochrome *c* oxidase I (COI) was then PCR amplified and the amplicon pools from each set of 10 samples were sequenced on an Ion Torrent S5 using a 530 chip. The sequences were subsequently analyzed using the Multiplex Barcode Research And Visualization Environment (mBRAVE – mbrave.net). All raw HTS datasets were deposited in the Sequence Read Archive (SRA – www.ncbi.nlm.nih.gov/sra/) under the BioProject accession number PRJNA629553.

Analyses

Sequence analysis of the 410 samples produced 367,823,207 reads across 41 S5 runs (mean reads per run = 8.97 million, see **Table S1**). Two thirds were filtered, leaving 126,253,260 reads that could be assigned to a BIN (Barcode Index Number; [33]) on BOLD [34] (**Figure S1**). Nearly all reads (99.3 %) found a BIN match on BOLD, but those that failed were *de novo* clustered using mBRAVE with a 99% similarity threshold. The latter analysis recognized an average of 28 additional OTUs per sample, but >96% of them reflected sequencing/PCR errors (e.g., chimeras, sequences with multiple indels) or NUMTs so they were excluded from further analysis. Consideration of the assigned reads revealed 26,263 BINs among the 52 sites with more than a third (9,301) found at only one site (**Figure 2b**).

The Chao 1 [35] estimate for the total number of BINs present at the 52 sites was 29,640 (**Figure 2a**) while species richness extrapolation based on the lognormal distribution (**Figure 2c**, [36]) suggested the presence of 31,516 BINs. On average, 0.3 million sequences were recovered per sample, and they revealed the presence of an average of 2,352 BINs per site (range 996–4,581 BINs, **Table S2**) with bi-weekly samples containing an average of 619±14.3 S.E. BINs (range 60–1666, **Table S3**). Most low BIN

counts occurred in spring (May) or fall (September) with diversity peaking in mid-summer (June/July). Taxonomic composition at an ordinal level was similar among samples with over half of the BINs being flies (Diptera), followed by Hymenoptera, Lepidoptera, Hemiptera, and Coleoptera.

Overlap in BIN composition was higher among parks in an ecoregion than among those in different ecoregions, even after geographical distance was considered (**Figure 3a**). Sites in the ECF had the highest mean phylogenetic diversity followed by EGL and finally SGL (**Figure 3b**), differences that were significant (KW and Dunn's posthoc $p < 0.003$). More BINs were collected in the ECF (14,001) than in the EGL (12,787) or SGL (10,958) (Figure 3c). The Chao 1 estimates for the number of BINs present in each ecoregion were 15,401 for ECF, 14,577 for EGL, and 12,602 for SGL. The three ecoregions shared 4,133 BINs while about a third of those in each region were not collected elsewhere. A two-dimensional NMDS Ordination plot revealed that BIN assemblages for sites in each ecoregion formed cohesive groupings (Figure 3d). PERMANOVA analysis also suggested that community structure varied between ecoregions ($R^2 = 0.141$, $P = 0.0001$) and decreased site elevation ($R^2 = 0.035$, $P = 0.03$).

Overall, α-diversity was highest in the ECF, intermediate in the EGL, and lowest in SGL (**Figure 4**). The α-diversity patterns for the varied insect orders followed the overall trend, but BIN richness for Collembola showed the opposite trend as it peaked in the SGL, while spider α-diversity was highest in the EGL.

Levels of turnover (**Figure 5**) were generally high among sites (species replacement by new species not found elsewhere) as well as high nestedness levels (gain and loss of species also found elsewhere). Lower levels of both turnover and nestedness were observed for most taxa at sites in the ECF while the highest values were found in the SGL.

Discussion

This study used metabarcoding to examine the species represented in 410 Malaise trap samples derived from 52 protected sites in three juxtaposed Canadian ecoregions.

Metabarcoding revealed 26,263 species of arthropods while Chao 1 and Preston lognormal extrapolations indicated that another 3,000–5,000 species await detection. As just 52 sites were surveyed, a more comprehensive sampling program in these ecoregions might reveal as many as 50,000 species of arthropods. Nearly 5-fold variation (996–4,581) in BIN counts were detected among sites; counts showed a similar range for the 30 sites where all samples were analyzed (996–4,508) and the 22 where just half were metabarcoded (1,312–4,581). On average, 619 BINs were recovered from each metabarcoded sample, a count that was 52.5% higher than the mean BIN count (406) for samples that were barcoded (Steinke et al. in prep). This difference suggests that more than half the BINs recovered from metabarcoded samples derive from environmental DNA attached to specimens in the sample or from their gut contents.

The three ecoregions examined in this study collectively span 160,000 km^2, just 1.6% of Canada's land surface, but two (SGL, EGL) are among the most heavily populated areas in the country [31]. The ecoregions showed considerable overlap in species composition; 33.1% of the BINs recorded from three or more sites were shared by the three ecoregions. BIN richness was lowest in the southernmost ecoregion (SGL) and highest in the most northerly (ECF). This difference coincided with a disturbance gradient -- from forested regions with low human density in the ECF (78% forest cover) to disturbed landscapes dominated by farmland/cities in the SGL (12% forest cover). The decline in species richness in response to disturbance is consistent with earlier studies [37-39], even though our collections all derived from protected areas. [40] reported that protected sites contain significantly higher species counts than adjacent disturbed areas, perhaps because communities in protected areas include representatives of original habitats and generalists from adjacent disturbed landscapes [41]. However, protected areas in the SGL were small islands of remnant forest in a landscape dominated by agricultural activity so they were undoubtedly heavily exposed to pesticides with agricultural fields creating dispersal barriers which further reduced diversity.

Our results indicate that α-diversity for major insect orders of flying insects (Diptera, Hymenoptera, Hemiptera, Lepidoptera) peaked in the least disturbed ecoregion (ECF). By

contrast, two groups of arthropods (Araneae, Collembola) lacking flight showed a different trend with their diversity peaking in other ecoregions. This difference might reflect the fact that Malaise traps only sample flightless taxa with resident populations near the trap but capture flying insects from distant habitats. As such, biodiversity patterns for flying insects provide a regional perspective while those for taxa without flight provide a local perspective. If so, the reduction in diversity of Collembola from the most southerly (SGL) to northerly (ECF) ecoregion might reflect the expected latitudinal gradient in biodiversity, undisrupted by disturbance because of the local source of specimens in each sample.

The present study establishes the feasibility of monitoring temporal changes in species composition of arthropod communities [42,43]. For all three ecoregions, temporal turnover was high, reflecting the seasonal succession of species. β-diversity was lowest for most taxonomic groups at sites in the ECF and highest in the SGL. Species turnover was generally higher than nestedness, suggesting the presence of many transient species [44]. As many species were only collected at one or two sites, many samples likely included transients passively transported by the wind [45].

Metabarcoding can already provide cost-effective biosurveillance as the present study analyzed about 856,000 specimens and generated 223,860 species occurrence records for $82,000, an analytical cost of less than $0.50 per record. By adopting simpler analytical protocols (e.g., destructive processing of samples) with ongoing reductions in sequencing costs [10], costs can be reduced by an order of magnitude, delivering species occurrence records for $0.04 apiece in the ecoregions targeted in this study. In settings with higher α-diversity, the cost could be halved. Aside from its cost-effectiveness for data acquisition, the digital format of metabarcoding results aids their curation, validation, and preservation. Although current metabarcoding protocols cannot estimate the abundance of each species in a sample, the situation shifts when multiple samples are analyzed as the abundance of a species can then be estimated from its frequency of occurrence in these samples (rare species will be recovered less frequently than abundant taxa).

As the 846 currently recognized ecoregions [30] were largely delineated based on distributional data for vascular plants and vertebrates, there remains a need to ascertain how well they represent diversity patterns in other taxa. [46] found that arthropods showed weak adherence to ecoregion boundaries and proposed this might reflect dispersal limitations linked to their small body size or to the biased assemblage of arthropod species with data. Our much larger dataset shows evidence of structuring by ecoregion as both phylogenetic diversity and BIN composition were significantly different among ecoregions, even when comparisons extended to widely separated sites. This result suggests that ecoregions do provide a useful structural framework, reinforcing results from earlier studies [47,48]. However, a third of species in this study crossed ecoregion boundaries and more extensive sampling would raise the incidence of shared species. The latter results make it clear that high sampling effort is required to better understand species distributions. In looking to the future, it is apparent that there is an immediate need for a more detailed understanding of the levels of species overlap between adjacent ecoregions. Is, for example, the pattern of high overlap in species composition among neighbouring ecoregions detected in this study a general pattern or are some ecoregion boundaries sharply delineated? Such information is critical in designing an effective global biomonitoring network to inform conservation efforts [49,50].

Potential Implications

Past monitoring programs have provided limited insights into the shifting distributions and abundances of arthropod species [51]. By coupling the use of an efficient collection method with the capacity of DNA metabarcoding to determine the species composition of bulk samples, this study has shown that compositional shifts in arthropod communities can be tracked [52]. The present results also indicate that the ecoregion concept not only furthers understanding of foundational biogeographic principles and improves their potential application to conservation efforts, but also provides a logical scaffold for large-scale monitoring networks.

Methods

Sample collection

An ez-Malaise trap (BioQuip Products) was deployed to collect arthropods at one site in each of 50 provincial parks while two sites were sampled in the final park (Algonquin) because of its large size. Trap catches were harvested every second week from early May through September, producing 10 samples per site for a total of 520 samples. These samples were preserved in 95% ethanol and held at -20° C until DNA extraction. Five samples (weeks 1+2, 5+6, 9+10, 13+14, 17+18) from each of 22 sites were employed for single specimen barcoding (Steinke et al., in prep) while the other 410 samples were analyzed in this study. A direct count indicated that 230,000 specimens were present in the 21.2% of the samples that were barcoded. On this basis, the remaining samples (78.8%), those examined in this study, included about 856,000 specimens.

DNA extraction and PCR

DNA extraction employed a membrane-based protocol [32] modified for bulk samples. Specimens were removed from ethanol by filtration through a sterile Microfunnel 0.45 µM Supor Membrane Filter (Pall Laboratory) using a 6-Funnel Manifold (Pall Laboratory). The wet weight of each sample was then ascertained to allow volume adjustment (**Table S4**) of the lysis buffer [32]. Each sample was then incubated overnight at 56°C while gently mixed on a shaker. Eight 50 µl aliquots (technical replicates) from each of the 410 lysates were then transferred into 3,280 separate wells in 96-well microplates and DNA extracts were generated using Acroprep 3.0 µm glass fiber/0.2 µm Bio-Inert membrane plates (Pall Laboratory). Each plate contained 80 lysate samples, 8 technical replicates of a positive control (lysate from a bulk sample whose component specimens were individually Sanger sequenced – public BOLD dataset - dx.doi.org/10.5883/DS-RRNGS) and 8 negative controls. Each lysate was mixed with 100 µl of binding mix, transferred to a column plate, and centrifuged at 5000 g for 5 min. DNA was then purified with three washes; the first employed 180 µl of protein wash buffer centrifuged at 5000 g for 5 min. Each column was then washed twice with 600 µl of wash buffer centrifuged at 5000 g for 5 min. Columns were transferred to clean tubes and spun dry at 5000 g for 5 min to remove residual buffer before their transfer to clean collection

tubes followed by incubation for 30 min at 56°C to dry the membrane. DNA was subsequently eluted by adding 60 µl of 10 mM Tris-HCl pH 8.0 followed by centrifugation at 5000 g for 5 min.

PCR reactions employed a standard protocol [53]. Briefly, each reaction included 5% trehalose (Fluka Analytical), 1× Platinum Taq reaction buffer (Invitrogen), 2.5 mM $MgCl_2$ (Invitrogen), 0.1 µM of each primer (Integrated DNA Technologies), 50 µM of each dNTP (KAPA Biosystems), 0.3 units of Platinum Taq (Invitrogen), 2 µl of DNA extract, and Hyclone ultra-pure water (Thermo Scientific) for a final volume of 12.5 µl. Two-stage PCR was used to generate amplicon libraries for sequencing on an Ion Torrent S5 platform. The first round of PCR used the primer combination AncientLepF3 [54] and LepR1 [55] to amplify a 463 bp fragment of COI. Prior to the second PCR, first round products were diluted 2x with ddH_2O. Fusion primers were then used to attach platform-specific unique molecular identifiers (UMIs) along with the sequencing adaptors required for Ion Torrent S5 libraries. Both rounds of PCR employed the same thermocycling conditions: initial denaturation at 94 °C for 2 min, followed by 20 cycles of denaturation at 94°C for 40 sec, annealing at 51°C for 1 min, and extension at 72 °C for 1 min, with a final extension at 72°C of 5 min.

HTS library construction

For each plate, labelled products were pooled prior to sequencing. In total, 41 libraries were assembled. Each included eight technical replicates of 10 samples plus eight technical replicates of a negative and a positive control respectively (i.e., 96 samples). The ten samples from each of the 30 sites that were only metabarcoded, together with positive and negative controls, were pooled after UMI tagging to create a library that was analyzed on a 530 chip (30 chips in total). Five samples were available from each the other 22 sites (where half the samples were retained for barcoding). The UMI-tagged amplicons from five samples from each of two sites were pooled with positive and negative controls to produce a single library. Amplicon libraries were prepared on an Ion Chef (Thermo Fisher Scientific) following and sequenced on an Ion Torrent S5 platform at the Centre for Biodiversity Genomics following manufacturer's instructions (Thermo Fisher Scientific).

Sequence analysis

Reads from the eight replicates for each sample were concatenated using a bash script and uploaded to mBRAVE (http://mbrave.net/) for quality filtering and subsequent queries using several reference libraries in an open reference approach. All reads were queried against five system libraries on mBRAVE: bacteria (SYS-CRLBACTERIA), chordates (SYS-CRLCHORDATA), insects (SYS-CRLINSECTA), non-insect arthropods (SYS-CRLNONINSECTARTH), and non-arthropod invertebrates (SYS-CRLNONARTHINVERT). Sequences were only included in this analysis if they possessed a minimum length >350 bp and met the following three quality criteria (Mean QV >20; <25% positions with a QV<20; <5% positions with QV<10). Reads were trimmed 30 bp from their 5' terminus with a set trim length of 450 bp. Reads were matched to the sequences in each reference library with an ID distance threshold of 3%, but were only retained for further analysis when at least three reads matched an OTU in the reference database. All reads failing to match any sequence in the five reference libraries were clustered at an OTU threshold of 1% with a minimum of five reads per cluster. All raw data are available in the NCBI Short Read Archive (PRJNA629553).

Using mBRAVE, we generated BIN (and OTU) tables including all library queries for each individual plate/run (10 samples, plus a negative and positive control - dx.doi.org/10.5883/DS-RRNGS - for each run). Read counts for any BINs recovered from the negative control on a plate were subtracted from the counts for the same BIN in the 80 non-control wells in the run. When this subtraction reduced the read count for a BIN to zero, its occurrence was removed. This step reduced the effects of rare tag switching on data integrity [56] and removed any background contamination.

Ecoregion analysis

To determine the completeness of sampling, we calculated accumulation curves and the Chao-1 estimator for total diversity [35] using the vegan package [57]. For further extrapolation of species richness, we used the lognormal species abundance distribution [36]. The fit of Fisher's Logseries [58] was used to determine relative BIN abundance. Both methods are implemented in vegan (fisherfit, prestonfit) [57]. We calculated

Sørensen's similarity coefficient to ascertain if differences in species assemblages were greater between or across ecoregion borders after controlling for distance. Differences in BIN composition among the three ecoregions were examined using non-metric multidimensional scaling (NMDS) with the Bray-Curtis index coefficient as implemented in vegan [57]. The adonis function of the vegan package was used to conduct a Permutational Multivariate Analysis of Variance (PERMANOVA) to partition distance matrices among sources of variation (factors such as latitude, longitude, elevation, and ecoregion).

A Maximum likelihood phylogeny was inferred for a BIN sequence alignment using RAxML Black box [59] on XCEDE via the CIPRES portal [60]. The resulting phylogeny comprising 26,263 BIN sequences was used to calculate Faith's phylogenetic distance (PD) [61] using the picante package [62]. Because this measure is influenced by polytomies in a phylogeny [63], only one representative was included per BIN to avoid bias introduced by variation in the number of records for each BIN. A Kruskal-Wallis test followed by a Dunn's posthoc analysis was used to determine if significant PD differences existed between ecoregions.

Alpha (α)-diversity was quantified as the number of BINs observed at a site. Beta (β)-diversity was computed as multi-site Sorensen and Simpson indices using the betapart 1.3. package [64]. β-diversity calculations between pairs of ecoregions were computed using 12 random sites from the total sites for each ecoregion, and resampled 1000 times. We then decomposed the among-site β-diversity into its turnover (species replacement from site to site) and nestedness (species gain/loss from sites) components. Pairwise BIN diversity among ecoregions was evaluated using the nonparametric multiple comparison function implemented in the R package dunn.test 1.2.4 [65]. dunn.test is equivalent to the Kruskall–Wallis and pair-wise Mann–Whitney post hoc tests with Bonferroni correction.

All analyses were performed in R v.3.4.4 [66].

Data availability

All raw HTS datasets are deposited in the Sequence Read Archive (SRA – www.ncbi.nlm.nih.gov/sra/) under the BioProject accession number PRJNA629553. Additional supporting data and materials are available on the GigaScience database.

Funding

This study was enabled by awards to PDNH from the Ontario Ministry of Research, Innovation and Science, the Canada Foundation for Innovation, and by a grant from the Canada First Research Excellence Fund to the University of Guelph's "Food From Thought" research program.

Author contributions

DS, EVZ, JRDW, PDNH designed the study. DS, JRDW, JES, KP coordinated the study. SLDW, NVI, SWJP, TWAB did the bench work and contributed to analyses. SR and MM oversaw database organisation. DS did the analyses and wrote the manuscript. PDNH, JRDW, EVZ, TWAB revised the manuscript.

Acknowledgements

We thank the collections and sequencing staff at the Centre for Biodiversity Genomics for acquiring and processing the specimens analyzed in this study. We are very grateful to Suz Bateson for improving the figures and to staff at the participating Ontario Provincial Parks for facilitating collections.

References

1. Hallmann CA, Sorg M, Jongejans E, Siepel H, Hofland N, Schwan H, Stenmans W, Müller A, Sumser H, Hörren T, Goulson D, de Kroon H. More than 75 percent decline over 27 years in total flying insect biomass in protected areas. *PLoS ONE*. 2017; 12(10): e0185809.

2. Lister BC, Garcia A. Climate-driven declines in arthropod abundance restructure a rainforest food web. *Proceedings of the National Academy of Sciences of the United States of America*. 2018; 115(44): E10397–E10406.

3. Macgregor CJ, Williams JH, Bell JR, Thomas CD. Moth biomass increases and decreases over 50 years in Britain. *Nature Ecology and Evolution.* 2019; 3: 1645–1649.

4. Seibold S, Gossner MM, Simons NK, Blüthgen N, Müller J, Ambarli D, Ammer C, Bauhus J, Fischer M, Habel JC, Linsenmair KE, Nauss T, Penone C, Prati D, Schall P, Schulze E-D, Vogt J, Wöllauer S, Weisser WW. Arthropod decline in grasslands and forests is associated with drivers at landscape level. *Nature.* 2019; 574: 671–674

5. Bush A, Sollmann R, Wilting A, Bohmann K, Cole B, Balzter H, Martius C, Zlinszky A, Calvignac-Spencer S, Cobbold CA, Dawson TP, Emerson BC, Ferrirer S, Gilbert MTP, Herold M, Jones L, Leendertz FH, Matthews L, Millington JDA, Olson JR, Ovaskainen O, Raffaelli D, Reeve R, Rödel M-O, Rodgers TW, Snape S, Visseren-Hamakers I, Vogler AP, White PCL, Wooster MJ, Yu DW. Connecting Earth observation to high-throughput biodiversity data. *Nature Ecology & Evolution.* 2017; 1: 0176.

6. Hebert PDN, Cywinska A, Ball SL, deWaard JR. Biological identifications through DNA barcodes. *Proceedings of the Royal Society B: Biological Science.* 2003; 270: 313–321.

7. Hebert PDN, Braukmann TWA, Prosser SWJ, Ratnasingham S, deWaard JR, Ivanova NV, Janzen DH, Hallwachs W, Naik S, Sones JE, Zakharov EV. A Sequel to Sanger: amplicon sequencing that scales. *BMC Genomics*. 2018; 19: 219.

8. Taberlet P, Coissac E, Pompanon F, Brochmann C, Willerslev E. Towards next-generation biodiversity assessment using DNA metabarcoding. *Molecular Ecology.* 2012; 21(8): 2045-2050.

9. O'Driscoll A, Daugelaite J, Sleator RD. 'Big Data', Hadoop and cloud computing in genomics. *Journal of Biomedical Informatics.* 2013; 46(5): 774–781.

10. Lightbody G, Haberland V, Browne F, Taggart L, Zheng H, Parkes E, Blayney JK. Review of applications of high-throughput sequencing in personalized medicine: barriers and facilitators of future progress in research and clinical application. *Briefings in Bioinformatics.* 2019; 20(5): 1795–1811.

11. Ji C, Chng KR, Hui Boey EJ, Ng AHQ, Wilm A, Nagarajan N. INC-Seq: accurate single molecule reads using nanopore sequencing. *Gigascience*. 2016; 5: 34.

12. Beng KC, Tomlinson KW, Shen XH, Surget-Groba Y, Hughes AC, Corlett RT, Slik JWF. The utility of DNA metabarcoding for studying the response of arthropod diversity and composition to land-use change in the tropics. *Scientific Reports*. 2016; 6: 1–13.

13. Elbrecht V, Vamos EE, Meissner K, Aroviita J, Leese F. Assessing strengths and weaknesses of DNA metabarcoding-based macroinvertebrate identification for routine stream monitoring. *Methods in Ecology and Evolution.* 2017; 8: 1–21.
14. D'Souza ML, van der Bank M, Zandisile S, Rattray RD, Stewart R, van Rooyen J, Govender D, Hebert PDN. Biodiversity baselines: tracking insects in Kruger National Park with DNA barcodes. *Biological Conservation.* 2021; 256: 109034.
15. Sato H, Sogo Y, Doi H, Yamanaka H. Usefulness and limitations of sample pooling for environmental DNA metabarcoding of freshwater fish communities. *Scientific Reports*. 2017; 7: 14860.
16. Bell KL. Applying pollen DNA metabarcoding to the study of plant-pollinator interactions. *Applications in Plant Sciences*. 2017; 5: apps.1600124
17. Vasselon V, Bouchez A, Rimet F, Jacquet S, Trobajo R, Corniquel M, Tapolczai K, Domaizon I. Avoiding quantification bias in metabarcoding: Application of a cell biovolume correction factor in diatom molecular biomonitoring (A. Mahon, Ed.). *Methods in Ecology and Evolution.* 2018; 9: 1060–1069.
18. Bellemain E, Davey ML, Kauserud H, Epp LS, Boessenkool S, Coissac E, Geml J, Edwards M, Willerslev E, Gussarova G, Taberlet P, Haile J, Brochmann C. Fungal palaeodiversity revealed using high-throughput metabarcoding of ancient DNA from arctic permafrost. *Environmental Microbiology*. 2012; 15: 1176–1189.
19. Aas AB, Davey ML, Kauserud H. ITS all right mama: investigating the formation of chimeric sequences in the ITS2 region by DNA metabarcoding analyses of fungal mock communities of different complexities. *Molecular Ecology Resources*. 2017; 17: 730–741.
20. Tedersoo L, Tooming-Klunderud A, Anslan S. PacBio metabarcoding of Fungi and other eukaryotes: errors, biases, and perspectives. New Phytologist. 2018; 217: 1370–1385.
21. Malaise R. A new insect trap. *Entomologisk Tidskrift*. 1937; 58: 148–160.
22. Karlsson D, Pape T, Johanson KA, Liljeblad J, Ronquist F. The Swedish Malaise Trap Project, or how many species of Hymenoptera and Diptera are there in Sweden? *Entomologisk Tidskrift*. 2005; 126**:** 43–53.
23. deWaard JR, Levesque-Beaudin V, deWaard SL, Ivanova NV, McKeown JTA, Miskie R, Naik S, Perez KHJ, Ratnasingham S, Sobel CN, Sones JE, Steinke C, Telfer AC, Young

A, Young MR, Zakharov EV, Hebert PDN. Expedited assessment of terrestrial arthropod diversity by coupling Malaise traps with DNA barcoding. *Genome*. 2019; 62: 85–95.

24. Steinke D, Braukmann TWA, Manerus L, Woodhouse A, Elbrecht V. Effects of Malaise trap spacing on species richness and composition of terrestrial arthropod bulk samples. *Metabarcoding and Metagenomics*. 2021; 5: 43–50.

25. Holdridge LR. Determination of world plant formations from simple climatic data. *Science*. 1947; 105: 367–368.

26. Whittaker RH. Classification of natural communities. *Botanical Reviews*. 1962; 28: 1–239.

27. Olson DM, Dinerstein E, Wikramanayake ED, Burgess ND, Powell GVN, Underwood EC, D’amico JA, Itoua I, Strand HE, Morrison JC, Loucks CJ, Allnutt TF, Ricketts TH, Kura Y, Lamoreux JF, Wettengel WW, Hedao P, Kassem KR. Terrestrial ecoregions of the world: a new map of life on earth. *Bioscience*. 2001; 51: 933–938.

28. Bailey RG. *Ecoregions*. Springer, New York; 2014.

29. Giakoumi S, Sini M, Gerovasileiou V, Mazor T, Beher J, Possingham HP, Abdulla A, Cinar ME, Dendrinos P, Gucu AC, Karamanlidis AA, Rodic P, Panayotidis P, Taskin E, Jaklin A, Voultsiadou E, Webster C, Zenetos A, Katsanevakis S. Ecoregion-based conservation planning in the Mediterranean: Dealing with large-scale heterogeneity. *PLoS ONE*. 2013; 8(10): e76449.

30. Dinerstein E, Olson D, Joshi A, Vynne C, Burgess ND, Wikramanayake E, Hahn N, Palminteri S, Hedao P, Noss R, Hansen M, Locke H, Ellis EC, Jones B, Barber CV, Hayes R, Kormos C, Martin V, Crist E, Sechrest W, Price L, Baillie JEM, Weeden D, Suckling K, Davis C, Sizer N, Moore R, Thau D, Birch T, Potapov P, Turubanova S, Tyukavina A, de Souza N, Pintea L, Brito JC, Llewellyn OA, Miller AG, Patzelt A, Ghazanfarm SA, Timberlake J, Klöser H, Shennan-Farpón Y, Kindt R, Barnekow Lillesø J-P, van Breugel P, Graudal L, Voge M, Al-Shammari KF, Saleem M. An ecoregion-based approach to protecting half the terrestrial realm. *Bioscience*. 2013; 67: 534–545.

31. Crins WJ, Gray PA, Uhlig PWC, Wester MC. *The Ecosystems of Ontario, Part 1: Ecozones and Ecoregions*. Technical Report SIB TER IMA TR-01, Ministry of Natural Resources, Ontario; 2009.

32. Ivanova NV, deWaard JR, Hebert PDN. An inexpensive, automation-friendly protocol for recovering high-quality DNA. *Molecular Ecology Resources*. 2006; 6: 998–1002.

33. Ratnasingham S and PDN Hebert. A DNA-based registry for all animal species: The Barcode Index Number (BIN) System. *PLoS ONE*. 2013; 8: e66213.

34. Ratnasingham S and PDN Hebert. BOLD: The Barcode of Life Data System (www.barcodinglife.org). *Molecular Ecology Notes*. 2007; 7: 355–364.

35. Magurran AE. *Measuring Biological Diversity*. Wiley-Blackwell, Malden, Massachusetts; 2003.

36. Preston FW. The canonical distribution of commonness and rarity: Part I. *Ecology*. 1962; 43: 185–215.

37. Luke SH, Fayle TM, Eggleton P, Turner EC, Davies RG. Functional structure of ant and termite assemblages in old growth forest, logged forest and oil palm plantation in Malaysian Borneo. *Biodiversity Conservation*. 2014; 23: 2817–2832.

38. Newbold T, Hudson LN, Phillips HRP, Hill SLL, Contu S, Lysenko I, Blandon A, Butchart SHM, Booth HL, Day J, De Palma A, Harrison MLK, Kirkpatrick L, Pynegar E, Robinson A, Simpson J, Mace GM, Scharlemann JPW, Purvis A. A global model of the response of tropical and sub-tropical forest biodiversity to anthropogenic pressures. *Proceedings of the Royal Society B*. 2014; 281: 20141435.

39. Phalan B, Onial M, Balmford A, Green RE. Reconciling food production and biodiversity conservation: Land sharing and land sparing compared. *Science.* 2011; 333: 1289–1291.

40. Gray CL, Hill SLL, Newbold T, Hudson LN, Boerger L, Contu S, Hoskins AJ, Ferrier S, Purvis A, Scharlemann JPW. Local biodiversity is higher inside than outside terrestrial protected areas worldwide. *Nature Communications*. 2016; 7: 12306.

41. Lingbeek BJ, Higgins CL, Muir JP, Kattes DH, Schwertner TW. Arthropod diversity and assemblage structure response to deforestation and desertification in the Sahel of western Senegal. *Global Ecology and Conservation*. 2017; 11: 165–176.

42. Tscharntke T, Tylianakis JM, Rand TA, Didham RK, Fahring L, Batary P, Bengtsson J, Clough Y, Crist TO, Dormann CF, Ewers RM, Fruend J, Holt RD, Holzschuh A, Klein AM, Kleijn D, Kremen C, Landis DA, Laurance W, Lindenmayer D, Scherber C, Sodhi N, Steffan-Dewenter I, Thies C, van der Putten WM, Westphal C. Landscape moderation of

biodiversity patterns and processes – eight hypotheses. *Biological Reviews*. 2012; 87: 661–685.

43. Myers JA, Chase JM, Jiminez I, Jorgensen PM, Araujo-Murakami A, Paniagua-Zambrana N, Seidel R. Beta-diversity in temperate and tropical forests reflects dissimilar mechanisms of community assembly. *Ecology Letters*. 2013; 16: 151–157.

44. Snell Taylor SJ, Evans BS, White EP, Hurlbert AH. The prevalence and impact of transient species in ecological communities. *Ecology*. 2018; 99(8): 1825–1835.

45. D'Souza ML, Hebert PDN. Stable baselines of temporal turnover underlie beta diversity in tropical arthropod communities. *Molecular Ecology*. 2018; 27: 2447–2460.

46. Smith JR, Letten AD, Ke P-J, Anderson CB, Hendershot JN, Dhami MK, Dlott GA, Grainger TN, Howard ME, Morrison BML, Routh D, San Juan PA, Mooney HA, Mordecai EA, Crowther TW, Daily GC. A global test of ecoregions. *Nature Ecology & Evolution*. 2018; 2: 1889–1896.

47. Lightfoot DC, Brantely SL, Allen CD. Geographic patterns of ground-dwelling arthropods across an ecological transition in the North American southwest. *Western North American Naturalist*. 2008; 68: 83–102.

48. Gonzales-Reyes AX, Corronca JA, Arroyo NC. Differences in alpha and beta diversities of epideous arthropod assemblages in two ecoregions of northwestern Argentina. *Zoological Studies*. 2012; 51: 1367–1379.

49. Watson JEM, Venter O. Ecology: a global plan for nature conservation. *Nature*. 2017; 550: 48–49.

50. Wilson EO. *Half-Earth: Our Planet's Fight for Life*, Liveright, New York; 2017.

51. Díaz S, Settele J, Brondízio ES, Ngo HT, Guèze M, Agard J, Arneth A, Balvanera P, Brauman KA, Butchart SHM, Chan KMA, Garibaldi LA, Ichii K, Liu J, Subramanian SM, Midgley GF, Miloslavich P, Molnár Z, Obura D, Pfaff A, Polasky S, Purvis A, Razzaque J, Reyers B, Chowdhury RR, Shin YJ, Visseren-Hamakers IJ, Willis KJ, Zayas CN (eds.). *Summary for policymakers of the global assessment report on biodiversity and ecosystem services of the Intergovernmental Science-Policy Platform on Biodiversity and Ecosystem Services*. IPBES secretariat, Bonn, Germany; 2019.

52. Hobern D. BIOSCAN: DNA barcoding to accelerate taxonomy and biogeography for conservation and sustainability. *Genome*. 2021; 64: 161–164.

53. Braukmann TWA, Prosser SJR, Ivanova NV, Elbrecht V, Steinke D, Ratnasingham R, deWaard JR, Sones JE, Zakharov EV, Hebert PDN. Metabarcoding a diverse arthropod mock community. *Molecular Ecology Resources*. 2019; 19: 711–727.

54. Prosser SWJ, deWaard JR, Miller SE, and PDN Hebert. DNA barcodes from century-old type specimens using next-generation sequencing. *Molecular Ecology Resources*. 2016; 16: 487–497.

55. Hebert PDN, Penton EH, Burns JM, Janzen DH, Hallwachs W. Ten species in one: DNA barcoding reveals cryptic species in the neotropical skipper butterfly *Astraptes fulgerator*. *Proceedings of the National Academy of Sciences of the United States of America*. 2004; 101: 14812–14817.

56. Elbrecht V, Steinke D. Scaling up DNA metabarcoding for freshwater macrozoobenthos monitoring. *Freshwater Biology*. 2018; 64: 380–387.

57. Oksanen J, Blanchet FG, Friendly M, Kindt R, Legendre P, McGlinn D, Minchin PR, O'Hara RB, Simpson GL, Solymos P, Stevens MHH, Szoecs E, Wagner H. *vegan: Community Ecology Package*. R package version 2.5-1. https://CRAN.R-project.org/package=vegan; 2018

58. Fisher RA, Corbet AS, Williams CB. The relation between the number of species and the number of individuals in a random sample of animal population. Journal of Animal Ecology. 1943; 12: 42–58.

59. Stamatakis A, Hoover P, Rougemont J. A rapid bootstrap algorithm for the RAxML web servers. Systematic Biology. 2008; 57(5): 758–771.

60. Miller MA, Pfeiffer W, Schwartz T. The CIPRES science gateway. In: *Proceedings of the 2011 TeraGrid Conference on Extreme Digital Discovery—TG '11*. New York, USA: ACM Press; 2011.

61. Faith DP. Conservation evaluation and phylogenetic diversity. *Biological Conservation*. 1992; 61: 1–10.

62. Kembel SW, Cowan PD, Helmus MR, Cornwell WK, Morlon H, Ackerly DD, Blomberg SP, Webb CO. Picante: R tools for integrating phylogenies and ecology. *Bioinformatics*. 2010; 26(11): 1463–1464.

63. Swenson NG. Phylogenetic resolution and quantifying the phylogenetic diversity and dispersion of communities. *PLoS ONE*. 2009; 4(2): e4390.

64. Baselga A, Orme CDL. betapart: an R package for the study of beta diversity. *Methods Ecology and Evolution*. 2012; 3: 808–812.

65. Dinno A. *dunn.test: Dunn's Test of Multiple Comparisons Using Rank Sums. R package version 1.3.2*. http://CRAN.R-project.org/package= dunn.test; 2016.

66. R Core Team. *R: A language and environment for statistical computing*. R Foundation for Statistical Computing, Vienna, Austria; 2018. URL https://www.R-project.org/.

Figure legends

Figure 1: Map of sampling locations and ecoregion boundaries.

Figure 2: a) BIN accumulation curve for the 410 Malaise trap samples collected in 51 Ontario Provincial Parks. b) Fisher's log series fit to the number of sites where each BIN was observed. c) Preston's lognormal species abundance curve showing the total BINs within each $\log_2$ abundance interval.

Figure 3: BIN compositional differences among three Ontario ecoregions: a) Relationship between geographical distance and mean community similarity (Sørensen's similarity coefficient) within and between ecoregions. b) Boxplots comparing Faith's Phylogenetic Diversity for the three ecoregions. c) Venn diagram depicting BIN overlap among ecoregions. d) Non-metric multidimensional scaling (NMDS) plot using Bray-Curtis index coefficient. Colour coding is based on ecoregion.

Figure 4: Comparison of α-diversity (± s.e.) in three Ontario ecoregions for all BINs and for ten arthropod taxa using 12 random sites from the total sites for each ecoregion. Statistical tests are based on Kruskal–Wallis followed by Mann–Whitney post-hoc comparisons with Bonferroni correction. Significant differences between pairs are indicated with different lowercase characters (a - ECF/EGL, b - ECF/SGL, c - EGL/SGL).

Figure 5: Total β-diversity (solid lines) and turnover (dotted lines) for three Ontario ecoregions. Values were computed using 1000 bootstrap samples of 12 random sites from each ecoregion.

Figure S1: Relationship between filtered read count and number of BINs for 410 metabarcoded samples from three ecoregions.

Figure 1

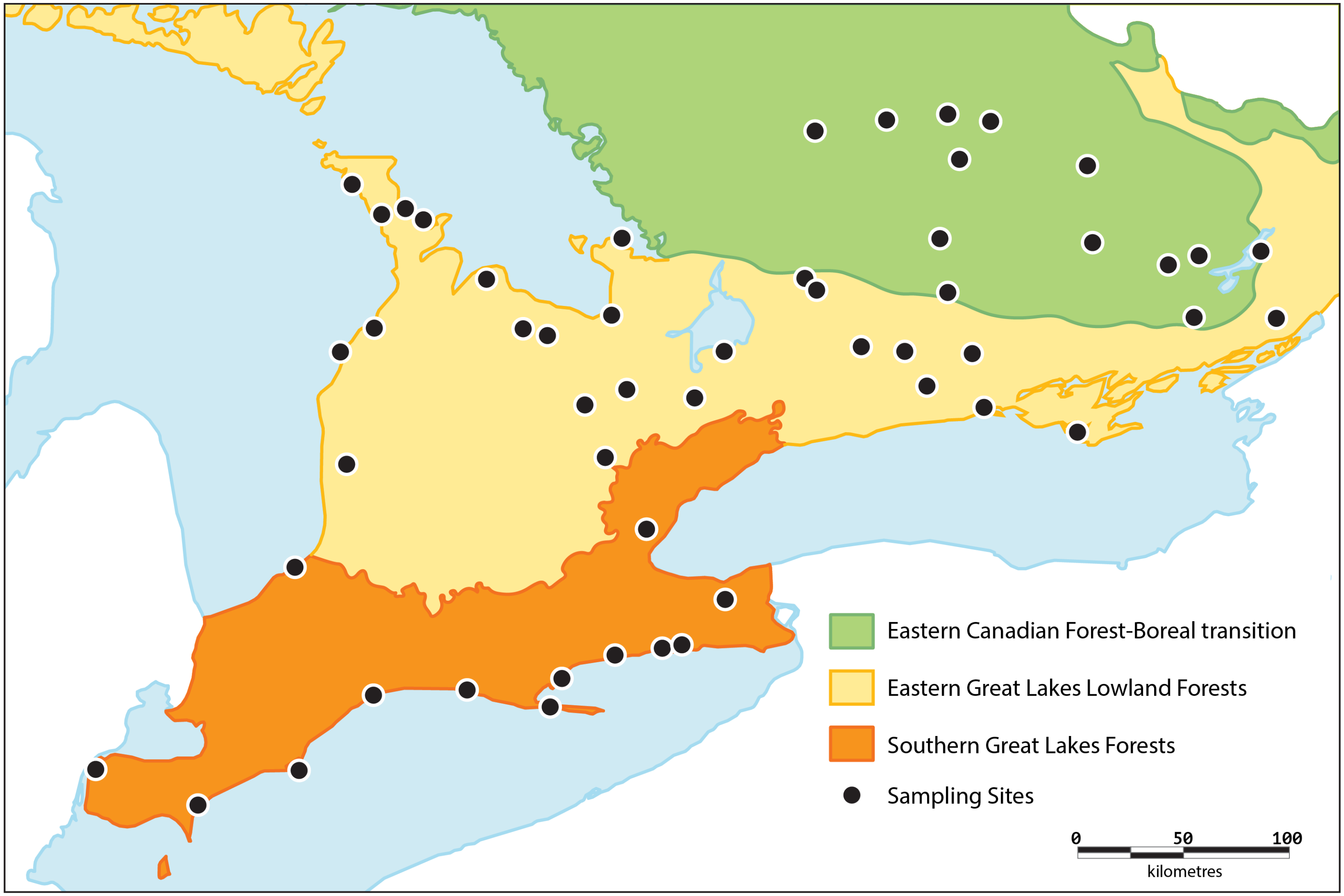
Eastern Canadian Forest-Boreal transition
Eastern Great Lakes Lowland Forests
Southern Great Lakes Forests
Sampling Sites
0
50
100
kilometres

Figure 2

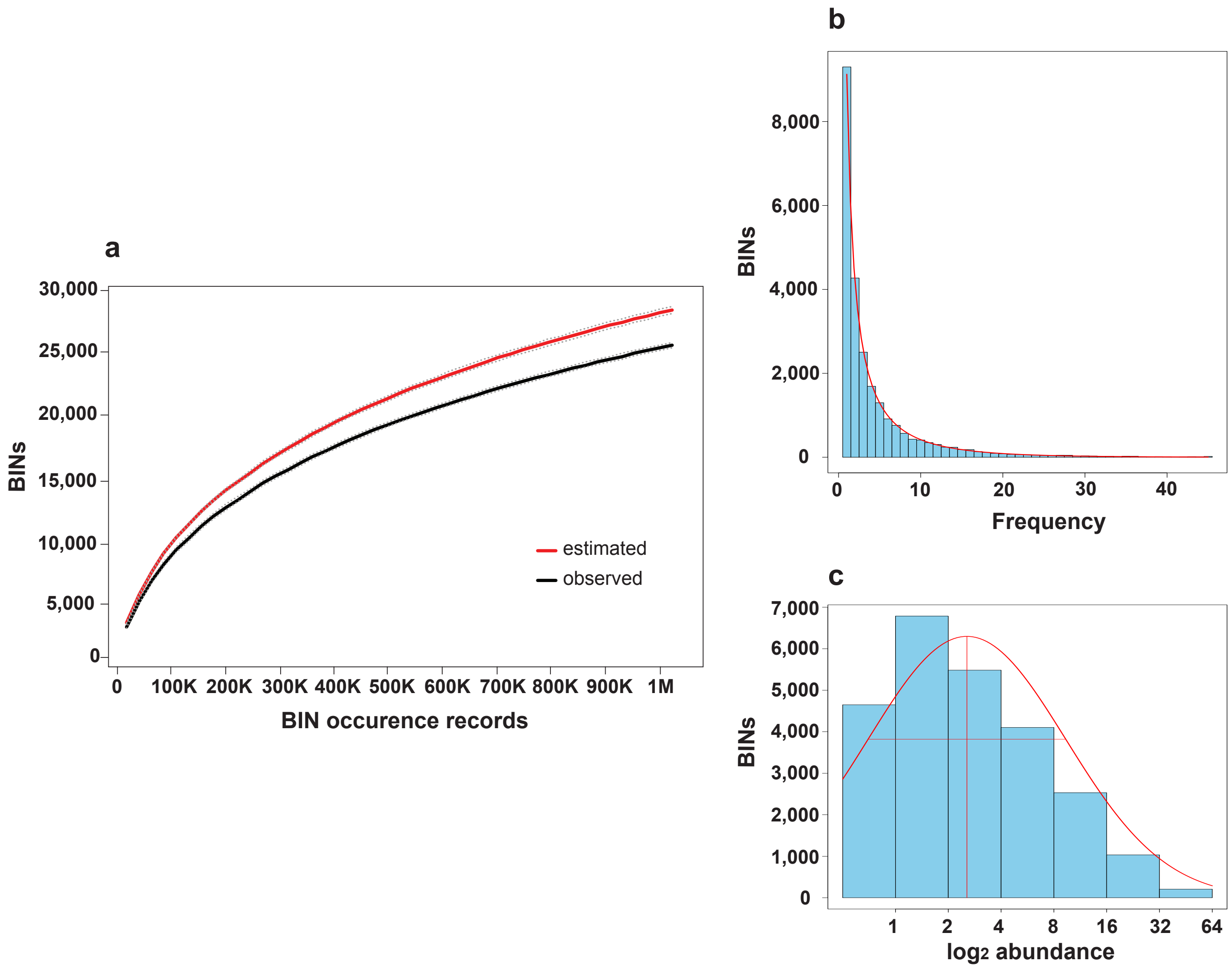
a
30,000
25,000
20,000
15,000
10,000
5,000
0
BINs
estimated
observed
0
100K
200K
300K
400K
500K
600K
700K
800K
900K
1M
BIN occurence records
b
8,000
6,000
4,000
2,000
0
BINs
0
10
20
30
40
Frequency
c
7,000
6,000
5,000
4,000
3,000
2,000
1,000
0
BINs
1
2
4
8
16
32
64
log2 abundance

Figure 3

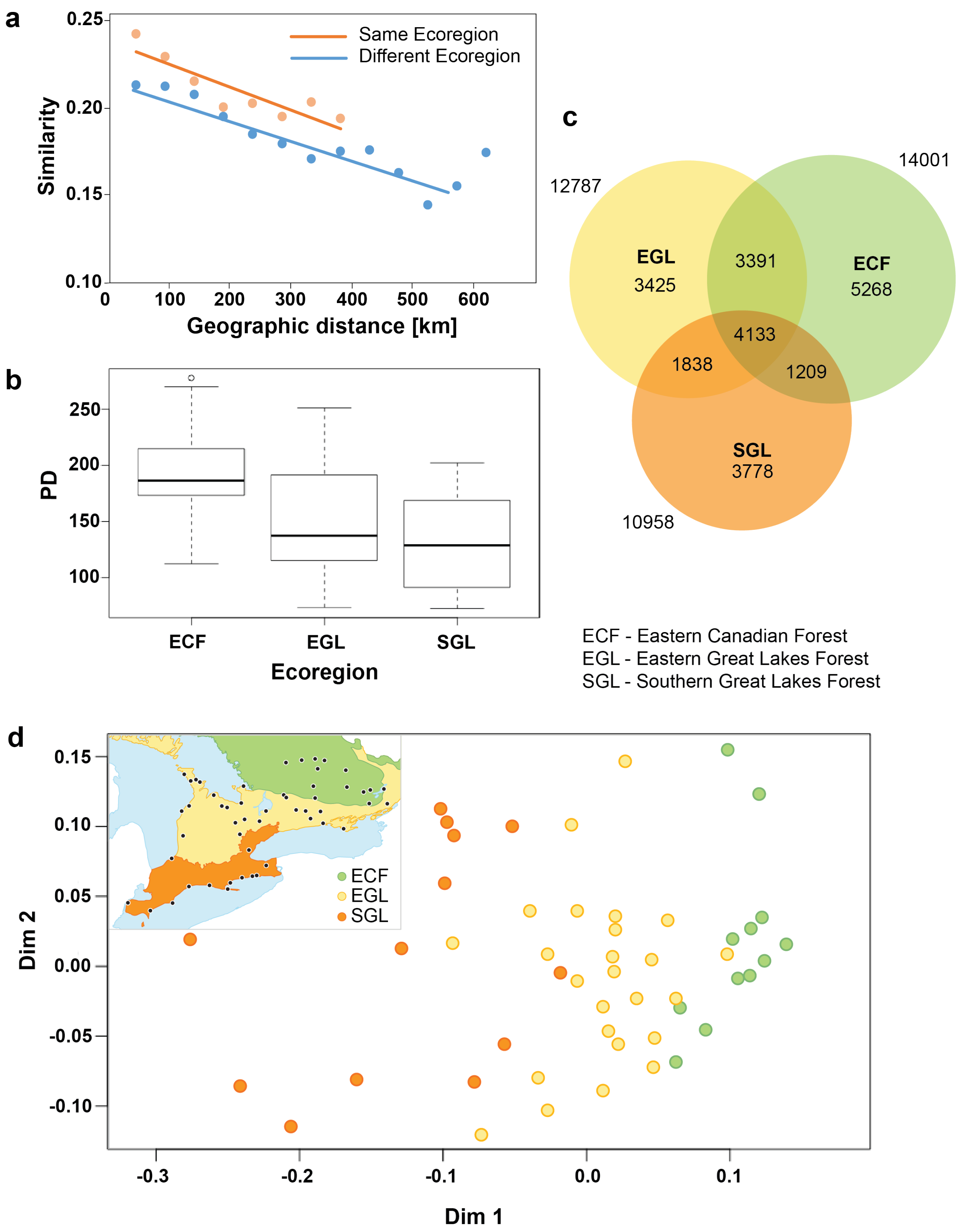
a
Same Ecoregion
Different Ecoregion
Similarity
0.25
0.20
0.15
0.10
0
100
200
300
400
500
600
Geographic distance [km]
b
PD
250
200
150
100
ECF
EGL
SGL
Ecoregion
c
12787
14001
10958
EGL
3425
3391
ECF
5268
4133
1838
1209
SGL
3778
ECF - Eastern Canadian Forest
EGL - Eastern Great Lakes Forest
SGL - Southern Great Lakes Forest
d
ECF
EGL
SGL
Dim 2
0.15
0.10
0.05
0.00
-0.05
-0.10
-0.3
-0.2
-0.1
0.0
0.1
Dim 1

Figure 4

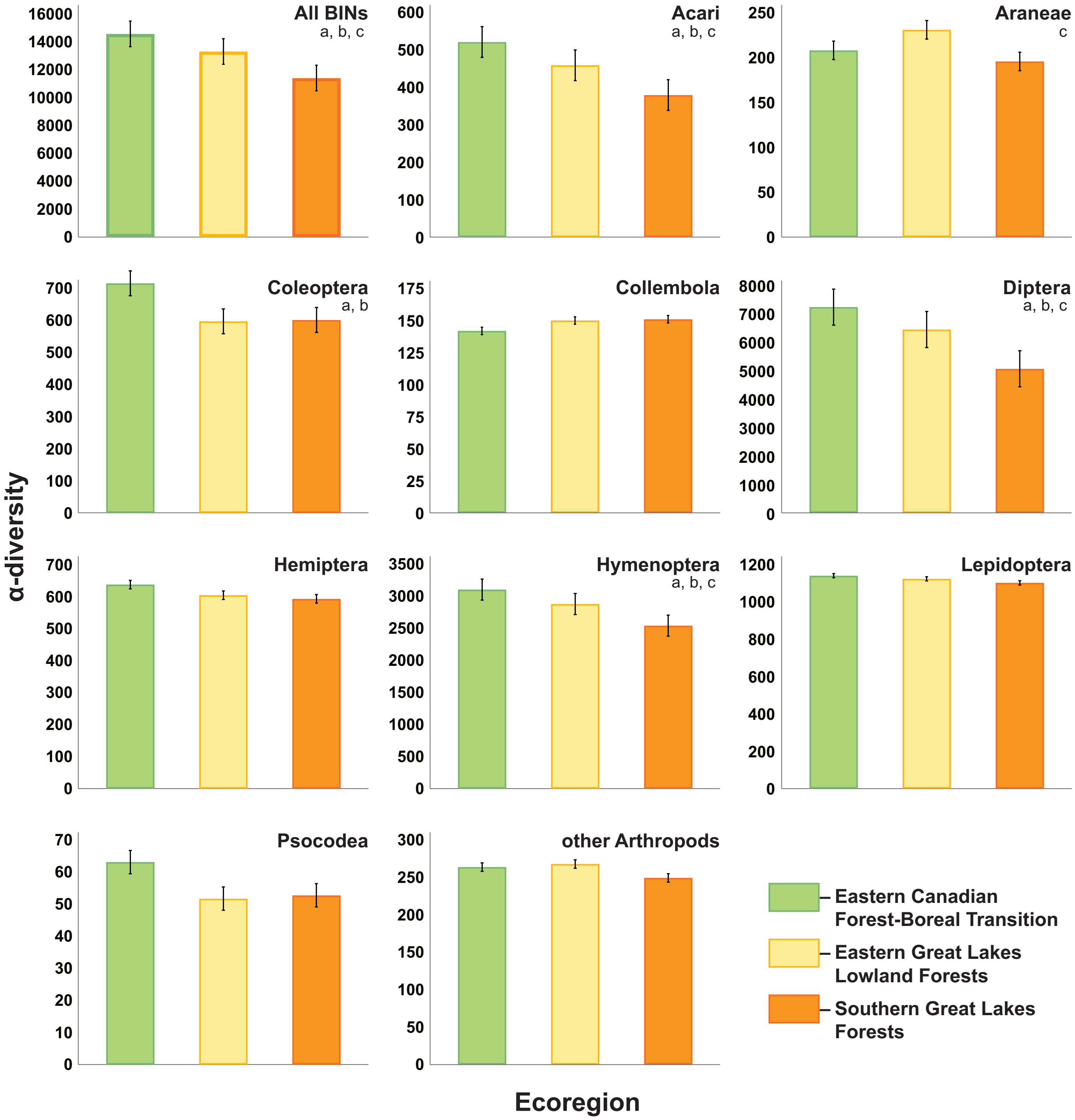
All BINs
a, b, c
Acari
a, b, c
Araneae
c
Coleoptera
a, b
Collembola
Diptera
a, b, c
Hemiptera
Hymenoptera
a, b, c
Lepidoptera
Psocodea
other Arthropods
α-diversity
Ecoregion
Eastern Canadian Forest-Boreal Transition
Eastern Great Lakes Lowland Forests
Southern Great Lakes Forests

Figure 5

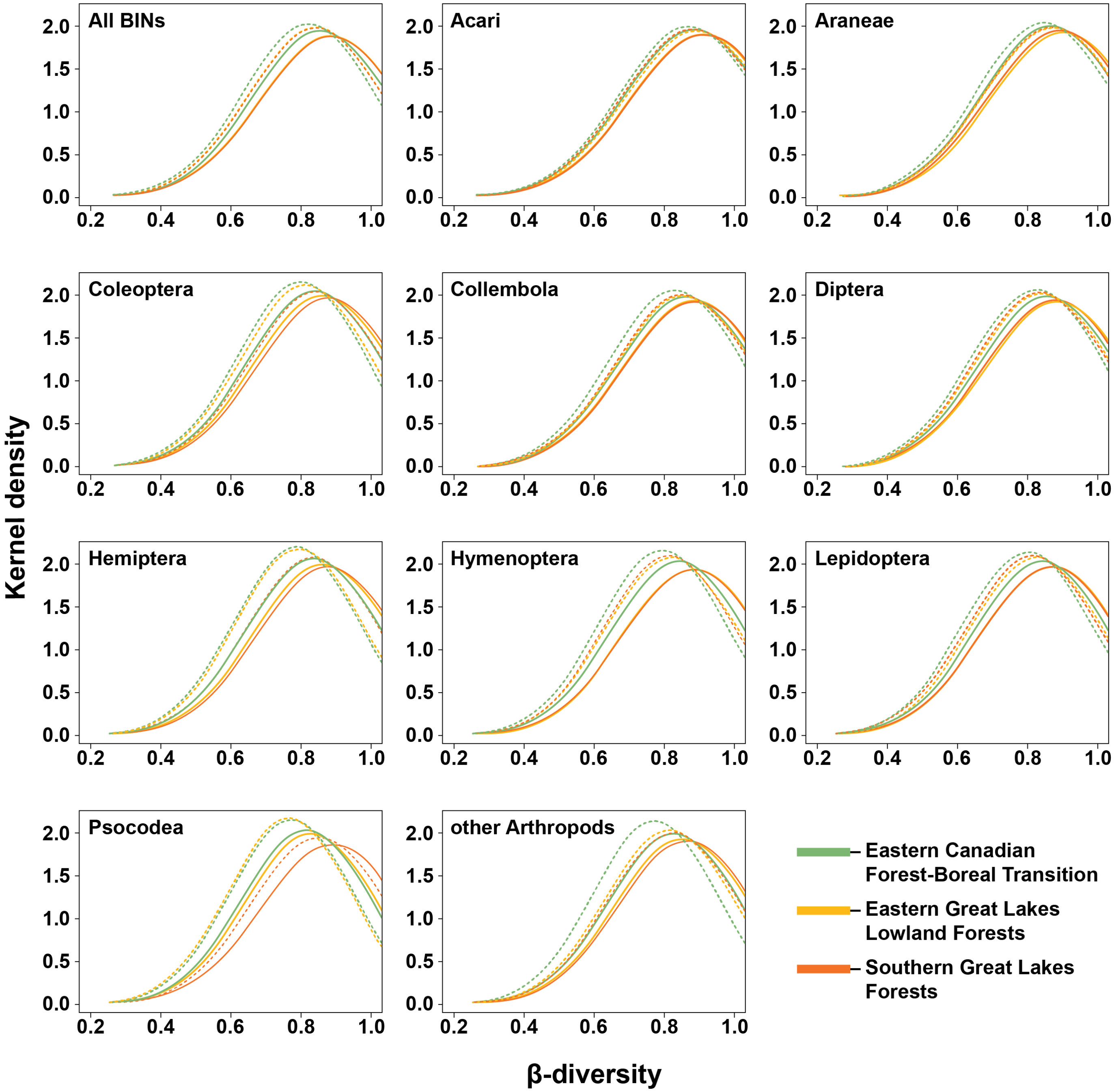

All BINs
Acari
Araneae
Coleoptera
Collembola
Diptera
Hemiptera
Hymenoptera
Lepidoptera
Psocodea
other Arthropods
Kernel density
β-diversity
0.0
0.5
1.0
1.5
2.0
0.2
0.4
0.6
0.8
1.0
Eastern Canadian Forest-Boreal Transition
Eastern Great Lakes Lowland Forests
Southern Great Lakes Forests

Figure S1

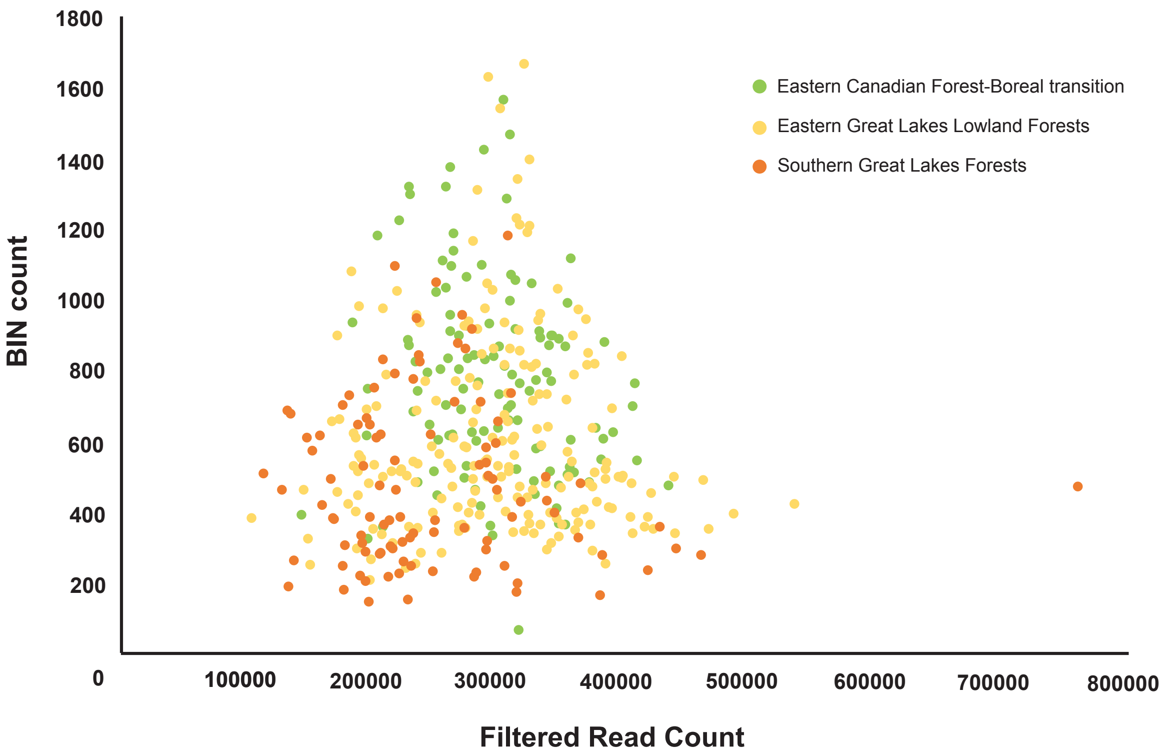

BIN count
1800
1600
1400
1200
1000
800
600
400
200
0
100000
200000
300000
400000
500000
600000
700000
800000
Filtered Read Count
Eastern Canadian Forest-Boreal transition
Eastern Great Lakes Lowland Forests
Southern Great Lakes Forests

Table S1: mBRAVE project codes as well as samples analyzed and read coverage for each 530 chip analyzed on the Ion Torrent S5. The 11 chips that included samples from two sites are highlighted in yellow.

**Project Code**	**Project Title**	**Reads**	**Filtered Reads**
MBR-OPPMAA	Ontario Provincial Parks 2014:CCDB-S5-0053_CBGMB-00003_Algonquin PP - Rock Lake Site 1	9376304	2592428
MBR-OPPMABMAC	Ontario Provincial Parks 2014:CCDB-S5-0066_CBGMB-00016_Algonquin PP - Oxtongue Site 3 + Awenda PP	8544988	2674401
MBR-OPPMADMAE	Ontario Provincial Parks 2014:CCDB-S5-0067_CBGMB-00017_Balsam Lake PP + Bayview Escarpment PP	8535733	3294349
MBR-OPPMAFMAI	Ontario Provincial Parks 2014:CCDB-S5-0068_CBGMB-00018_Bell Bay PP + Boyne Valley PP	6430734	1847104
MBR-OPPMAHMBD	Ontario Provincial Parks 2014:CCDB-S5-0086_CBGMB-00035_Bon Echo PP - Site 1 + Morris Tract PP	7271497	2424720
MBR-OPPMAJ	Ontario Provincial Parks 2014:CCDB-S5-0057_CBGMB-00007_Bronte Creek PP	6892296	2517868
MBR-OPPMAKMAO	Ontario Provincial Parks 2014:CCDB-S5-0073_CBGMB-00019_Charleston Lake PP + Ferris PP	10020289	3480857
MBR-OPPMAL	Ontario Provincial Parks 2014:CCDB-S5-0058_CBGMB-00008_Duncan Escarpment PP	9293842	3435885
MBR-OPPMAN	Ontario Provincial Parks 2014:CCDB-S5-0061_CBGMB-00009_Emily PP	8315694	3169017
MBR-OPPMAP	Ontario Provincial Parks 2014:CCDB-S5-0063_CBGMB-00010_Forks of the Credit PP	10348276	4090743
MBR-OPPMAQMAU	Ontario Provincial Parks 2014:CCDB-S5-0069_CBGMB-00020_Frontenac PP + Inverhuron PP	7931246	2871585
MBR-OPPMAR	Ontario Provincial Parks 2014:CCDB-S5-0064_CBGMB-00012_Holland Landing Prairie PP	8124964	2701838
MBR-OPPMAS	Ontario Provincial Parks 2014:CCDB-S5-0054_CBGMB-00004_Hope Bay Forest PP	9227593	3463105
MBR-OPPMAT	Ontario Provincial Parks 2014:CCDB-S5-0065_CBGMB-00013_Indian Point PP - Site 1	9151934	3599629
MBR-OPPMAWMAZ	Ontario Provincial Parks 2014:CCDB-S5-0070_CBGMB-00021_John E Pearce PP + Lions Head PP	10693129	3641703
MBR-OPPMAX	Ontario Provincial Parks 2014:CCDB-S5-0080_CBGMB-00026_Johnston Harbour PP	7927583	2339035
MBR-OPPMAY	Ontario Provincial Parks 2014:CCDB-S5-0081_CBGMB-00027_Lake St Peter PP	9380178	3266285
MBR-OPPMBAMBE	Ontario Provincial Parks 2014:CCDB-S5-0072_CBGMB-00022_Lower Madawaska River PP + Murphys Point PP	10315593	3815438
MBR-OPPMBB	Ontario Provincial Parks 2014:CCDB-S5-0082_CBGMB-00028_MacGregor Point PP	9944565	3854418
MBR-OPPMBC	Ontario Provincial Parks 2014:CCDB-S5-0056_CBGMB-00006_Mark S Burnham PP	10796469	4536699
MBR-OPPMBFMBH	Ontario Provincial Parks 2014:CCDB-S5-0074_CBGMB-00023_Ojibway Prairie PP + Pinery PP - Site 2	9804179	3243861
MBR-OPPMBG	Ontario Provincial Parks 2014:CCDB-S5-0083_CBGMB-00029_Petroglyphs PP	10645785	3989178

MBR-OPPMBI	Ontario Provincial Parks 2014:CCDB-S5-0084_CBGMB-00030_Port Burwell PP	10140496	3058298
MBR-OPPMBJ	Ontario Provincial Parks 2014:CCDB-S5-0085_CBGMB-00031_Presqu'ile PP	10651981	4259741
MBR-OPPMBMMBN	Ontario Provincial Parks 2014:CCDB-S5-0075_CBGMB-00024_Rondeau PP - Site 1 + Sandbanks PP	10725061	3868406
MBR-OPPMBO	Ontario Provincial Parks 2014:CCDB-S5-0087_CBGMB-00036_Selkirk PP	9153371	2649363
MBR-OPPMBP	Ontario Provincial Parks 2014:CCDB-S5-0055_CBGMB-00005_Sharbot Lake PP	8344274	3368635
MBR-OPPMBQMBU	Ontario Provincial Parks 2014:CCDB-S5-0079_CBGMB-00025_Short Hills PP + Turkey Point PP	7941400	2702280
MBR-OPPMBR	Ontario Provincial Parks 2014:CCDB-S5-0088_CBGMB-00037_Sibbald Point PP	8752087	3151909
MBR-OPPMBS	Ontario Provincial Parks 2014:CCDB-S5-0089_CBGMB-00038_Silent Lake PP	8381273	2870078
MBR-OPPMBT	Ontario Provincial Parks 2014:CCDB-S5-0090_CBGMB-00039_Silver Lake PP	8105235	2674401
MBR-OPPMBX	Ontario Provincial Parks 2014:CCDB-S5-0097_CBGMB-00040_Wheatley PP	9036296	2592428
MBR-OPPMBZZ	Ontario Provincial Parks 2014:CCDB-S5-0052_CBGMB-00002_Peter's Woods PP Malaise	8048432	3302341
MBR-OPPMBY	Ontario Provincial Parks 2014:CCDB-S5-041_CBGMB-00001_Long Point PP Malaise	11177387	4232828
MBR-OPPMAG	Ontario Provincial Parks 2014:CCDB-S5-0127_CBGMB-00068_Black Creek PP	7877916	2491752
MBR-OPPMAM	Ontario Provincial Parks 2014:CCDB-S5-0128_CBGMB-00069_Earl Rowe PP	8955778	3043981
MBR-OPPMAV	Ontario Provincial Parks 2014:CCDB-S5-0129_CBGMB-00070_James N Allan PP	6594231	1605985
MBR-OPPMBK	Ontario Provincial Parks 2014:CCDB-S5-0130_CBGMB-00071_Pretty River Valley PP	7497170	2158049
MBR-OPPMBL	Ontario Provincial Parks 2014:CCDB-S5-0131_CBGMB-00072_Rock Point PP	8479191	1624386
MBR-OPPMBV	Ontario Provincial Parks 2014:CCDB-S5-0132_CBGMB-00073_Upper Madawaska River PP	9938090	3074949
MBR-OPPMBW	Ontario Provincial Parks 2014:CCDB-S5-0133_CBGMB-00074_Wasaga Beach PP	9050667	2673304

Table S2: GPS coordinates, elevation (m), and ecoregion assignment for the 52 sampling sites and the number of BINs recovered from each site. ECF = Eastern Canadian Forest (15 sites); EGL = Eastern Great Lakes Forests (24 sites); SGL = Southern Great Lakes Forests (13 sites).

Provincial Park	Latitude	Longitude	Elevation(m)	BINs	Ecoregion
Algonquin - Oxtongue River	45.4621	-78.796	427	1642	ECF
Algonquin - Rock Lake	45.51962	-78.39752	353	2030	ECF
Bell Bay	45.51506	-77.81812	320	3186	ECF
Bon Echo	44.89405	-77.19691	272	2638	ECF
Charleston Lake	44.49798	-76.0414	88	3021	ECF
Frontenac	44.51783	-76.53944	166	3498	ECF
Indian Point	44.60414	-78.82895	258	2403	ECF
Lake St Peter	45.3202	-78.02496	406	2664	ECF
Lower Madawaska River	45.25606	-77.19221	262	3333	ECF
Murphys Point	44.78118	-76.2336	132	4581	ECF
Petroglyphs	44.61605	-78.04084	265	3194	ECF
Sharbot Lake	44.77952	-76.72379	201	2878	ECF
Silent Lake	44.92144	-78.06931	360	2895	ECF
Silver Lake	44.83129	-76.57565	184	4508	ECF
Upper Madawaska River	45.53535	-78.04699	318	3796	ECF
Awenda	44.82534	-79.98458	224	3135	EGL
Balsam Lake	44.62857	-78.8614	274	3227	EGL
Bayview Escarpment	44.63367	-80.69829	329	3966	EGL
Black Creek	44.96797	-81.36156	179	2369	EGL
Boyne Valley	44.11563	-80.12777	460	1609	EGL
Duncan Escarpment	44.42305	-80.46923	395	1957	EGL
Earl Rowe	44.15176	-79.903	216	1479	EGL
Emily	44.34143	-78.53746	251	1916	EGL
Ferris	44.28286	-77.79627	132	4005	EGL
Forks of the Credit	43.82415	-80.00309	403	2753	EGL
Holland Landing Prairie	44.11894	-79.48795	225	2545	EGL
Hope Bay Forest	44.92509	-81.15563	253	1071	EGL
Inverhuron	44.29838	-81.59065	182	1785	EGL
Johnston Harbour-Pine Tree Point	45.1171	-81.53679	177	1939	EGL
Lions Head	44.99539	-81.2334	219	2074	EGL
MacGregor Point	44.41072	-81.44641	191	2607	EGL
Mark S Burnham	44.29882	-78.26779	209	1633	EGL
Morris Tract	43.72995	-81.64166	257	1673	EGL
Peters Woods	44.12845	-78.04057	236	4197	EGL
Presqu'ile	44.00914	-77.7424	77	3197	EGL
Pretty River Valley	44.41232	-80.30035	337	3550	EGL

Sandbanks	43.90287	-77.26929	85	1312	EGL
Sibbald Point	44.32982	-79.32737	221	1872	EGL
Wasaga Beach	44.51258	-80.01165	188	1314	EGL
Bronte Creek	43.4023	-79.7617	125	2800	SGL
James N Allan	42.84962	-79.66397	175	2029	SGL
John E Pearce	42.60595	-81.44243	195	1339	SGL
Long Point	42.58006	-80.38538	175	1363	SCF
Ojibway Prairie	42.26278	-83.07246	180	3311	SGL
Pinery	43.26987	-81.82706	182	1978	SGL
Port Burwell	42.6544	-80.81493	196	996	SGL
Rock Point	42.85405	-79.55536	176	2246	SGL
Rondeau	42.30206	-81.85306	175	1356	SGL
Selkirk	42.81676	-79.95736	180	1146	SGL
Short Hills	43.11288	-79.27376	95	2541	SGL
Turkey Point	42.70515	-80.32849	222	2513	SGL
Wheatley	42.09199	-82.44227	181	1210	SGL

Table S3: Number of reads, BINs, and OTUs for each of the 410 samples from the 52 sites that were metabarcoded.

Provincial Park	Start Date	End Date	Project Name	Run Name	Reads	BINs	Filtered reads	OTU Count
Algonquin - Oxtongue	21-May-14	03-Jun-14	MBR-OPPMABMAC	GMP#04618_CCDB-S5-0066	844737	518	315413	28
Algonquin - Oxtongue	17-Jun-14	01-Jul-14	MBR-OPPMABMAC	GMP#04620_CCDB-S5-0066	874866	525	275802	20
Algonquin - Oxtongue	15-Jul-14	29-Jul-14	MBR-OPPMABMAC	GMP#04622_CCDB-S5-0066	789560	480	236636	31
Algonquin - Oxtongue	12-Aug-14	26-Aug-14	MBR-OPPMABMAC	GMP#04624_CCDB-S5-0066	809049	359	354373	40
Algonquin - Oxtongue	09-Sep-14	23-Sep-14	MBR-OPPMABMAC	GMP#04626_CCDB-S5-0066	718303	329	296773	20
Algonquin - Rock Lake	06-May-14	21-May-14	MBR-OPPMAA	GMP#04597_CCDB-S5-0053	1003100	471	435638	37
Algonquin - Rock Lake	21-May-14	03-Jun-14	MBR-OPPMAA	GMP#04598_CCDB-S5-0053	947819	543	383007	36
Algonquin - Rock Lake	03-Jun-14	17-Jun-14	MBR-OPPMAA	GMP#04599_CCDB-S5-0053	826499	522	357669	28
Algonquin - Rock Lake	17-Jun-14	01-Jul-14	MBR-OPPMAA	GMP#04600_CCDB-S5-0053	887071	698	310705	26
Algonquin - Rock Lake	15-Jul-14	29-Jul-14	MBR-OPPMAA	GMP#04602_CCDB-S5-0053	844585	482	329125	49
Algonquin - Rock Lake	29-Jul-14	12-Aug-14	MBR-OPPMAA	GMP#04603_CCDB-S5-0053	774474	446	329818	31
Algonquin - Rock Lake	12-Aug-14	26-Aug-14	MBR-OPPMAA	GMP#04604_CCDB-S5-0053	885213	506	360810	50
Algonquin - Rock Lake	26-Aug-14	09-Sep-14	MBR-OPPMAA	GMP#04605_CCDB-S5-0053	875623	362	349362	52
Algonquin - Rock Lake	09-Sep-14	23-Sep-14	MBR-OPPMAA	GMP#04606_CCDB-S5-0053	565808	320	197081	34
Algonquin - Rock Lake	1-Jul-2014	15-Jul-2014	MBR-OPPMAA	GMP#04611_CCDB-S5-0053	911899	791	339632	39
Bell Bay	22-May-14	04-Jun-14	MBR-OPPMAFMAI	GMP#04658_CCDB-S5-0068	638807	743	197057	9
Bell Bay	18-Jun-2014	2-Jul-2014	MBR-OPPMAFMAI	GMP#04660_CCDB-S5-0068	680480	1178	205457	9
Bell Bay	16-Jul-14	30-Jul-14	MBR-OPPMAFMAI	GMP#04662_CCDB-S5-0068	655110	932	185354	8
Bell Bay	13-Aug-14	27-Aug-14	MBR-OPPMAFMAI	GMP#04664_CCDB-S5-0068	646714	611	196766	9
Bell Bay	10-Sep-14	24-Sep-14	MBR-OPPMAFMAI	GMP#04666_CCDB-S5-0068	498632	387	144725	12
Bon Echo	22-May-14	04-Jun-14	MBR-OPPMAHMBD	GMP#03668_CCDB-S5-0086	751958	598	283465	31
Bon Echo	18-Jun-14	02-Jul-14	MBR-OPPMAHMBD	GMP#03670_CCDB-S5-0086	730555	799	254799	26
Bon Echo	16-Jul-14	30-Jul-14	MBR-OPPMAHMBD	GMP#03672_CCDB-S5-0086	676557	791	244764	26
Bon Echo	13-Aug-14	27-Aug-14	MBR-OPPMAHMBD	GMP#03674_CCDB-S5-0086	712717	643	246017	24
Bon Echo	10-Sep-14	24-Sep-14	MBR-OPPMAHMBD	GMP#04582_CCDB-S5-0086	620386	353	209139	32
Charleston Lake	23-May-14	05-Jun-14	MBR-OPPMAKMAO	GMP#03708_CCDB-S5-0073	1016970	890	333977	35
Charleston Lake	19-Jun-14	03-Jul-14	MBR-OPPMAKMAO	GMP#03710_CCDB-S5-0073	990071	994	310127	38
Charleston Lake	17-Jul-14	31-Jul-14	MBR-OPPMAKMAO	GMP#03712_CCDB-S5-0073	895735	1053	314851	38
Charleston Lake	14-Aug-14	28-Aug-14	MBR-OPPMAKMAO	GMP#03714_CCDB-S5-0073	921329	867	341614	30
Charleston Lake	11-Sep-14	25-Sep-14	MBR-OPPMAKMAO	GMP#04587_CCDB-S5-0073	916810	604	384385	30

Frontenac	23-May-14	05-Jun-14	MBR-OPPMAQMAU	GMP#03716_CCDB-S5-0069	807008	907	263178	28
Frontenac	19-Jun-14	03-Jul-14	MBR-OPPMAQMAU	GMP#03718_CCDB-S5-0069	772854	1093	263942	15
Frontenac	17-Jul-14	31-Jul-14	MBR-OPPMAQMAU	GMP#03720_CCDB-S5-0069	726646	1019	251745	9
Frontenac	14-Aug-14	28-Aug-14	MBR-OPPMAQMAU	GMP#03722_CCDB-S5-0069	766997	745	273278	21
Frontenac	11-Sep-14	25-Sep-14	MBR-OPPMAQMAU	GMP#04588_CCDB-S5-0069	795459	510	341835	30
Indian Point	30-Apr-14	19-May-14	MBR-OPPMAT	GMP#03611_CCDB-S5-0065	912045	502	356130	36
Indian Point	19-May-14	02-Jun-14	MBR-OPPMAT	GMP#03612_CCDB-S5-0065	874160	896	343593	15
Indian Point	02-Jun-14	16-Jun-14	MBR-OPPMAT	GMP#03613_CCDB-S5-0065	849050	656	316507	25
Indian Point	16-Jun-14	30-Jun-14	MBR-OPPMAT	GMP#03614_CCDB-S5-0065	850842	761	317868	33
Indian Point	30-Jun-14	14-Jul-14	MBR-OPPMAT	GMP#03615_CCDB-S5-0065	823052	838	297333	23
Indian Point	14-Jul-14	28-Jul-14	MBR-OPPMAT	GMP#03616_CCDB-S5-0065	799424	633	300409	20
Indian Point	28-Jul-14	11-Aug-14	MBR-OPPMAT	GMP#03617_CCDB-S5-0065	747554	621	280113	20
Indian Point	11-Aug-14	25-Aug-14	MBR-OPPMAT	GMP#03618_CCDB-S5-0065	872463	696	407894	25
Indian Point	25-Aug-14	08-Sep-14	MBR-OPPMAT	GMP#04523_CCDB-S5-0065	831488	481	373536	25
Indian Point	08-Sep-14	22-Sep-14	MBR-OPPMAT	GMP#04575_CCDB-S5-0065	724719	355	294954	20
Lake St Peter	06-May-14	21-May-14	MBR-OPPMAY	GMP#03659_CCDB-S5-0081	953516	470	348779	31
Lake St Peter	21-May-14	03-Jun-14	MBR-OPPMAY	GMP#03660_CCDB-S5-0081	904431	738	326084	19
Lake St Peter	03-Jun-14	17-Jun-14	MBR-OPPMAY	GMP#03661_CCDB-S5-0081	785604	493	273687	34
Lake St Peter	17-Jun-14	01-Jul-14	MBR-OPPMAY	GMP#03662_CCDB-S5-0081	860141	785	311817	36
Lake St Peter	01-Jul-14	15-Jul-14	MBR-OPPMAY	GMP#03663_CCDB-S5-0081	733692	739	237245	41
Lake St Peter	15-Jul-14	29-Jul-14	MBR-OPPMAY	GMP#03664_CCDB-S5-0081	834801	697	259273	32
Lake St Peter	29-Jul-14	12-Aug-14	MBR-OPPMAY	GMP#03665_CCDB-S5-0081	768612	799	270019	33
Lake St Peter	12-Aug-14	26-Aug-14	MBR-OPPMAY	GMP#03666_CCDB-S5-0081	773713	685	271422	22
Lake St Peter	26-Aug-14	09-Sep-14	MBR-OPPMAY	GMP#04529_CCDB-S5-0081	760724	615	264304	32
Lake St Peter	09-Sep-14	23-Sep-14	MBR-OPPMAY	GMP#04581_CCDB-S5-0081	775526	411	286958	27
Lower Madawaska River	22-May-14	04-Jun-14	MBR-OPPMBAMBE	GMP#04648_CCDB-S5-0072	983613	865	353835	41
Lower Madawaska River	18-Jun-2014	2-Jul-2014	MBR-OPPMBAMBE	GMP#04650_CCDB-S5-0072	944474	1042	327415	46
Lower Madawaska River	16-Jul-14	30-Jul-14	MBR-OPPMBAMBE	GMP#04652_CCDB-S5-0072	945176	886	349294	38
Lower Madawaska River	13-Aug-14	27-Aug-14	MBR-OPPMBAMBE	GMP#04654_CCDB-S5-0072	1014340	761	409702	48
Lower Madawaska River	10-Sep-14	24-Sep-14	MBR-OPPMBAMBE	GMP#04656_CCDB-S5-0072	839242	407	347173	30
Murphys Point	23-May-14	05-Jun-14	MBR-OPPMBAMBE	GMP#03700_CCDB-S5-0072	918087	1283	307945	34
Murphys Point	19-Jun-14	03-Jul-14	MBR-OPPMBAMBE	GMP#03702_CCDB-S5-0072	835990	1563	305411	20
Murphys Point	17-Jul-14	31-Jul-14	MBR-OPPMBAMBE	GMP#03704_CCDB-S5-0072	918549	1466	310558	38

Murphys Point	14-Aug-14	28-Aug-14	MBR-OPPMBAMBE	GMP#03706_CCDB-S5-0072	928385	1113	358668	46
Murphys Point	11-Sep-14	25-Sep-14	MBR-OPPMBAMBE	GMP#04586_CCDB-S5-0072	939127	632	378176	47
Petroglyphs	30-Apr-14	19-May-14	MBR-OPPMBG	GMP#03635_CCDB-S5-0083	1160480	620	391954	35
Petroglyphs	19-May-14	02-Jun-14	MBR-OPPMBG	GMP#03636_CCDB-S5-0083	1065690	878	384768	24
Petroglyphs	02-Jun-14	16-Jun-14	MBR-OPPMBG	GMP#03637_CCDB-S5-0083	916303	906	333876	21
Petroglyphs	16-Jun-14	30-Jun-14	MBR-OPPMBG	GMP#03638_CCDB-S5-0083	938924	770	331195	31
Petroglyphs	30-Jun-14	14-Jul-14	MBR-OPPMBG	GMP#03639_CCDB-S5-0083	833565	1066	311163	36
Petroglyphs	14-Jul-14	28-Jul-14	MBR-OPPMBG	GMP#03640_CCDB-S5-0083	912435	988	355537	33
Petroglyphs	28-Jul-14	11-Aug-14	MBR-OPPMBG	GMP#03641_CCDB-S5-0083	824265	915	314533	29
Petroglyphs	11-Aug-14	25-Aug-14	MBR-OPPMBG	GMP#03642_CCDB-S5-0083	914876	765	342733	34
Petroglyphs	25-Aug-14	08-Sep-14	MBR-OPPMBG	GMP#04526_CCDB-S5-0083	830065	599	358410	32
Petroglyphs	08-Sep-14	22-Sep-14	MBR-OPPMBG	GMP#04578_CCDB-S5-0083	918003	542	410741	58
Sharbot Lake	08-May-14	22-May-14	MBR-OPPMBP	GMP#03683_CCDB-S5-0055	862000	656	309719	31
Sharbot Lake	22-May-14	04-Jun-14	MBR-OPPMBP	GMP#03684_CCDB-S5-0055	782739	841	281804	20
Sharbot Lake	04-Jun-14	18-Jun-14	MBR-OPPMBP	GMP#03685_CCDB-S5-0055	776436	809	305587	12
Sharbot Lake	18-Jun-14	02-Jul-14	MBR-OPPMBP	GMP#03686_CCDB-S5-0055	833241	894	343236	25
Sharbot Lake	02-Jul-14	16-Jul-14	MBR-OPPMBP	GMP#03687_CCDB-S5-0055	732624	930	293650	28
Sharbot Lake	16-Jul-14	30-Jul-14	MBR-OPPMBP	GMP#03688_CCDB-S5-0055	731893	830	276668	35
Sharbot Lake	30-Jul-14	13-Aug-14	MBR-OPPMBP	GMP#03689_CCDB-S5-0055	712920	730	301577	31
Sharbot Lake	13-Aug-14	27-Aug-14	MBR-OPPMBP	GMP#03690_CCDB-S5-0055	801316	581	334252	38
Sharbot Lake	27-Aug-14	10-Sep-14	MBR-OPPMBP	GMP#04532_CCDB-S5-0055	793053	688	308642	30
Sharbot Lake	10-Sep-14	24-Sep-14	MBR-OPPMBP	GMP#04584_CCDB-S5-0055	691937	457	282548	30
Silent Lake	29-Apr-14	21-May-14	MBR-OPPMBS	GMP#03651_CCDB-S5-0089	802981	511	249918	26
Silent Lake	21-May-14	02-Jun-14	MBR-OPPMBS	GMP#03652_CCDB-S5-0089	888062	625	289299	33
Silent Lake	02-Jun-14	16-Jun-14	MBR-OPPMBS	GMP#03653_CCDB-S5-0089	830306	60	317280	34
Silent Lake	16-Jun-14	30-Jun-14	MBR-OPPMBS	GMP#03654_CCDB-S5-0089	829714	764	285474	29
Silent Lake	30-Jun-14	14-Jul-14	MBR-OPPMBS	GMP#03655_CCDB-S5-0089	781166	864	301858	29
Silent Lake	14-Jul-14	28-Jul-14	MBR-OPPMBS	GMP#03656_CCDB-S5-0089	777000	922	275946	25
Silent Lake	28-Jul-14	11-Aug-14	MBR-OPPMBS	GMP#03657_CCDB-S5-0089	717115	883	228910	49
Silent Lake	11-Aug-14	25-Aug-14	MBR-OPPMBS	GMP#03658_CCDB-S5-0089	798452	821	235324	66
Silent Lake	25-Aug-14	08-Sep-14	MBR-OPPMBS	GMP#04528_CCDB-S5-0089	663059	680	233401	39
Silent Lake	08-Sep-14	23-Sep-14	MBR-OPPMBS	GMP#04580_CCDB-S5-0089	677856	442	252144	24
Silver Lake	08-May-14	22-May-14	MBR-OPPMBT	GMP#03691_CCDB-S5-0090	854380	953	263002	19

Silver Lake	22-May-14	05-Jun-14	MBR-OPPMBT	GMP#03692_CCDB-S5-0090	850977	1316	259150	21
Silver Lake	05-Jun-14	19-Jun-14	MBR-OPPMBT	GMP#03693_CCDB-S5-0090	818324	1371	262921	22
Silver Lake	19-Jun-14	03-Jul-14	MBR-OPPMBT	GMP#03694_CCDB-S5-0090	698069	1316	229698	18
Silver Lake	03-Jul-14	17-Jul-14	MBR-OPPMBT	GMP#03695_CCDB-S5-0090	606866	1222	222044	22
Silver Lake	17-Jul-14	31-Jul-14	MBR-OPPMBT	GMP#03696_CCDB-S5-0090	718354	1295	231254	17
Silver Lake	31-Jul-14	14-Aug-14	MBR-OPPMBT	GMP#03697_CCDB-S5-0090	777805	1183	265690	23
Silver Lake	14-Aug-14	28-Aug-14	MBR-OPPMBT	GMP#03698_CCDB-S5-0090	777946	830	261094	31
Silver Lake	28-Aug-14	11-Sep-14	MBR-OPPMBT	GMP#04533_CCDB-S5-0090	673941	868	230045	16
Silver Lake	11-Sep-14	25-Sep-14	MBR-OPPMBT	GMP#04585_CCDB-S5-0090	708537	600	253210	19
Uer Madawaska River	07-May-14	22-May-14	MBR-OPPMBV	GMP#04637_CCDB-S5-0073	1091440	575	330625	43
Uer Madawaska River	22-May-14	04-Jun-14	MBR-OPPMBV	GMP#04638_CCDB-S5-0073	929115	828	289972	30
Uer Madawaska River	04-Jun-14	18-Jun-14	MBR-OPPMBV	GMP#04639_CCDB-S5-0073	935574	1062	276120	20
Uer Madawaska River	18-Jun-14	02-Jul-14	MBR-OPPMBV	GMP#04640_CCDB-S5-0073	948650	1420	289599	23
Uer Madawaska River	02-Jul-14	16-Jul-14	MBR-OPPMBV	GMP#04641_CCDB-S5-0073	913071	1095	287466	35
Uer Madawaska River	16-Jul-14	30-Jul-14	MBR-OPPMBV	GMP#04642_CCDB-S5-0073	880011	1136	264949	20
Uer Madawaska River	30-Jul-14	13-Aug-14	MBR-OPPMBV	GMP#04643_CCDB-S5-0073	857327	1106	256814	27
Uer Madawaska River	13-Aug-14	27-Aug-14	MBR-OPPMBV	GMP#04644_CCDB-S5-0073	815565	1032	259401	27
Uer Madawaska River	27-Aug-14	10-Sep-14	MBR-OPPMBV	GMP#04645_CCDB-S5-0073	828975	894	269341	34
Uer Madawaska River	10-Sep-14	24-Sep-14	MBR-OPPMBV	GMP#04646_CCDB-S5-0073	781088	612	261623	26
Awenda	16-May-14	30-May-14	MBR-OPPMABMAC	GMP#03444_CCDB-S5-0066	818191	526	300493	26
Awenda	13-Jun-14	27-Jun-14	MBR-OPPMABMAC	GMP#03446_CCDB-S5-0066	718570	859	297534	33
Awenda	11-Jul-14	25-Jul-14	MBR-OPPMABMAC	GMP#03448_CCDB-S5-0066	736111	932	306259	28
Awenda	08-Aug-14	22-Aug-14	MBR-OPPMABMAC	GMP#03450_CCDB-S5-0066	724520	842	287645	14
Awenda	05-Sep-14	19-Sep-14	MBR-OPPMABMAC	GMP#04570_CCDB-S5-0066	630232	531	236621	16
Balsam Lake	19-May-14	02-Jun-14	MBR-OPPMADMAE	GMP#03628_CCDB-S5-0067	911348	730	339448	33
Balsam Lake	16-Jun-14	30-Jun-14	MBR-OPPMADMAE	GMP#03630_CCDB-S5-0067	798118	973	290456	20
Balsam Lake	14-Jul-14	28-Jul-14	MBR-OPPMADMAE	GMP#03632_CCDB-S5-0067	775026	934	277677	20
Balsam Lake	11-Aug-14	25-Aug-14	MBR-OPPMADMAE	GMP#03634_CCDB-S5-0067	851003	857	310549	51
Balsam Lake	08-Sep-14	22-Sep-14	MBR-OPPMADMAE	GMP#04577_CCDB-S5-0067	857458	518	385900	40
Bayview Escarpment	15-May-14	29-May-14	MBR-OPPMADMAE	GMP#03412_CCDB-S5-0067	755669	600	311612	20
Bayview Escarpment	12-Jun-14	26-Jun-14	MBR-OPPMADMAE	GMP#03414_CCDB-S5-0067	687590	1164	281112	23
Bayview Escarpment	10-Jul-14	24-Jul-14	MBR-OPPMADMAE	GMP#03416_CCDB-S5-0067	721775	1307	284362	28
Bayview Escarpment	07-Aug-14	21-Aug-14	MBR-OPPMADMAE	GMP#03418_CCDB-S5-0067	728550	1044	292256	18

Bayview Escarpment	04-Sep-14	18-Sep-14	MBR-OPPMADMAE	GMP#04566_CCDB-S5-0067	679848	710	251276	28
Black Creek	01-May-14	15-May-14	MBR-OPPMAG	GMP#03379_CCDB-S5-0068	742597	261	199645	37
Black Creek	15-May-14	29-May-14	MBR-OPPMAG	GMP#03380_CCDB-S5-0068	722317	306	216954	17
Black Creek	29-May-14	12-Jun-14	MBR-OPPMAG	GMP#03381_CCDB-S5-0068	690384	617	186452	22
Black Creek	12-Jun-14	26-Jun-14	MBR-OPPMAG	GMP#03382_CCDB-S5-0068	756436	694	203826	31
Black Creek	26-Jun-14	10-Jul-14	MBR-OPPMAG	GMP#03383_CCDB-S5-0068	727852	683	236496	35
Black Creek	10-Jul-14	24-Jul-14	MBR-OPPMAG	GMP#03384_CCDB-S5-0068	639616	783	212061	34
Black Creek	24-Jul-14	07-Aug-14	MBR-OPPMAG	GMP#03385_CCDB-S5-0068	648999	687	196751	37
Black Creek	07-Aug-14	21-Aug-14	MBR-OPPMAG	GMP#03386_CCDB-S5-0068	697244	539	233723	38
Black Creek	21-Aug-14	04-Sep-14	MBR-OPPMAG	GMP#04510_CCDB-S5-0068	718558	435	300781	45
Black Creek	04-Sep-14	18-Sep-14	MBR-OPPMAG	GMP#04562_CCDB-S5-0068	701255	353	229871	30
Boyne Valley	16-May-14	30-May-14	MBR-OPPMAFMAI	GMP#03468_CCDB-S5-0068	583784	419	181783	3
Boyne Valley	13-Jun-14	27-Jun-14	MBR-OPPMAFMAI	GMP#03470_CCDB-S5-0068	455387	377	105497	5
Boyne Valley	11-Jul-14	25-Jul-14	MBR-OPPMAFMAI	GMP#03472_CCDB-S5-0068	546965	459	146622	3
Boyne Valley	08-Aug-14	22-Aug-14	MBR-OPPMAFMAI	GMP#03474_CCDB-S5-0068	546421	318	150288	5
Boyne Valley	05-Sep-14	19-Sep-14	MBR-OPPMAFMAI	GMP#04573_CCDB-S5-0068	526329	245	151831	11
Duncan Escarpment	28-Apr-14	15-May-14	MBR-OPPMAL	GMP#03419_CCDB-S5-0058	963348	496	356781	37
Duncan Escarpment	15-May-14	29-May-14	MBR-OPPMAL	GMP#03420_CCDB-S5-0058	878953	468	317573	31
Duncan Escarpment	29-May-14	12-Jun-14	MBR-OPPMAL	GMP#03421_CCDB-S5-0058	754664	455	282724	24
Duncan Escarpment	12-Jun-14	26-Jun-14	MBR-OPPMAL	GMP#03422_CCDB-S5-0058	704202	481	235004	31
Duncan Escarpment	26-Jun-14	10-Jul-14	MBR-OPPMAL	GMP#03423_CCDB-S5-0058	801010	686	283845	25
Duncan Escarpment	10-Jul-14	24-Jul-14	MBR-OPPMAL	GMP#03424_CCDB-S5-0058	909771	565	355905	52
Duncan Escarpment	24-Jul-14	07-Aug-14	MBR-OPPMAL	GMP#03425_CCDB-S5-0058	849900	495	303399	38
Duncan Escarpment	07-Aug-14	21-Aug-14	MBR-OPPMAL	GMP#03426_CCDB-S5-0058	849567	378	347503	28
Duncan Escarpment	21-Aug-14	04-Sep-14	MBR-OPPMAL	GMP#04515_CCDB-S5-0058	888901	404	368971	37
Duncan Escarpment	04-Sep-14	18-Sep-14	MBR-OPPMAL	GMP#04567_CCDB-S5-0058	784765	355	270055	52
Earl Rowe	28-Apr-14	16-May-14	MBR-OPPMAM	GMP#03459_CCDB-S5-0128	848279	349	237052	25
Earl Rowe	16-May-14	30-May-14	MBR-OPPMAM	GMP#03460_CCDB-S5-0128	852008	351	273970	27
Earl Rowe	30-May-14	13-Jun-14	MBR-OPPMAM	GMP#03461_CCDB-S5-0128	803327	358	272539	46
Earl Rowe	13-Jun-14	27-Jun-14	MBR-OPPMAM	GMP#03462_CCDB-S5-0128	827062	393	277298	38
Earl Rowe	27-Jun-14	11-Jul-14	MBR-OPPMAM	GMP#03463_CCDB-S5-0128	807062	521	263846	38
Earl Rowe	11-Jul-14	25-Jul-14	MBR-OPPMAM	GMP#03464_CCDB-S5-0128	865938	459	328648	48
Earl Rowe	25-Jul-14	08-Aug-14	MBR-OPPMAM	GMP#03465_CCDB-S5-0128	829864	387	324731	39

Earl Rowe	08-Aug-14	22-Aug-14	MBR-OPPMAM	GMP#03466_CCDB-S5-0128	807509	336	329222	32
Earl Rowe	22-Aug-14	05-Sep-14	MBR-OPPMAM	GMP#04520_CCDB-S5-0128	734386	393	249238	36
Earl Rowe	05-Sep-14	19-Sep-14	MBR-OPPMAM	GMP#04572_CCDB-S5-0128	730637	249	234816	26
Emily	05-May-14	23-May-14	MBR-OPPMAN	GMP#03747_CCDB-S5-0061	878902	477	338840	35
Emily	23-May-14	06-Jun-14	MBR-OPPMAN	GMP#03748_CCDB-S5-0061	840003	543	292504	23
Emily	06-Jun-14	20-Jun-14	MBR-OPPMAN	GMP#03749_CCDB-S5-0061	753001	496	291661	30
Emily	20-Jun-14	04-Jul-14	MBR-OPPMAN	GMP#03750_CCDB-S5-0061	729427	581	274036	34
Emily	04-Jul-14	18-Jul-14	MBR-OPPMAN	GMP#03751_CCDB-S5-0061	718291	561	254407	14
Emily	18-Jul-14	01-Aug-14	MBR-OPPMAN	GMP#03752_CCDB-S5-0061	778332	524	309110	29
Emily	01-Aug-14	15-Aug-14	MBR-OPPMAN	GMP#03753_CCDB-S5-0061	693375	533	261176	32
Emily	15-Aug-14	29-Aug-14	MBR-OPPMAN	GMP#03754_CCDB-S5-0061	627167	515	223957	27
Emily	29-Aug-14	12-Sep-14	MBR-OPPMAN	GMP#04540_CCDB-S5-0061	714422	527	282074	36
Emily	12-Sep-14	26-Sep-14	MBR-OPPMAN	GMP#04592_CCDB-S5-0061	779544	344	362086	22
Ferris	23-May-14	05-Jun-14	MBR-OPPMAKMAO	GMP#03740_CCDB-S5-0073	930942	1208	317855	24
Ferris	19-Jun-14	03-Jul-14	MBR-OPPMAKMAO	GMP#03742_CCDB-S5-0073	822709	1227	315539	11
Ferris	17-Jul-14	31-Jul-14	MBR-OPPMAKMAO	GMP#03744_CCDB-S5-0073	864538	1339	315939	12
Ferris	14-Aug-14	28-Aug-14	MBR-OPPMAKMAO	GMP#03746_CCDB-S5-0073	811779	1025	296579	11
Ferris	12-Sep-14	26-Sep-14	MBR-OPPMAKMAO	GMP#04591_CCDB-S5-0073	766414	547	192375	29
Forks of the Credit	28-Apr-14	16-May-14	MBR-OPPMAP	GMP#04015_CCDB-S5-0063	953951	393	342030	44
Forks of the Credit	16-May-14	30-May-14	MBR-OPPMAP	GMP#04016_CCDB-S5-0063	1047150	690	391623	45
Forks of the Credit	30-May-14	13-Jun-14	MBR-OPPMAP	GMP#04017_CCDB-S5-0063	950426	836	398874	32
Forks of the Credit	13-Jun-14	27-Jun-14	MBR-OPPMAP	GMP#04018_CCDB-S5-0063	943285	816	377441	33
Forks of the Credit	27-Jun-14	11-Jul-14	MBR-OPPMAP	GMP#04019_CCDB-S5-0063	888530	1026	347988	27
Forks of the Credit	11-Jul-14	25-Jul-14	MBR-OPPMAP	GMP#04020_CCDB-S5-0063	887030	896	359891	31
Forks of the Credit	25-Jul-14	08-Aug-14	MBR-OPPMAP	GMP#04021_CCDB-S5-0063	910778	938	332673	32
Forks of the Credit	08-Aug-14	22-Aug-14	MBR-OPPMAP	GMP#04022_CCDB-S5-0063	897965	811	371497	21
Forks of the Credit	22-Aug-14	05-Sep-14	MBR-OPPMAP	GMP#04544_CCDB-S5-0063	922643	847	372252	35
Forks of the Credit	05-Sep-14	19-Sep-14	MBR-OPPMAP	GMP#04596_CCDB-S5-0063	804347	537	359515	25
Holland Landing Prairie	01-May-14	19-May-14	MBR-OPPMAR	GMP#03603_CCDB-S5-0064	812146	558	313434	30
Holland Landing Prairie	19-May-14	02-Jun-14	MBR-OPPMAR	GMP#03604_CCDB-S5-0064	778900	650	281111	32
Holland Landing Prairie	02-Jun-14	16-Jun-14	MBR-OPPMAR	GMP#03605_CCDB-S5-0064	715926	577	275979	30
Holland Landing Prairie	16-Jun-14	30-Jun-14	MBR-OPPMAR	GMP#03606_CCDB-S5-0064	747657	774	278602	31
Holland Landing Prairie	30-Jun-14	14-Jul-14	MBR-OPPMAR	GMP#03607_CCDB-S5-0064	722911	924	274048	20

Holland Landing Prairie	14-Jul-14	28-Jul-14	MBR-OPPMAR	GMP#03608_CCDB-S5-0064	709151	766	266795	24
Holland Landing Prairie	28-Jul-14	11-Aug-14	MBR-OPPMAR	GMP#03609_CCDB-S5-0064	668655	581	248312	20
Holland Landing Prairie	11-Aug-14	25-Aug-14	MBR-OPPMAR	GMP#03610_CCDB-S5-0064	709589	607	296312	37
Holland Landing Prairie	25-Aug-14	08-Sep-14	MBR-OPPMAR	GMP#04522_CCDB-S5-0064	702446	606	265204	17
Holland Landing Prairie	08-Sep-14	22-Sep-14	MBR-OPPMAR	GMP#04574_CCDB-S5-0064	643055	351	275261	20
Hope Bay Forest	02-May-14	15-May-14	MBR-OPPMAS	GMP#03403_CCDB-S5-0054	976643	354	416907	40
Hope Bay Forest	15-May-14	29-May-14	MBR-OPPMAS	GMP#03404_CCDB-S5-0054	953456	347	424032	28
Hope Bay Forest	29-May-14	12-Jun-14	MBR-OPPMAS	GMP#03405_CCDB-S5-0054	912008	286	376098	19
Hope Bay Forest	12-Jun-14	26-Jun-14	MBR-OPPMAS	GMP#03406_CCDB-S5-0054	885956	250	386410	20
Hope Bay Forest	26-Jun-14	10-Jul-14	MBR-OPPMAS	GMP#03407_CCDB-S5-0054	882785	359	351437	32
Hope Bay Forest	10-Jul-14	24-Jul-14	MBR-OPPMAS	GMP#03408_CCDB-S5-0054	822028	366	364232	28
Hope Bay Forest	24-Jul-14	07-Aug-14	MBR-OPPMAS	GMP#03409_CCDB-S5-0054	690122	280	256323	23
Hope Bay Forest	07-Aug-14	21-Aug-14	MBR-OPPMAS	GMP#03410_CCDB-S5-0054	797965	340	269761	22
Hope Bay Forest	21-Aug-14	04-Sep-14	MBR-OPPMAS	GMP#04513_CCDB-S5-0054	676899	325	348743	12
Hope Bay Forest	04-Sep-14	18-Sep-14	MBR-OPPMAS	GMP#04565_CCDB-S5-0054	617635	237	227541	16
Inverhuron	14-May-14	28-May-14	MBR-OPPMAQMAU	GMP#03364_CCDB-S5-0069	727499	339	312861	28
Inverhuron	11-Jun-14	25-Jun-14	MBR-OPPMAQMAU	GMP#03366_CCDB-S5-0069	637841	498	228034	11
Inverhuron	09-Jul-14	23-Jul-14	MBR-OPPMAQMAU	GMP#03368_CCDB-S5-0069	708060	765	242726	14
Inverhuron	06-Aug-14	20-Aug-14	MBR-OPPMAQMAU	GMP#03370_CCDB-S5-0069	705468	488	281080	27
Inverhuron	03-Sep-14	17-Sep-14	MBR-OPPMAQMAU	GMP#04560_CCDB-S5-0069	685601	332	208310	20
Johnston Harbour - Pine Tree Point	01-May-14	15-May-14	MBR-OPPMAX	GMP#03387_CCDB-S5-0080	724654	291	188683	29
Johnston Harbour - Pine Tree Point	15-May-14	29-May-14	MBR-OPPMAX	GMP#03388_CCDB-S5-0080	769501	509	216000	32
Johnston Harbour - Pine Tree Point	29-May-14	12-Jun-14	MBR-OPPMAX	GMP#03389_CCDB-S5-0080	737162	509	223530	18
Johnston Harbour - Pine Tree Point	12-Jun-14	26-Jun-14	MBR-OPPMAX	GMP#03390_CCDB-S5-0080	807198	362	325949	27
Johnston Harbour - Pine Tree Point	26-Jun-14	10-Jul-14	MBR-OPPMAX	GMP#03391_CCDB-S5-0080	691388	530	202381	22
Johnston Harbour - Pine Tree Point	10-Jul-14	24-Jul-14	MBR-OPPMAX	GMP#03392_CCDB-S5-0080	671234	658	174502	19
Johnston Harbour - Pine Tree Point	24-Jul-14	07-Aug-14	MBR-OPPMAX	GMP#03393_CCDB-S5-0080	673577	525	186278	26
Johnston Harbour - Pine Tree Point	07-Aug-14	21-Aug-14	MBR-OPPMAX	GMP#03394_CCDB-S5-0080	687943	556	190237	33
Johnston Harbour - Pine Tree Point	21-Aug-14	04-Sep-14	MBR-OPPMAX	GMP#04511_CCDB-S5-0080	678894	511	189490	28
Johnston Harbour - Pine Tree Point	04-Sep-14	18-Sep-14	MBR-OPPMAX	GMP#04563_CCDB-S5-0080	671947	348	201213	19
Lions Head	15-May-14	29-May-14	MBR-OPPMAWMAZ	GMP#03396_CCDB-S5-0070	955478	468	376080	39
Lions Head	12-Jun-14	26-Jun-14	MBR-OPPMAWMAZ	GMP#03398_CCDB-S5-0070	873506	435	324209	30
Lions Head	10-Jul-14	24-Jul-14	MBR-OPPMAWMAZ	GMP#03400_CCDB-S5-0070	866966	636	340401	27

Lions Head	07-Aug-14	21-Aug-14	MBR-OPPMAWMAZ	GMP#03402_CCDB-S5-0070	899173	585	335398	35
Lions Head	04-Sep-14	18-Sep-14	MBR-OPPMAWMAZ	GMP#04564_CCDB-S5-0070	799233	419	536222	43
MacGregor Point	01-May-14	14-May-14	MBR-OPPMBB	GMP#03371_CCDB-S5-0082	882710	435	316325	34
MacGregor Point	14-May-14	28-May-14	MBR-OPPMBB	GMP#03372_CCDB-S5-0082	945078	535	386904	39
MacGregor Point	28-May-14	11-Jun-14	MBR-OPPMBB	GMP#03373_CCDB-S5-0082	921332	633	375495	39
MacGregor Point	11-Jun-14	25-Jun-14	MBR-OPPMBB	GMP#03374_CCDB-S5-0082	861726	728	333858	20
MacGregor Point	25-Jun-14	09-Jul-14	MBR-OPPMBB	GMP#03375_CCDB-S5-0082	814354	816	330629	26
MacGregor Point	09-Jul-14	23-Jul-14	MBR-OPPMBB	GMP#03376_CCDB-S5-0082	874746	811	321321	26
MacGregor Point	23-Jul-14	06-Aug-14	MBR-OPPMBB	GMP#03377_CCDB-S5-0082	771196	670	305828	27
MacGregor Point	06-Aug-14	20-Aug-14	MBR-OPPMBB	GMP#03378_CCDB-S5-0082	827106	732	308378	28
MacGregor Point	20-Aug-14	03-Sep-14	MBR-OPPMBB	GMP#04509_CCDB-S5-0082	863654	712	354738	20
MacGregor Point	03-Sep-14	17-Sep-14	MBR-OPPMBB	GMP#04561_CCDB-S5-0082	880169	464	351010	41
Mark S Burnham	05-May-14	23-May-14	MBR-OPPMBC	GMP#03755_CCDB-S5-0056	1080940	487	463735	30
Mark S Burnham	23-May-14	06-Jun-14	MBR-OPPMBC	GMP#03756_CCDB-S5-0056	1062250	391	487306	36
Mark S Burnham	06-Jun-14	20-Jun-14	MBR-OPPMBC	GMP#03757_CCDB-S5-0056	928450	449	422380	24
Mark S Burnham	20-Jun-14	04-Jul-14	MBR-OPPMBC	GMP#03758_CCDB-S5-0056	916766	405	391473	28
Mark S Burnham	04-Jul-14	18-Jul-14	MBR-OPPMBC	GMP#03759_CCDB-S5-0056	960472	495	440521	27
Mark S Burnham	18-Jul-14	01-Aug-14	MBR-OPPMBC	GMP#03760_CCDB-S5-0056	911696	477	406508	33
Mark S Burnham	01-Aug-14	15-Aug-14	MBR-OPPMBC	GMP#03761_CCDB-S5-0056	871916	492	398810	33
Mark S Burnham	15-Aug-14	29-Aug-14	MBR-OPPMBC	GMP#03762_CCDB-S5-0056	926145	360	333928	31
Mark S Burnham	29-Aug-14	12-Sep-14	MBR-OPPMBC	GMP#04541_CCDB-S5-0056	902908	388	286299	50
Mark S Burnham	12-Sep-14	26-Sep-14	MBR-OPPMBC	GMP#04593_CCDB-S5-0056	830300	308	343134	39
Morris Tract	14-May-14	28-May-14	MBR-OPPMAHMBD	GMP#03356_CCDB-S5-0086	709672	432	255906	18
Morris Tract	11-Jun-14	25-Jun-14	MBR-OPPMAHMBD	GMP#03358_CCDB-S5-0086	625540	494	209229	13
Morris Tract	09-Jul-14	23-Jul-14	MBR-OPPMAHMBD	GMP#03360_CCDB-S5-0086	698041	478	198459	27
Morris Tract	06-Aug-14	20-Aug-14	MBR-OPPMAHMBD	GMP#03362_CCDB-S5-0086	611572	451	173269	26
Morris Tract	03-Sep-14	17-Sep-14	MBR-OPPMAHMBD	GMP#04559_CCDB-S5-0086	610537	395	187651	17
Peters Woods	20-Jun-14	04-Jul-14	MBR-OPPMBZZ	GMP#03647_CCDB-S5-0052	763316	1207	325913	28
Peters Woods	05-May-14	23-May-14	MBR-OPPMBZZ	GMP#03771_CCDB-S5-0052	897942	940	370475	32
Peters Woods	23-May-14	06-Jun-14	MBR-OPPMBZZ	GMP#03772_CCDB-S5-0052	842295	1394	325496	19
Peters Woods	06-Jun-14	20-Jun-14	MBR-OPPMBZZ	GMP#03773_CCDB-S5-0052	775032	1537	302832	21
Peters Woods	04-Jul-14	18-Jul-14	MBR-OPPMBZZ	GMP#03775_CCDB-S5-0052	779646	1666	321307	26
Peters Woods	18-Jul-14	01-Aug-14	MBR-OPPMBZZ	GMP#03776_CCDB-S5-0052	707897	1629	293069	21

Peters Woods	01-Aug-14	15-Aug-14	MBR-OPPMBZZ	GMP#03777_CCDB-S5-0052	691282	915	284577	20
Peters Woods	15-Aug-14	29-Aug-14	MBR-OPPMBZZ	GMP#03778_CCDB-S5-0052	475431	204	198844	30
Peters Woods	29-Aug-14	12-Sep-14	MBR-OPPMBZZ	GMP#04543_CCDB-S5-0052	722631	911	317127	31
Peters Woods	12-Sep-14	26-Sep-14	MBR-OPPMBZZ	GMP#04595_CCDB-S5-0052	727262	652	308412	25
Presqu'ile	05-May-14	23-May-14	MBR-OPPMBJ	GMP#03731_CCDB-S5-0085	902173	629	332139	31
Presqu'ile	23-May-14	06-Jun-14	MBR-OPPMBJ	GMP#03732_CCDB-S5-0085	812687	711	328324	22
Presqu'ile	06-Jun-14	20-Jun-14	MBR-OPPMBJ	GMP#03733_CCDB-S5-0085	953592	969	364540	27
Presqu'ile	20-Jun-14	04-Jul-14	MBR-OPPMBJ	GMP#03734_CCDB-S5-0085	926873	1189	324161	26
Presqu'ile	04-Jul-14	18-Jul-14	MBR-OPPMBJ	GMP#03735_CCDB-S5-0085	845205	813	305776	25
Presqu'ile	18-Jul-14	01-Aug-14	MBR-OPPMBJ	GMP#03736_CCDB-S5-0085	887429	958	333925	30
Presqu'ile	01-Aug-14	15-Aug-14	MBR-OPPMBJ	GMP#03737_CCDB-S5-0085	819898	806	326999	18
Presqu'ile	15-Aug-14	29-Aug-14	MBR-OPPMBJ	GMP#03738_CCDB-S5-0085	924025	783	360982	26
Presqu'ile	29-Aug-14	12-Sep-14	MBR-OPPMBJ	GMP#04538_CCDB-S5-0085	836419	851	317948	31
Presqu'ile	12-Sep-14	26-Sep-14	MBR-OPPMBJ	GMP#04590_CCDB-S5-0085	877836	755	284547	40
Pretty River Valley	28-Apr-14	15-May-14	MBR-OPPMBK	GMP#03427_CCDB-S5-0071	801574	442	189413	30
Pretty River Valley	15-May-14	29-May-14	MBR-OPPMBK	GMP#03428_CCDB-S5-0071	713379	652	168434	23
Pretty River Valley	29-May-14	12-Jun-14	MBR-OPPMBK	GMP#03429_CCDB-S5-0071	712297	1020	220526	28
Pretty River Valley	12-Jun-14	26-Jun-14	MBR-OPPMBK	GMP#03430_CCDB-S5-0071	681139	973	209623	16
Pretty River Valley	26-Jun-14	10-Jul-14	MBR-OPPMBK	GMP#03431_CCDB-S5-0071	622961	1078	184194	21
Pretty River Valley	10-Jul-14	24-Jul-14	MBR-OPPMBK	GMP#03432_CCDB-S5-0071	624171	895	173160	17
Pretty River Valley	24-Jul-14	07-Aug-14	MBR-OPPMBK	GMP#03433_CCDB-S5-0071	673392	979	190338	19
Pretty River Valley	07-Aug-14	21-Aug-14	MBR-OPPMBK	GMP#03434_CCDB-S5-0071	718818	933	238525	22
Pretty River Valley	21-Aug-14	04-Sep-14	MBR-OPPMBK	GMP#04516_CCDB-S5-0071	671897	953	235804	23
Pretty River Valley	04-Sep-14	18-Sep-14	MBR-OPPMBK	GMP#04568_CCDB-S5-0071	693012	606	187564	20
Sandbanks	23-May-14	05-Jun-14	MBR-OPPMBMMBN	GMP#03724_CCDB-S5-0075	1031990	359	373650	23
Sandbanks	19-Jun-14	03-Jul-14	MBR-OPPMBMMBN	GMP#03726_CCDB-S5-0075	906681	408	388764	34
Sandbanks	17-Jul-14	31-Jul-14	MBR-OPPMBMMBN	GMP#03728_CCDB-S5-0075	919020	424	379444	38
Sandbanks	14-Aug-14	28-Aug-14	MBR-OPPMBMMBN	GMP#03730_CCDB-S5-0075	935484	380	419939	39
Sandbanks	11-Sep-14	25-Sep-14	MBR-OPPMBMMBN	GMP#04589_CCDB-S5-0075	907357	288	339848	28
Sibbald Point	01-May-14	19-May-14	MBR-OPPMBR	GMP#03763_CCDB-S5-0088	808088	421	280023	20
Sibbald Point	19-May-14	02-Jun-14	MBR-OPPMBR	GMP#03764_CCDB-S5-0088	900686	396	306367	22
Sibbald Point	02-Jun-14	16-Jun-14	MBR-OPPMBR	GMP#03765_CCDB-S5-0088_	837190	539	290440	29
Sibbald Point	16-Jun-14	30-Jun-14	MBR-OPPMBR	GMP#03766_CCDB-S5-0088	847666	598	304247	34

Sibbald Point	30-Jun-14	14-Jul-14	MBR-OPPMBR	GMP#03767_CCDB-S5-0088	796220	610	313575	33
Sibbald Point	14-Jul-14	28-Jul-14	MBR-OPPMBR	GMP#03768_CCDB-S5-0088	827020	510	309426	28
Sibbald Point	28-Jul-14	11-Aug-14	MBR-OPPMBR	GMP#03769_CCDB-S5-0088	732922	466	264246	27
Sibbald Point	11-Aug-14	25-Aug-14	MBR-OPPMBR	GMP#03770_CCDB-S5-0088	782761	410	268657	37
Sibbald Point	25-Aug-14	08-Sep-14	MBR-OPPMBR	GMP#04542_CCDB-S5-0088	763727	340	321601	42
Sibbald Point	08-Sep-14	22-Sep-14	MBR-OPPMBR	GMP#04594_CCDB-S5-0088	701369	281	239843	19
Wasaga Beach	29-Apr-14	16-May-14	MBR-OPPMBW	GMP#03435_CCDB-S5-0074	929191	346	467503	94
Wasaga Beach	16-May-14	30-May-14	MBR-OPPMBW	GMP#03436_CCDB-S5-0074	851075	334	440834	98
Wasaga Beach	30-May-14	13-Jun-14	MBR-OPPMBW	GMP#03437_CCDB-S5-0074	757829	380	405491	89
Wasaga Beach	13-Jun-14	27-Jun-14	MBR-OPPMBW	GMP#03438_CCDB-S5-0074	808294	394	363054	91
Wasaga Beach	27-Jun-14	11-Jul-14	MBR-OPPMBW	GMP#03439_CCDB-S5-0074	781852	494	396625	93
Wasaga Beach	11-Jul-14	25-Jul-14	MBR-OPPMBW	GMP#03440_CCDB-S5-0074	766615	488	385711	112
Wasaga Beach	25-Jul-14	08-Aug-14	MBR-OPPMBW	GMP#03441_CCDB-S5-0074	778874	478	373429	94
Wasaga Beach	08-Aug-14	22-Aug-14	MBR-OPPMBW	GMP#03442_CCDB-S5-0074	781876	508	377107	98
Wasaga Beach	22-Aug-14	05-Sep-14	MBR-OPPMBW	GMP#04517_CCDB-S5-0074	748086	497	399949	103
Wasaga Beach	05-Sep-14	19-Sep-14	MBR-OPPMBW	GMP#04569_CCDB-S5-0074	791391	335	407630	97
Bronte Creek	28-Apr-14	12-May-14	MBR-OPPMAJ	GMP#03243_CCDB-S5-0057	658058	323	230976	8
Bronte Creek	12-May-14	26-May-14	MBR-OPPMAJ	GMP#03244_CCDB-S5-0057	651019	471	206673	13
Bronte Creek	26-May-14	09-Jun-14	MBR-OPPMAJ	GMP#03245_CCDB-S5-0057	580340	788	219014	13
Bronte Creek	09-Jun-14	23-Jun-14	MBR-OPPMAJ	GMP#03246_CCDB-S5-0057	624816	773	233120	11
Bronte Creek	23-Jun-14	07-Jul-14	MBR-OPPMAJ	GMP#03247_CCDB-S5-0057	603783	1092	219138	9
Bronte Creek	07-Jul-14	21-Jul-14	MBR-OPPMAJ	GMP#03248_CCDB-S5-0057	704885	954	272582	15
Bronte Creek	21-Jul-14	04-Aug-14	MBR-OPPMAJ	GMP#03249_CCDB-S5-0057	631565	944	235867	14
Bronte Creek	04-Aug-14	18-Aug-14	MBR-OPPMAJ	GMP#03250_CCDB-S5-0057	671111	820	238253	16
Bronte Creek	18-Aug-14	01-Sep-14	MBR-OPPMAJ	GMP#04493_CCDB-S5-0057	634880	841	237862	9
Bronte Creek	01-Sep-14	15-Sep-14	MBR-OPPMAJ	GMP#04545_CCDB-S5-0057	668655	707	266055	9
James N Allan	28-Apr-14	12-May-14	MBR-OPPMAV	GMP#03274_CCDB-S5-0129	594922	489	167822	21
James N Allan	12-May-14	26-May-14	MBR-OPPMAV	GMP#03267_CCDB-S5-0129	611927	184	134643	12
James N Allan	26-May-14	09-Jun-14	MBR-OPPMAV	GMP#03268_CCDB-S5-0129	582525	259	138953	20
James N Allan	09-Jun-14	23-Jun-14	MBR-OPPMAV	GMP#03269_CCDB-S5-0129	687894	416	161299	29
James N Allan	23-Jun-14	07-Jul-14	MBR-OPPMAV	GMP#03270_CCDB-S5-0129	677540	613	159367	13
James N Allan	07-Jul-14	21-Jul-14	MBR-OPPMAV	GMP#03271_CCDB-S5-0129	596602	673	135699	16
James N Allan	21-Jul-14	04-Aug-14	MBR-OPPMAV	GMP#03272_CCDB-S5-0129	569798	682	133587	8

James N Allan	04-Aug-14	18-Aug-14	MBR-OPPMAV	GMP#03273_CCDB-S5-0129	562811	605	149439	14
James N Allan	18-Aug-14	01-Sep-14	MBR-OPPMAV	GMP#04496_CCDB-S5-0129	595953	570	153347	18
James N Allan	01-Sep-14	15-Sep-14	MBR-OPPMAV	GMP#04548_CCDB-S5-0129	538915	505	114538	10
John E Pearce	13-May-14	27-May-14	MBR-OPPMAWMAZ	GMP#03308_CCDB-S5-0070	836894	338	250266	40
John E Pearce	10-Jun-14	24-Jun-14	MBR-OPPMAWMAZ	GMP#03310_CCDB-S5-0070	1025380	382	223318	16
John E Pearce	08-Jul-14	22-Jul-14	MBR-OPPMAWMAZ	GMP#03312_CCDB-S5-0070	978009	371	214884	19
John E Pearce	05-Aug-14	19-Aug-14	MBR-OPPMAWMAZ	GMP#03314_CCDB-S5-0070	1117930	354	428952	46
John E Pearce	02-Sep-14	16-Sep-14	MBR-OPPMAWMAZ	GMP#04553_CCDB-S5-0070	863571	314	291674	34
Long Point	28-Apr-14	13-May-14	MBR-OPPMBY	GMP#03291_CCDB-S5-0041	1116840	273	461680	41
Long Point	13-May-14	27-May-14	MBR-OPPMBY	GMP#03292_CCDB-S5-0041	1047380	292	441793	46
Long Point	27-May-14	10-Jun-14	MBR-OPPMBY	GMP#03293_CCDB-S5-0041	1938610	467	760581	57
Long Point	10-Jun-14	24-Jun-14	MBR-OPPMBY	GMP#03294_CCDB-S5-0041	906817	496	338921	21
Long Point	24-Jun-14	08-Jul-14	MBR-OPPMBY	GMP#03295_CCDB-S5-0041	915831	578	291299	17
Long Point	08-Jul-14	22-Jul-14	MBR-OPPMBY	GMP#03296_CCDB-S5-0041	736485	148	229475	9
Long Point	22-Jul-14	05-Aug-14	MBR-OPPMBY	GMP#03297_CCDB-S5-0041	922361	490	296195	19
Long Point	05-Aug-14	19-Aug-14	MBR-OPPMBY	GMP#03298_CCDB-S5-0041	800802	160	381316	11
Long Point	19-Aug-14	02-Sep-14	MBR-OPPMBY	GMP#04499_CCDB-S5-0041	846228	242	305874	30
Long Point	02-Sep-14	16-Sep-14	MBR-OPPMBY	GMP#04551_CCDB-S5-0041	807700	382	311652	25
Ojibway Prairie	14-May-14	28-May-14	MBR-OPPMBFMBH	GMP#03332_CCDB-S5-0074	919805	590	299290	26
Ojibway Prairie	11-Jun-14	25-Jun-14	MBR-OPPMBFMBH	GMP#03334_CCDB-S5-0074	918899	1179	308083	23
Ojibway Prairie	09-Jul-14	23-Jul-14	MBR-OPPMBFMBH	GMP#03336_CCDB-S5-0074	804085	1045	251805	22
Ojibway Prairie	06-Aug-14	20-Aug-14	MBR-OPPMBFMBH	GMP#03338_CCDB-S5-0074	861740	914	279808	16
Ojibway Prairie	03-Sep-14	17-Sep-14	MBR-OPPMBFMBH	GMP#04556_CCDB-S5-0074	852877	732	310872	25
Pinery	14-May-14	28-May-14	MBR-OPPMBFMBH	GMP#03348_CCDB-S5-0074	930698	498	292992	40
Pinery	11-Jun-14	25-Jun-14	MBR-OPPMBFMBH	GMP#03350_CCDB-S5-0074	875855	615	247325	20
Pinery	09-Jul-14	23-Jul-14	MBR-OPPMBFMBH	GMP#03352_CCDB-S5-0074	941329	528	286145	33
Pinery	06-Aug-14	20-Aug-14	MBR-OPPMBFMBH	GMP#03354_CCDB-S5-0074	958263	424	319172	48
Pinery	03-Sep-14	17-Sep-14	MBR-OPPMBFMBH	GMP#04558_CCDB-S5-0074	919608	478	366344	26
Port Burwell	29-Apr-14	13-May-14	MBR-OPPMBI	GMP#03299_CCDB-S5-0084	1123850	231	419838	27
Port Burwell	13-May-14	27-May-14	MBR-OPPMBI	GMP#03300_CCDB-S5-0084	911682	222	221993	18
Port Burwell	27-May-14	10-Jun-14	MBR-OPPMBI	GMP#03301_CCDB-S5-0084	824772	199	195299	13
Port Burwell	10-Jun-14	24-Jun-14	MBR-OPPMBI	GMP#03302_CCDB-S5-0084	774369	216	191336	13
Port Burwell	24-Jun-14	08-Jul-14	MBR-OPPMBI	GMP#03303_CCDB-S5-0084	760139	382	199065	18

Port Burwell	08-Jul-14	22-Jul-14	MBR-OPPMBI	GMP#03304_CCDB-S5-0084	788998	359	210108	18
Port Burwell	22-Jul-14	05-Aug-14	MBR-OPPMBI	GMP#03305_CCDB-S5-0084	828756	169	315063	24
Port Burwell	05-Aug-14	19-Aug-14	MBR-OPPMBI	GMP#03306_CCDB-S5-0084	868031	225	283246	20
Port Burwell	19-Aug-14	02-Sep-14	MBR-OPPMBI	GMP#04500_CCDB-S5-0084	675841	140	198372	20
Port Burwell	02-Sep-14	16-Sep-14	MBR-OPPMBI	GMP#04552_CCDB-S5-0084	862757	243	231951	16
Rock Point	28-Apr-14	12-May-14	MBR-OPPMBL	GMP#03259_CCDB-S5-0072	839122	280	208018	20
Rock Point	12-May-14	26-May-14	MBR-OPPMBL	GMP#03260_CCDB-S5-0072	793412	330	192077	18
Rock Point	26-May-14	09-Jun-14	MBR-OPPMBL	GMP#03261_CCDB-S5-0072	700461	458	129223	13
Rock Point	09-Jun-14	23-Jun-14	MBR-OPPMBL	GMP#03262_CCDB-S5-0072	790337	699	177406	18
Rock Point	23-Jun-14	07-Jul-14	MBR-OPPMBL	GMP#03263_CCDB-S5-0072	788186	727	182834	18
Rock Point	07-Jul-14	21-Jul-14	MBR-OPPMBL	GMP#03264_CCDB-S5-0072	766060	660	196415	20
Rock Point	21-Jul-14	04-Aug-14	MBR-OPPMBL	GMP#03265_CCDB-S5-0072	691100	644	189498	24
Rock Point	04-Aug-14	18-Aug-14	MBR-OPPMBL	GMP#03266_CCDB-S5-0072	718469	614	207725	24
Rock Point	18-Aug-14	01-Sep-14	MBR-OPPMBL	GMP#04495_CCDB-S5-0072	722979	607	204088	25
Rock Point	01-Sep-14	15-Sep-14	MBR-OPPMBL	GMP#04547_CCDB-S5-0072	710206	458	219406	21
Rondeau	13-May-14	27-May-14	MBR-OPPMBMMBN	GMP#03316_CCDB-S5-0075	1009030	394	345559	31
Rondeau	10-Jun-14	24-Jun-14	MBR-OPPMBMMBN	GMP#03318_CCDB-S5-0075	1082920	457	299784	33
Rondeau	08-Jul-14	22-Jul-14	MBR-OPPMBMMBN	GMP#03320_CCDB-S5-0075	1057380	427	339094	38
Rondeau	05-Aug-14	19-Aug-14	MBR-OPPMBMMBN	GMP#03322_CCDB-S5-0075	1058500	322	364079	37
Rondeau	02-Sep-14	16-Sep-14	MBR-OPPMBMMBN	GMP#04554_CCDB-S5-0075	833385	372	250949	29
Selkirk	28-Apr-14	12-May-14	MBR-OPPMBO	GMP#03275_CCDB-S5-0087	843237	290	291443	26
Selkirk	12-May-14	26-May-14	MBR-OPPMBO	GMP#03276_CCDB-S5-0087	942794	350	274170	31
Selkirk	26-May-14	09-Jun-14	MBR-OPPMBO	GMP#03277_CCDB-S5-0087	840624	309	225261	24
Selkirk	09-Jun-14	23-Jun-14	MBR-OPPMBO	GMP#03278_CCDB-S5-0087_	882045	292	216876	31
Selkirk	23-Jun-14	07-Jul-14	MBR-OPPMBO	GMP#03279_CCDB-S5-0087	767299	379	169388	30
Selkirk	07-Jul-14	21-Jul-14	MBR-OPPMBO	GMP#03280_CCDB-S5-0087	772567	298	215244	27
Selkirk	21-Jul-14	04-Aug-14	MBR-OPPMBO	GMP#03281_CCDB-S5-0087	771385	306	192737	23
Selkirk	04-Aug-14	18-Aug-14	MBR-OPPMBO	GMP#03282_CCDB-S5-0087	826300	254	225597	33
Selkirk	18-Aug-14	01-Sep-14	MBR-OPPMBO	GMP#04497_CCDB-S5-0087	805237	193	316186	13
Selkirk	01-Sep-14	15-Sep-14	MBR-OPPMBO	GMP#04549_CCDB-S5-0087	723173	176	178140	17
Short Hills	12-May-14	26-May-14	MBR-OPPMBQMBU	GMP#03252_CCDB-S5-0079	784032	536	291126	28
Short Hills	09-Jun-14	23-Jun-14	MBR-OPPMBQMBU	GMP#03254_CCDB-S5-0079	742214	875	268908	20
Short Hills	07-Jul-14	21-Jul-14	MBR-OPPMBQMBU	GMP#03256_CCDB-S5-0079	714520	859	274769	18

Short Hills	04-Aug-14	18-Aug-14	MBR-OPPMBQMBU	GMP#03258_CCDB-S5-0079	750246	706	286871	26
Short Hills	01-Sep-14	15-Sep-14	MBR-OPPMBQMBU	GMP#04546_CCDB-S5-0079	740631	653	301043	24
Turkey Point	12-May-14	26-May-14	MBR-OPPMBQMBU	GMP#03284_CCDB-S5-0079	689965	542	218969	28
Turkey Point	09-Jun-14	23-Jun-14	MBR-OPPMBQMBU	GMP#03286_CCDB-S5-0079	710855	826	208956	11
Turkey Point	07-Jul-14	21-Jul-14	MBR-OPPMBQMBU	GMP#03288_CCDB-S5-0079	724419	747	202132	18
Turkey Point	04-Aug-14	18-Aug-14	MBR-OPPMBQMBU	GMP#03290_CCDB-S5-0079	670259	642	199365	21
Turkey Point	01-Sep-14	15-Sep-14	MBR-OPPMBQMBU	GMP#04550_CCDB-S5-0079	678548	527	193417	22
Wheatley	29-Apr-14	13-May-14	MBR-OPPMBX	GMP#03323_CCDB-S5-0097	1034310	273	383053	21
Wheatley	13-May-14	27-May-14	MBR-OPPMBX	GMP#03324_CCDB-S5-0097	799151	283	195785	13
Wheatley	27-May-14	10-Jun-14	MBR-OPPMBX	GMP#03325_CCDB-S5-0097	760957	242	177634	8
Wheatley	10-Jun-14	24-Jun-14	MBR-OPPMBX	GMP#03326_CCDB-S5-0097	785048	301	179129	9
Wheatley	24-Jun-14	08-Jul-14	MBR-OPPMBX	GMP#03327_CCDB-S5-0097	710855	375	170800	11
Wheatley	08-Jul-14	22-Jul-14	MBR-OPPMBX	GMP#03328_CCDB-S5-0097	778572	336	233055	21
Wheatley	22-Jul-14	05-Aug-14	MBR-OPPMBX	GMP#03329_CCDB-S5-0097	864601	213	282136	14
Wheatley	05-Aug-14	19-Aug-14	MBR-OPPMBX	GMP#03330_CCDB-S5-0097	844843	227	249372	20
Wheatley	19-Aug-14	02-Sep-14	MBR-OPPMBX	GMP#04503_CCDB-S5-0097	735171	275	206937	13
Wheatley	02-Sep-14	16-Sep-14	MBR-OPPMBX	GMP#04555_CCDB-S5-0097	689051	211	213927	26

Table S4: Wet weight (g) to insect lysis buffer volume (mL) ratios for Malaise trap bulk samples.

Wet Weight of Bulk Sample (g)	Insect Lysis Buffer Volume (mL)
<1.5	15
1.5-4.9	20
5.0-9.9	50
10.0-19.9	100
20.0-29.9	200
>30.0	250
