# Supplementary material for: Message in a Bottle—Metabarcoding enables biodiversity comparisons across ecoregions
Source: Gigascience. 2022 Apr 28;11:giac040. doi: 10.1093/gigascience/giac040 (PMC9049109; doi:10.1093/gigascience/giac040)
Supplement: giac040_GIGA-D-21-00198_Revision_1 [file giac040_giga-d-21-00198_revision_1.pdf]

GigaScience

Message in a Bottle – Metabarcoding Enables Biodiversity Comparisons Across Ecoregions

--Manuscript Draft--

**Manuscript Number:**	GIGA-D-21-00198R1
**Full Title:**	Message in a Bottle – Metabarcoding Enables Biodiversity Comparisons Across Ecoregions
**Article Type:**	Research
**Funding Information:**	ontario ministry of research, innovation and science — Dr Paul DN Hebert<br>canada foundation for innovation — Dr Paul DN Hebert<br>canada first research excellence fund — Dr Paul DN Hebert
**Abstract:**	Background<br><br>Traditional biomonitoring approaches have delivered a basic understanding of biodiversity, but they cannot support the large-scale assessments required to manage and protect entire ecosystems. This study employed DNA metabarcoding to assess spatial and temporal variation in species richness and diversity in arthropod communities from 52 protected areas spanning three Canadian ecoregions.<br><br>Results<br><br>This study revealed the presence of 26,263 arthropod species in the three ecoregions and indicated that at least another 3,000–5,000 await detection. Results further demonstrate that communities are more similar within than between ecoregions, even after controlling for geographical distance. Overall α-diversity declined from east to west, reflecting a gradient in habitat disturbance. Shifts in species composition were high at every site with turnover greater than nestedness, suggesting the presence of many transient species.<br><br>Conclusions<br><br>Differences in species composition among their arthropod communities confirm that ecoregions are a useful synoptic for biogeographic patterns and for structuring conservation efforts. The present results also demonstrate that metabarcoding enables large-scale monitoring of shifts in species composition, making it possible to move beyond the biomass measurements that have been the key metric employed in prior efforts to track change in arthropod communities.
**Corresponding Author:**	Dirk Steinke, Dr. rer. nat.<br>University of Guelph<br>Guelph, Ontario CANADA
**Corresponding Author Secondary Information:**	
**Corresponding Author's Institution:**	University of Guelph
**Corresponding Author's Secondary Institution:**	
**First Author:**	Dirk Steinke, Dr. rer. nat.
**First Author Secondary Information:**	
**Order of Authors:**	Dirk Steinke, Dr. rer. nat.<br>Stephanie L deWaard<br>Jayme E Sones<br>Natalia V Ivanova<br>Sean SW Prosser

Powered by Editorial Manager® and ProduXion Manager® from Aries Systems Corporation

	Kate Perez
	Thomas Wa Braukmann
	Megan Milton
	Evgeny V Zakharov
	Jeremy R deWaard
	Sujeevan Ratnasingham
	Paul DN Hebert
**Order of Authors Secondary Information:**	
**Response to Reviewers:**	Reviewer reports:<br>Reviewer #1: The manuscript is very well written and a great contribution to the field. However some analytical aspects need to be better described. Also, it would be great the authors provide their R-script in the supplementary material. Below my comments.<br><br>Line 166: R2 = 0.035 is very low, it needs to be better considered.<br>We changed the text so that it becomes clear that this is very low.<br>Lines 168-171: The alpha diversity comparison was based just in visual inspection or any test was made?<br>It was properly tested as described in the methods and the figure 4 caption (Kruskal-Wallis followed by Mann-Whitney post-hoc)<br>Lines 173-176: There was any test to significance? It need to be reported.<br>No, we added more explanation how to read this in the Figure 5 caption - usually for these plots significant differences between entities are detected when the peaks of the density plots do not overlap.<br>Lines 213-219: It is a nice discussion about local versus regional diversity, but very speculative, need at least some citations to support it.<br>It is speculative because not much is known about these traps despite a rather long history of use. We added two citations for more supportive context.<br>Lines 357-358: It reduce background contamination, you never can remove all.<br>we changed the text to indicate that it was done to reduce not remove all background contamination<br>Lines 365-367: How the distances were controlled, any analysis of spatial correlation?<br>This part of the sentence was misleading. We did not control for distances but (as shown in Figue 3a) looked at between vs across ecoregion border similarities across a range of distances between sites. We removed this part.<br>Lines 367_370: The NMDS was with abundance or presence/absence data? If it was abundance, any correction was applied?<br>It was done with presence/absence data. This is now clearer in the manuscript.<br>Lines 374-376: How the author checked the quality of the tree as it was made with very short fragment? the blackbox toll set all parameters on the model?<br>We added a sentence to mention the model that is used in RAxML Blackboxs (GTRCAT). It also uses a build-in bootstrap routine.<br>Line 382: Was there any correction to BINs table? Rarefaction, Shannon entropy? It is very necessary to metabarcoding data. Also why just BIN richness, other diversity measures may be included as Shannon or Fisher diversity on phyloseq, or the effective number of BINs with entropart.<br>Our intention was to show alpha-diversity for which BIN (MOTU) richness is perfectly sufficient. We also lacked some data (also as the result of the overall sample size) for proper use of phyloseq. The necessity of corrections (rarefaction, shannon entropy) is still debated especially with respect to which method to use. The study goal wasn't so much a contribution to this important aspect but we plan to use these and other data to further explore proper correction models.<br>Figure 1 needs a reference o Canada to better understand where the region is.<br>We changed the figure to include a small reference map of a part of Canada.<br><br><br>Reviewer #2: Steinke et al. used a metebarcoding method to investigate the species compositions for 410 insect bulk samples collected in 3 ecoregions. The manuscript is well written, all the materials and methods were clearly described, I think the manuscript should be accepted for publication after addressing several minor issues as

Powered by Editorial Manager® and ProduXion Manager® from Aries Systems Corporation

follows:

1. Line 126, as Ion torrent is not widely used nowadays, may the authors add some words regarding its sequencing length, error rate, throughput et al.
this has been done
2. Please unify the format of chao 1 (or chao-1).
this has been done
3. A rarefaction curve for each sample may need to check whether the species diversity is well represented by its raw reads.
We don't think that this would add anything useful to the study especially given the large quantity of filtered reads (as a consequence of using the IonTorrent System). For instance the presence of chimaeras would skew any plot. Aside from that, the study consists of 410 samples. Plots for each one of these would be way too many for any reader to deal with.
4. Line 187 - 191. This BIN number inflation may also boil down to sequence errors introduced during PCR amplification or sequencing.
we added a line to include these potential sources as well
5. Please pay attention to the citation format. For example, in line 202, reference # 40 should follow the first author's name.
this has been fixed
6. Line 226 - 227, please add some words to better explain the speculation of "passively transported by wind".
this has been done

Reviewer #3: General comments
Steinke et al used DNA metabarcoding of malaise trap samples from 52 protected areas spanning three Canadian ecoregions to assess the spatial patterns of arthropod biodiversity. The research question is relevant and interesting, the study is well designed, data collected are comprehensive, and manuscript is well written and easy to follow. I enjoyed reading it and would like to thank the authors for such a great contribution. My main concern is that the temporal aspect of the study was not explored even though it was mentioned as part of the research objective.
Specific comments
L60-62: These reductions are not only for abundance but also for diversity, at least based on the fourth reference cited here. I would therefore include "diversity" or "richness" in this statement.
this has been done
L63 & L105: The authors use biosurveillance in some places in the text and bio-surveillance in others. Isn't it better to stick to the same spelling all through, at least for consistency?
we corrected this
L132: I am a bit confused here. Are these "Analyses" or "Results"? The whole subsection from
L133-L176 read like results to me.
The manuscript format provided by GigaScience didn't suggest a Results section but rather Analyses. We do agree with the reviewer and made the change in the hopes that it is acceptable to the publisher
L329: "of" omitted! Five samples were available from each of the other 22 sites...
we corrected this
L332-334: The first "following" in this sentence can be either omitted or that part of the sentence completed using "manufacturer's instructions"
we corrected this
L345-346: "Reads were trimmed 30 bp from their 5' terminus with a set trim length of 450 bp". Perhaps this needs more clarification. The amplified length was 463 bp, trimming 30 bp gives 433 bp. How then can set trim length be 450 bp?
In fact, IonTorrent instruments produce reads of varying length, some of which are longer than the targeted amplicon size. mBRAVE does the front trim first and the does length filtering based on the trim length setting. This allowed some sequences (>433 and <450) through. Most of those are filtered out later in the routine because these are often chimaeras or other errors. We change the sentence slightly to indicate the the trim length setting function on mBRAVE is a length filter.
L348-349: What was the criterion for using "at least three reads matched an OTU in the reference database"? I mean why not at least two or at least four reads? If this was

arbitrary please clarify.
We had to correct this number to five (original was incorrect) but the number is indeed arbitrary resulting from experience with other datasets using mBRAVE and the IonTorrent platform. We added text to explain that.
L349-350: Same question as above, why use "a minimum of five reads per cluster"? It would be nice to indicate if any benchmarking was applied a priori or if this was set arbitrarily.
We added text to explain that we used benchmarking (unpublished)
L346-349: Since the authors were mostly interested in arthropods, were reads that matched sequences from bacteria (SYS-CRLBACTERIA), chordates (SYS-CRLCHORDATA) and non-arthropod invertebrates (SYS CRLNONARTHINVERT) discarded or retained? This should be mentioned here and estimates of the number of reads, BINs or OTUs matching each of these categories should be provided.
We added the numbers for each category to table S3 and clarified that all non-arthropod reads were discarded for the remainder of the analysis.
L149-153: These are interesting results. It would be nice to present them graphically, at least in the supplementary. The aim of the study was "to assess spatial and temporal variation in species richness and diversity in arthropod communities from 52 protected areas spanning three Canadian ecoregions" but the temporal aspect of the study was not fully explored. Although it is stated that "trap catches were harvested every second week from early May through September", this information has not be used in the analysis. Should the aim of the study be redefined and restricted to just spatial patterns then?
We generated a new supplementary Figure S2 to show the temporal variation of species richness for all sites. We also added a reference to it in the discussion section that discusses temporal variation.
L152-153: Without any table or figure to support these results, why not provide the actual number or proportion or percentage of BINs for each arthropod order in the text?
We generated a new supplementary Figure S3 to rectify this.
L157-158: Please add some symbols (e.g. asterisks *, **, ***or alphabet a, b, c) to Figure 3b to represent significant differences. Looking at the present figure without referring to the text does not tell the reader if the differences are significant. Besides, the authors only report a single p value ($p < 0.003$) which probably means at least one of the groups is different from the others but failed to report the pairwise multiple comparison tests that tell the reader which pairs or groups (e.g. ECF vs EGL, ECF vs SGL, EGL vs SGL) are significantly different.
We changed the text and added all p values for the three comparisons that were all significant. The figure was amended with a,b,c to indicate the same.
L159: Are the patterns similar if you control for the total number of sites per ecoregion? For example, taking 12 sites per ecoregion and resampling them 100 or 1000 times, similar to the approach used for beta diversity. It could be that one site is driving this pattern, as shown in Figure 2b and reported in L141 "…with more than a third (9,301) found at only one site (Figure 2b)".
Actually, both Figure 2b and L141 refer to the fact that 9301 BINs were only obtained at one site but not all at the same site. It rather means each BIN occurs only at one of all 52 sites.
L164-166: Please provide the full PERMANOVA results in a table in the text or supplementary and reference it here. It is not clear what "decreased site elevation (R2 166 =0.035, P =0.03)" means.
We added this as table S5
L168-171: Do these patterns change or remain the same if the same number of sites per ecoregion is used? This needs to be tested given that one site (probably from ECF or EGL?) is disproportionate species-rich and SGL has the lowest number of sites.
Again, there isn't a single site that is disproportionate species-rich. This can be clearly seen in Figure S2. We are using the same number of sites for each ecoregion here. We added a better description to the methods section.
L173-176: What about levels of turnover across time? Were they any temporal trends in alpha and beta diversity? Was the temporal dropped from the study objective and why?
We included a new supplementary figure S2 showing alpha diversity over the season for all sites.
L221-223: Same question as above, were temporal changes in species composition considered? Which results, tables or figures point to this or how did the authors arrive at these statements?

Powered by Editorial Manager® and ProduXion Manager® from Aries Systems Corporation

We did not explore temporal differences in species composition. Some parts of the discussion were toned done a bit to reflect this.

Reviewer #4: This manuscript assesses the variation in arthropod communities in three ecoregions in Canada. The study is well done, and the sampling was very thorough with a big sampling effort. I only have minor comments. Specially I consider that the aim can be focused on the ecoregions instead of the feasibility of the method, as this has already been shown. In addition, it would be nice to have more details in certain sections in the data analyses and in the results. I have addressed these comments below.

-I am not sure why the title "Message in a bottle".
That's both a reference to the collecting bottle of a Malaise trap and a famous song of the 1980s. We would like to keep it.
-Line 65- Could you specify which indicator species have been targeted? Or cite studies that target those species?
We included a new reference (a review paper on indicator species selection and use)
- Line 96- Based on the limitations of the ecoregions, it is not clear why ecoregions are an obvious candidate.
We expanded the text to better explain this
-In line 104 seems that your aim is to demonstrate how feasible is to use metabarcoding for large-scale monitoring and that you use the ecoregions to prove that. However, showing the feasibility of this method for large-scale studies has already been done (e.g. Svenningsen et al 2021, Detecting flying insects using car nets and DNA metabarcoding; Bush et al 2020, DNA metabarcoding reveals metacommunity dynamics in a threatened boreal wetland wilderness). I suggest keeping it focused on the need to apply this method in different ecoregions.
We changed the text to reflect this
-In the Data description section, you mention that you examined phylogenetic diversity, but in the Analyses section you vaguely mention it. The phylogenetic diversity findings are discussed later on, but it is difficult to follow the discussion when the results were not presented previously. In addition, the authors use the findings in phylogenetic diversity to support the idea of a structure in the ecoregions, so I suggest making more emphasis in this in the results section.
We do mention phylogenetic diversity in the Analyses/Results section (158-160 and Figure 3b). In fact. we describe the results of pairwise PD comparisons (Kruskal-Wallis and Dunn's posthoc tests) to show that there are differences in composition of ecoregions.
-Line 189. I agree that the higher number of BINs could be due to eDNA, but couldn't another reason be that the BINs were oversplit during data analysis?
we added a line to include sequence error as another potential source (also requested by reviewer 2)
-Line 215-217. Has this been found previously in other studies using Malaise trap? If so, please reference to those findings.
We don't think that it has been reported as such.
-Line222- This is a brief discussion about temporal turnover. However, these results are not presented previously, or at least not clearly enough.
we have added a new supplementary figure and text to rectify this (see responses to reviewer 3)
-Line 266-267- Yes, you showed compositional shifts using metabarcoding in bulk arthropod samples, but the way this sentence is structured it sounds like you are the first to show this. Compositional shifts in arthropods have been shown previously in other studies using metabarcoding.
we toned down this sentence accordingly
-Line 321- Did you have negative PCR controls? In line 326 you mention negative controls, but I assume you refer to the extraction negative controls.
no, we didn’t use negative PCR controls, the one we refer to is indeed an extraction control, we clarified that in the text
-Line 340- It is not clear why you queried the data against a bacterial library.
we added some text to explain that we screened for contamination by bacterial endosymbionts such as Wolbachia
-Line 348- What was the reason for choosing "at least three reads"? and the same for line 350 where you cluster sequences with a minimum of 5 reads per cluster.

Powered by Editorial Manager® and ProduXion Manager® from Aries Systems Corporation

	see response to reviewer 3 - we clarified this in the text<br>-Line 357- If you see tag switching in your negative controls that means that most likely you have it in the rest of the data. How did you ensure that the rest of the data did not have that? You may have tags switching in sequences not found in the negative controls but found in your samples.<br>That is possible, which is why we said "reduces the effects of tag switching" - the use of negative controls that are sequenced has been recommended. Other options such as the use of matching tags or PCR replicates were not used. We only had technical replicates. Some also list the careful handling of tagged amplicons which is a given for such studies.<br>-Line 369- As you used the Bray-Curtis index in this metabarcoding data, did you convert your data to presence/absence? It is known that for metabarcoding data the use of read numbers for community analysis is not adequate (see Nichols et al 2018 "Minimizing polymerase biases in metabarcoding").<br>Yes we did convert to p/a and used the resulting matrices for further analysis. This is has been included in the text.
**Additional Information:**	
**Question**	**Response**
Are you submitting this manuscript to a special series or article collection?	No
**Experimental design and statistics**<br><br>Full details of the experimental design and statistical methods used should be given in the Methods section, as detailed in our Minimum Standards Reporting Checklist. Information essential to interpreting the data presented should be made available in the figure legends.<br><br>Have you included all the information requested in your manuscript?	Yes
**Resources**<br><br>A description of all resources used, including antibodies, cell lines, animals and software tools, with enough information to allow them to be uniquely identified, should be included in the Methods section. Authors are strongly encouraged to cite Research Resource Identifiers (RRIDs) for antibodies, model organisms and tools, where possible.<br><br>Have you included the information requested as detailed in our Minimum Standards Reporting Checklist?	Yes

Powered by Editorial Manager® and ProduXion Manager® from Aries Systems Corporation

**Availability of data and materials**<br><br>All datasets and code on which the conclusions of the paper rely must be either included in your submission or deposited in publicly available repositories (where available and ethically appropriate), referencing such data using a unique identifier in the references and in the "Availability of Data and Materials" section of your manuscript.<br><br>Have you have met the above requirement as detailed in our Minimum Standards Reporting Checklist?	Yes

Powered by Editorial Manager® and ProduXion Manager® from Aries Systems Corporation

Manuscript

Click here to access/download;Manuscript;OPP_ecoregions_revision

Revision

Message in a Bottle – Metabarcoding Enables Biodiversity Comparisons Across Ecoregions

Steinke D[1,2*], deWaard SL[1], Sones, JE[1], Ivanova NV[1,2], Prosser SWJ[1], Perez K[1], Braukmann TWA[1], Milton M[1], Zakharov EV[1,2], deWaard JR[1,3], Ratnasingham S [1,2] Hebert PDN[1,2]

Affiliations:

[1]Centre for Biodiversity Genomics, University of Guelph, 50 Stone Road East, Guelph, Ontario, N1G 2W1, Canada

[2]Department of Integrative Biology, University of Guelph, 50 Stone Road East, Guelph, Ontario, N1G 2W1, Canada

[3]School of Environmental Sciences, University of Guelph, 50 Stone Road East, Guelph, Ontario, N1G 2W1, Canada

*Corresponding author: Dirk Steinke (dsteinke@uoguelph.ca)

Abstract

Background

Traditional biomonitoring approaches have delivered a basic understanding of biodiversity, but they cannot support the large-scale assessments required to manage and protect entire ecosystems. This study employed DNA metabarcoding to assess spatial and temporal variation in species richness and diversity in arthropod communities from 52 protected areas spanning three Canadian ecoregions.

Results

This study revealed the presence of 26,263 arthropod species in the three ecoregions and indicated that at least another 3,000–5,000 await detection. Results further demonstrate that communities are more similar within than between ecoregions, even after controlling for geographical distance. Overall α-diversity declined from east to west, reflecting a gradient in habitat disturbance. Shifts in species composition were high at every site with turnover greater than nestedness, suggesting the presence of many transient species.

Conclusions

Differences in species composition among their arthropod communities confirm that ecoregions are a useful synoptic for biogeographic patterns and for structuring conservation efforts. The present results also demonstrate that metabarcoding enables large-scale monitoring of shifts in species composition, making it possible to move beyond the biomass measurements that have been the key metric employed in prior efforts to track change in arthropod communities.

Background

Terrestrial organisms are exposed to diverse anthropogenic stressors, including climate change, resource extraction, and agriculture. Habitat degradation, pesticide usage, invasive species, and associated shifts in food webs have provoked major reductions in the diversity and abundance of terrestrial arthropods [1-4]. These declines have led to calls for more comprehensive biosurveillance to inform environmental management and conservation. Long-term monitoring of species composition is essential to quantify biological change, but efforts employing morphological diagnostics have targeted a small set of indicator species [5] because of the need for taxonomic experts for each group. As a consequence, they cannot support the broad assessments needed to manage and protect ecosystems, let alone forecast human impacts on them by integrating statistical modelling. The latter methods demand comprehensive data on species distributions and abundance [6], information that is currently unavailable because of the prior focus on selected biotic compartments at limited geographic scale.

Two methodological advances promise to meet the need for comprehensive biodiversity data. Firstly, identification systems based on the analysis of sequence variation in short, standardized gene regions (i.e., DNA barcodes) enable species discrimination [7]. Secondly, high-throughput sequencers (HTS) permit the inexpensive acquisition of millions of DNA barcode records [8]. These advances now enable biodiversity surveys at speeds and scales that were previously inconceivable. In particular, the coupling of HTS with DNA barcoding, known as metabarcoding [9], has a compelling advantage over traditional approaches for tracking shifts in species presence. It can generate georeferenced occurrence data from bulk samples at low cost, and a single instrument can process hundreds of bulk samples each week. Because the sequencing output of HTS is doubling every nine months [10,11], analytical costs are certain to sharply decline, allowing production to soar. This augmented capacity for data generation has already enabled large-scale biotic surveys of aquatic and terrestrial arthropods [12-15], vertebrates [16], pollen [17], diatoms [18], and fungi [19-21].

Access to large collections of specimens is essential to capitalize on the analytical capacity provided by DNA metabarcoding. Among the many approaches used to sample terrestrial arthropods, Malaise traps [22] have gained wide adoption because they collect large, diverse samples with little effort [23]. Although most-effective for sampling flying insects, they also collect ground-active arthropods. By coupling DNA barcoding with Malaise trapping [24,25], high-resolution monitoring networks for arthropods are within reach, but there are challenges. Data interpretation requires a well-parameterized DNA barcode reference library for the region under investigation, creating the need for a system to aid site selection. Ecoregions are designed to serve as spatial framework for the research, assessment, and monitoring of ecosystems and therefore represent a good candidate [26-29] although their boundaries are rarely sharply defined, and they are based on distributional data for a narrow range of taxa. Despite these limitations, ecoregions have been widely and successfully used to guide management decisions and to explore species and community diversity patterns [30,31]. As a result, they are a good candidate to serve as the backbone for a large-scale monitoring network. The most widely adopted schema partitions the world's 14 terrestrial biomes into 846 ecoregions [31].

This study demonstrates the feasibility of employing metabarcoding for the comparison of temporal and spatial patterns of arthropod communities in three of Canada's 47 terrestrial ecoregions: the Eastern Canadian Forest – Boreal Transition (ECF – 75,000 km^2), the Eastern Great Lakes Lowland Forests (EGL – 63,000 km^2), and the Southern Great Lakes Forests (SGL – 22,000 km^2) (Figure 1). Forest cover declines from 77.7% in the ECF to 30.1% in the EGL and just 12.1% in the SGL while cropland/pastures cover 78% of the SGL, 57% of the EGL, and 3% of the ECF [31]. The EGL and SGL are the most populated ecoregions in Ontario with developed land (e.g., urban, road networks) encompassing more than 7% of the SGL [32]. As such, these ecoregions provide a good basis for assessing the impacts of varied disturbance regimes on biodiversity.

Data Description

Collections were made by deploying a Malaise trap at 52 sites in these three ecoregions and samples were metabarcoded to examine variation in their species richness, community

composition, phylogenetic diversity, as well as alpha (α) and beta (β)-diversity. Malaise traps were deployed for 20 weeks at 15 sites in the ECF, 24 sites in the EGL, and 13 sites in the SGL. Catches were harvested at two-week intervals and 410 of the resultant 520 samples were designated for metabarcoding (the others were reserved for single specimen barcoding). Analysis began with non-destructive lysis of the specimens in each bi-weekly sample, followed by DNA extraction using a membrane-based protocol [33]. A 463 bp amplicon of cytochrome *c* oxidase I (COI) was then PCR amplified and the amplicon pools from each set of 10 samples were sequenced on an Ion Torrent S5 using a 530 chip with a maximum read length output of 600bp. This chipset usually produces 9-12 Million reads of varying length at a 1-2% error rate. The sequences were subsequently analyzed using the Multiplex Barcode Research And Visualization Environment (mBRAVE – mbrave.net). All raw HTS datasets were deposited in the Sequence Read Archive (SRA – www.ncbi.nlm.nih.gov/sra/) under the BioProject accession number PRJNA629553.

Results

Sequence analysis of the 410 samples produced 367,823,207 reads across 41 S5 runs (mean reads per run = 8.97 million, see **Table S1**). Two thirds were filtered, leaving 126,253,260 reads that could be assigned to a BIN (Barcode Index Number; [34]) on BOLD [35] (**Figure S1**). Nearly all reads (99.3 %) found a BIN match on BOLD, but those that failed were *de novo* clustered using mBRAVE with a 99% similarity threshold. The latter analysis recognized an average of 28 additional OTUs per sample, but >96% of them reflected sequencing/PCR errors (e.g., chimeras, sequences with multiple indels) or NUMTs so they were excluded from further analysis. Consideration of the assigned reads revealed 26,263 BINs among the 52 sites with more than a third (9,301) found at only one site, respectively (**Figure 2b**).

The Chao 1 [36] estimate for the total number of BINs present at the 52 sites was 29,640 (**Figure 2a**) while species richness extrapolation based on the lognormal distribution (**Figure 2c**, [37]) suggested the presence of 31,516 BINs. On average, 0.3 million sequences were recovered per sample, and they revealed the presence of an average of 2,352 BINs per site (range 996–4,581 BINs, **Table S2**) with bi-weekly samples

containing an average of 619±14.3 S.E. BINs (range 60–1666, **Table S3**). Most low BIN counts occurred in spring (May) or fall (September) with diversity peaking in mid-summer (June/July) (**Figure S2**). Taxonomic composition at an ordinal level was similar among samples with over half of the BINs being flies (Diptera), followed by Hymenoptera, Lepidoptera, Hemiptera, and Coleoptera (**Figure S3**).

Overlap in BIN composition was higher among parks in an ecoregion than among those in different ecoregions, even after geographical distance was considered (**Figure 3a**). Sites in the ECF had the highest mean phylogenetic diversity followed by EGL and finally SGL (**Figure 3b**), differences that were significant for all pairwise comparisons (KW and Dunn's posthoc $p < 0.005$ for ECF/EGL, $p < 0.003$ for ECF/SGL, $p < 0.05$ for EGL/SGL). More BINs were collected in the ECF (14,001) than in the EGL (12,787) or SGL (10,958) (Figure 3c). The Chao 1 estimates for the number of BINs present in each ecoregion were 15,401 for ECF, 14,577 for EGL, and 12,602 for SGL. The three ecoregions shared 4,133 BINs while about a third of those in each region were not collected elsewhere. A two-dimensional NMDS Ordination plot revealed that BIN assemblages for sites in each ecoregion formed cohesive groupings (Figure 3d). PERMANOVA analysis also suggested that community structure varied between ecoregions ($R^2 = 0.141$, $P = 0.0001$) and minimally with decreasing site elevation ($R^2 = 0.035$, $P = 0.03$) (**Table S4**).

Overall, α-diversity was highest in the ECF, intermediate in the EGL, and lowest in SGL (**Figure 4**). The α-diversity patterns for the varied insect orders followed the overall trend, but BIN richness for Collembola showed the opposite trend as it peaked in the SGL, while spider α-diversity was highest in the EGL.

Levels of turnover (**Figure 5**) were generally high among sites (species replacement by new species not found elsewhere) as well as high nestedness levels (gain and loss of species also found elsewhere). Lower levels of both turnover and nestedness were observed for most taxa at sites in the ECF while the highest values were found in the SGL.

Discussion

This study used metabarcoding to examine the species represented in 410 Malaise trap samples derived from 52 protected sites in three juxtaposed Canadian ecoregions. Metabarcoding revealed 26,263 species of arthropods while Chao 1 and Preston lognormal extrapolations indicated that another 3,000–5,000 species await detection. As just 52 sites were surveyed, a more comprehensive sampling program in these ecoregions might reveal as many as 50,000 species of arthropods. Nearly 5-fold variation (996–4,581) in BIN counts were detected among sites; counts showed a similar range for the 30 sites where all samples were analyzed (996–4,508) and the 22 where just half were metabarcoded (1,312–4,581). On average, 619 BINs were recovered from each metabarcoded sample, a count that was 52.5% higher than the mean BIN count (406) for samples that were barcoded using the Pacific Biosystem Sequel platform (Steinke et al. in prep). This difference suggests that more than half the BINs recovered from metabarcoded samples derive from environmental DNA attached to specimens in the sample, from their gut contents or from sequence errors that escaped the stringent filtering conditions.

The three ecoregions examined in this study collectively span 160,000 km^2, just 1.6% of Canada's land surface, but two (SGL, EGL) are among the most heavily populated areas in the country [32]. The ecoregions showed considerable overlap in species composition; 33.1% of the BINs recorded from three or more sites were shared by the three ecoregions. BIN richness was lowest in the southernmost ecoregion (SGL) and highest in the most northerly (ECF). This difference coincided with a disturbance gradient -- from forested regions with low human density in the ECF (78% forest cover) to disturbed landscapes dominated by farmland/cities in the SGL (12% forest cover). The decline in species richness in response to disturbance is consistent with earlier studies [38-40], even though our collections all derived from protected areas. Gray et al. [41] reported that protected sites contain significantly higher species counts than adjacent disturbed areas, perhaps because communities in protected areas include representatives of original habitats and generalists from adjacent disturbed landscapes [42]. However, protected areas in the SGL were small islands of remnant forest in a landscape dominated by agricultural activity so they were undoubtedly heavily exposed to pesticides with agricultural fields creating dispersal barriers which further reduced diversity.

Our results indicate that α-diversity for major insect orders of flying insects (Diptera, Hymenoptera, Hemiptera, Lepidoptera) peaked in the least disturbed ecoregion (ECF). By contrast, two groups of arthropods (Araneae, Collembola) lacking flight showed a different trend with their diversity peaking in other ecoregions. This difference might reflect the fact that Malaise traps only sample flightless taxa with resident populations near the trap but capture flying insects from distant habitats. As such, biodiversity patterns for flying insects provide a regional perspective while those for taxa without flight provide a local perspective [25,43]. If so, the reduction in diversity of Collembola from the most southerly (SGL) to northerly (ECF) ecoregion might reflect the expected latitudinal gradient in biodiversity, undisrupted by disturbance because of the local source of specimens in each sample.

The present study establishes the feasibility of monitoring changes in species composition of arthropod communities [44,45]. For all three ecoregions, temporal turnover was high, reflecting the seasonal succession of species. Species richness was lower at the beginning and end of the season and peaked in the summer months (**Figure S2**). β-diversity was lowest for most taxonomic groups at sites in the ECF and highest in the SGL. Species turnover was generally higher than nestedness, suggesting the presence of many transient species [46]. As many species were only collected at one or two sites (**Figure 2b**), many samples likely included transients passively transported by the wind [47]. Wingless and small insects generally depend on air currents to carry them to new sites and the malaise trap can function as a windbreak.

Metabarcoding can already provide cost-effective biosurveillance as the present study analyzed about 856,000 specimens and generated 223,860 species occurrence records for $82,000, an analytical cost of less than $0.50 per record. By adopting simpler analytical protocols (e.g., destructive processing of samples) with ongoing reductions in sequencing costs [11], costs can be reduced by an order of magnitude, delivering species occurrence records for $0.04 apiece in the ecoregions targeted in this study. In settings with higher α-diversity, the cost could be halved. Aside from its cost-effectiveness for data acquisition, the digital format of metabarcoding results aids their curation, validation, and preservation.

Although current metabarcoding protocols cannot estimate the abundance of each species in a sample, the situation shifts when multiple samples are analyzed as the abundance of a species can then be estimated from its frequency of occurrence in these samples (rare species will be recovered less frequently than abundant taxa).

As the 846 currently recognized ecoregions [31] were largely delineated based on distributional data for vascular plants and vertebrates, there remains a need to ascertain how well they represent diversity patterns in other taxa. [48] found that arthropods showed weak adherence to ecoregion boundaries and proposed this might reflect dispersal limitations linked to their small body size or to the biased assemblage of arthropod species with data. Our much larger dataset shows evidence of structuring by ecoregion as both phylogenetic diversity and BIN composition were significantly different among ecoregions, even when comparisons extended to widely separated sites. This result suggests that ecoregions do provide a useful structural framework, reinforcing results from earlier studies [49,50]. However, a third of species in this study crossed ecoregion boundaries and more extensive sampling would raise the incidence of shared species. The latter results make it clear that high sampling effort is required to better understand species distributions. In looking to the future, it is apparent that there is an immediate need for a more detailed understanding of the levels of species overlap between adjacent ecoregions. Is, for example, the pattern of high overlap in species composition among neighbouring ecoregions detected in this study a general pattern or are some ecoregion boundaries sharply delineated? Such information is critical in designing an effective global biomonitoring network to inform conservation efforts [51,52].

Potential Implications

Past monitoring programs have provided limited insights into the shifting distributions and abundances of arthropod species [53]. By coupling the use of an efficient collection method with the capacity of DNA metabarcoding to determine the species composition of bulk samples, this study confirms that compositional shifts in arthropod communities can be tracked using DNA metabarcoding [54]. The present results also indicate that the

ecoregion concept not only furthers understanding of foundational biogeographic principles and improves their potential application to conservation efforts, but also provides a logical scaffold for large-scale monitoring networks.

Methods

Sample collection

An ez-Malaise trap (BioQuip Products) was deployed to collect arthropods at one site in each of 50 provincial parks while two sites were sampled in the final park (Algonquin) because of its large size. Trap catches were harvested every second week from early May through September, producing 10 samples per site for a total of 520 samples. These samples were preserved in 95% ethanol and held at -20° C until DNA extraction. Five samples (weeks 1+2, 5+6, 9+10, 13+14, 17+18) from each of 22 sites were employed for single specimen barcoding (Steinke et al., in prep) while the other 410 samples were analyzed in this study. A direct count indicated that 230,000 specimens were present in the 21.2% of the samples that were barcoded. On this basis, the remaining samples (78.8%), those examined in this study, included about 856,000 specimens.

DNA extraction and PCR

DNA extraction employed a membrane-based protocol [33] modified for bulk samples. Specimens were removed from ethanol by filtration through a sterile Microfunnel 0.45 μM Supor Membrane Filter (Pall Laboratory) using a 6-Funnel Manifold (Pall Laboratory). The wet weight of each sample was then ascertained to allow volume adjustment (**Table S5**) of the lysis buffer [33]. Each sample was then incubated overnight at 56°C while gently mixed on a shaker. Eight 50 μl aliquots (technical replicates) from each of the 410 lysates were then transferred into 3,280 separate wells in 96-well microplates and DNA extracts were generated using Acroprep 3.0 μm glass fiber/0.2 μm Bio-Inert membrane plates (Pall Laboratory). Each plate contained 80 lysate samples, 8 technical replicates of a positive control (lysate from a bulk sample whose component specimens were individually Sanger sequenced – public BOLD dataset - dx.doi.org/10.5883/DS-RRNGS) and 8 negative controls. Each lysate was mixed with 100

µl of binding mix, transferred to a column plate, and centrifuged at 5000 g for 5 min. DNA was then purified with three washes; the first employed 180 µl of protein wash buffer centrifuged at 5000 g for 5 min. Each column was then washed twice with 600 µl of wash buffer centrifuged at 5000 g for 5 min. Columns were transferred to clean tubes and spun dry at 5000 g for 5 min to remove residual buffer before their transfer to clean collection tubes followed by incubation for 30 min at 56°C to dry the membrane. DNA was subsequently eluted by adding 60 µl of 10 mM Tris-HCl pH 8.0 followed by centrifugation at 5000 g for 5 min.

PCR reactions employed a standard protocol [55]. Briefly, each reaction included 5% trehalose (Fluka Analytical), 1× Platinum Taq reaction buffer (Invitrogen), 2.5 mM $MgCl_2$ (Invitrogen), 0.1 µM of each primer (Integrated DNA Technologies), 50 µM of each dNTP (KAPA Biosystems), 0.3 units of Platinum Taq (Invitrogen), 2 µl of DNA extract, and Hyclone ultra-pure water (Thermo Scientific) for a final volume of 12.5 µl. Two-stage PCR was used to generate amplicon libraries for sequencing on an Ion Torrent S5 platform. The first round of PCR used the primer combination AncientLepF3 [56] and LepR1 [57] to amplify a 463 bp fragment of COI. Prior to the second PCR, first round products were diluted 2x with ddH_2O. Fusion primers were then used to attach platform-specific unique molecular identifiers (UMIs) along with the sequencing adaptors required for Ion Torrent S5 libraries. Both rounds of PCR employed the same thermocycling conditions: initial denaturation at 94 °C for 2 min, followed by 20 cycles of denaturation at 94°C for 40 sec, annealing at 51°C for 1 min, and extension at 72 °C for 1 min, with a final extension at 72°C of 5 min.

HTS library construction

For each plate, labelled products were pooled prior to sequencing. In total, 41 libraries were assembled. Each included eight technical replicates of 10 samples plus eight technical replicates of an extraction negative and a positive control respectively (i.e., 96 samples). The ten samples from each of the 30 sites that were only metabarcoded, together with positive and negative controls, were pooled after UMI tagging to create a library that was analyzed on a 530 chip (30 chips in total). Five samples were available from each of the

other 22 sites (where half the samples were retained for barcoding). The UMI-tagged amplicons from five samples from each of two sites were pooled with positive and negative controls to produce a single library. Amplicon libraries were prepared on an Ion Chef (Thermo Fisher Scientific) and sequenced on an Ion Torrent S5 platform at the Centre for Biodiversity Genomics following manufacturer's instructions (Thermo Fisher Scientific).

Sequence analysis

Reads from the eight replicates for each sample were concatenated using a bash script and uploaded to mBRAVE (http://mbrave.net/) for quality filtering and subsequent queries using several reference libraries in an open reference approach. All reads were queried against five system libraries on mBRAVE: bacteria (SYS-CRLBACTERIA) to screen for potential contamination, e.g., by endosymbionts such as *Wolbachia*, chordates (SYS-CRLCHORDATA), insects (SYS-CRLINSECTA), non-insect arthropods (SYS-CRLNONINSECTARTH), and non-arthropod invertebrates (SYS-CRLNONARTHINVERT). All non-arthropod reads were discarded for further analysis. Sequences were only included in this analysis if they possessed a minimum length >350 bp and met the following three quality criteria (Mean QV >20; <25% positions with a QV<20; <5% positions with QV<10). Reads were trimmed 30 bp from their 5' terminus with a set trim length filter of 450 bp. Reads were matched to the sequences in each reference library with an ID distance threshold of 3%, but were only retained for further analysis when at least five reads matched an OTU in the reference database. This number is based on earlier benchmarking of the assignment algorithm on mBRAVE and IonTorrent generated sequences provided the best compromise between removing error and retaining real matches. All reads failing to match any sequence in the five reference libraries were clustered at an OTU threshold of 1% with a minimum of five reads per cluster, again a value based on initial benchmarking. All raw data are available in the NCBI Short Read Archive (PRJNA629553).

Using mBRAVE, we generated BIN (and OTU) tables including all library queries for each individual plate/run (10 samples, plus a negative and positive control - dx.doi.org/10.5883/DS-RRNGS - for each run). Read counts for any BINs recovered from the negative control on a plate were subtracted from the counts for the same BIN in the 80

non-control wells in the run. When this subtraction reduced the read count for a BIN to zero, its occurrence was removed. This step reduced the effects of rare tag switching on data integrity [58] and to reduce background contamination.

Ecoregion analysis

OTU tables were converted to presence/absence matrices. To determine the completeness of sampling, we calculated accumulation curves and the Chao 1 estimator for total diversity [36] using the vegan package [59]. For further extrapolation of species richness, we used the lognormal species abundance distribution [37]. The fit of Fisher's Logseries [60] was used to determine relative BIN abundance. Both methods are implemented in vegan (fisherfit, prestonfit) [59]. We calculated Sørensen's similarity coefficient to ascertain if differences in species assemblages were greater between or across ecoregion borders. Differences in BIN composition among the three ecoregions were examined using non-metric multidimensional scaling (NMDS) with the Bray-Curtis index coefficient as implemented in vegan [59]. The adonis function of the vegan package was used to conduct a Permutational Multivariate Analysis of Variance (PERMANOVA) to partition distance matrices among sources of variation (factors such as elevation, and ecoregion).

A Maximum likelihood phylogeny was inferred for a BIN sequence alignment using RAxML Black box [61] on XCEDE via the CIPRES portal [62]. This system uses a GTRCAT model which is recommended for larger datasets. The resulting phylogeny comprising 26,263 BIN sequences was used to calculate Faith's phylogenetic distance (PD) [63] using the picante package [64]. Because this measure is influenced by polytomies in a phylogeny [65], only one representative was included per BIN to avoid bias introduced by variation in the number of records for each BIN. A Kruskal-Wallis test followed by a Dunn's posthoc analysis was used to determine if significant PD differences existed between ecoregions.

Alpha (α)-diversity was quantified as the number of BINs observed at a site. It was calculated using 12 random sites from the total sites for each ecoregion. Pairwise BIN diversity among ecoregions was evaluated using the nonparametric multiple comparison function implemented in the R package dunn.test 1.2.4 [66]. dunn.test is equivalent to the

Kruskall–Wallis and pair-wise Mann–Whitney post hoc tests with Bonferroni correction. Beta (β)-diversity was computed as multi-site Sorensen and Simpson indices using the betapart 1.3. package [67]. β-diversity calculations between pairs of ecoregions were computed using 12 random sites from the total pool of sites for each ecoregion, and resampled 1000 times. We then split among-site β-diversity into turnover and nestedness.

All analyses were performed in R v.3.4.4 [68].

Data availability

All raw HTS datasets are deposited in the Sequence Read Archive (SRA – www.ncbi.nlm.nih.gov/sra/) under the BioProject accession number PRJNA629553. Additional supporting data and materials are available on the GigaScience database.

Funding

This study was enabled by awards to PDNH from the Ontario Ministry of Research, Innovation and Science, the Canada Foundation for Innovation, and by a grant from the Canada First Research Excellence Fund to the University of Guelph's "Food From Thought" research program.

Author contributions

DS, EVZ, JRDW, PDNH designed the study. DS, JRDW, JES, KP coordinated the study. SLDW, NVI, SWJP, TWAB did the bench work and contributed to analyses. SR and MM oversaw database organisation. DS did the analyses and wrote the manuscript. PDNH, JRDW, EVZ, TWAB revised the manuscript.

Acknowledgements

We thank the collections and sequencing staff at the Centre for Biodiversity Genomics for acquiring and processing the specimens analyzed in this study. We are very grateful to Suz Bateson for improving the figures and to staff at the participating Ontario Provincial Parks for facilitating collections.

References

1. Hallmann CA, Sorg M, Jongejans E, Siepel H, Hofland N, Schwan H, Stenmans W, Müller A, Sumser H, Hörren T, Goulson D, de Kroon H. More than 75 percent decline over 27 years in total flying insect biomass in protected areas. *PLoS ONE*. 2017; 12(10): e0185809.

2. Lister BC, Garcia A. Climate-driven declines in arthropod abundance restructure a rainforest food web. *Proceedings of the National Academy of Sciences of the United States of America*. 2018; 115(44): E10397–E10406.

3. Macgregor CJ, Williams JH, Bell JR, Thomas CD. Moth biomass increases and decreases over 50 years in Britain. *Nature Ecology and Evolution.* 2019; 3: 1645–1649.

4. Seibold S, Gossner MM, Simons NK, Blüthgen N, Müller J, Ambarli D, Ammer C, Bauhus J, Fischer M, Habel JC, Linsenmair KE, Nauss T, Penone C, Prati D, Schall P, Schulze E-D, Vogt J, Wöllauer S, Weisser WW. Arthropod decline in grasslands and forests is associated with drivers at landscape level. *Nature.* 2019; 574: 671–674

5. Siddig AAH, Ellison AM, Ochs A, Villar-Leeman, Lau MK. How do ecologists select and use indicator species to monitor ecological change? Insights from 14 years of publication in Ecological Indicators. 2016; 60: 223-230.

6. Bush A, Sollmann R, Wilting A, Bohmann K, Cole B, Balzter H, Martius C, Zlinszky A, Calvignac-Spencer S, Cobbold CA, Dawson TP, Emerson BC, Ferrirer S, Gilbert MTP, Herold M, Jones L, Leendertz FH, Matthews L, Millington JDA, Olson JR, Ovaskainen O, Raffaelli D, Reeve R, Rödel M-O, Rodgers TW, Snape S, Visseren-Hamakers I, Vogler AP, White PCL, Wooster MJ, Yu DW. Connecting Earth observation to high-throughput biodiversity data. *Nature Ecology & Evolution.* 2017; 1: 0176.

7. Hebert PDN, Cywinska A, Ball SL, deWaard JR. Biological identifications through DNA barcodes. *Proceedings of the Royal Society B: Biological Science.* 2003; 270: 313–321.

8. Hebert PDN, Braukmann TWA, Prosser SWJ, Ratnasingham S, deWaard JR, Ivanova NV, Janzen DH, Hallwachs W, Naik S, Sones JE, Zakharov EV. A Sequel to Sanger: amplicon sequencing that scales. *BMC Genomics*. 2018; 19: 219.

9. Taberlet P, Coissac E, Pompanon F, Brochmann C, Willerslev E. Towards next-generation biodiversity assessment using DNA metabarcoding. *Molecular Ecology*. 2012; 21(8): 2045-2050.

10. O'Driscoll A, Daugelaite J, Sleator RD. 'Big Data', Hadoop and cloud computing in genomics. *Journal of Biomedical Informatics*. 2013; 46(5): 774–781.

11. Lightbody G, Haberland V, Browne F, Taggart L, Zheng H, Parkes E, Blayney JK. Review of applications of high-throughput sequencing in personalized medicine: barriers and facilitators of future progress in research and clinical application. *Briefings in Bioinformatics*. 2019; 20(5): 1795–1811.

12. Ji C, Chng KR, Hui Boey EJ, Ng AHQ, Wilm A, Nagarajan N. INC-Seq: accurate single molecule reads using nanopore sequencing. *Gigascience*. 2016; 5: 34.

13. Beng KC, Tomlinson KW, Shen XH, Surget-Groba Y, Hughes AC, Corlett RT, Slik JWF. The utility of DNA metabarcoding for studying the response of arthropod diversity and composition to land-use change in the tropics. *Scientific Reports*. 2016; 6: 1–13.

14. Elbrecht V, Vamos EE, Meissner K, Aroviita J, Leese F. Assessing strengths and weaknesses of DNA metabarcoding-based macroinvertebrate identification for routine stream monitoring. *Methods in Ecology and Evolution*. 2017; 8: 1–21.

15. D'Souza ML, van der Bank M, Zandisile S, Rattray RD, Stewart R, van Rooyen J, Govender D, Hebert PDN. Biodiversity baselines: tracking insects in Kruger National Park with DNA barcodes. *Biological Conservation*. 2021; 256: 109034.

16. Sato H, Sogo Y, Doi H, Yamanaka H. Usefulness and limitations of sample pooling for environmental DNA metabarcoding of freshwater fish communities. *Scientific Reports*. 2017; 7: 14860.

17. Bell KL. Applying pollen DNA metabarcoding to the study of plant-pollinator interactions. *Applications in Plant Sciences*. 2017; 5: apps.1600124

18. Vasselon V, Bouchez A, Rimet F, Jacquet S, Trobajo R, Corniquel M, Tapolczai K, Domaizon I. Avoiding quantification bias in metabarcoding: Application of a cell biovolume correction factor in diatom molecular biomonitoring (A. Mahon, Ed.). *Methods in Ecology and Evolution*. 2018; 9: 1060–1069.

19. Bellemain E, Davey ML, Kauserud H, Epp LS, Boessenkool S, Coissac E, Geml J, Edwards M, Willerslev E, Gussarova G, Taberlet P, Haile J, Brochmann C. Fungal

palaeodiversity revealed using high-throughput metabarcoding of ancient DNA from arctic permafrost. *Environmental Microbiology*. 2012; 15: 1176–1189.

20. Aas AB, Davey ML, Kauserud H. ITS all right mama: investigating the formation of chimeric sequences in the ITS2 region by DNA metabarcoding analyses of fungal mock communities of different complexities. *Molecular Ecology Resources*. 2017; 17: 730–741.

21. Tedersoo L, Tooming-Klunderud A, Anslan S. PacBio metabarcoding of Fungi and other eukaryotes: errors, biases, and perspectives. New Phytologist. 2018; 217: 1370–1385.

22. Malaise R. A new insect trap. *Entomologisk Tidskrift*. 1937; 58: 148–160.

23. Karlsson D, Pape T, Johanson KA, Liljeblad J, Ronquist F. The Swedish Malaise Trap Project, or how many species of Hymenoptera and Diptera are there in Sweden? *Entomologisk Tidskrift*. 2005; 126**:** 43–53.

24. deWaard JR, Levesque-Beaudin V, deWaard SL, Ivanova NV, McKeown JTA, Miskie R, Naik S, Perez KHJ, Ratnasingham S, Sobel CN, Sones JE, Steinke C, Telfer AC, Young A, Young MR, Zakharov EV, Hebert PDN. Expedited assessment of terrestrial arthropod diversity by coupling Malaise traps with DNA barcoding. *Genome*. 2019; 62: 85–95.

25. Steinke D, Braukmann TWA, Manerus L, Woodhouse A, Elbrecht V. Effects of Malaise trap spacing on species richness and composition of terrestrial arthropod bulk samples. *Metabarcoding and Metagenomics*. 2021; 5: 43–50.

26. Holdridge LR. Determination of world plant formations from simple climatic data. *Science*. 1947; 105: 367–368.

27. Whittaker RH. Classification of natural communities. *Botanical Reviews*. 1962; 28: 1–239.

28. Olson DM, Dinerstein E, Wikramanayake ED, Burgess ND, Powell GVN, Underwood EC, D'amico JA, Itoua I, Strand HE, Morrison JC, Loucks CJ, Allnutt TF, Ricketts TH, Kura Y, Lamoreux JF, Wettengel WW, Hedao P, Kassem KR. Terrestrial ecoregions of the world: a new map of life on earth. *Bioscience*. 2001; 51: 933–938.

29. Bailey RG. *Ecoregions*. Springer, New York; 2014.

30. Giakoumi S, Sini M, Gerovasileiou V, Mazor T, Beher J, Possingham HP, Abdulla A, Cinar ME, Dendrinos P, Gucu AC, Karamanlidis AA, Rodic P, Panayotidis P, Taskin E, Jaklin A, Voultsiadou E, Webster C, Zenetos A, Katsanevakis S. Ecoregion-based

conservation planning in the Mediterranean: Dealing with large-scale heterogeneity. *PLoS ONE*. 2013; 8(10): e76449.

31. Dinerstein E, Olson D, Joshi A, Vynne C, Burgess ND, Wikramanayake E, Hahn N, Palminteri S, Hedao P, Noss R, Hansen M, Locke H, Ellis EC, Jones B, Barber CV, Hayes R, Kormos C, Martin V, Crist E, Sechrest W, Price L, Baillie JEM, Weeden D, Suckling K, Davis C, Sizer N, Moore R, Thau D, Birch T, Potapov P, Turubanova S, Tyukavina A, de Souza N, Pintea L, Brito JC, Llewellyn OA, Miller AG, Patzelt A, Ghazanfarm SA, Timberlake J, Klöser H, Shennan-Farpón Y, Kindt R, Barnekow Lillesø J-P, van Breugel P, Graudal L, Voge M, Al-Shammari KF, Saleem M. An ecoregion-based approach to protecting half the terrestrial realm. *Bioscience*. 2013; 67: 534–545.

32. Crins WJ, Gray PA, Uhlig PWC, Wester MC. *The Ecosystems of Ontario, Part 1: Ecozones and Ecoregions*. Technical Report SIB TER IMA TR-01, Ministry of Natural Resources, Ontario; 2009.

33. Ivanova NV, deWaard JR, Hebert PDN. An inexpensive, automation-friendly protocol for recovering high-quality DNA. *Molecular Ecology Resources*. 2006; 6: 998–1002.

34. Ratnasingham S and PDN Hebert. A DNA-based registry for all animal species: The Barcode Index Number (BIN) System. *PLoS ONE*. 2013; 8: e66213.

35. Ratnasingham S and PDN Hebert. BOLD: The Barcode of Life Data System (www.barcodinglife.org). *Molecular Ecology Notes*. 2007; 7: 355–364.

36. Magurran AE. *Measuring Biological Diversity*. Wiley-Blackwell, Malden, Massachusetts; 2003.

37. Preston FW. The canonical distribution of commonness and rarity: Part I. *Ecology*. 1962; 43: 185–215.

38. Luke SH, Fayle TM, Eggleton P, Turner EC, Davies RG. Functional structure of ant and termite assemblages in old growth forest, logged forest and oil palm plantation in Malaysian Borneo. *Biodiversity Conservation*. 2014; 23: 2817–2832.

39. Newbold T, Hudson LN, Phillips HRP, Hill SLL, Contu S, Lysenko I, Blandon A, Butchart SHM, Booth HL, Day J, De Palma A, Harrison MLK, Kirkpatrick L, Pynegar E, Robinson A, Simpson J, Mace GM, Scharlemann JPW, Purvis A. A global model of the response of tropical and sub-tropical forest biodiversity to anthropogenic pressures. *Proceedings of the Royal Society B*. 2014; 281: 20141435.

40. Phalan B, Onial M, Balmford A, Green RE. Reconciling food production and biodiversity conservation: Land sharing and land sparing compared. *Science.* 2011; 333: 1289–1291.

41. Gray CL, Hill SLL, Newbold T, Hudson LN, Boerger L, Contu S, Hoskins AJ, Ferrier S, Purvis A, Scharlemann JPW. Local biodiversity is higher inside than outside terrestrial protected areas worldwide. *Nature Communications*. 2016; 7: 12306.

42. Lingbeek BJ, Higgins CL, Muir JP, Kattes DH, Schwertner TW. Arthropod diversity and assemblage structure response to deforestation and desertification in the Sahel of western Senegal. *Global Ecology and Conservation*. 2017; 11: 165–176.

43. Kirse A, Bourlat SJ, Langen K, Fonseca VG. Metabarcoding Malaise traps and soil eDNA reveals seasonal and local arthropod diversity shifts. Scientific Reports. 2021; 11: 10498.

44. Tscharntke T, Tylianakis JM, Rand TA, Didham RK, Fahring L, Batary P, Bengtsson J, Clough Y, Crist TO, Dormann CF, Ewers RM, Fruend J, Holt RD, Holzschuh A, Klein AM, Kleijn D, Kremen C, Landis DA, Laurance W, Lindenmayer D, Scherber C, Sodhi N, Steffan-Dewenter I, Thies C, van der Putten WM, Westphal C. Landscape moderation of biodiversity patterns and processes – eight hypotheses. *Biological Reviews*. 2012; 87: 661–685.

45. Myers JA, Chase JM, Jiminez I, Jorgensen PM, Araujo-Murakami A, Paniagua-Zambrana N, Seidel R. Beta-diversity in temperate and tropical forests reflects dissimilar mechanisms of community assembly. *Ecology Letters*. 2013; 16: 151–157.

46. Snell Taylor SJ, Evans BS, White EP, Hurlbert AH. The prevalence and impact of transient species in ecological communities. *Ecology*. 2018; 99(8): 1825–1835.

47. D'Souza ML, Hebert PDN. Stable baselines of temporal turnover underlie beta diversity in tropical arthropod communities. *Molecular Ecology*. 2018; 27: 2447–2460.

48. Smith JR, Letten AD, Ke P-J, Anderson CB, Hendershot JN, Dhami MK, Dlott GA, Grainger TN, Howard ME, Morrison BML, Routh D, San Juan PA, Mooney HA, Mordecai EA, Crowther TW, Daily GC. A global test of ecoregions. *Nature Ecology & Evolution*. 2018; 2: 1889–1896.

49. Lightfoot DC, Brantely SL, Allen CD. Geographic patterns of ground-dwelling arthropods across an ecological transition in the North American southwest. *Western North American Naturalist*. 2008; 68: 83–102.

50. Gonzales-Reyes AX, Corronca JA, Arroyo NC. Differences in alpha and beta diversities of epideous arthropod assemblages in two ecoregions of northwestern Argentina. *Zoological Studies*. 2012; 51: 1367–1379.

51. Watson JEM, Venter O. Ecology: a global plan for nature conservation. *Nature*. 2017; 550: 48–49.

52. Wilson EO. *Half-Earth: Our Planet's Fight for Life*, Liveright, New York; 2017.

53. Díaz S, Settele J, Brondízio ES, Ngo HT, Guèze M, Agard J, Arneth A, Balvanera P, Brauman KA, Butchart SHM, Chan KMA, Garibaldi LA, Ichii K, Liu J, Subramanian SM, Midgley GF, Miloslavich P, Molnár Z, Obura D, Pfaff A, Polasky S, Purvis A, Razzaque J, Reyers B, Chowdhury RR, Shin YJ, Visseren-Hamakers IJ, Willis KJ, Zayas CN (eds.). *Summary for policymakers of the global assessment report on biodiversity and ecosystem services of the Intergovernmental Science-Policy Platform on Biodiversity and Ecosystem Services*. IPBES secretariat, Bonn, Germany; 2019.

54. Hobern D. BIOSCAN: DNA barcoding to accelerate taxonomy and biogeography for conservation and sustainability. *Genome*. 2021; 64: 161–164.

55. Braukmann TWA, Prosser SJR, Ivanova NV, Elbrecht V, Steinke D, Ratnasingham R, deWaard JR, Sones JE, Zakharov EV, Hebert PDN. Metabarcoding a diverse arthropod mock community. *Molecular Ecology Resources*. 2019; 19: 711–727.

56. Prosser SWJ, deWaard JR, Miller SE, and PDN Hebert. DNA barcodes from century-old type specimens using next-generation sequencing. *Molecular Ecology Resources*. 2016; 16: 487–497.

57. Hebert PDN, Penton EH, Burns JM, Janzen DH, Hallwachs W. Ten species in one: DNA barcoding reveals cryptic species in the neotropical skipper butterfly *Astraptes fulgerator*. *Proceedings of the National Academy of Sciences of the United States of America*. 2004; 101: 14812–14817.

58. Elbrecht V, Steinke D. Scaling up DNA metabarcoding for freshwater macrozoobenthos monitoring. *Freshwater Biology*. 2018; 64: 380–387.

59. Oksanen J, Blanchet FG, Friendly M, Kindt R, Legendre P, McGlinn D, Minchin PR, O'Hara RB, Simpson GL, Solymos P, Stevens MHH, Szoecs E, Wagner H. *vegan: Community Ecology Package*. R package version 2.5-1. https://CRAN.R-project.org/package=vegan; 2018

60. Fisher RA, Corbet AS, Williams CB. The relation between the number of species and the number of individuals in a random sample of animal population. Journal of Animal Ecology. 1943; 12: 42–58.

61. Stamatakis A, Hoover P, Rougemont J. A rapid bootstrap algorithm for the RAxML web servers. Systematic Biology. 2008; 57(5): 758–771.

62. Miller MA, Pfeiffer W, Schwartz T. The CIPRES science gateway. In: *Proceedings of the 2011 TeraGrid Conference on Extreme Digital Discovery—TG '11*. New York, USA: ACM Press; 2011.

63. Faith DP. Conservation evaluation and phylogenetic diversity. *Biological Conservation*. 1992; 61: 1–10.

64. Kembel SW, Cowan PD, Helmus MR, Cornwell WK, Morlon H, Ackerly DD, Blomberg SP, Webb CO. Picante: R tools for integrating phylogenies and ecology. *Bioinformatics*. 2010; 26(11): 1463–1464.

65. Swenson NG. Phylogenetic resolution and quantifying the phylogenetic diversity and dispersion of communities. *PLoS ONE*. 2009; 4(2): e4390.

66. Dinno A. *dunn.test: Dunn's Test of Multiple Comparisons Using Rank Sums. R package version 1.3.2*. http://CRAN.R-project.org/package= dunn.test; 2016.

67. Baselga A, Orme CDL. betapart: an R package for the study of beta diversity. *Methods Ecology and Evolution*. 2012; 3: 808–812.

68. R Core Team. *R: A language and environment for statistical computing*. R Foundation for Statistical Computing, Vienna, Austria; 2018. URL https://www.R-project.org/.

Figure legends

Figure 1: Map of sampling locations and ecoregion boundaries in Southern Ontario, Canada.

Figure 2: a) BIN accumulation curve for the 410 Malaise trap samples collected in 51 Ontario Provincial Parks. b) Fisher's log series fit to the number of sites where each BIN was observed. c) Preston's lognormal species abundance curve showing the total BINs within each log_2 abundance interval.

Figure 3: BIN compositional differences among three Ontario ecoregions: a) Relationship between geographical distance and mean community similarity (Sørensen's similarity coefficient) within and between ecoregions. b) Boxplots comparing Faith's Phylogenetic Diversity for the three ecoregions. Significant differences between pairs are indicated with different lowercase characters (a - ECF/EGL, b - ECF/SGL, c - EGL/SGL). c) Venn diagram depicting BIN overlap among ecoregions. d) Non-metric multidimensional scaling (NMDS) plot using Bray-Curtis index coefficient. Colour coding is based on ecoregion.

Figure 4: Comparison of α-diversity (± s.e.) in three Ontario ecoregions for all BINs and for ten arthropod taxa using 12 random sites from the total sites for each ecoregion. Statistical tests are based on Kruskal–Wallis followed by Mann–Whitney post-hoc comparisons with Bonferroni correction. Significant differences between pairs are indicated with different lowercase characters (a - ECF/EGL, b - ECF/SGL, c - EGL/SGL).

Figure 5: Total β-diversity (solid lines) and turnover (dotted lines) for three Ontario ecoregions. Values were computed using 1000 bootstrap samples of 12 random sites from each ecoregion. Significant differences between ecoregions are detected when the peaks of the density plots do not overlap.

Figure S1: Relationship between filtered read count and number of BINs for 410 metabarcoded samples from three ecoregions.

Figure S2: Bar plot showing α-diversity per month for all 52 sites

Figure S3: Patterns of α-diversity and read abundance per major arthropod group and site

673

 Click here to access/download;Table;Table S1.docx

Table S1: mBRAVE project codes as well as samples analyzed and read coverage for each 530 chip analyzed on the Ion Torrent S5. The 11 chips that included samples from two sites are highlighted in yellow.

Project Code	Project Title	Reads	Filtered Reads
MBR-OPPMAA	Ontario Provincial Parks 2014:CCDB-S5-0053_CBGMB-00003_Algonquin PP - Rock Lake Site 1	9376304	2592428
MBR-OPPMABMAC	Ontario Provincial Parks 2014:CCDB-S5-0066_CBGMB-00016_Algonquin PP - Oxtongue Site 3 + Awenda PP	8544988	2674401
MBR-OPPMADMAE	Ontario Provincial Parks 2014:CCDB-S5-0067_CBGMB-00017_Balsam Lake PP + Bayview Escarpment PP	8535733	3294349
MBR-OPPMAFMAI	Ontario Provincial Parks 2014:CCDB-S5-0068_CBGMB-00018_Bell Bay PP + Boyne Valley PP	6430734	1847104
MBR-OPPMAHMBD	Ontario Provincial Parks 2014:CCDB-S5-0086_CBGMB-00035_Bon Echo PP - Site 1 + Morris Tract PP	7271497	2424720
MBR-OPPMAJ	Ontario Provincial Parks 2014:CCDB-S5-0057_CBGMB-00007_Bronte Creek PP	6892296	2517868
MBR-OPPMAKMAO	Ontario Provincial Parks 2014:CCDB-S5-0073_CBGMB-00019_Charleston Lake PP + Ferris PP	10020289	3480857
MBR-OPPMAL	Ontario Provincial Parks 2014:CCDB-S5-0058_CBGMB-00008_Duncan Escarpment PP	9293842	3435885
MBR-OPPMAN	Ontario Provincial Parks 2014:CCDB-S5-0061_CBGMB-00009_Emily PP	8315694	3169017
MBR-OPPMAP	Ontario Provincial Parks 2014:CCDB-S5-0063_CBGMB-00010_Forks of the Credit PP	10348276	4090743
MBR-OPPMAQMAU	Ontario Provincial Parks 2014:CCDB-S5-0069_CBGMB-00020_Frontenac PP + Inverhuron PP	7931246	2871585
MBR-OPPMAR	Ontario Provincial Parks 2014:CCDB-S5-0064_CBGMB-00012_Holland Landing Prairie PP	8124964	2701838
MBR-OPPMAS	Ontario Provincial Parks 2014:CCDB-S5-0054_CBGMB-00004_Hope Bay Forest PP	9227593	3463105
MBR-OPPMAT	Ontario Provincial Parks 2014:CCDB-S5-0065_CBGMB-00013_Indian Point PP - Site 1	9151934	3599629
MBR-OPPMAWMAZ	Ontario Provincial Parks 2014:CCDB-S5-0070_CBGMB-00021_John E Pearce PP + Lions Head PP	10693129	3641703
MBR-OPPMAX	Ontario Provincial Parks 2014:CCDB-S5-0080_CBGMB-00026_Johnston Harbour PP	7927583	2339035
MBR-OPPMAY	Ontario Provincial Parks 2014:CCDB-S5-0081_CBGMB-00027_Lake St Peter PP	9380178	3266285
MBR-OPPMBAMBE	Ontario Provincial Parks 2014:CCDB-S5-0072_CBGMB-00022_Lower Madawaska River PP + Murphys Point PP	10315593	3815438
MBR-OPPMBB	Ontario Provincial Parks 2014:CCDB-S5-0082_CBGMB-00028_MacGregor Point PP	9944565	3854418
MBR-OPPMBC	Ontario Provincial Parks 2014:CCDB-S5-0056_CBGMB-00006_Mark S Burnham PP	10796469	4536699
MBR-OPPMBFMBH	Ontario Provincial Parks 2014:CCDB-S5-0074_CBGMB-00023_Ojibway Prairie PP + Pinery PP - Site 2	9804179	3243861
MBR-OPPMBG	Ontario Provincial Parks 2014:CCDB-S5-0083_CBGMB-00029_Petroglyphs PP	10645785	3989178
MBR-OPPMBI	Ontario Provincial Parks 2014:CCDB-S5-0084_CBGMB-00030_Port Burwell PP	10140496	3058298
MBR-OPPMBJ	Ontario Provincial Parks 2014:CCDB-S5-0085_CBGMB-00031_Presqu'ile PP	10651981	4259741
MBR-OPPMBMMBN	Ontario Provincial Parks 2014:CCDB-S5-0075_CBGMB-00024_Rondeau PP - Site 1 + Sandbanks PP	10725061	3868406

MBR-OPPMBO	Ontario Provincial Parks 2014:CCDB-S5-0087_CBGMB-00036_Selkirk PP	9153371	2649363
MBR-OPPMBP	Ontario Provincial Parks 2014:CCDB-S5-0055_CBGMB-00005_Sharbot Lake PP	8344274	3368635
MBR-OPPMBQMBU	Ontario Provincial Parks 2014:CCDB-S5-0079_CBGMB-00025_Short Hills PP + Turkey Point PP	7941400	2702280
MBR-OPPMBR	Ontario Provincial Parks 2014:CCDB-S5-0088_CBGMB-00037_Sibbald Point PP	8752087	3151909
MBR-OPPMBS	Ontario Provincial Parks 2014:CCDB-S5-0089_CBGMB-00038_Silent Lake PP	8381273	2870078
MBR-OPPMBT	Ontario Provincial Parks 2014:CCDB-S5-0090_CBGMB-00039_Silver Lake PP	8105235	2674401
MBR-OPPMBX	Ontario Provincial Parks 2014:CCDB-S5-0097_CBGMB-00040_Wheatley PP	9036296	2592428
MBR-OPPMBZZ	Ontario Provincial Parks 2014:CCDB-S5-0052_CBGMB-00002_Peter's Woods PP Malaise	8048432	3302341
MBR-OPPMBY	Ontario Provincial Parks 2014:CCDB-S5-041_CBGMB-00001_Long Point PP Malaise	11177387	4232828
MBR-OPPMAG	Ontario Provincial Parks 2014:CCDB-S5-0127_CBGMB-00068_Black Creek PP	7877916	2491752
MBR-OPPMAM	Ontario Provincial Parks 2014:CCDB-S5-0128_CBGMB-00069_Earl Rowe PP	8955778	3043981
MBR-OPPMAV	Ontario Provincial Parks 2014:CCDB-S5-0129_CBGMB-00070_James N Allan PP	6594231	1605985
MBR-OPPMBK	Ontario Provincial Parks 2014:CCDB-S5-0130_CBGMB-00071_Pretty River Valley PP	7497170	2158049
MBR-OPPMBL	Ontario Provincial Parks 2014:CCDB-S5-0131_CBGMB-00072_Rock Point PP	8479191	1624386
MBR-OPPMBV	Ontario Provincial Parks 2014:CCDB-S5-0132_CBGMB-00073_Upper Madawaska River PP	9938090	3074949
MBR-OPPMBW	Ontario Provincial Parks 2014:CCDB-S5-0133_CBGMB-00074_Wasaga Beach PP	9050667	2673304

Figure Click here to access/download;Table;Table S2.docx

Table S2: GPS coordinates, elevation (m), and ecoregion assignment for the 52 sampling sites and the number of BINs recovered from each site. ECF = Eastern Canadian Forest (15 sites); EGL = Eastern Great Lakes Forests (24 sites); SGL = Southern Great Lakes Forests (13 sites).

Provincial Park	Latitude	Longitude	Elevation(m)	BINs	Ecoregion
Algonquin - Oxtongue River	45.4621	-78.796	427	1642	ECF
Algonquin - Rock Lake	45.51962	-78.39752	353	2030	ECF
Bell Bay	45.51506	-77.81812	320	3186	ECF
Bon Echo	44.89405	-77.19691	272	2638	ECF
Charleston Lake	44.49798	-76.0414	88	3021	ECF
Frontenac	44.51783	-76.53944	166	3498	ECF
Indian Point	44.60414	-78.82895	258	2403	ECF
Lake St Peter	45.3202	-78.02496	406	2664	ECF
Lower Madawaska River	45.25606	-77.19221	262	3333	ECF
Murphys Point	44.78118	-76.2336	132	4581	ECF
Petroglyphs	44.61605	-78.04084	265	3194	ECF
Sharbot Lake	44.77952	-76.72379	201	2878	ECF
Silent Lake	44.92144	-78.06931	360	2895	ECF
Silver Lake	44.83129	-76.57565	184	4508	ECF
Upper Madawaska River	45.53535	-78.04699	318	3796	ECF
Awenda	44.82534	-79.98458	224	3135	EGL
Balsam Lake	44.62857	-78.8614	274	3227	EGL
Bayview Escarpment	44.63367	-80.69829	329	3966	EGL
Black Creek	44.96797	-81.36156	179	2369	EGL
Boyne Valley	44.11563	-80.12777	460	1609	EGL
Duncan Escarpment	44.42305	-80.46923	395	1957	EGL
Earl Rowe	44.15176	-79.903	216	1479	EGL
Emily	44.34143	-78.53746	251	1916	EGL
Ferris	44.28286	-77.79627	132	4005	EGL
Forks of the Credit	43.82415	-80.00309	403	2753	EGL
Holland Landing Prairie	44.11894	-79.48795	225	2545	EGL
Hope Bay Forest	44.92509	-81.15563	253	1071	EGL
Inverhuron	44.29838	-81.59065	182	1785	EGL
Johnston Harbour-Pine Tree Point	45.1171	-81.53679	177	1939	EGL
Lions Head	44.99539	-81.2334	219	2074	EGL
MacGregor Point	44.41072	-81.44641	191	2607	EGL
Mark S Burnham	44.29882	-78.26779	209	1633	EGL
Morris Tract	43.72995	-81.64166	257	1673	EGL
Peters Woods	44.12845	-78.04057	236	4197	EGL
Presqu'ile	44.00914	-77.7424	77	3197	EGL
Pretty River Valley	44.41232	-80.30035	337	3550	EGL
Sandbanks	43.90287	-77.26929	85	1312	EGL

Sibbald Point	44.32982	-79.32737	221	1872	EGL
Wasaga Beach	44.51258	-80.01165	188	1314	EGL
Bronte Creek	43.4023	-79.7617	125	2800	SGL
James N Allan	42.84962	-79.66397	175	2029	SGL
John E Pearce	42.60595	-81.44243	195	1339	SGL
Long Point	42.58006	-80.38538	175	1363	SCF
Ojibway Prairie	42.26278	-83.07246	180	3311	SGL
Pinery	43.26987	-81.82706	182	1978	SGL
Port Burwell	42.6544	-80.81493	196	996	SGL
Rock Point	42.85405	-79.55536	176	2246	SGL
Rondeau	42.30206	-81.85306	175	1356	SGL
Selkirk	42.81676	-79.95736	180	1146	SGL
Short Hills	43.11288	-79.27376	95	2541	SGL
Turkey Point	42.70515	-80.32849	222	2513	SGL
Wheatley	42.09199	-82.44227	181	1210	SGL

 Click here to access/download;Table;Table S3.xlsx

Table S3: Sampling dates, pre- and post-filtering read counts, BIN and OTU co

Provincial Park	Start Date	End Date	Project Name
Algonquin - Oxtongue	21-May-14	3-Jun-14	MBR-OPPMABMAC
Algonquin - Oxtongue	17-Jun-14	1-Jul-14	MBR-OPPMABMAC
Algonquin - Oxtongue	15-Jul-14	29-Jul-14	MBR-OPPMABMAC
Algonquin - Oxtongue	12-Aug-14	26-Aug-14	MBR-OPPMABMAC
Algonquin - Oxtongue	9-Sep-14	23-Sep-14	MBR-OPPMABMAC
Algonquin - Rock Lake	6-May-14	21-May-14	MBR-OPPMAA
Algonquin - Rock Lake	21-May-14	3-Jun-14	MBR-OPPMAA
Algonquin - Rock Lake	3-Jun-14	17-Jun-14	MBR-OPPMAA
Algonquin - Rock Lake	17-Jun-14	1-Jul-14	MBR-OPPMAA
Algonquin - Rock Lake	15-Jul-14	29-Jul-14	MBR-OPPMAA
Algonquin - Rock Lake	29-Jul-14	12-Aug-14	MBR-OPPMAA
Algonquin - Rock Lake	12-Aug-14	26-Aug-14	MBR-OPPMAA
Algonquin - Rock Lake	26-Aug-14	9-Sep-14	MBR-OPPMAA
Algonquin - Rock Lake	9-Sep-14	23-Sep-14	MBR-OPPMAA
Algonquin - Rock Lake	1-Jul-2014	15-Jul-2014	MBR-OPPMAA
Bell Bay	22-May-14	4-Jun-14	MBR-OPPMAFMAI
Bell Bay	18-Jun-2014	2-Jul-2014	MBR-OPPMAFMAI
Bell Bay	16-Jul-14	30-Jul-14	MBR-OPPMAFMAI
Bell Bay	13-Aug-14	27-Aug-14	MBR-OPPMAFMAI
Bell Bay	10-Sep-14	24-Sep-14	MBR-OPPMAFMAI
Bon Echo	22-May-14	4-Jun-14	MBR-OPPMAHMBD
Bon Echo	18-Jun-14	2-Jul-14	MBR-OPPMAHMBD
Bon Echo	16-Jul-14	30-Jul-14	MBR-OPPMAHMBD
Bon Echo	13-Aug-14	27-Aug-14	MBR-OPPMAHMBD
Bon Echo	10-Sep-14	24-Sep-14	MBR-OPPMAHMBD
Charleston Lake	23-May-14	5-Jun-14	MBR-OPPMAKMAO
Charleston Lake	19-Jun-14	3-Jul-14	MBR-OPPMAKMAO
Charleston Lake	17-Jul-14	31-Jul-14	MBR-OPPMAKMAO
Charleston Lake	14-Aug-14	28-Aug-14	MBR-OPPMAKMAO
Charleston Lake	11-Sep-14	25-Sep-14	MBR-OPPMAKMAO
Frontenac	23-May-14	5-Jun-14	MBR-OPPMAQMAU
Frontenac	19-Jun-14	3-Jul-14	MBR-OPPMAQMAU
Frontenac	17-Jul-14	31-Jul-14	MBR-OPPMAQMAU
Frontenac	14-Aug-14	28-Aug-14	MBR-OPPMAQMAU
Frontenac	11-Sep-14	25-Sep-14	MBR-OPPMAQMAU
Indian Point	30-Apr-14	19-May-14	MBR-OPPMAT
Indian Point	19-May-14	2-Jun-14	MBR-OPPMAT
Indian Point	2-Jun-14	16-Jun-14	MBR-OPPMAT
Indian Point	16-Jun-14	30-Jun-14	MBR-OPPMAT
Indian Point	30-Jun-14	14-Jul-14	MBR-OPPMAT
Indian Point	14-Jul-14	28-Jul-14	MBR-OPPMAT
Indian Point	28-Jul-14	11-Aug-14	MBR-OPPMAT

Indian Point	11-Aug-14	25-Aug-14	MBR-OPPMAT
Indian Point	25-Aug-14	8-Sep-14	MBR-OPPMAT
Indian Point	8-Sep-14	22-Sep-14	MBR-OPPMAT
Lake St Peter	6-May-14	21-May-14	MBR-OPPMAY
Lake St Peter	21-May-14	3-Jun-14	MBR-OPPMAY
Lake St Peter	3-Jun-14	17-Jun-14	MBR-OPPMAY
Lake St Peter	17-Jun-14	1-Jul-14	MBR-OPPMAY
Lake St Peter	1-Jul-14	15-Jul-14	MBR-OPPMAY
Lake St Peter	15-Jul-14	29-Jul-14	MBR-OPPMAY
Lake St Peter	29-Jul-14	12-Aug-14	MBR-OPPMAY
Lake St Peter	12-Aug-14	26-Aug-14	MBR-OPPMAY
Lake St Peter	26-Aug-14	9-Sep-14	MBR-OPPMAY
Lake St Peter	9-Sep-14	23-Sep-14	MBR-OPPMAY
Lower Madawaska River	22-May-14	4-Jun-14	MBR-OPPMBAMBE
Lower Madawaska River	18-Jun-2014	2-Jul-201	MBR-OPPMBAMBE
Lower Madawaska River	16-Jul-14	30-Jul-14	MBR-OPPMBAMBE
Lower Madawaska River	13-Aug-14	27-Aug-14	MBR-OPPMBAMBE
Lower Madawaska River	10-Sep-14	24-Sep-14	MBR-OPPMBAMBE
Murphys Point	23-May-14	5-Jun-14	MBR-OPPMBAMBE
Murphys Point	19-Jun-14	3-Jul-14	MBR-OPPMBAMBE
Murphys Point	17-Jul-14	31-Jul-14	MBR-OPPMBAMBE
Murphys Point	14-Aug-14	28-Aug-14	MBR-OPPMBAMBE
Murphys Point	11-Sep-14	25-Sep-14	MBR-OPPMBAMBE
Petroglyphs	30-Apr-14	19-May-14	MBR-OPPMBG
Petroglyphs	19-May-14	2-Jun-14	MBR-OPPMBG
Petroglyphs	2-Jun-14	16-Jun-14	MBR-OPPMBG
Petroglyphs	16-Jun-14	30-Jun-14	MBR-OPPMBG
Petroglyphs	30-Jun-14	14-Jul-14	MBR-OPPMBG
Petroglyphs	14-Jul-14	28-Jul-14	MBR-OPPMBG
Petroglyphs	28-Jul-14	11-Aug-14	MBR-OPPMBG
Petroglyphs	11-Aug-14	25-Aug-14	MBR-OPPMBG
Petroglyphs	25-Aug-14	8-Sep-14	MBR-OPPMBG
Petroglyphs	8-Sep-14	22-Sep-14	MBR-OPPMBG
Sharbot Lake	8-May-14	22-May-14	MBR-OPPMBP
Sharbot Lake	22-May-14	4-Jun-14	MBR-OPPMBP
Sharbot Lake	4-Jun-14	18-Jun-14	MBR-OPPMBP
Sharbot Lake	18-Jun-14	2-Jul-14	MBR-OPPMBP
Sharbot Lake	2-Jul-14	16-Jul-14	MBR-OPPMBP
Sharbot Lake	16-Jul-14	30-Jul-14	MBR-OPPMBP
Sharbot Lake	30-Jul-14	13-Aug-14	MBR-OPPMBP
Sharbot Lake	13-Aug-14	27-Aug-14	MBR-OPPMBP
Sharbot Lake	27-Aug-14	10-Sep-14	MBR-OPPMBP
Sharbot Lake	10-Sep-14	24-Sep-14	MBR-OPPMBP
Silent Lake	29-Apr-14	21-May-14	MBR-OPPMBS
Silent Lake	21-May-14	2-Jun-14	MBR-OPPMBS
Silent Lake	2-Jun-14	16-Jun-14	MBR-OPPMBS
Silent Lake	16-Jun-14	30-Jun-14	MBR-OPPMBS

Silent Lake	30-Jun-14	14-Jul-14	MBR-OPPMBS
Silent Lake	14-Jul-14	28-Jul-14	MBR-OPPMBS
Silent Lake	28-Jul-14	11-Aug-14	MBR-OPPMBS
Silent Lake	11-Aug-14	25-Aug-14	MBR-OPPMBS
Silent Lake	25-Aug-14	8-Sep-14	MBR-OPPMBS
Silent Lake	8-Sep-14	23-Sep-14	MBR-OPPMBS
Silver Lake	8-May-14	22-May-14	MBR-OPPMBT
Silver Lake	22-May-14	5-Jun-14	MBR-OPPMBT
Silver Lake	5-Jun-14	19-Jun-14	MBR-OPPMBT
Silver Lake	19-Jun-14	3-Jul-14	MBR-OPPMBT
Silver Lake	3-Jul-14	17-Jul-14	MBR-OPPMBT
Silver Lake	17-Jul-14	31-Jul-14	MBR-OPPMBT
Silver Lake	31-Jul-14	14-Aug-14	MBR-OPPMBT
Silver Lake	14-Aug-14	28-Aug-14	MBR-OPPMBT
Silver Lake	28-Aug-14	11-Sep-14	MBR-OPPMBT
Silver Lake	11-Sep-14	25-Sep-14	MBR-OPPMBT
Upper Madawaska River	7-May-14	22-May-14	MBR-OPPMBV
Upper Madawaska River	22-May-14	4-Jun-14	MBR-OPPMBV
Upper Madawaska River	4-Jun-14	18-Jun-14	MBR-OPPMBV
Upper Madawaska River	18-Jun-14	2-Jul-14	MBR-OPPMBV
Upper Madawaska River	2-Jul-14	16-Jul-14	MBR-OPPMBV
Upper Madawaska River	16-Jul-14	30-Jul-14	MBR-OPPMBV
Upper Madawaska River	30-Jul-14	13-Aug-14	MBR-OPPMBV
Upper Madawaska River	13-Aug-14	27-Aug-14	MBR-OPPMBV
Upper Madawaska River	27-Aug-14	10-Sep-14	MBR-OPPMBV
Upper Madawaska River	10-Sep-14	24-Sep-14	MBR-OPPMBV
Awenda	16-May-14	30-May-14	MBR-OPPMABMAC
Awenda	13-Jun-14	27-Jun-14	MBR-OPPMABMAC
Awenda	11-Jul-14	25-Jul-14	MBR-OPPMABMAC
Awenda	8-Aug-14	22-Aug-14	MBR-OPPMABMAC
Awenda	5-Sep-14	19-Sep-14	MBR-OPPMABMAC
Balsam Lake	19-May-14	2-Jun-14	MBR-OPPMADMAE
Balsam Lake	16-Jun-14	30-Jun-14	MBR-OPPMADMAE
Balsam Lake	14-Jul-14	28-Jul-14	MBR-OPPMADMAE
Balsam Lake	11-Aug-14	25-Aug-14	MBR-OPPMADMAE
Balsam Lake	8-Sep-14	22-Sep-14	MBR-OPPMADMAE
Bayview Escarpment	15-May-14	29-May-14	MBR-OPPMADMAE
Bayview Escarpment	12-Jun-14	26-Jun-14	MBR-OPPMADMAE
Bayview Escarpment	10-Jul-14	24-Jul-14	MBR-OPPMADMAE
Bayview Escarpment	7-Aug-14	21-Aug-14	MBR-OPPMADMAE
Bayview Escarpment	4-Sep-14	18-Sep-14	MBR-OPPMADMAE
Black Creek	1-May-14	15-May-14	MBR-OPPMAG
Black Creek	15-May-14	29-May-14	MBR-OPPMAG
Black Creek	29-May-14	12-Jun-14	MBR-OPPMAG
Black Creek	12-Jun-14	26-Jun-14	MBR-OPPMAG
Black Creek	26-Jun-14	10-Jul-14	MBR-OPPMAG
Black Creek	10-Jul-14	24-Jul-14	MBR-OPPMAG

Black Creek	24-Jul-14	7-Aug-14	MBR-OPPMAG
Black Creek	7-Aug-14	21-Aug-14	MBR-OPPMAG
Black Creek	21-Aug-14	4-Sep-14	MBR-OPPMAG
Black Creek	4-Sep-14	18-Sep-14	MBR-OPPMAG
Boyne Valley	16-May-14	30-May-14	MBR-OPPMAFMAI
Boyne Valley	13-Jun-14	27-Jun-14	MBR-OPPMAFMAI
Boyne Valley	11-Jul-14	25-Jul-14	MBR-OPPMAFMAI
Boyne Valley	8-Aug-14	22-Aug-14	MBR-OPPMAFMAI
Boyne Valley	5-Sep-14	19-Sep-14	MBR-OPPMAFMAI
Duncan Escarpment	28-Apr-14	15-May-14	MBR-OPPMAL
Duncan Escarpment	15-May-14	29-May-14	MBR-OPPMAL
Duncan Escarpment	29-May-14	12-Jun-14	MBR-OPPMAL
Duncan Escarpment	12-Jun-14	26-Jun-14	MBR-OPPMAL
Duncan Escarpment	26-Jun-14	10-Jul-14	MBR-OPPMAL
Duncan Escarpment	10-Jul-14	24-Jul-14	MBR-OPPMAL
Duncan Escarpment	24-Jul-14	7-Aug-14	MBR-OPPMAL
Duncan Escarpment	7-Aug-14	21-Aug-14	MBR-OPPMAL
Duncan Escarpment	21-Aug-14	4-Sep-14	MBR-OPPMAL
Duncan Escarpment	4-Sep-14	18-Sep-14	MBR-OPPMAL
Earl Rowe	28-Apr-14	16-May-14	MBR-OPPMAM
Earl Rowe	16-May-14	30-May-14	MBR-OPPMAM
Earl Rowe	30-May-14	13-Jun-14	MBR-OPPMAM
Earl Rowe	13-Jun-14	27-Jun-14	MBR-OPPMAM
Earl Rowe	27-Jun-14	11-Jul-14	MBR-OPPMAM
Earl Rowe	11-Jul-14	25-Jul-14	MBR-OPPMAM
Earl Rowe	25-Jul-14	8-Aug-14	MBR-OPPMAM
Earl Rowe	8-Aug-14	22-Aug-14	MBR-OPPMAM
Earl Rowe	22-Aug-14	5-Sep-14	MBR-OPPMAM
Earl Rowe	5-Sep-14	19-Sep-14	MBR-OPPMAM
Emily	5-May-14	23-May-14	MBR-OPPMAN
Emily	23-May-14	6-Jun-14	MBR-OPPMAN
Emily	6-Jun-14	20-Jun-14	MBR-OPPMAN
Emily	20-Jun-14	4-Jul-14	MBR-OPPMAN
Emily	4-Jul-14	18-Jul-14	MBR-OPPMAN
Emily	18-Jul-14	1-Aug-14	MBR-OPPMAN
Emily	1-Aug-14	15-Aug-14	MBR-OPPMAN
Emily	15-Aug-14	29-Aug-14	MBR-OPPMAN
Emily	29-Aug-14	12-Sep-14	MBR-OPPMAN
Emily	12-Sep-14	26-Sep-14	MBR-OPPMAN
Ferris	23-May-14	5-Jun-14	MBR-OPPMAKMAO
Ferris	19-Jun-14	3-Jul-14	MBR-OPPMAKMAO
Ferris	17-Jul-14	31-Jul-14	MBR-OPPMAKMAO
Ferris	14-Aug-14	28-Aug-14	MBR-OPPMAKMAO
Ferris	12-Sep-14	26-Sep-14	MBR-OPPMAKMAO
Forks of the Credit	28-Apr-14	16-May-14	MBR-OPPMAP
Forks of the Credit	16-May-14	30-May-14	MBR-OPPMAP
Forks of the Credit	30-May-14	13-Jun-14	MBR-OPPMAP

Forks of the Credit	13-Jun-14	27-Jun-14	MBR-OPPMAP
Forks of the Credit	27-Jun-14	11-Jul-14	MBR-OPPMAP
Forks of the Credit	11-Jul-14	25-Jul-14	MBR-OPPMAP
Forks of the Credit	25-Jul-14	8-Aug-14	MBR-OPPMAP
Forks of the Credit	8-Aug-14	22-Aug-14	MBR-OPPMAP
Forks of the Credit	22-Aug-14	5-Sep-14	MBR-OPPMAP
Forks of the Credit	5-Sep-14	19-Sep-14	MBR-OPPMAP
Holland Landing Prairie	1-May-14	19-May-14	MBR-OPPMAR
Holland Landing Prairie	19-May-14	2-Jun-14	MBR-OPPMAR
Holland Landing Prairie	2-Jun-14	16-Jun-14	MBR-OPPMAR
Holland Landing Prairie	16-Jun-14	30-Jun-14	MBR-OPPMAR
Holland Landing Prairie	30-Jun-14	14-Jul-14	MBR-OPPMAR
Holland Landing Prairie	14-Jul-14	28-Jul-14	MBR-OPPMAR
Holland Landing Prairie	28-Jul-14	11-Aug-14	MBR-OPPMAR
Holland Landing Prairie	11-Aug-14	25-Aug-14	MBR-OPPMAR
Holland Landing Prairie	25-Aug-14	8-Sep-14	MBR-OPPMAR
Holland Landing Prairie	8-Sep-14	22-Sep-14	MBR-OPPMAR
Hope Bay Forest	2-May-14	15-May-14	MBR-OPPMAS
Hope Bay Forest	15-May-14	29-May-14	MBR-OPPMAS
Hope Bay Forest	29-May-14	12-Jun-14	MBR-OPPMAS
Hope Bay Forest	12-Jun-14	26-Jun-14	MBR-OPPMAS
Hope Bay Forest	26-Jun-14	10-Jul-14	MBR-OPPMAS
Hope Bay Forest	10-Jul-14	24-Jul-14	MBR-OPPMAS
Hope Bay Forest	24-Jul-14	7-Aug-14	MBR-OPPMAS
Hope Bay Forest	7-Aug-14	21-Aug-14	MBR-OPPMAS
Hope Bay Forest	21-Aug-14	4-Sep-14	MBR-OPPMAS
Hope Bay Forest	4-Sep-14	18-Sep-14	MBR-OPPMAS
Inverhuron	14-May-14	28-May-14	MBR-OPPMAQMAU
Inverhuron	11-Jun-14	25-Jun-14	MBR-OPPMAQMAU
Inverhuron	9-Jul-14	23-Jul-14	MBR-OPPMAQMAU
Inverhuron	6-Aug-14	20-Aug-14	MBR-OPPMAQMAU
Inverhuron	3-Sep-14	17-Sep-14	MBR-OPPMAQMAU
Johnston Harbour - Pine Tree Poi	1-May-14	15-May-14	MBR-OPPMAX
Johnston Harbour - Pine Tree Poi	15-May-14	29-May-14	MBR-OPPMAX
Johnston Harbour - Pine Tree Poi	29-May-14	12-Jun-14	MBR-OPPMAX
Johnston Harbour - Pine Tree Poi	12-Jun-14	26-Jun-14	MBR-OPPMAX
Johnston Harbour - Pine Tree Poi	26-Jun-14	10-Jul-14	MBR-OPPMAX
Johnston Harbour - Pine Tree Poi	10-Jul-14	24-Jul-14	MBR-OPPMAX
Johnston Harbour - Pine Tree Poi	24-Jul-14	7-Aug-14	MBR-OPPMAX
Johnston Harbour - Pine Tree Poi	7-Aug-14	21-Aug-14	MBR-OPPMAX
Johnston Harbour - Pine Tree Poi	21-Aug-14	4-Sep-14	MBR-OPPMAX
Johnston Harbour - Pine Tree Poi	4-Sep-14	18-Sep-14	MBR-OPPMAX
Lions Head	15-May-14	29-May-14	MBR-OPPMAWMAZ
Lions Head	12-Jun-14	26-Jun-14	MBR-OPPMAWMAZ
Lions Head	10-Jul-14	24-Jul-14	MBR-OPPMAWMAZ
Lions Head	7-Aug-14	21-Aug-14	MBR-OPPMAWMAZ
Lions Head	4-Sep-14	18-Sep-14	MBR-OPPMAWMAZ

MacGregor Point	1-May-14	14-May-14	MBR-OPPMBB
MacGregor Point	14-May-14	28-May-14	MBR-OPPMBB
MacGregor Point	28-May-14	11-Jun-14	MBR-OPPMBB
MacGregor Point	11-Jun-14	25-Jun-14	MBR-OPPMBB
MacGregor Point	25-Jun-14	9-Jul-14	MBR-OPPMBB
MacGregor Point	9-Jul-14	23-Jul-14	MBR-OPPMBB
MacGregor Point	23-Jul-14	6-Aug-14	MBR-OPPMBB
MacGregor Point	6-Aug-14	20-Aug-14	MBR-OPPMBB
MacGregor Point	20-Aug-14	3-Sep-14	MBR-OPPMBB
MacGregor Point	3-Sep-14	17-Sep-14	MBR-OPPMBB
Mark S Burnham	5-May-14	23-May-14	MBR-OPPMBC
Mark S Burnham	23-May-14	6-Jun-14	MBR-OPPMBC
Mark S Burnham	6-Jun-14	20-Jun-14	MBR-OPPMBC
Mark S Burnham	20-Jun-14	4-Jul-14	MBR-OPPMBC
Mark S Burnham	4-Jul-14	18-Jul-14	MBR-OPPMBC
Mark S Burnham	18-Jul-14	1-Aug-14	MBR-OPPMBC
Mark S Burnham	1-Aug-14	15-Aug-14	MBR-OPPMBC
Mark S Burnham	15-Aug-14	29-Aug-14	MBR-OPPMBC
Mark S Burnham	29-Aug-14	12-Sep-14	MBR-OPPMBC
Mark S Burnham	12-Sep-14	26-Sep-14	MBR-OPPMBC
Morris Tract	14-May-14	28-May-14	MBR-OPPMAHMBD
Morris Tract	11-Jun-14	25-Jun-14	MBR-OPPMAHMBD
Morris Tract	9-Jul-14	23-Jul-14	MBR-OPPMAHMBD
Morris Tract	6-Aug-14	20-Aug-14	MBR-OPPMAHMBD
Morris Tract	3-Sep-14	17-Sep-14	MBR-OPPMAHMBD
Peters Woods	20-Jun-14	4-Jul-14	MBR-OPPMBZZ
Peters Woods	5-May-14	23-May-14	MBR-OPPMBZZ
Peters Woods	23-May-14	6-Jun-14	MBR-OPPMBZZ
Peters Woods	6-Jun-14	20-Jun-14	MBR-OPPMBZZ
Peters Woods	4-Jul-14	18-Jul-14	MBR-OPPMBZZ
Peters Woods	18-Jul-14	1-Aug-14	MBR-OPPMBZZ
Peters Woods	1-Aug-14	15-Aug-14	MBR-OPPMBZZ
Peters Woods	15-Aug-14	29-Aug-14	MBR-OPPMBZZ
Peters Woods	29-Aug-14	12-Sep-14	MBR-OPPMBZZ
Peters Woods	12-Sep-14	26-Sep-14	MBR-OPPMBZZ
Presqu'ile	5-May-14	23-May-14	MBR-OPPMBJ
Presqu'ile	23-May-14	6-Jun-14	MBR-OPPMBJ
Presqu'ile	6-Jun-14	20-Jun-14	MBR-OPPMBJ
Presqu'ile	20-Jun-14	4-Jul-14	MBR-OPPMBJ
Presqu'ile	4-Jul-14	18-Jul-14	MBR-OPPMBJ
Presqu'ile	18-Jul-14	1-Aug-14	MBR-OPPMBJ
Presqu'ile	1-Aug-14	15-Aug-14	MBR-OPPMBJ
Presqu'ile	15-Aug-14	29-Aug-14	MBR-OPPMBJ
Presqu'ile	29-Aug-14	12-Sep-14	MBR-OPPMBJ
Presqu'ile	12-Sep-14	26-Sep-14	MBR-OPPMBJ
Pretty River Valley	28-Apr-14	15-May-14	MBR-OPPMBK
Pretty River Valley	15-May-14	29-May-14	MBR-OPPMBK

Pretty River Valley	29-May-14	12-Jun-14	MBR-OPPMBK
Pretty River Valley	12-Jun-14	26-Jun-14	MBR-OPPMBK
Pretty River Valley	26-Jun-14	10-Jul-14	MBR-OPPMBK
Pretty River Valley	10-Jul-14	24-Jul-14	MBR-OPPMBK
Pretty River Valley	24-Jul-14	7-Aug-14	MBR-OPPMBK
Pretty River Valley	7-Aug-14	21-Aug-14	MBR-OPPMBK
Pretty River Valley	21-Aug-14	4-Sep-14	MBR-OPPMBK
Pretty River Valley	4-Sep-14	18-Sep-14	MBR-OPPMBK
Sandbanks	23-May-14	5-Jun-14	MBR-OPPMBMMBN
Sandbanks	19-Jun-14	3-Jul-14	MBR-OPPMBMMBN
Sandbanks	17-Jul-14	31-Jul-14	MBR-OPPMBMMBN
Sandbanks	14-Aug-14	28-Aug-14	MBR-OPPMBMMBN
Sandbanks	11-Sep-14	25-Sep-14	MBR-OPPMBMMBN
Sibbald Point	1-May-14	19-May-14	MBR-OPPMBR
Sibbald Point	19-May-14	2-Jun-14	MBR-OPPMBR
Sibbald Point	2-Jun-14	16-Jun-14	MBR-OPPMBR
Sibbald Point	16-Jun-14	30-Jun-14	MBR-OPPMBR
Sibbald Point	30-Jun-14	14-Jul-14	MBR-OPPMBR
Sibbald Point	14-Jul-14	28-Jul-14	MBR-OPPMBR
Sibbald Point	28-Jul-14	11-Aug-14	MBR-OPPMBR
Sibbald Point	11-Aug-14	25-Aug-14	MBR-OPPMBR
Sibbald Point	25-Aug-14	8-Sep-14	MBR-OPPMBR
Sibbald Point	8-Sep-14	22-Sep-14	MBR-OPPMBR
Wasaga Beach	29-Apr-14	16-May-14	MBR-OPPMBW
Wasaga Beach	16-May-14	30-May-14	MBR-OPPMBW
Wasaga Beach	30-May-14	13-Jun-14	MBR-OPPMBW
Wasaga Beach	13-Jun-14	27-Jun-14	MBR-OPPMBW
Wasaga Beach	27-Jun-14	11-Jul-14	MBR-OPPMBW
Wasaga Beach	11-Jul-14	25-Jul-14	MBR-OPPMBW
Wasaga Beach	25-Jul-14	8-Aug-14	MBR-OPPMBW
Wasaga Beach	8-Aug-14	22-Aug-14	MBR-OPPMBW
Wasaga Beach	22-Aug-14	5-Sep-14	MBR-OPPMBW
Wasaga Beach	5-Sep-14	19-Sep-14	MBR-OPPMBW
Bronte Creek	28-Apr-14	12-May-14	MBR-OPPMAJ
Bronte Creek	12-May-14	26-May-14	MBR-OPPMAJ
Bronte Creek	26-May-14	9-Jun-14	MBR-OPPMAJ
Bronte Creek	9-Jun-14	23-Jun-14	MBR-OPPMAJ
Bronte Creek	23-Jun-14	7-Jul-14	MBR-OPPMAJ
Bronte Creek	7-Jul-14	21-Jul-14	MBR-OPPMAJ
Bronte Creek	21-Jul-14	4-Aug-14	MBR-OPPMAJ
Bronte Creek	4-Aug-14	18-Aug-14	MBR-OPPMAJ
Bronte Creek	18-Aug-14	1-Sep-14	MBR-OPPMAJ
Bronte Creek	1-Sep-14	15-Sep-14	MBR-OPPMAJ
James N Allan	28-Apr-14	12-May-14	MBR-OPPMAV
James N Allan	12-May-14	26-May-14	MBR-OPPMAV
James N Allan	26-May-14	9-Jun-14	MBR-OPPMAV
James N Allan	9-Jun-14	23-Jun-14	MBR-OPPMAV

James N Allan	23-Jun-14	7-Jul-14	MBR-OPPMAV
James N Allan	7-Jul-14	21-Jul-14	MBR-OPPMAV
James N Allan	21-Jul-14	4-Aug-14	MBR-OPPMAV
James N Allan	4-Aug-14	18-Aug-14	MBR-OPPMAV
James N Allan	18-Aug-14	1-Sep-14	MBR-OPPMAV
James N Allan	1-Sep-14	15-Sep-14	MBR-OPPMAV
John E Pearce	13-May-14	27-May-14	MBR-OPPMAWMAZ
John E Pearce	10-Jun-14	24-Jun-14	MBR-OPPMAWMAZ
John E Pearce	8-Jul-14	22-Jul-14	MBR-OPPMAWMAZ
John E Pearce	5-Aug-14	19-Aug-14	MBR-OPPMAWMAZ
John E Pearce	2-Sep-14	16-Sep-14	MBR-OPPMAWMAZ
Long Point	28-Apr-14	13-May-14	MBR-OPPMBY
Long Point	13-May-14	27-May-14	MBR-OPPMBY
Long Point	27-May-14	10-Jun-14	MBR-OPPMBY
Long Point	10-Jun-14	24-Jun-14	MBR-OPPMBY
Long Point	24-Jun-14	8-Jul-14	MBR-OPPMBY
Long Point	8-Jul-14	22-Jul-14	MBR-OPPMBY
Long Point	22-Jul-14	5-Aug-14	MBR-OPPMBY
Long Point	5-Aug-14	19-Aug-14	MBR-OPPMBY
Long Point	19-Aug-14	2-Sep-14	MBR-OPPMBY
Long Point	2-Sep-14	16-Sep-14	MBR-OPPMBY
Ojibway Prairie	14-May-14	28-May-14	MBR-OPPMBFMBH
Ojibway Prairie	11-Jun-14	25-Jun-14	MBR-OPPMBFMBH
Ojibway Prairie	9-Jul-14	23-Jul-14	MBR-OPPMBFMBH
Ojibway Prairie	6-Aug-14	20-Aug-14	MBR-OPPMBFMBH
Ojibway Prairie	3-Sep-14	17-Sep-14	MBR-OPPMBFMBH
Pinery	14-May-14	28-May-14	MBR-OPPMBFMBH
Pinery	11-Jun-14	25-Jun-14	MBR-OPPMBFMBH
Pinery	9-Jul-14	23-Jul-14	MBR-OPPMBFMBH
Pinery	6-Aug-14	20-Aug-14	MBR-OPPMBFMBH
Pinery	3-Sep-14	17-Sep-14	MBR-OPPMBFMBH
Port Burwell	29-Apr-14	13-May-14	MBR-OPPMBI
Port Burwell	13-May-14	27-May-14	MBR-OPPMBI
Port Burwell	27-May-14	10-Jun-14	MBR-OPPMBI
Port Burwell	10-Jun-14	24-Jun-14	MBR-OPPMBI
Port Burwell	24-Jun-14	8-Jul-14	MBR-OPPMBI
Port Burwell	8-Jul-14	22-Jul-14	MBR-OPPMBI
Port Burwell	22-Jul-14	5-Aug-14	MBR-OPPMBI
Port Burwell	5-Aug-14	19-Aug-14	MBR-OPPMBI
Port Burwell	19-Aug-14	2-Sep-14	MBR-OPPMBI
Port Burwell	2-Sep-14	16-Sep-14	MBR-OPPMBI
Rock Point	28-Apr-14	12-May-14	MBR-OPPMBL
Rock Point	12-May-14	26-May-14	MBR-OPPMBL
Rock Point	26-May-14	9-Jun-14	MBR-OPPMBL
Rock Point	9-Jun-14	23-Jun-14	MBR-OPPMBL
Rock Point	23-Jun-14	7-Jul-14	MBR-OPPMBL
Rock Point	7-Jul-14	21-Jul-14	MBR-OPPMBL

Rock Point	21-Jul-14	4-Aug-14	MBR-OPPMBL
Rock Point	4-Aug-14	18-Aug-14	MBR-OPPMBL
Rock Point	18-Aug-14	1-Sep-14	MBR-OPPMBL
Rock Point	1-Sep-14	15-Sep-14	MBR-OPPMBL
Rondeau	13-May-14	27-May-14	MBR-OPPMBMMBN
Rondeau	10-Jun-14	24-Jun-14	MBR-OPPMBMMBN
Rondeau	8-Jul-14	22-Jul-14	MBR-OPPMBMMBN
Rondeau	5-Aug-14	19-Aug-14	MBR-OPPMBMMBN
Rondeau	2-Sep-14	16-Sep-14	MBR-OPPMBMMBN
Selkirk	28-Apr-14	12-May-14	MBR-OPPMBO
Selkirk	12-May-14	26-May-14	MBR-OPPMBO
Selkirk	26-May-14	9-Jun-14	MBR-OPPMBO
Selkirk	9-Jun-14	23-Jun-14	MBR-OPPMBO
Selkirk	23-Jun-14	7-Jul-14	MBR-OPPMBO
Selkirk	7-Jul-14	21-Jul-14	MBR-OPPMBO
Selkirk	21-Jul-14	4-Aug-14	MBR-OPPMBO
Selkirk	4-Aug-14	18-Aug-14	MBR-OPPMBO
Selkirk	18-Aug-14	1-Sep-14	MBR-OPPMBO
Selkirk	1-Sep-14	15-Sep-14	MBR-OPPMBO
Short Hills	12-May-14	26-May-14	MBR-OPPMBQMBU
Short Hills	9-Jun-14	23-Jun-14	MBR-OPPMBQMBU
Short Hills	7-Jul-14	21-Jul-14	MBR-OPPMBQMBU
Short Hills	4-Aug-14	18-Aug-14	MBR-OPPMBQMBU
Short Hills	1-Sep-14	15-Sep-14	MBR-OPPMBQMBU
Turkey Point	12-May-14	26-May-14	MBR-OPPMBQMBU
Turkey Point	9-Jun-14	23-Jun-14	MBR-OPPMBQMBU
Turkey Point	7-Jul-14	21-Jul-14	MBR-OPPMBQMBU
Turkey Point	4-Aug-14	18-Aug-14	MBR-OPPMBQMBU
Turkey Point	1-Sep-14	15-Sep-14	MBR-OPPMBQMBU
Wheatley	29-Apr-14	13-May-14	MBR-OPPMBX
Wheatley	13-May-14	27-May-14	MBR-OPPMBX
Wheatley	27-May-14	10-Jun-14	MBR-OPPMBX
Wheatley	10-Jun-14	24-Jun-14	MBR-OPPMBX
Wheatley	24-Jun-14	8-Jul-14	MBR-OPPMBX
Wheatley	8-Jul-14	22-Jul-14	MBR-OPPMBX
Wheatley	22-Jul-14	5-Aug-14	MBR-OPPMBX
Wheatley	5-Aug-14	19-Aug-14	MBR-OPPMBX
Wheatley	19-Aug-14	2-Sep-14	MBR-OPPMBX
Wheatley	2-Sep-14	16-Sep-14	MBR-OPPMBX

unts for the 410 samples. ECF = Eastern Canadian Forest (15 sites); EGL = Eastern Great Lakes Fore

Run Name	Reads	Assigned reads	BINs total	BINs Bacteria	BINs Chordata	BINs non-arthropods
GMP#04618_CCDB-S5-0066	844737	315413	518	17	0	1
GMP#04620_CCDB-S5-0066	874866	275802	525	10	0	1
GMP#04622_CCDB-S5-0066	789560	236636	480	5	0	0
GMP#04624_CCDB-S5-0066	809049	354373	359	7	0	0
GMP#04626_CCDB-S5-0066	718303	296773	329	9	0	1
GMP#04597_CCDB-S5-0053	1E+06	435638	491	8	1	3
GMP#04598_CCDB-S5-0053	947819	383007	561	11	0	2
GMP#04599_CCDB-S5-0053	826499	357669	536	6	0	0
GMP#04600_CCDB-S5-0053	887071	310705	727	22	1	1
GMP#04602_CCDB-S5-0053	844585	329125	510	8	0	1
GMP#04603_CCDB-S5-0053	774474	329818	473	5	1	1
GMP#04604_CCDB-S5-0053	885213	360810	527	6	0	1
GMP#04605_CCDB-S5-0053	875623	349362	378	4	0	1
GMP#04606_CCDB-S5-0053	565808	197081	341	6	0	2
GMP#04611_CCDB-S5-0053	911899	339632	825	6	2	1
GMP#04658_CCDB-S5-0068	638807	197057	743	25	0	0
GMP#04660_CCDB-S5-0068	680480	205457	1178	22	1	3
GMP#04662_CCDB-S5-0068	655110	185354	932	11	0	3
GMP#04664_CCDB-S5-0068	646714	196766	611	12	0	2
GMP#04666_CCDB-S5-0068	498632	144725	387	5	0	2
GMP#03668_CCDB-S5-0086	751958	283465	598	25	0	0
GMP#03670_CCDB-S5-0086	730555	254799	799	16	0	2
GMP#03672_CCDB-S5-0086	676557	244764	791	9	0	3
GMP#03674_CCDB-S5-0086	712717	246017	643	20	0	4
GMP#04582_CCDB-S5-0086	620386	209139	353	12	0	4
GMP#03708_CCDB-S5-0073	1E+06	333977	890	7	1	2
GMP#03710_CCDB-S5-0073	990071	310127	994	22	1	2
GMP#03712_CCDB-S5-0073	895735	314851	1053	21	1	7
GMP#03714_CCDB-S5-0073	921329	341614	867	11	0	4
GMP#04587_CCDB-S5-0073	916810	384385	604	5	0	0
GMP#03716_CCDB-S5-0069	807008	263178	907	20	0	1
GMP#03718_CCDB-S5-0069	772854	263942	1093	11	1	2
GMP#03720_CCDB-S5-0069	726646	251745	1019	7	0	3
GMP#03722_CCDB-S5-0069	766997	273278	745	19	0	4
GMP#04588_CCDB-S5-0069	795459	341835	510	5	0	4
GMP#03611_CCDB-S5-0065	912045	356130	502	17	0	3
GMP#03612_CCDB-S5-0065	874160	343593	896	19	0	0
GMP#03613_CCDB-S5-0065	849050	316507	656	5	0	0
GMP#03614_CCDB-S5-0065	850842	317868	761	6	0	1
GMP#03615_CCDB-S5-0065	823052	297333	838	8	0	0
GMP#03616_CCDB-S5-0065	799424	300409	633	11	0	1
GMP#03617_CCDB-S5-0065	747554	280113	621	13	0	0

GMP#03618_CCDB-S5-0065	872463	407894	696	17	0	0
GMP#04523_CCDB-S5-0065	831488	373536	481	18	0	0
GMP#04575_CCDB-S5-0065	724719	294954	355	10	1	0
GMP#03659_CCDB-S5-0081	953516	348779	470	11	0	1
GMP#03660_CCDB-S5-0081	904431	326084	738	23	0	0
GMP#03661_CCDB-S5-0081	785604	273687	493	16	0	3
GMP#03662_CCDB-S5-0081	860141	311817	785	4	0	3
GMP#03663_CCDB-S5-0081	733692	237245	739	8	1	2
GMP#03664_CCDB-S5-0081	834801	259273	697	6	1	2
GMP#03665_CCDB-S5-0081	768612	270019	799	12	1	0
GMP#03666_CCDB-S5-0081	773713	271422	685	12	0	3
GMP#04529_CCDB-S5-0081	760724	264304	615	10	0	6
GMP#04581_CCDB-S5-0081	775526	286958	411	6	1	6
GMP#04648_CCDB-S5-0072	983613	353835	865	23	0	1
GMP#04650_CCDB-S5-0072	944474	327415	1042	11	0	3
GMP#04652_CCDB-S5-0072	945176	349294	886	22	0	4
GMP#04654_CCDB-S5-0072	1E+06	409702	761	14	0	5
GMP#04656_CCDB-S5-0072	839242	347173	407	7	0	2
GMP#03700_CCDB-S5-0072	918087	307945	1283	11	0	2
GMP#03702_CCDB-S5-0072	835990	305411	1563	28	0	1
GMP#03704_CCDB-S5-0072	918549	310558	1466	24	0	5
GMP#03706_CCDB-S5-0072	928385	358668	1113	15	0	0
GMP#04586_CCDB-S5-0072	939127	378176	632	16	0	1
GMP#03635_CCDB-S5-0083	1E+06	391954	620	14	0	0
GMP#03636_CCDB-S5-0083	1E+06	384768	878	16	0	0
GMP#03637_CCDB-S5-0083	916303	333876	906	16	0	0
GMP#03638_CCDB-S5-0083	938924	331195	770	9	0	0
GMP#03639_CCDB-S5-0083	833565	311163	1066	21	0	1
GMP#03640_CCDB-S5-0083	912435	355537	988	17	0	1
GMP#03641_CCDB-S5-0083	824265	314533	915	12	0	1
GMP#03642_CCDB-S5-0083	914876	342733	765	22	1	3
GMP#04526_CCDB-S5-0083	830065	358410	599	14	0	0
GMP#04578_CCDB-S5-0083	918003	410741	542	6	0	1
GMP#03683_CCDB-S5-0055	862000	309719	656	7	0	1
GMP#03684_CCDB-S5-0055	782739	281804	841	24	0	1
GMP#03685_CCDB-S5-0055	776436	305587	809	9	0	2
GMP#03686_CCDB-S5-0055	833241	343236	894	11	0	2
GMP#03687_CCDB-S5-0055	732624	293650	930	12	0	2
GMP#03688_CCDB-S5-0055	731893	276668	830	23	0	1
GMP#03689_CCDB-S5-0055	712920	301577	730	13	1	1
GMP#03690_CCDB-S5-0055	801316	334252	581	6	1	2
GMP#04532_CCDB-S5-0055	793053	308642	688	10	1	3
GMP#04584_CCDB-S5-0055	691937	282548	457	11	0	0
GMP#03651_CCDB-S5-0089	802981	249918	511	9	0	2
GMP#03652_CCDB-S5-0089	888062	289299	625	12	1	1
GMP#03653_CCDB-S5-0089	830306	317280	60	10	0	0
GMP#03654_CCDB-S5-0089	829714	285474	764	10	0	0

GMP#03655_CCDB-S5-0089	781166	301858	864	10	0	3
GMP#03656_CCDB-S5-0089	777000	275946	922	10	0	1
GMP#03657_CCDB-S5-0089	717115	228910	883	10	0	1
GMP#03658_CCDB-S5-0089	798452	235324	821	10	0	3
GMP#04528_CCDB-S5-0089	663059	233401	680	20	2	7
GMP#04580_CCDB-S5-0089	677856	252144	442	9	0	3
GMP#03691_CCDB-S5-0090	854380	263002	953	9	0	0
GMP#03692_CCDB-S5-0090	850977	259150	1316	11	0	1
GMP#03693_CCDB-S5-0090	818324	262921	1371	15	0	1
GMP#03694_CCDB-S5-0090	698069	229698	1316	8	0	1
GMP#03695_CCDB-S5-0090	606866	222044	1222	13	0	0
GMP#03696_CCDB-S5-0090	718354	231254	1295	24	0	1
GMP#03697_CCDB-S5-0090	777805	265690	1183	28	0	1
GMP#03698_CCDB-S5-0090	777946	261094	830	29	0	0
GMP#04533_CCDB-S5-0090	673941	230045	868	12	0	0
GMP#04585_CCDB-S5-0090	708537	253210	600	14	0	0
GMP#04637_CCDB-S5-0073	1E+06	330625	575	10	0	0
GMP#04638_CCDB-S5-0073	929115	289972	828	20	0	1
GMP#04639_CCDB-S5-0073	935574	276120	1062	11	0	1
GMP#04640_CCDB-S5-0073	948650	289599	1420	20	0	4
GMP#04641_CCDB-S5-0073	913071	287466	1095	14	0	3
GMP#04642_CCDB-S5-0073	880011	264949	1136	15	0	3
GMP#04643_CCDB-S5-0073	857327	256814	1106	18	0	1
GMP#04644_CCDB-S5-0073	815565	259401	1032	17	0	5
GMP#04645_CCDB-S5-0073	828975	269341	894	18	0	4
GMP#04646_CCDB-S5-0073	781088	261623	612	18	0	3
GMP#03444_CCDB-S5-0066	818191	300493	526	7	0	6
GMP#03446_CCDB-S5-0066	718570	297534	859	12	0	0
GMP#03448_CCDB-S5-0066	736111	306259	932	10	0	2
GMP#03450_CCDB-S5-0066	724520	287645	842	14	0	7
GMP#04570_CCDB-S5-0066	630232	236621	531	19	0	7
GMP#03628_CCDB-S5-0067	911348	339448	730	13	0	3
GMP#03630_CCDB-S5-0067	798118	290456	973	5	0	1
GMP#03632_CCDB-S5-0067	775026	277677	934	9	1	2
GMP#03634_CCDB-S5-0067	851003	310549	857	8	0	1
GMP#04577_CCDB-S5-0067	857458	385900	518	10	0	3
GMP#03412_CCDB-S5-0067	755669	311612	600	11	0	4
GMP#03414_CCDB-S5-0067	687590	281112	1164	19	1	2
GMP#03416_CCDB-S5-0067	721775	284362	1307	18	0	0
GMP#03418_CCDB-S5-0067	728550	292256	1044	10	0	1
GMP#04566_CCDB-S5-0067	679848	251276	710	18	0	2
GMP#03379_CCDB-S5-0068	742597	199645	261	6	0	2
GMP#03380_CCDB-S5-0068	722317	216954	306	0	0	2
GMP#03381_CCDB-S5-0068	690384	186452	617	11	0	3
GMP#03382_CCDB-S5-0068	756436	203826	694	7	0	1
GMP#03383_CCDB-S5-0068	727852	236496	683	7	0	0
GMP#03384_CCDB-S5-0068	639616	212061	783	8	0	0

GMP#03385_CCDB-S5-0068	648999	196751	687	9	0	3
GMP#03386_CCDB-S5-0068	697244	233723	539	11	0	1
GMP#04510_CCDB-S5-0068	718558	300781	435	7	0	1
GMP#04562_CCDB-S5-0068	701255	229871	353	11	0	3
GMP#03468_CCDB-S5-0068	583784	181783	419	12	0	1
GMP#03470_CCDB-S5-0068	455387	105497	377	4	0	1
GMP#03472_CCDB-S5-0068	546965	146622	459	13	0	2
GMP#03474_CCDB-S5-0068	546421	150288	318	8	0	3
GMP#04573_CCDB-S5-0068	526329	151831	245	3	0	3
GMP#03419_CCDB-S5-0058	963348	356781	496	10	0	2
GMP#03420_CCDB-S5-0058	878953	317573	468	7	0	1
GMP#03421_CCDB-S5-0058	754664	282724	455	7	1	0
GMP#03422_CCDB-S5-0058	704202	235004	481	5	0	2
GMP#03423_CCDB-S5-0058	801010	283845	686	5	0	1
GMP#03424_CCDB-S5-0058	909771	355905	565	20	0	2
GMP#03425_CCDB-S5-0058	849900	303399	495	5	0	1
GMP#03426_CCDB-S5-0058	849567	347503	378	10	0	2
GMP#04515_CCDB-S5-0058	888901	368971	404	12	0	6
GMP#04567_CCDB-S5-0058	784765	270055	355	5	0	1
GMP#03459_CCDB-S5-0128	848279	237052	349	5	0	2
GMP#03460_CCDB-S5-0128	852008	273970	351	8	0	0
GMP#03461_CCDB-S5-0128	803327	272539	358	7	0	0
GMP#03462_CCDB-S5-0128	827062	277298	393	4	0	1
GMP#03463_CCDB-S5-0128	807062	263846	521	8	0	2
GMP#03464_CCDB-S5-0128	865938	328648	459	18	0	2
GMP#03465_CCDB-S5-0128	829864	324731	387	10	0	4
GMP#03466_CCDB-S5-0128	807509	329222	336	14	0	1
GMP#04520_CCDB-S5-0128	734386	249238	393	19	0	1
GMP#04572_CCDB-S5-0128	730637	234816	249	5	0	3
GMP#03747_CCDB-S5-0061	878902	338840	477	3	0	0
GMP#03748_CCDB-S5-0061	840003	292504	543	7	0	2
GMP#03749_CCDB-S5-0061	753001	291661	496	7	0	1
GMP#03750_CCDB-S5-0061	729427	274036	581	19	0	1
GMP#03751_CCDB-S5-0061	718291	254407	561	23	0	3
GMP#03752_CCDB-S5-0061	778332	309110	524	16	1	1
GMP#03753_CCDB-S5-0061	693375	261176	533	9	1	2
GMP#03754_CCDB-S5-0061	627167	223957	515	8	0	2
GMP#04540_CCDB-S5-0061	714422	282074	527	14	0	4
GMP#04592_CCDB-S5-0061	779544	362086	344	5	0	2
GMP#03740_CCDB-S5-0073	930942	317855	1208	13	0	1
GMP#03742_CCDB-S5-0073	822709	315539	1227	16	0	0
GMP#03744_CCDB-S5-0073	864538	315939	1339	14	1	0
GMP#03746_CCDB-S5-0073	811779	296579	1025	12	0	2
GMP#04591_CCDB-S5-0073	766414	192375	547	10	0	1
GMP#04015_CCDB-S5-0063	953951	342030	393	5	0	2
GMP#04016_CCDB-S5-0063	1E+06	391623	690	9	0	0
GMP#04017_CCDB-S5-0063	950426	398874	836	11	0	5

GMP#04018_CCDB-S5-0063	943285	377441	816	6	0	1
GMP#04019_CCDB-S5-0063	888530	347988	1026	14	0	2
GMP#04020_CCDB-S5-0063	887030	359891	896	21	0	4
GMP#04021_CCDB-S5-0063	910778	332673	938	18	0	4
GMP#04022_CCDB-S5-0063	897965	371497	811	16	0	7
GMP#04544_CCDB-S5-0063	922643	372252	847	21	1	8
GMP#04596_CCDB-S5-0063	804347	359515	537	8	0	4
GMP#03603_CCDB-S5-0064	812146	313434	558	13	1	1
GMP#03604_CCDB-S5-0064	778900	281111	650	15	0	0
GMP#03605_CCDB-S5-0064	715926	275979	577	14	0	2
GMP#03606_CCDB-S5-0064	747657	278602	774	16	1	2
GMP#03607_CCDB-S5-0064	722911	274048	924	23	0	3
GMP#03608_CCDB-S5-0064	709151	266795	766	27	0	1
GMP#03609_CCDB-S5-0064	668655	248312	581	17	1	2
GMP#03610_CCDB-S5-0064	709589	296312	607	10	0	3
GMP#04522_CCDB-S5-0064	702446	265204	606	7	0	6
GMP#04574_CCDB-S5-0064	643055	275261	351	4	0	5
GMP#03403_CCDB-S5-0054	976643	416907	354	6	1	2
GMP#03404_CCDB-S5-0054	953456	424032	347	3	0	0
GMP#03405_CCDB-S5-0054	912008	376098	286	3	0	1
GMP#03406_CCDB-S5-0054	885956	386410	250	6	1	0
GMP#03407_CCDB-S5-0054	882785	351437	359	5	0	2
GMP#03408_CCDB-S5-0054	822028	364232	366	5	0	2
GMP#03409_CCDB-S5-0054	690122	256323	280	10	0	2
GMP#03410_CCDB-S5-0054	797965	269761	340	4	1	1
GMP#04513_CCDB-S5-0054	676899	348743	325	15	1	2
GMP#04565_CCDB-S5-0054	617635	227541	237	5	1	4
GMP#03364_CCDB-S5-0069	727499	312861	339	1	0	0
GMP#03366_CCDB-S5-0069	637841	228034	498	9	0	0
GMP#03368_CCDB-S5-0069	708060	242726	765	15	0	0
GMP#03370_CCDB-S5-0069	705468	281080	488	9	0	0
GMP#04560_CCDB-S5-0069	685601	208310	332	6	0	0
GMP#03387_CCDB-S5-0080	724654	188683	291	4	0	0
GMP#03388_CCDB-S5-0080	769501	216000	509	2	0	1
GMP#03389_CCDB-S5-0080	737162	223530	509	1	0	2
GMP#03390_CCDB-S5-0080	807198	325949	362	7	0	1
GMP#03391_CCDB-S5-0080	691388	202381	530	8	0	0
GMP#03392_CCDB-S5-0080	671234	174502	658	5	0	0
GMP#03393_CCDB-S5-0080	673577	186278	525	9	0	4
GMP#03394_CCDB-S5-0080	687943	190237	556	7	0	1
GMP#04511_CCDB-S5-0080	678894	189490	511	11	0	2
GMP#04563_CCDB-S5-0080	671947	201213	348	7	0	0
GMP#03396_CCDB-S5-0070	955478	376080	468	6	0	1
GMP#03398_CCDB-S5-0070	873506	324209	435	7	0	4
GMP#03400_CCDB-S5-0070	866966	340401	636	7	0	3
GMP#03402_CCDB-S5-0070	899173	335398	585	13	1	5
GMP#04564_CCDB-S5-0070	799233	536222	419	10	0	6

GMP#03371_CCDB-S5-0082	882710	316325	435	8	0	1
GMP#03372_CCDB-S5-0082	945078	386904	535	11	0	1
GMP#03373_CCDB-S5-0082	921332	375495	633	8	0	0
GMP#03374_CCDB-S5-0082	861726	333858	728	11	0	0
GMP#03375_CCDB-S5-0082	814354	330629	816	24	0	1
GMP#03376_CCDB-S5-0082	874746	321321	811	11	0	0
GMP#03377_CCDB-S5-0082	771196	305828	670	8	0	1
GMP#03378_CCDB-S5-0082	827106	308378	732	13	0	1
GMP#04509_CCDB-S5-0082	863654	354738	712	14	2	0
GMP#04561_CCDB-S5-0082	880169	351010	464	13	0	3
GMP#03755_CCDB-S5-0056	1E+06	463735	487	7	0	1
GMP#03756_CCDB-S5-0056	1E+06	487306	391	5	0	0
GMP#03757_CCDB-S5-0056	928450	422380	449	7	0	0
GMP#03758_CCDB-S5-0056	916766	391473	405	7	0	0
GMP#03759_CCDB-S5-0056	960472	440521	495	23	0	0
GMP#03760_CCDB-S5-0056	911696	406508	477	6	0	2
GMP#03761_CCDB-S5-0056	871916	398810	492	7	0	0
GMP#03762_CCDB-S5-0056	926145	333928	360	2	0	1
GMP#04541_CCDB-S5-0056	902908	286299	388	6	0	2
GMP#04593_CCDB-S5-0056	830300	343134	308	5	0	2
GMP#03356_CCDB-S5-0086	709672	255906	432	3	1	1
GMP#03358_CCDB-S5-0086	625540	209229	494	14	0	0
GMP#03360_CCDB-S5-0086	698041	198459	478	3	0	0
GMP#03362_CCDB-S5-0086	611572	173269	451	7	0	0
GMP#04559_CCDB-S5-0086	610537	187651	395	7	0	1
GMP#03647_CCDB-S5-0052	763316	325913	1207	35	0	2
GMP#03771_CCDB-S5-0052	897942	370475	940	9	0	3
GMP#03772_CCDB-S5-0052	842295	325496	1394	24	0	3
GMP#03773_CCDB-S5-0052	775032	302832	1537	22	0	3
GMP#03775_CCDB-S5-0052	779646	321307	1666	26	0	2
GMP#03776_CCDB-S5-0052	707897	293069	1629	26	0	5
GMP#03777_CCDB-S5-0052	691282	284577	915	10	0	6
GMP#03778_CCDB-S5-0052	475431	198844	204	8	0	3
GMP#04543_CCDB-S5-0052	722631	317127	911	2	0	2
GMP#04595_CCDB-S5-0052	727262	308412	652	22	0	3
GMP#03731_CCDB-S5-0085	902173	332139	629	6	0	0
GMP#03732_CCDB-S5-0085	812687	328324	711	5	0	0
GMP#03733_CCDB-S5-0085	953592	364540	969	20	0	1
GMP#03734_CCDB-S5-0085	926873	324161	1189	27	0	0
GMP#03735_CCDB-S5-0085	845205	305776	813	19	1	0
GMP#03736_CCDB-S5-0085	887429	333925	958	40	2	2
GMP#03737_CCDB-S5-0085	819898	326999	806	19	0	0
GMP#03738_CCDB-S5-0085	924025	360982	783	13	0	0
GMP#04538_CCDB-S5-0085	836419	317948	851	12	0	0
GMP#04590_CCDB-S5-0085	877836	284547	755	8	1	0
GMP#03427_CCDB-S5-0071	801574	189413	442	9	0	0
GMP#03428_CCDB-S5-0071	713379	168434	652	11	0	0

GMP#03429_CCDB-S5-0071	712297	220526	1020	16	0	0
GMP#03430_CCDB-S5-0071	681139	209623	973	19	1	1
GMP#03431_CCDB-S5-0071	622961	184194	1078	18	0	0
GMP#03432_CCDB-S5-0071	624171	173160	895	15	0	2
GMP#03433_CCDB-S5-0071	673392	190338	979	22	0	1
GMP#03434_CCDB-S5-0071	718818	238525	933	14	0	2
GMP#04516_CCDB-S5-0071	671897	235804	953	22	0	1
GMP#04568_CCDB-S5-0071	693012	187564	606	21	0	0
GMP#03724_CCDB-S5-0075	1E+06	373650	359	4	1	1
GMP#03726_CCDB-S5-0075	906681	388764	408	13	2	1
GMP#03728_CCDB-S5-0075	919020	379444	424	6	0	0
GMP#03730_CCDB-S5-0075	935484	419939	380	10	5	1
GMP#04589_CCDB-S5-0075	907357	339848	288	5	2	0
GMP#03763_CCDB-S5-0088	808088	280023	421	5	0	1
GMP#03764_CCDB-S5-0088	900686	306367	396	1	0	1
GMP#03765_CCDB-S5-0088_	837190	290440	539	7	0	3
GMP#03766_CCDB-S5-0088	847666	304247	598	14	0	1
GMP#03767_CCDB-S5-0088	796220	313575	610	7	0	4
GMP#03768_CCDB-S5-0088	827020	309426	510	8	0	3
GMP#03769_CCDB-S5-0088	732922	264246	466	9	0	1
GMP#03770_CCDB-S5-0088	782761	268657	410	17	0	2
GMP#04542_CCDB-S5-0088	763727	321601	340	14	0	1
GMP#04594_CCDB-S5-0088	701369	239843	281	9	0	1
GMP#03435_CCDB-S5-0074	929191	467503	346	3	0	0
GMP#03436_CCDB-S5-0074	851075	440834	334	3	0	1
GMP#03437_CCDB-S5-0074	757829	405491	380	6	0	1
GMP#03438_CCDB-S5-0074	808294	363054	394	7	0	0
GMP#03439_CCDB-S5-0074	781852	396625	494	6	1	3
GMP#03440_CCDB-S5-0074	766615	385711	488	14	0	2
GMP#03441_CCDB-S5-0074	778874	373429	478	6	0	1
GMP#03442_CCDB-S5-0074	781876	377107	508	7	0	5
GMP#04517_CCDB-S5-0074	748086	399949	497	6	0	2
GMP#04569_CCDB-S5-0074	791391	407630	335	5	0	0
GMP#03243_CCDB-S5-0057	658058	230976	323	4	0	0
GMP#03244_CCDB-S5-0057	651019	206673	471	5	0	2
GMP#03245_CCDB-S5-0057	580340	219014	788	21	0	2
GMP#03246_CCDB-S5-0057	624816	233120	773	19	0	0
GMP#03247_CCDB-S5-0057	603783	219138	1092	19	0	2
GMP#03248_CCDB-S5-0057	704885	272582	954	15	0	3
GMP#03249_CCDB-S5-0057	631565	235867	944	11	1	5
GMP#03250_CCDB-S5-0057	671111	238253	820	11	0	4
GMP#04493_CCDB-S5-0057	634880	237862	841	9	0	4
GMP#04545_CCDB-S5-0057	668655	266055	707	15	0	4
GMP#03274_CCDB-S5-0129	594922	167822	489	6	0	3
GMP#03267_CCDB-S5-0129	611927	134643	184	1	0	0
GMP#03268_CCDB-S5-0129	582525	138953	259	2	0	0
GMP#03269_CCDB-S5-0129	687894	161299	416	7	0	1

GMP#03270_CCDB-S5-0129	677540	159367	613	8	0	0
GMP#03271_CCDB-S5-0129	596602	135699	673	11	0	0
GMP#03272_CCDB-S5-0129	569798	133587	682	11	0	3
GMP#03273_CCDB-S5-0129	562811	149439	605	12	0	0
GMP#04496_CCDB-S5-0129	595953	153347	570	11	0	3
GMP#04548_CCDB-S5-0129	538915	114538	505	11	0	0
GMP#03308_CCDB-S5-0070	836894	250266	338	5	0	1
GMP#03310_CCDB-S5-0070	1E+06	223318	382	10	0	0
GMP#03312_CCDB-S5-0070	978009	214884	371	9	0	1
GMP#03314_CCDB-S5-0070	1E+06	428952	354	6	0	5
GMP#04553_CCDB-S5-0070	863571	291674	314	8	0	3
GMP#03291_CCDB-S5-0041	1E+06	461680	273	4	0	0
GMP#03292_CCDB-S5-0041	1E+06	441793	292	1	0	0
GMP#03293_CCDB-S5-0041	2E+06	760581	467	4	0	0
GMP#03294_CCDB-S5-0041	906817	338921	496	1	0	0
GMP#03295_CCDB-S5-0041	915831	291299	578	8	0	1
GMP#03296_CCDB-S5-0041	736485	229475	148	4	0	0
GMP#03297_CCDB-S5-0041	922361	296195	490	19	0	0
GMP#03298_CCDB-S5-0041	800802	381316	160	8	0	0
GMP#04499_CCDB-S5-0041	846228	305874	242	4	0	0
GMP#04551_CCDB-S5-0041	807700	311652	382	10	0	0
GMP#03332_CCDB-S5-0074	919805	299290	590	7	0	0
GMP#03334_CCDB-S5-0074	918899	308083	1179	26	0	1
GMP#03336_CCDB-S5-0074	804085	251805	1045	17	0	1
GMP#03338_CCDB-S5-0074	861740	279808	914	22	0	0
GMP#04556_CCDB-S5-0074	852877	310872	732	15	0	0
GMP#03348_CCDB-S5-0074	930698	292992	498	11	0	0
GMP#03350_CCDB-S5-0074	875855	247325	615	8	0	0
GMP#03352_CCDB-S5-0074	941329	286145	528	13	0	0
GMP#03354_CCDB-S5-0074	958263	319172	424	12	0	1
GMP#04558_CCDB-S5-0074	919608	366344	478	11	0	0
GMP#03299_CCDB-S5-0084	1E+06	419838	231	4	0	0
GMP#03300_CCDB-S5-0084	911682	221993	222	3	0	0
GMP#03301_CCDB-S5-0084	824772	195299	199	2	0	0
GMP#03302_CCDB-S5-0084	774369	191336	216	2	0	4
GMP#03303_CCDB-S5-0084	760139	199065	382	5	0	5
GMP#03304_CCDB-S5-0084	788998	210108	359	14	0	5
GMP#03305_CCDB-S5-0084	828756	315063	169	5	0	1
GMP#03306_CCDB-S5-0084	868031	283246	225	6	0	0
GMP#04500_CCDB-S5-0084	675841	198372	140	5	0	0
GMP#04552_CCDB-S5-0084	862757	231951	243	1	0	1
GMP#03259_CCDB-S5-0072	839122	208018	280	2	0	0
GMP#03260_CCDB-S5-0072	793412	192077	330	10	0	0
GMP#03261_CCDB-S5-0072	700461	129223	458	8	0	0
GMP#03262_CCDB-S5-0072	790337	177406	699	9	0	0
GMP#03263_CCDB-S5-0072	788186	182834	727	8	0	0
GMP#03264_CCDB-S5-0072	766060	196415	660	10	0	0

GMP#03265_CCDB-S5-0072	691100	189498	644	11	0	1
GMP#03266_CCDB-S5-0072	718469	207725	614	12	0	0
GMP#04495_CCDB-S5-0072	722979	204088	607	14	0	1
GMP#04547_CCDB-S5-0072	710206	219406	458	9	0	1
GMP#03316_CCDB-S5-0075	1E+06	345559	394	11	1	0
GMP#03318_CCDB-S5-0075	1E+06	299784	457	7	1	0
GMP#03320_CCDB-S5-0075	1E+06	339094	427	11	2	1
GMP#03322_CCDB-S5-0075	1E+06	364079	322	8	1	1
GMP#04554_CCDB-S5-0075	833385	250949	372	3	0	2
GMP#03275_CCDB-S5-0087	843237	291443	290	3	0	0
GMP#03276_CCDB-S5-0087	942794	274170	350	6	0	0
GMP#03277_CCDB-S5-0087	840624	225261	309	4	0	0
GMP#03278_CCDB-S5-0087_	882045	216876	292	4	0	0
GMP#03279_CCDB-S5-0087	767299	169388	379	15	1	1
GMP#03280_CCDB-S5-0087	772567	215244	298	19	1	0
GMP#03281_CCDB-S5-0087	771385	192737	306	8	0	1
GMP#03282_CCDB-S5-0087	826300	225597	254	12	0	1
GMP#04497_CCDB-S5-0087	805237	316186	193	5	0	0
GMP#04549_CCDB-S5-0087	723173	178140	176	1	0	0
GMP#03252_CCDB-S5-0079	784032	291126	536	16	0	0
GMP#03254_CCDB-S5-0079	742214	268908	875	16	0	2
GMP#03256_CCDB-S5-0079	714520	274769	859	20	0	2
GMP#03258_CCDB-S5-0079	750246	286871	706	21	0	2
GMP#04546_CCDB-S5-0079	740631	301043	653	15	0	2
GMP#03284_CCDB-S5-0079	689965	218969	542	6	0	0
GMP#03286_CCDB-S5-0079	710855	208956	826	14	0	0
GMP#03288_CCDB-S5-0079	724419	202132	747	12	0	2
GMP#03290_CCDB-S5-0079	670259	199365	642	6	0	4
GMP#04550_CCDB-S5-0079	678548	193417	527	6	0	0
GMP#03323_CCDB-S5-0097	1E+06	383053	273	2	0	1
GMP#03324_CCDB-S5-0097	799151	195785	283	0	1	1
GMP#03325_CCDB-S5-0097	760957	177634	242	1	0	1
GMP#03326_CCDB-S5-0097	785048	179129	301	3	1	1
GMP#03327_CCDB-S5-0097	710855	170800	375	7	1	1
GMP#03328_CCDB-S5-0097	778572	233055	336	7	1	1
GMP#03329_CCDB-S5-0097	864601	282136	213	4	1	0
GMP#03330_CCDB-S5-0097	844843	249372	227	3	1	1
GMP#04503_CCDB-S5-0097	735171	206937	275	3	0	1
GMP#04555_CCDB-S5-0097	689051	213927	211	6	2	2

sts (24 sites); SGL = Southern Great Lakes Forests (13 sites).

BINs non-insects	BINs insects	OTU Count
25	475	28
44	471	20
39	437	31
37	315	40
28	292	20
54	425	37
52	496	36
34	496	28
69	634	26
47	454	49
51	415	31
78	442	50
69	304	52
57	276	34
66	750	39
44	674	9
47	1106	9
38	880	8
33	564	9
26	355	12
42	532	31
64	717	26
37	742	26
62	557	24
40	297	32
52	829	35
57	913	38
53	972	38
71	781	30
59	540	30
36	850	28
49	1030	15
42	967	9
50	672	21
26	475	30
20	462	36
36	843	15
35	617	25
47	708	33
55	776	23
40	581	20
37	572	20

41	638	25
32	431	25
26	318	20
44	414	31
44	672	19
35	439	34
52	727	36
52	677	41
45	645	32
36	750	33
42	629	22
39	561	32
32	368	27
52	789	41
58	970	46
52	808	38
50	692	48
35	363	30
41	1229	34
46	1489	20
29	1409	38
45	1054	46
32	583	47
32	574	35
61	799	24
59	832	21
64	696	31
58	987	36
59	913	33
40	863	29
48	691	34
42	544	32
44	491	58
42	610	31
35	781	20
52	747	12
45	837	25
39	878	28
41	765	35
33	683	31
46	527	38
42	632	30
34	412	30
41	459	26
47	565	33
57	534	34
62	695	29

42	812	29
47	868	25
38	836	49
47	764	66
46	605	39
31	399	24
28	918	19
24	1280	21
51	1309	22
31	1276	18
23	1186	22
24	1248	17
25	1130	23
24	777	31
29	827	16
23	563	19
30	535	43
47	760	30
48	1003	20
51	1346	23
34	1046	35
31	1088	20
24	1065	27
30	981	27
29	843	34
34	557	26
32	483	26
45	803	33
57	864	28
52	769	14
46	459	16
27	688	33
29	939	20
31	891	20
25	823	51
26	479	40
20	566	20
17	1128	23
24	1266	28
20	1013	18
22	669	28
29	224	37
19	285	17
35	568	22
39	647	31
45	631	35
44	731	34

41	634	37
41	486	38
34	393	45
30	309	30
13	393	3
18	354	5
16	428	3
5	302	5
10	229	11
26	458	37
22	439	31
39	408	24
38	436	31
39	641	25
39	504	52
31	458	38
24	343	28
35	351	37
33	316	52
27	315	25
40	303	27
24	327	46
32	356	38
39	472	38
32	407	48
31	343	39
33	288	32
31	342	36
40	202	26
37	439	35
33	501	23
32	457	30
44	517	34
54	482	14
34	473	29
47	474	32
43	462	27
43	466	36
29	308	22
43	1151	24
56	1156	11
57	1268	12
72	939	11
41	496	29
32	355	44
43	640	45
29	792	32

25	785	33
38	974	27
33	839	31
40	879	32
32	757	21
32	785	35
38	487	25
31	512	30
50	586	32
34	527	30
39	717	31
40	859	20
36	702	24
31	531	20
42	552	37
41	552	17
32	310	20
18	328	40
23	321	28
38	244	19
34	209	20
29	323	32
38	321	28
22	246	23
28	306	22
21	286	12
24	203	16
29	309	28
55	434	11
44	706	14
35	445	27
32	295	20
8	280	29
12	495	32
21	487	18
18	337	27
15	508	22
23	631	19
15	498	26
24	524	33
23	476	28
17	325	19
32	430	39
37	388	30
48	578	27
36	530	35
45	358	43

19	408	34
40	484	39
43	583	39
42	676	20
32	759	26
54	746	26
39	622	27
41	677	28
37	659	20
30	419	41
61	418	30
61	325	36
58	384	24
58	340	28
51	421	27
53	416	33
47	438	33
47	310	31
57	323	50
51	251	39
44	383	18
49	431	13
50	425	27
45	399	26
47	340	17
34	1137	28
24	905	32
37	1332	19
36	1477	21
41	1599	26
35	1565	21
30	870	20
18	625	30
5	195	31
29	859	25
27	597	31
33	675	22
52	897	27
47	1117	26
48	748	25
48	868	30
47	741	18
63	707	26
45	796	31
52	695	40
10	423	30
10	632	23

16	988	28
14	940	16
17	1046	21
13	865	17
16	940	19
13	904	22
24	906	23
22	563	20
47	307	23
49	343	34
50	368	38
56	308	39
38	244	28
28	387	20
27	368	22
59	470	29
53	530	34
52	548	33
48	451	28
40	416	27
44	347	37
24	301	42
28	243	19
39	304	94
50	280	98
55	318	89
54	333	91
79	406	93
64	408	112
46	425	94
52	444	98
62	427	103
48	282	97
13	306	8
25	440	13
34	732	13
36	721	11
41	1032	9
52	886	15
41	887	14
46	760	16
47	781	9
45	645	9
15	465	21
7	176	12
7	250	20
23	385	29

21	584	13
20	642	16
18	650	8
26	567	14
11	545	18
12	482	10
32	300	40
35	337	16
32	329	19
47	296	46
47	256	34
13	257	41
7	284	46
12	451	57
12	484	21
17	553	17
18	126	9
20	452	19
19	133	11
7	231	30
18	355	25
23	562	26
40	1112	23
40	988	22
44	848	16
34	684	25
29	458	40
51	556	20
64	451	33
60	351	48
61	407	26
28	199	27
30	189	18
20	177	13
18	192	13
36	336	18
38	302	18
20	143	24
33	186	20
13	122	20
31	210	16
8	270	20
21	299	18
19	432	13
37	654	18
32	689	18
37	614	20

38	595	24
30	572	24
30	562	25
24	424	21
50	333	31
61	388	33
73	340	38
58	254	37
81	286	29
31	257	26
44	303	31
44	261	24
39	249	31
34	329	30
28	250	27
46	251	23
43	198	33
32	156	13
32	143	17
40	480	28
43	815	20
36	802	18
39	644	26
54	583	24
59	480	28
64	749	11
66	668	18
60	572	21
66	455	22
22	248	21
26	255	13
20	220	8
27	269	9
33	333	11
42	285	21
32	176	14
39	183	20
33	238	13
38	163	26

Figure Click here to access/download;Table;Table S4.docx

Table S4: Results of PERMANOVA to partition distance matrices among sources of variation

	df	Sum of Squares	Mean Squares	F-Model	R^2	P value
Ecoregion	1	0.11516	0.115160	8.3852	0.14095	0.0001***
Elevation	1	0.02891	0.028910	2.1050	0.03538	0.0391*
Residuals	49	0.67296	0.013734		0.82367	
Total	51	0.81703				

Figure Click here to access/download;Table;Table S5.docx

Table S4: Wet weight (g) to insect lysis buffer volume (mL) ratios for Malaise trap bulk samples.

Wet Weight of Bulk Sample (g)	Insect Lysis Buffer Volume (mL)
<1.5	15
1.5-4.9	20
5.0-9.9	50
10.0-19.9	100
20.0-29.9	200
>30.0	250

Figure

Click here to access/download;Figure;Figure 1.pdf

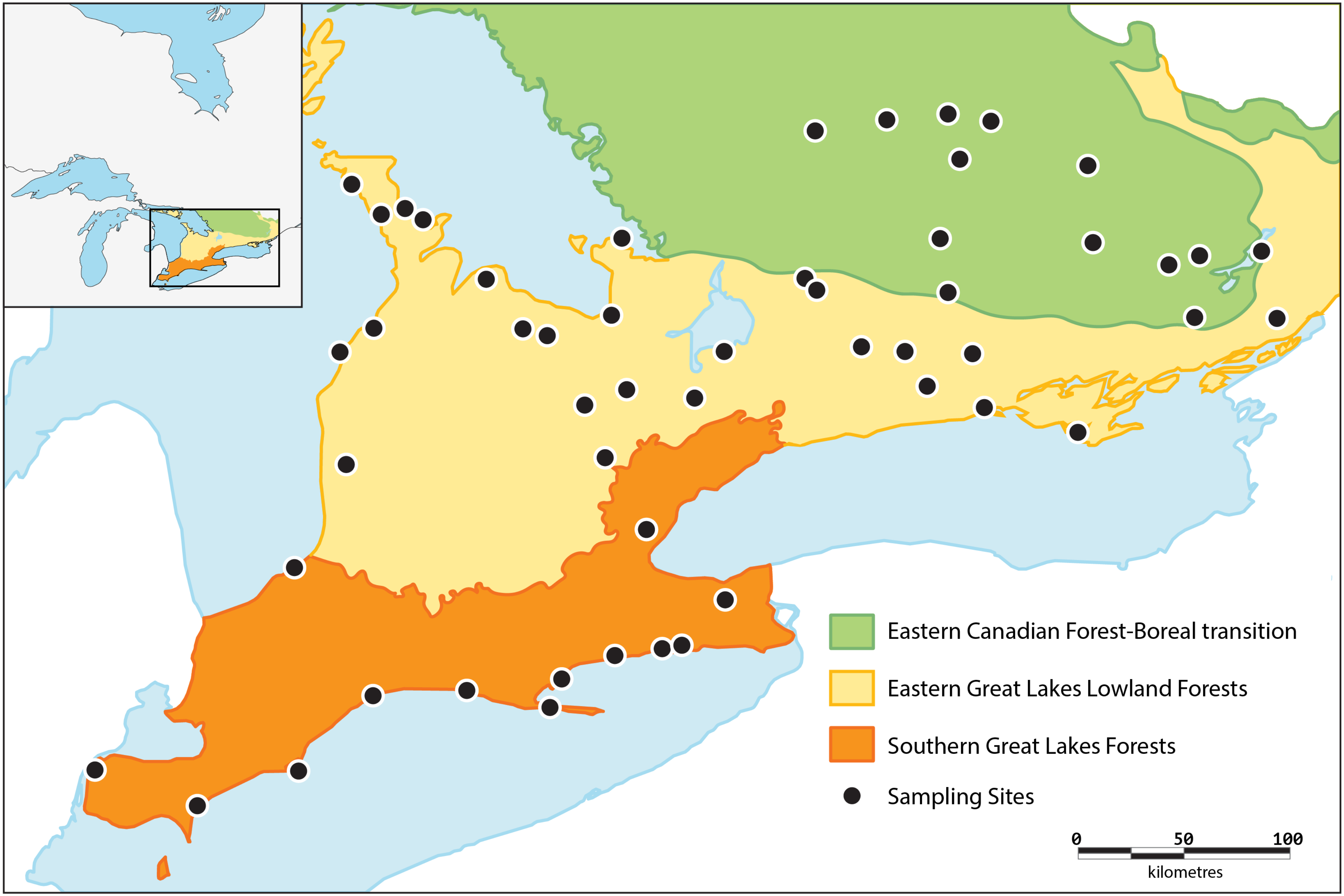

Figure

Click here to access/download;Figure;Figure 2.pdf

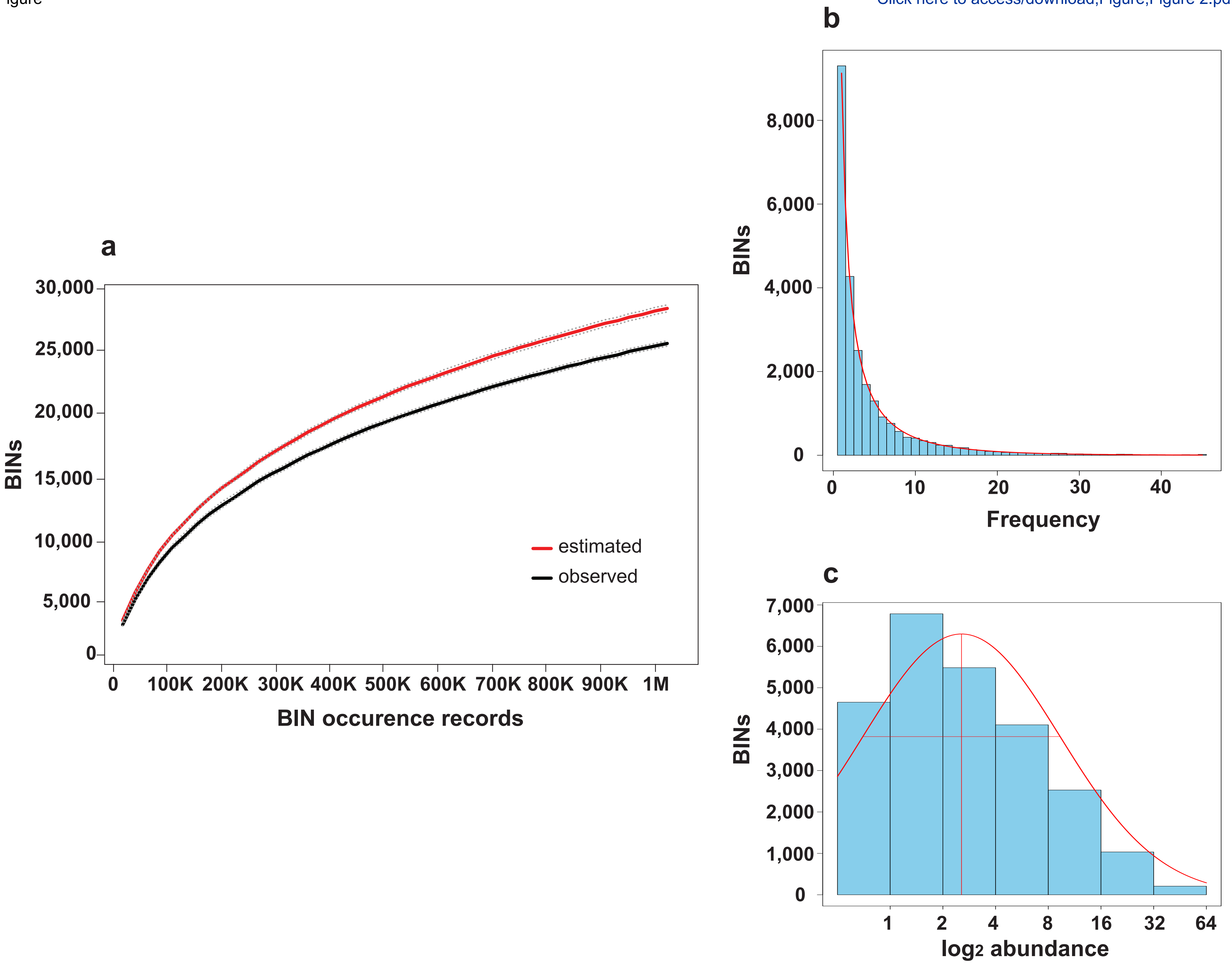

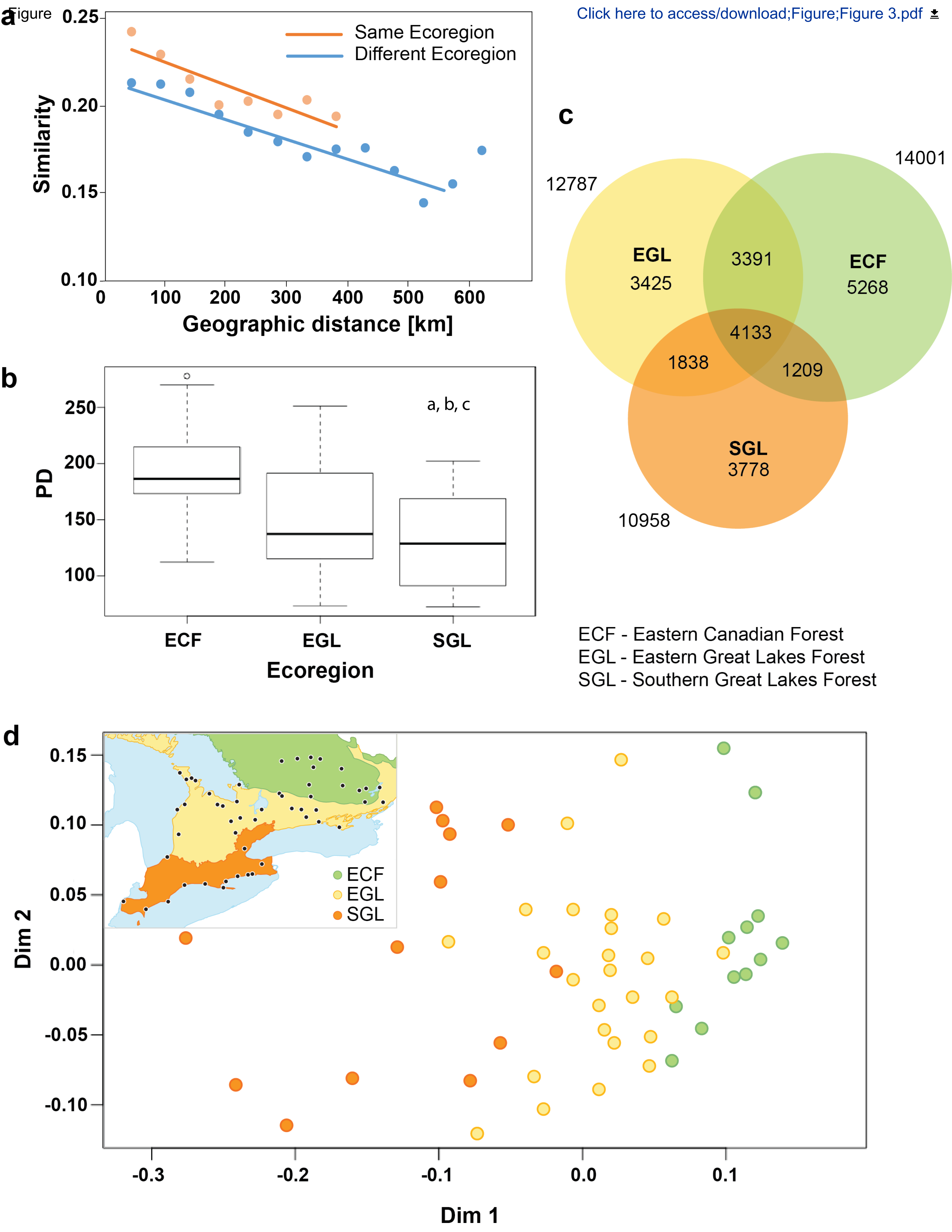
a
Same Ecoregion
Different Ecoregion
Similarity
0.25
0.20
0.15
0.10
0
100
200
300
400
500
600
Geographic distance [km]
b
PD
250
200
150
100
a, b, c
ECF
EGL
SGL
Ecoregion
c
12787
14001
10958
EGL
3425
3391
ECF
5268
4133
1838
1209
SGL
3778
ECF - Eastern Canadian Forest
EGL - Eastern Great Lakes Forest
SGL - Southern Great Lakes Forest
d
ECF
EGL
SGL
Dim 2
0.15
0.10
0.05
0.00
-0.05
-0.10
-0.3
-0.2
-0.1
0.0
0.1
Dim 1

Figure Click here to access/download;Figure;Figure 4.pdf

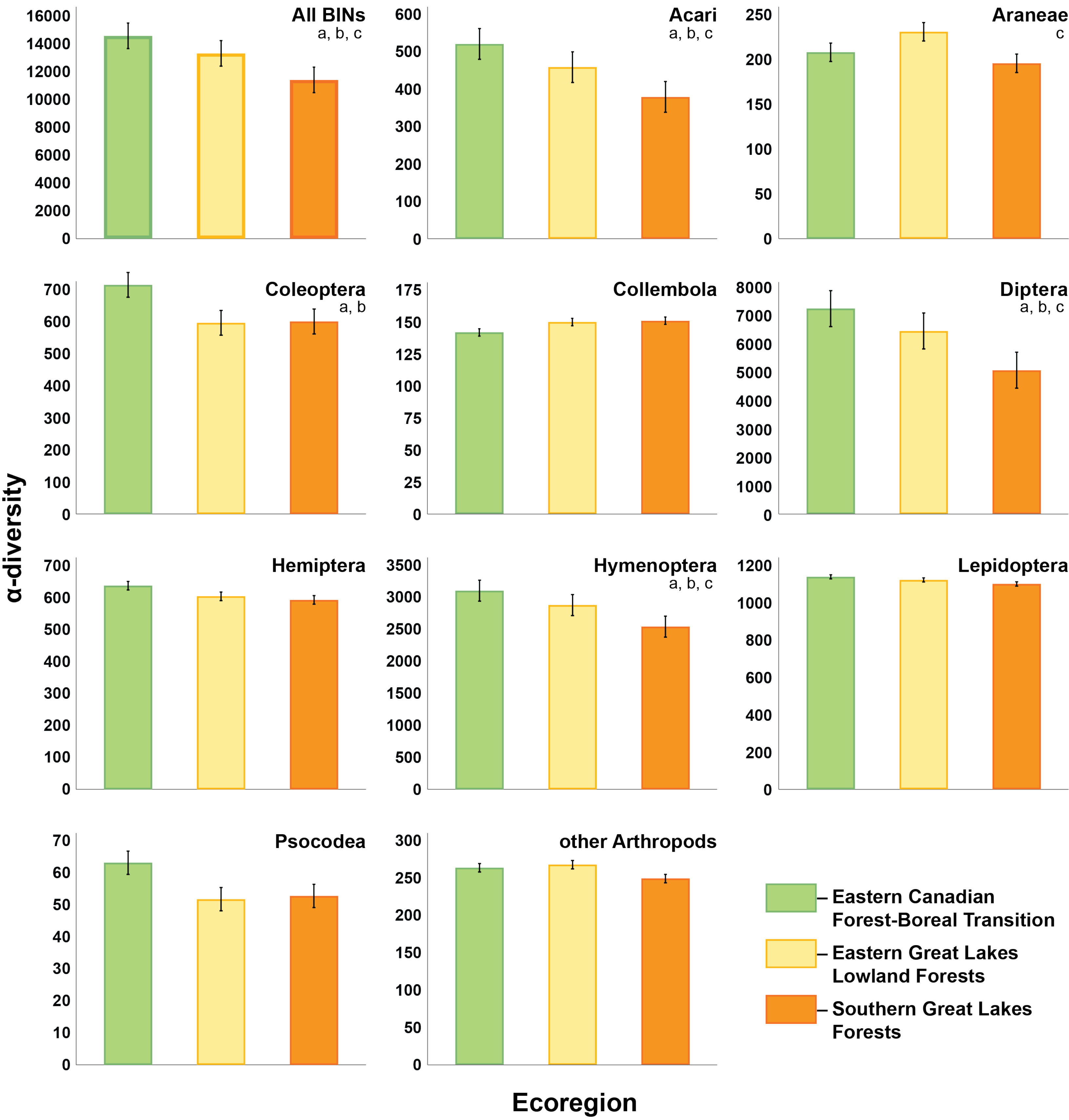

Figure

Click here to access/download;Figure;Figure 5.pdf

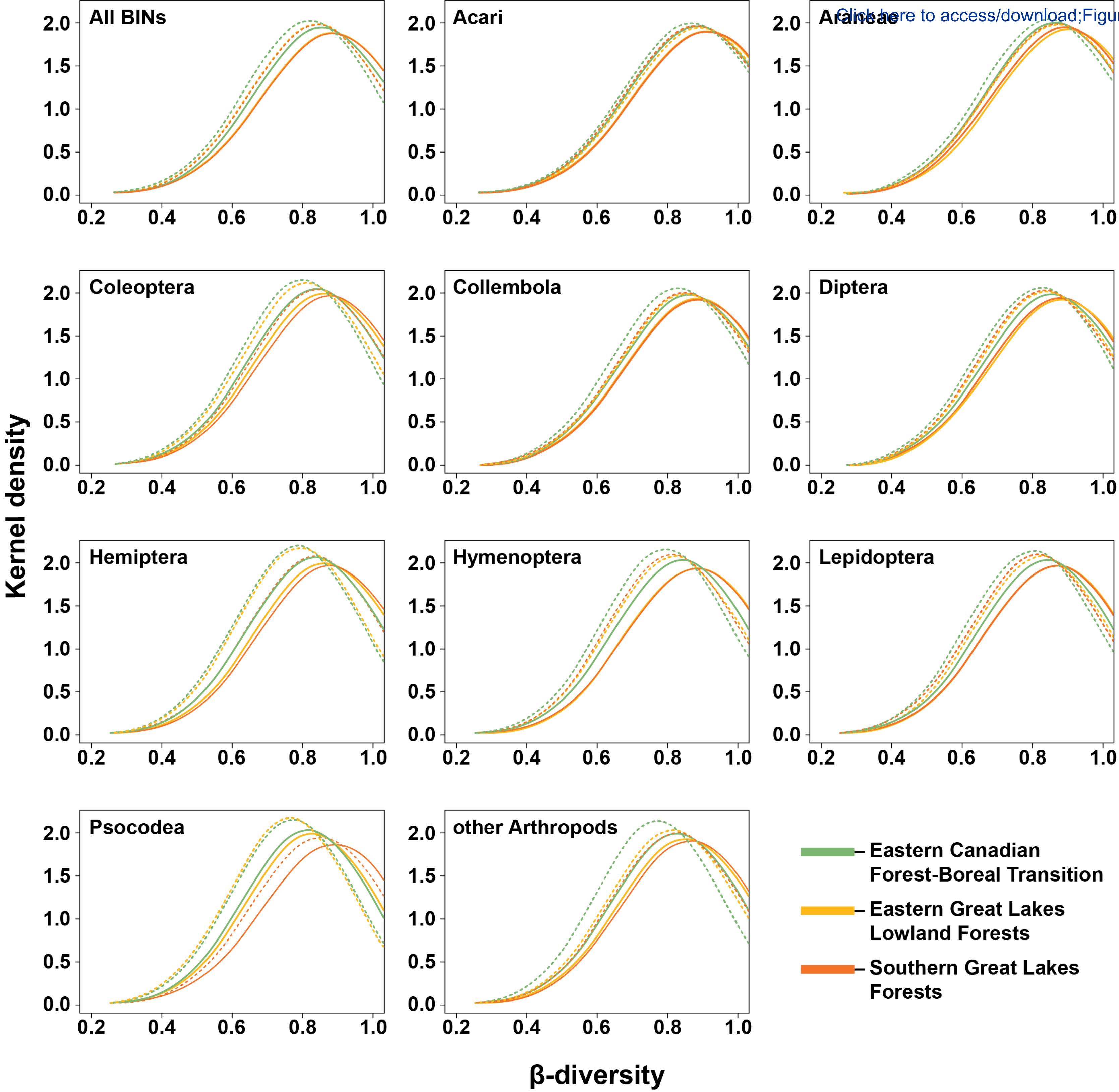

Click here to access/download;Figure;Figure S1.pdf

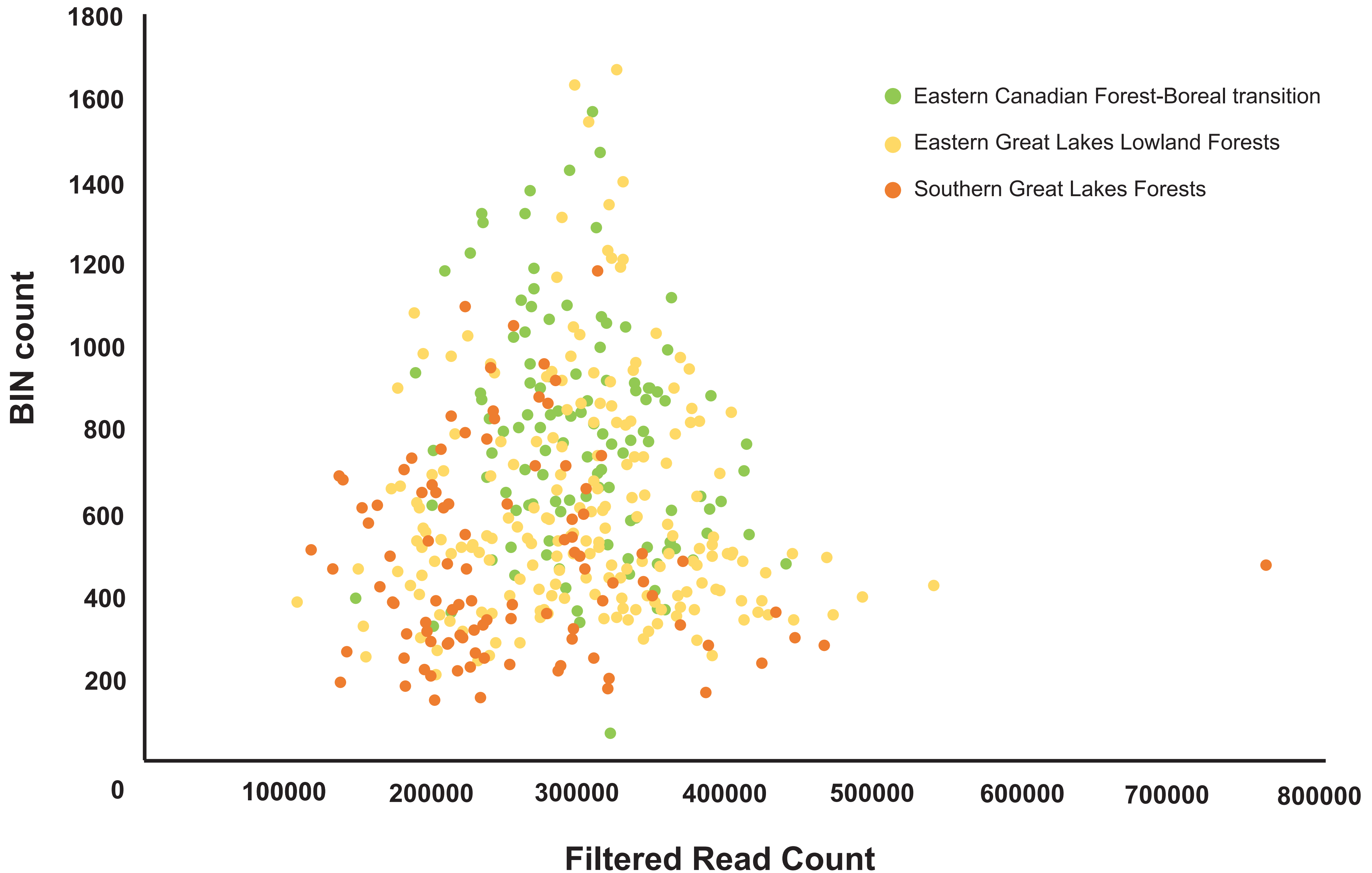

Figure
Click here to access/download;Figure;Figure S2.pdf

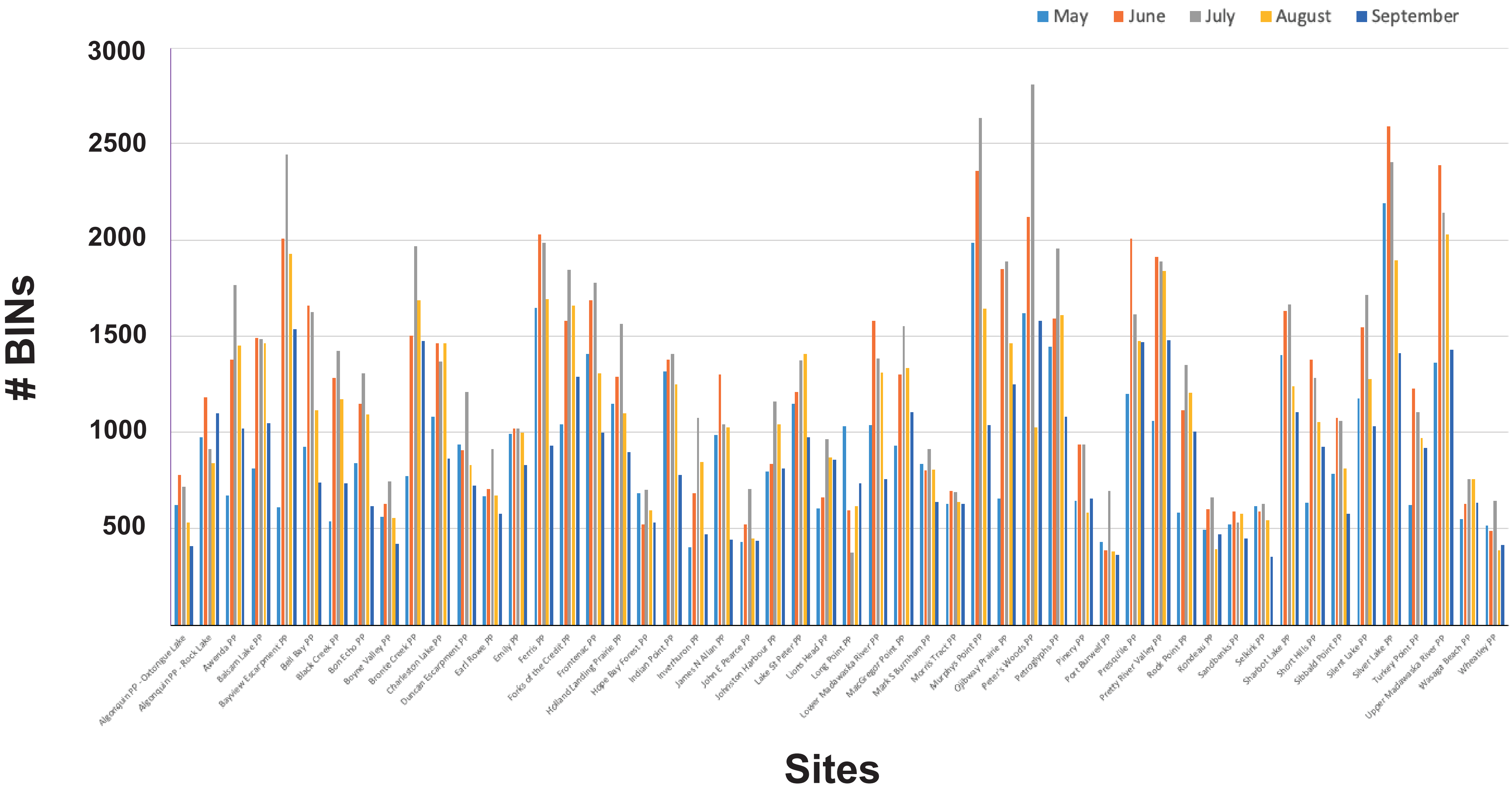

Click here to access/download;Figure;Figure S3.pdf

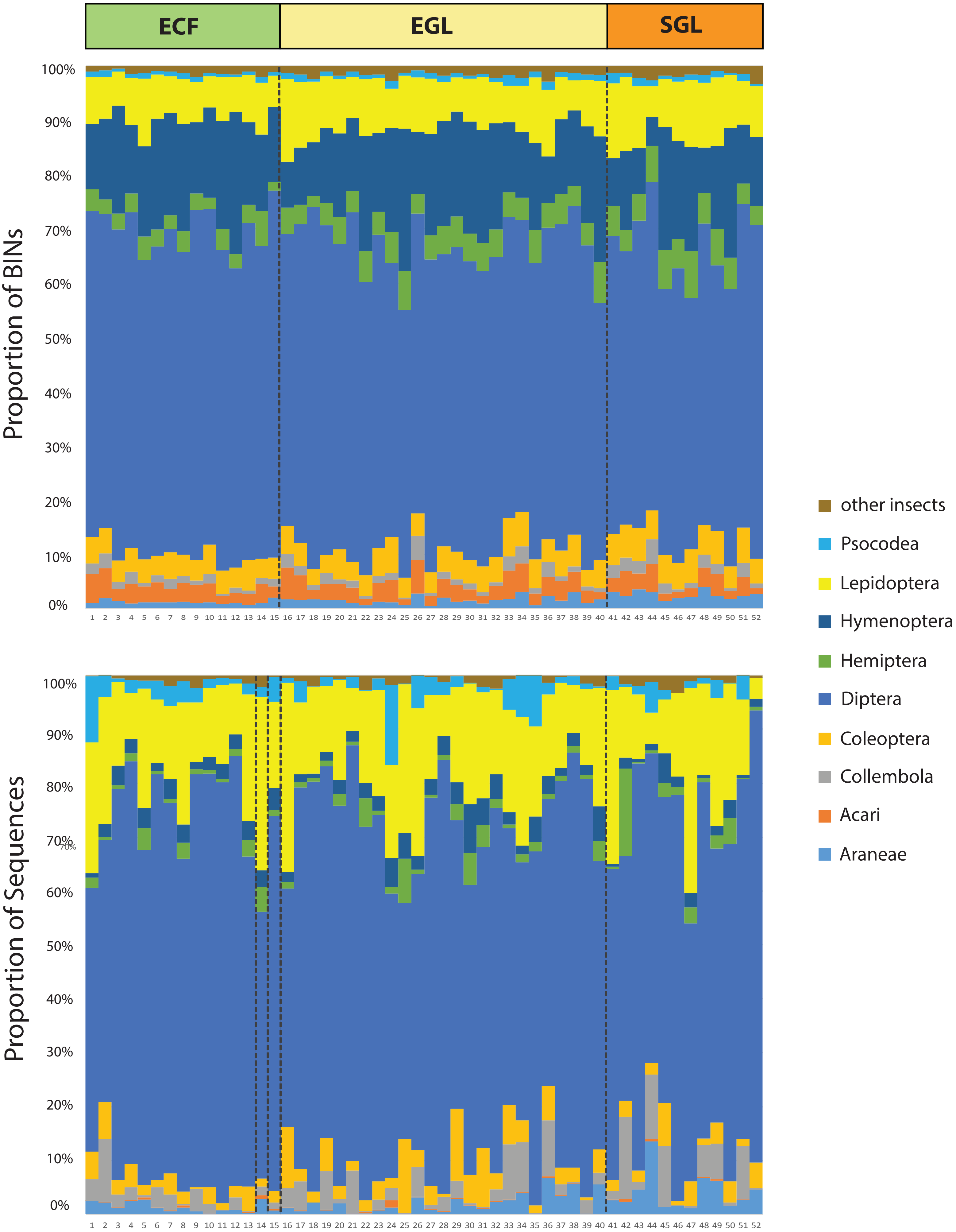

Personal Cover

Click here to access/download;Personal Cover;Reviewer reports answered.docx

Reviewer reports:

Reviewer #1: The manuscript is very well written and a great contribution to the field. However some analytical aspects need to be better described. Also, it would be great the authors provide their R-script in the supplementary material. Below my comments.

Line 166: R2 = 0.035 is very low, it needs to be better considered.

We changed the text so that it becomes clear that this is very low.

Lines 168-171: The alpha diversity comparison was based just in visual inspection or any test was made?

It was properly tested as described in the methods and the figure 4 caption (Kruskal-Wallis followed by Mann-Whitney post-hoc)

Lines 173-176: There was any test to significance? It need to be reported.

No, we added more explanation how to read this in the Figure 5 caption - usually for these plots significant differences between entities are detected when the peaks of the density plots do not overlap.

Lines 213-219: It is a nice discussion about local versus regional diversity, but very speculative, need at least some citations to support it.

It is speculative because not much is known about these traps despite a rather long history of use. We added two citations for more supportive context.

Lines 357-358: It reduce background contamination, you never can remove all.

we changed the text to indicate that it was done to reduce not remove all background contamination

Lines 365-367: How the distances were controlled, any analysis of spatial correlation?

This part of the sentence was misleading. We did not control for distances but (as shown in Figue 3a) looked at between vs across ecoregion border similarities across a range of distances between sites. We removed this part.

Lines 367_370: The NMDS was with abundance or presence/absence data? If it was abundance, any correction was applied?

It was done with presence/absence data. This is now clearer in the manuscript.

Lines 374-376: How the author checked the quality of the tree as it was made with very short fragment? the blackbox toll set all parameters on the model?

We added a sentence to mention the model that is used in RAxML Blackboxs (GTRCAT). It also uses a build-in bootstrap routine.

Line 382: Was there any correction to BINs table? Rarefaction, Shannon entropy? It is very necessary to metabarcoding data. Also why just BIN richness, other diversity measures may be included as Shannon or Fisher diversity on phyloseq, or the effective number of BINs with entropart.

Our intention was to show alpha-diversity for which BIN (MOTU) richness is perfectly sufficient. We also lacked some data (also as the result of the overall sample size) for proper use of phyloseq. The necessity of corrections (rarefaction, shannon entropy) is still debated especially with respect to which method to use. The study goal wasn't so much a contribution to this important aspect but we plan to use these and other data to further explore proper correction models.

Figure 1 needs a reference o Canada to better understand where the region is.

We changed the figure to include a small reference map of a part of Canada.

Reviewer #2: Steinke et al. used a metebarcoding method to investigate the species compositions for 410 insect bulk samples collected in 3 ecoregions. The manuscript is well written, all the materials and methods were clearly described, I think the manuscript should be accepted for publication after addressing several minor issues as follows:

1. Line 126, as Ion torrent is not widely used nowadays, may the authors add some words regarding its sequencing length, error rate, throughput et al.
this has been done
2. Please unify the format of chao 1 (or chao-1).
this has been done
3. A rarefaction curve for each sample may need to check whether the species diversity is well represented by its raw reads.
We don't think that this would add anything useful to the study especially given the large quantity of filtered reads (as a consequence of using the IonTorrent System). For instance the presence of chimaeras would skew any plot. Aside from that, the study consists of 410 samples. Plots for each one of these would be way too many for any reader to deal with.
4. Line 187 - 191. This BIN number inflation may also boil down to sequence errors introduced during PCR amplification or sequencing.
we added a line to include these potential sources as well
5. Please pay attention to the citation format. For example, in line 202, reference # 40 should follow the first author's name.
this has been fixed
6. Line 226 - 227, please add some words to better explain the speculation of "passively transported by wind".
this has been done

Reviewer #3: General comments
Steinke et al used DNA metabarcoding of malaise trap samples from 52 protected areas spanning three Canadian ecoregions to assess the spatial patterns of arthropod biodiversity. The research question is relevant and interesting, the study is well designed, data collected are comprehensive, and manuscript is well written and easy to follow. I enjoyed reading it and would like to thank the authors for such a great contribution. My main concern is that the temporal aspect of the study was not explored even though it was mentioned as part of the research objective.
Specific comments
L60-62: These reductions are not only for abundance but also for diversity, at least based on the fourth reference cited here. I would therefore include "diversity" or "richness" in this statement.
this has been done

L63 & L105: The authors use biosurveillance in some places in the text and bio-surveillance in others. Isn't it better to stick to the same spelling all through, at least for consistency?
we corrected this
L132: I am a bit confused here. Are these "Analyses" or "Results"? The whole subsection from L133-L176 read like results to me.
The manuscript format provided by GigaScience didn't suggest a Results section but rather Analyses. We do agree with the reviewer and made the change in the hopes that it is acceptable to the publisher
L329: "of" omitted! Five samples were available from each of the other 22 sites...
we corrected this
L332-334: The first "following" in this sentence can be either omitted or that part of the sentence completed using "manufacturer's instructions"
we corrected this
L345-346: "Reads were trimmed 30 bp from their 5' terminus with a set trim length of 450 bp". Perhaps this needs more clarification. The amplified length was 463 bp, trimming 30 bp gives 433 bp. How then can set trim length be 450 bp?
In fact, IonTorrent instruments produce reads of varying length, some of which are longer than the targeted amplicon size. mBRAVE does the front trim first and the does length filtering based on the trim length setting. This allowed some sequences (>433 and <450) through. Most of those are filtered out later in the routine because these are often chimaeras or other errors. We change the sentence slightly to indicate the the trim length setting function on mBRAVE is a length filter.
L348-349: What was the criterion for using "at least three reads matched an OTU in the reference database"? I mean why not at least two or at least four reads? If this was arbitrary please clarify.
We had to correct this number to five (original was incorrect) but the number is indeed arbitrary resulting from experience with other datasets using mBRAVE and the IonTorrent platform. We added text to explain that.
L349-350: Same question as above, why use "a minimum of five reads per cluster"? It would be nice to indicate if any benchmarking was applied a priori or if this was set arbitrarily.
We added text to explain that we used benchmarking (unpublished)
L346-349: Since the authors were mostly interested in arthropods, were reads that matched sequences from bacteria (SYS-CRLBACTERIA), chordates (SYS-CRLCHORDATA) and non-arthropod invertebrates (SYS CRLNONARTHINVERT) discarded or retained? This should be mentioned here and estimates of the number of reads, BINs or OTUs matching each of these categories should be provided.
We added the numbers for each category to table S3 and clarified that all non-arthropod reads were discarded for the remainder of the analysis.
L149-153: These are interesting results. It would be nice to present them graphically, at least in the supplementary. The aim of the study was "to assess spatial and temporal variation in species richness and diversity in arthropod communities from 52 protected areas spanning three Canadian ecoregions" but the temporal aspect of the study was not fully explored. Although it is stated that "trap catches were harvested every second week from early May

through September", this information has not be used in the analysis. Should the aim of the study be redefined and restricted to just spatial patterns then?

We generated a new supplementary Figure S2 to show the temporal variation of species richness for all sites. We also added a reference to it in the discussion section that discusses temporal variation.

L152-153: Without any table or figure to support these results, why not provide the actual number or proportion or percentage of BINs for each arthropod order in the text?

We generated a new supplementary Figure S3 to rectify this.

L157-158: Please add some symbols (e.g. asterisks *, **, ***or alphabet a, b, c) to Figure 3b to represent significant differences. Looking at the present figure without referring to the text does not tell the reader if the differences are significant. Besides, the authors only report a single p value ($p < 0.003$) which probably means at least one of the groups is different from the others but failed to report the pairwise multiple comparison tests that tell the reader which pairs or groups (e.g. ECF vs EGL, ECF vs SGL, EGL vs SGL) are significantly different.

We changed the text and added all p values for the three comparisons that were all significant. The figure was amended with a,b,c to indicate the same.

L159: Are the patterns similar if you control for the total number of sites per ecoregion? For example, taking 12 sites per ecoregion and resampling them 100 or 1000 times, similar to the approach used for beta diversity. It could be that one site is driving this pattern, as shown in Figure 2b and reported in L141 "…with more than a third (9,301) found at only one site (Figure 2b)".

Actually, both Figure 2b and L141 refer to the fact that 9301 BINs were only obtained at one site but not all at the same site. It rather means each BIN occurs only at one of all 52 sites.

L164-166: Please provide the full PERMANOVA results in a table in the text or supplementary and reference it here. It is not clear what "decreased site elevation (R2 166 = 0.035, P = 0.03)" means.

We added this as table S5

L168-171: Do these patterns change or remain the same if the same number of sites per ecoregion is used? This needs to be tested given that one site (probably from ECF or EGL?) is disproportionate species-rich and SGL has the lowest number of sites.

Again, there isn't a single site that is disproportionate species-rich. This can be clearly seen in Figure S2. We are using the same number of sites for each ecoregion here. We added a better description to the methods section.

L173-176: What about levels of turnover across time? Were they any temporal trends in alpha and beta diversity? Was the temporal dropped from the study objective and why?

We included a new supplementary figure S2 showing alpha diversity over the season for all sites.

L221-223: Same question as above, were temporal changes in species composition considered? Which results, tables or figures point to this or how did the authors arrive at these statements?

We did not explore temporal differences in species composition. Some parts of the discussion were toned done a bit to reflect this.

Reviewer #4: This manuscript assesses the variation in arthropod communities in three ecoregions in Canada. The study is well done, and the sampling was very thorough with a big sampling effort. I only have minor comments. Specially I consider that the aim can be focused on the ecoregions instead of the feasibility of the method, as this has already been shown. In addition, it would be nice to have more details in certain sections in the data analyses and in the results. I have addressed these comments below.

-I am not sure why the title "Message in a bottle".

That's both a reference to the collecting bottle of a Malaise trap and a famous song of the 1980s. We would like to keep it.

-Line 65- Could you specify which indicator species have been targeted? Or cite studies that target those species?

We included a new reference (a review paper on indicator species selection and use)

- Line 96- Based on the limitations of the ecoregions, it is not clear why ecoregions are an obvious candidate.

We expanded the text to better explain this

-In line 104 seems that your aim is to demonstrate how feasible is to use metabarcoding for large-scale monitoring and that you use the ecoregions to prove that. However, showing the feasibility of this method for large-scale studies has already been done (e.g. Svenningsen et al 2021, Detecting flying insects using car nets and DNA metabarcoding; Bush et al 2020, DNA metabarcoding reveals metacommunity dynamics in a threatened boreal wetland wilderness). I suggest keeping it focused on the need to apply this method in different ecoregions.

We changed the text to reflect this

-In the Data description section, you mention that you examined phylogenetic diversity, but in the Analyses section you vaguely mention it. The phylogenetic diversity findings are discussed later on, but it is difficult to follow the discussion when the results were not presented previously. In addition, the authors use the findings in phylogenetic diversity to support the idea of a structure in the ecoregions, so I suggest making more emphasis in this in the results section.

We do mention phylogenetic diversity in the Analyses/Results section (158-160 and Figure 3b). In fact. we describe the results of pairwise PD comparisons (Kruskal-Wallis and Dunn's posthoc tests) to show that there are differences in composition of ecoregions.

-Line 189. I agree that the higher number of BINs could be due to eDNA, but couldn't another reason be that the BINs were oversplit during data analysis?

we added a line to include sequence error as another potential source (also requested by reviewer 2)

-Line 215-217. Has this been found previously in other studies using Malaise trap? If so, please reference to those findings.

We don't think that it has been reported as such.

-Line222- This is a brief discussion about temporal turnover. However, these results are not presented previously, or at least not clearly enough.

we have added a new supplementary figure and text to rectify this (see responses to reviewer 3)

-Line 266-267- Yes, you showed compositional shifts using metabarcoding in bulk arthropod samples, but the way this sentence is structured it sounds like you are the first to show this. Compositional shifts in arthropods have been shown previously in other studies using metabarcoding.

we toned down this sentence accordingly

-Line 321- Did you have negative PCR controls? In line 326 you mention negative controls, but I assume you refer to the extraction negative controls.

no, we didn't use negative PCR controls, the one we refer to is indeed an extraction control, we clarified that in the text

-Line 340- It is not clear why you queried the data against a bacterial library.

we added some text to explain that we screened for contamination by bacterial endosymbionts such as Wolbachia

-Line 348- What was the reason for choosing "at least three reads"? and the same for line 350 where you cluster sequences with a minimum of 5 reads per cluster.

see response to reviewer 3 - we clarified this in the text

-Line 357- If you see tag switching in your negative controls that means that most likely you have it in the rest of the data. How did you ensure that the rest of the data did not have that? You may have tags switching in sequences not found in the negative controls but found in your samples.

That is possible, which is why we said "reduces the effects of tag switching" - the use of negative controls that are sequenced has been recommended. Other options such as the use of matching tags or PCR replicates were not used. We only had technical replicates. Some also list the careful handling of tagged amplicons which is a given for such studies.

-Line 369- As you used the Bray-Curtis index in this metabarcoding data, did you convert your data to presence/absence? It is known that for metabarcoding data the use of read numbers for community analysis is not adequate (see Nichols et al 2018 "Minimizing polymerase biases in metabarcoding").

Yes we did convert to p/a and used the resulting matrices for further analysis. This is has been included in the text.
