# Supplementary material for: Message in a Bottle—Metabarcoding enables biodiversity comparisons across ecoregions
Source: Gigascience. 2022 Apr 28;11:giac040. doi: 10.1093/gigascience/giac040 (PMC9049109; doi:10.1093/gigascience/giac040)
Supplement: giac040_Supplemental_Figures_and_Tables [file giac040_supplemental_figures_and_tables.zip › Table S4.docx]

**Table S4**: Results of PERMANOVA to partition distance matrices among sources of variation

|  | df | Sum of Squares | Mean Squares | F-Model | R^2^ | P value |
| --- | --- | --- | --- | --- | --- | --- |
| Ecoregion | 1 | 0.11516 | 0.115160 | 8.3852 | 0.14095 | 0.0001*** |
| Elevation | 1 | 0.02891 | 0.028910 | 2.1050 | 0.03538 | 0.0391* |
| Residuals | 49 | 0.67296 | 0.013734 |  | 0.82367 |  |
| Total | 51 | 0.81703 |  |  |  |  |
