# Supplementary material for: Message in a Bottle—Metabarcoding enables biodiversity comparisons across ecoregions
Source: Gigascience. 2022 Apr 28;11:giac040. doi: 10.1093/gigascience/giac040 (PMC9049109; doi:10.1093/gigascience/giac040)
Supplement: giac040_Supplemental_Figures_and_Tables [file giac040_supplemental_figures_and_tables.zip › Table S5.docx]

**Table S4:** Wet weight (g) to insect lysis buffer volume (mL) ratios for Malaise trap bulk samples.

| **Wet Weight of Bulk Sample (g)** | **Insect Lysis Buffer Volume (mL)** |
| --- | --- |
| <1.5 | 15 |
| 1.5-4.9 | 20 |
| 5.0-9.9 | 50 |
| 10.0-19.9 | 100 |
| 20.0-29.9 | 200 |
| >30.0 | 250 |
