# Supplementary figures and images for: Message in a Bottle—Metabarcoding enables biodiversity comparisons across ecoregions
Source: Gigascience. 2022 Apr 28;11:giac040. doi: 10.1093/gigascience/giac040 (PMC9049109; doi:10.1093/gigascience/giac040)

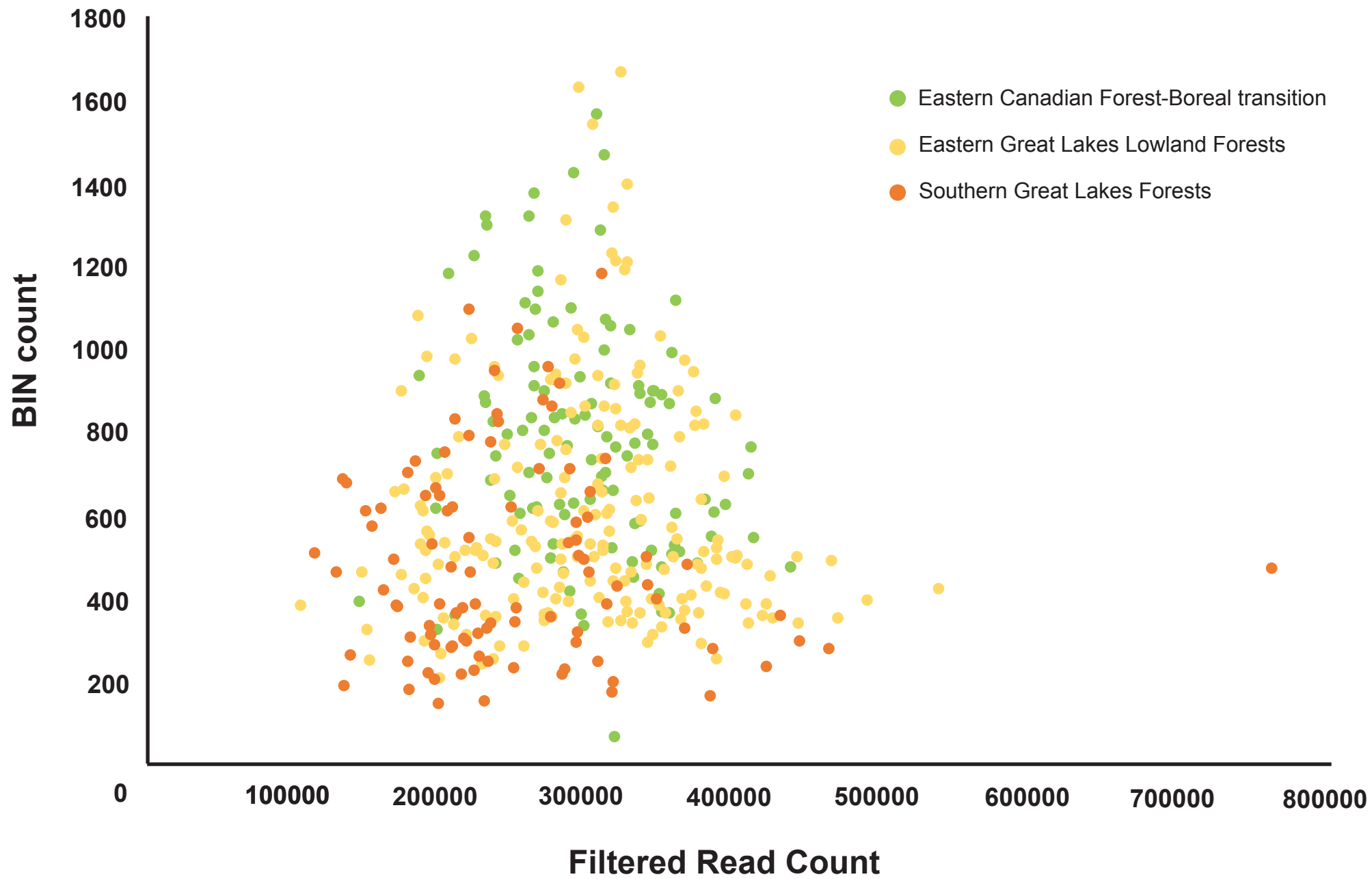

Supplement: giac040_Supplemental_Figures_and_Tables [file giac040_supplemental_figures_and_tables.zip › Figure S1.pdf]

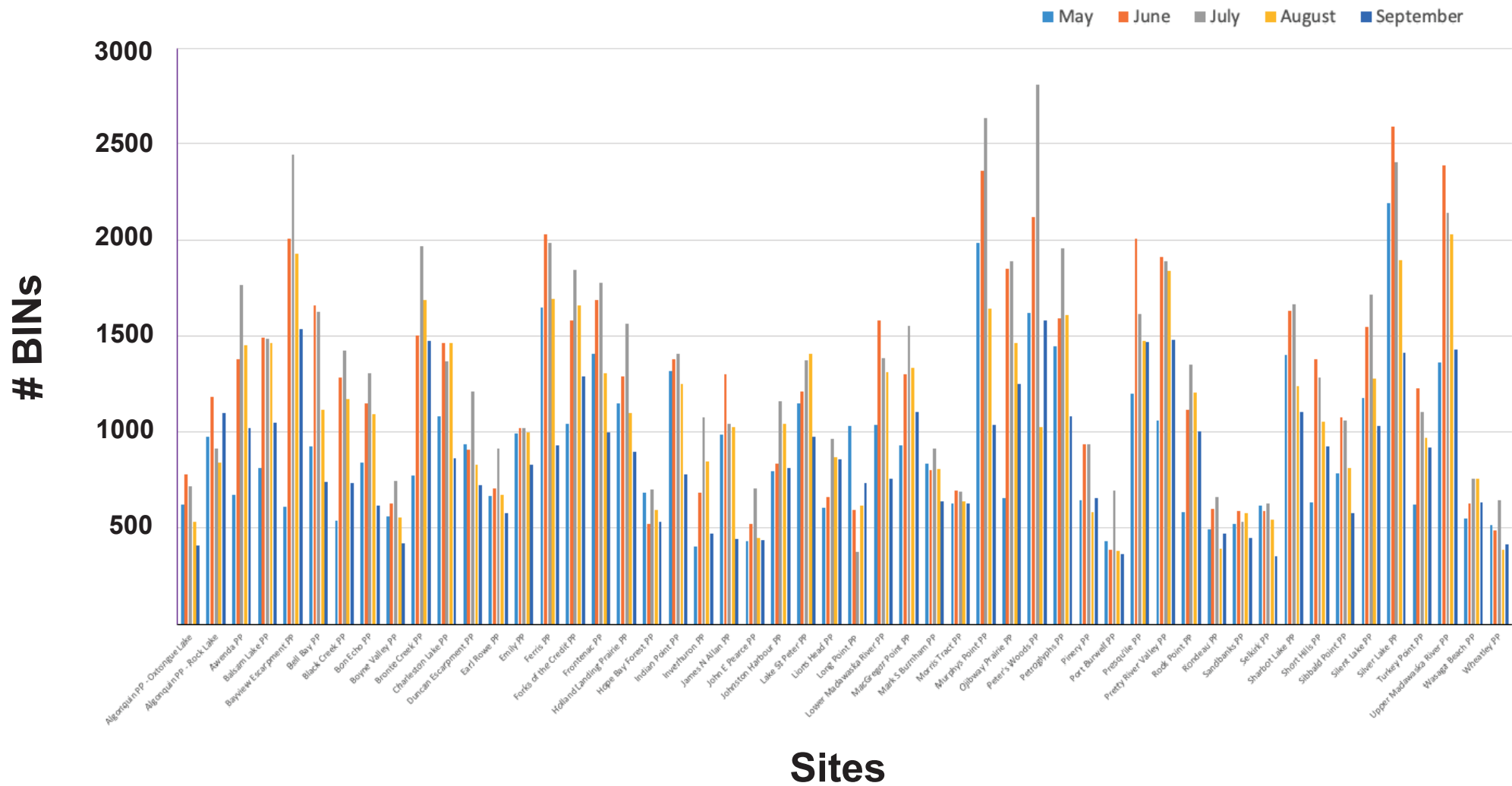

Supplement: giac040_Supplemental_Figures_and_Tables [file giac040_supplemental_figures_and_tables.zip › Figure S2.pdf]

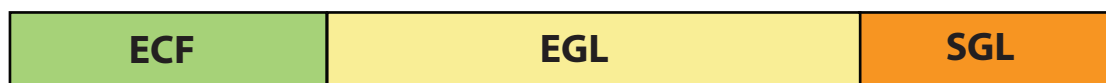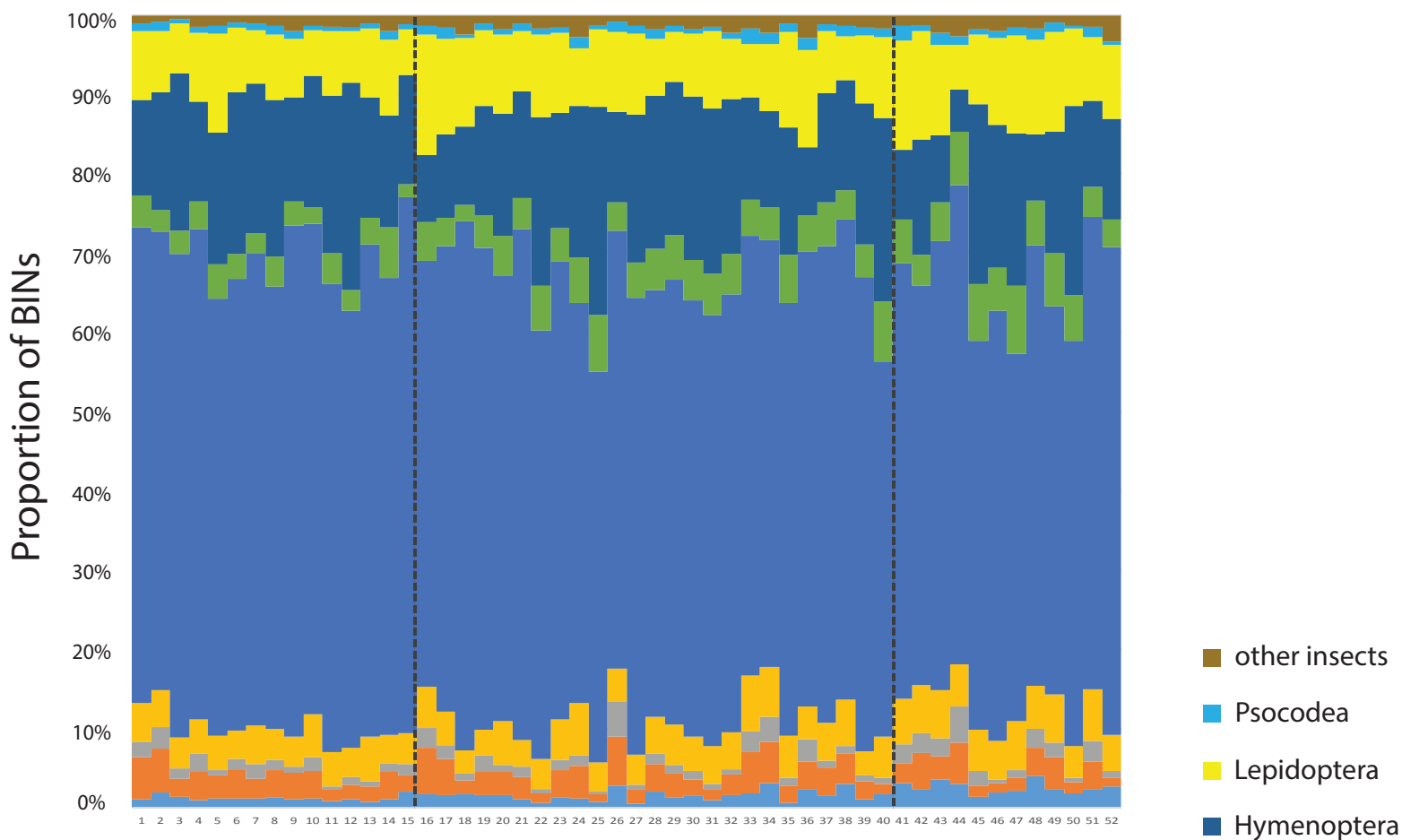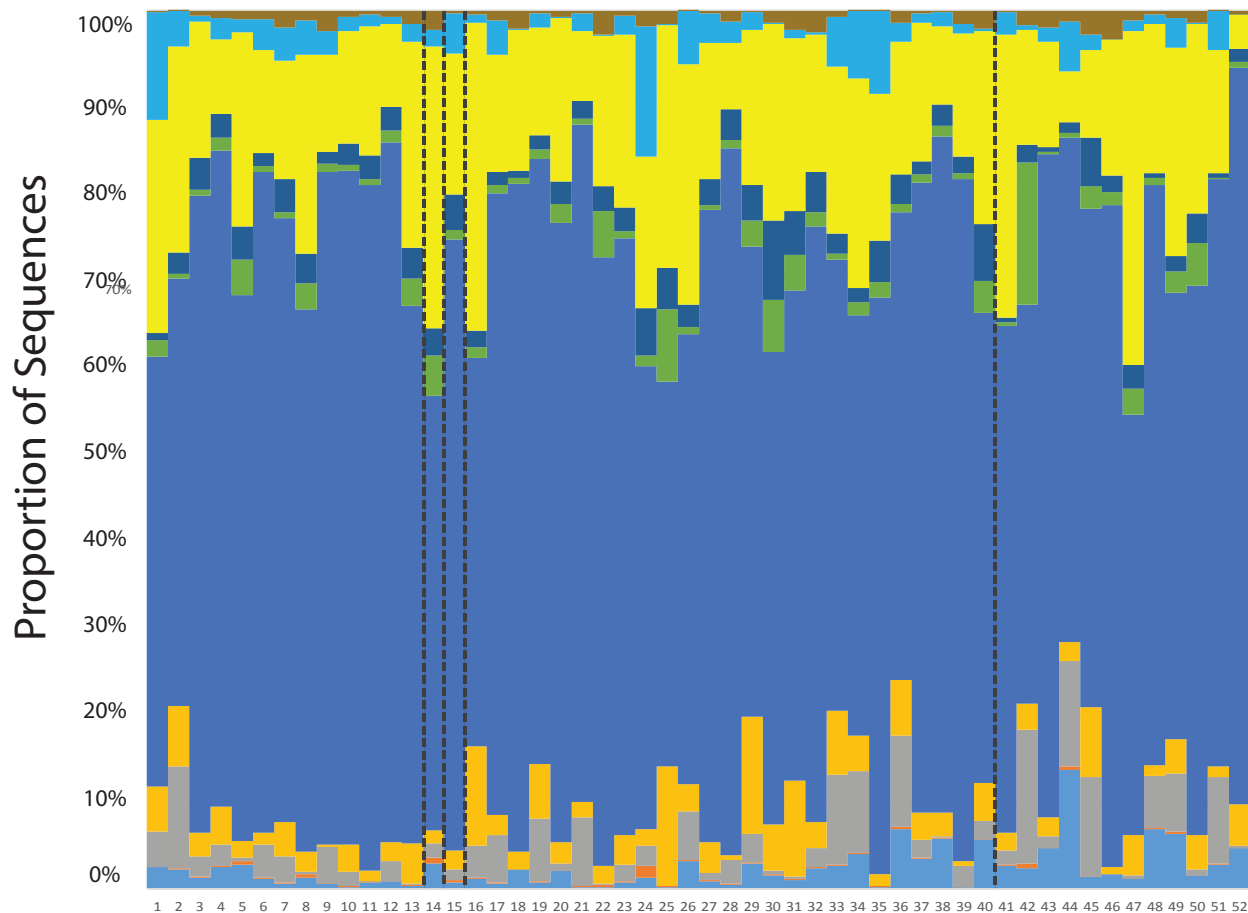

Supplement: giac040_Supplemental_Figures_and_Tables [file giac040_supplemental_figures_and_tables.zip › Figure S3.pdf]
