# Supplementary material for: Message in a Bottle—Metabarcoding enables biodiversity comparisons across ecoregions
Source: Gigascience. 2022 Apr 28;11:giac040. doi: 10.1093/gigascience/giac040 (PMC9049109; doi:10.1093/gigascience/giac040)
Supplement: giac040_Response_to_Reviewer_Comments_Original_Submission [file giac040_response_to_reviewer_comments_original_submission.pdf]

Author's Response To Reviewer Comments

Close

Reviewer reports:
Reviewer #1: The manuscript is very well written and a great contribution to the field. However some analytical aspects need to be better described. Also, it would be great the authors provide their R-script in the supplementary material. Below my comments.

Line 166: R2 = 0.035 is very low, it needs to be better considered.
We changed the text so that it becomes clear that this is very low.
Lines 168-171: The alpha diversity comparison was based just in visual inspection or any test was made?
It was properly tested as described in the methods and the figure 4 caption (Kruskal-Wallis followed by Mann-Whitney post-hoc)
Lines 173-176: There was any test to significance? It need to be reported.
No, we added more explanation how to read this in the Figure 5 caption - usually for these plots significant differences between entities are detected when the peaks of the density plots do not overlap.
Lines 213-219: It is a nice discussion about local versus regional diversity, but very speculative, need at least some citations to support it.
It is speculative because not much is known about these traps despite a rather long history of use. We added two citations for more supportive context.
Lines 357-358: It reduce background contamination, you never can remove all.
we changed the text to indicate that it was done to reduce not remove all background contamination
Lines 365-367: How the distances were controlled, any analysis of spatial correlation?
This part of the sentence was misleading. We did not control for distances but (as shown in Figue 3a) looked at between vs across ecoregion border similarities across a range of distances between sites. We removed this part.
Lines 367_370: The NMDS was with abundance or presence/absence data? If it was abundance, any correction was applied?
It was done with presence/absence data. This is now clearer in the manuscript.
Lines 374-376: How the author checked the quality of the tree as it was made with very short fragment? the blackbox toll set all parameters on the model?
We added a sentence to mention the model that is used in RAxML Blackboxs (GTRCAT). It also uses a build-in bootstrap routine.
Line 382: Was there any correction to BINs table? Rarefaction, Shannon entropy? It is very necessary to metabarcoding data. Also why just BIN richness, other diversity measures may be included as Shannon or Fisher diversity on phyloseq, or the effective number of BINs with entropart.
Our intention was to show alpha-diversity for which BIN (MOTU) richness is perfectly sufficient. We also lacked some data (also as the result of the overall sample size) for proper use of phyloseq. The necessity of corrections (rarefaction, shannon entropy) is still debated especially with respect to which method to use. The study goal wasn't so much a contribution to this important aspect but we plan to use these and other data to further explore proper correction models.
Figure 1 needs a reference o Canada to better understand where the region is.
We changed the figure to include a small reference map of a part of Canada.

Reviewer #2: Steinke et al. used a metebarcoding method to investigate the species compositions for 410 insect bulk samples collected in 3 ecoregions. The manuscript is well written, all the materials and methods were clearly described, I think the manuscript should be accepted for publication after addressing several minor issues as follows:

1. Line 126, as Ion torrent is not widely used nowadays, may the authors add some words regarding its sequencing length, error rate, throughput et al.
this has been done
2. Please unify the format of chao 1 (or chao-1).
this has been done

3. A rarefaction curve for each sample may need to check whether the species diversity is well represented by its raw reads.
We don't think that this would add anything useful to the study especially given the large quantity of filtered reads (as a consequence of using the IonTorrent System). For instance the presence of chimaeras would skew any plot. Aside from that, the study consists of 410 samples. Plots for each one of these would be way too many for any reader to deal with.
4. Line 187 - 191. This BIN number inflation may also boil down to sequence errors introduced during PCR amplification or sequencing.
we added a line to include these potential sources as well
5. Please pay attention to the citation format. For example, in line 202, reference # 40 should follow the first author's name.
this has been fixed
6. Line 226 - 227, please add some words to better explain the speculation of "passively transported by wind".
this has been done

Reviewer #3: General comments
Steinke et al used DNA metabarcoding of malaise trap samples from 52 protected areas spanning three Canadian ecoregions to assess the spatial patterns of arthropod biodiversity. The research question is relevant and interesting, the study is well designed, data collected are comprehensive, and manuscript is well written and easy to follow. I enjoyed reading it and would like to thank the authors for such a great contribution. My main concern is that the temporal aspect of the study was not explored even though it was mentioned as part of the research objective.
Specific comments
L60-62: These reductions are not only for abundance but also for diversity, at least based on the fourth reference cited here. I would therefore include "diversity" or "richness" in this statement.
this has been done
L63 & L105: The authors use biosurveillance in some places in the text and bio-surveillance in others. Isn't it better to stick to the same spelling all through, at least for consistency?
we corrected this
L132: I am a bit confused here. Are these "Analyses" or "Results"? The whole subsection from L133-L176 read like results to me.
The manuscript format provided by GigaScience didn't suggest a Results section but rather Analyses. We do agree with the reviewer and made the change in the hopes that it is acceptable to the publisher
L329: "of" omitted! Five samples were available from each of the other 22 sites...
we corrected this
L332-334: The first "following" in this sentence can be either omitted or that part of the sentence completed using "manufacturer's instructions"
we corrected this
L345-346: "Reads were trimmed 30 bp from their 5' terminus with a set trim length of 450 bp". Perhaps this needs more clarification. The amplified length was 463 bp, trimming 30 bp gives 433 bp. How then can set trim length be 450 bp?
In fact, IonTorrent instruments produce reads of varying length, some of which are longer than the targeted amplicon size. mBRAVE does the front trim first and the does length filtering based on the trim length setting. This allowed some sequences (>433 and <450) through. Most of those are filtered out later in the routine because these are often chimaeras or other errors. We change the sentence slightly to indicate the the trim length setting function on mBRAVE is a length filter.
L348-349: What was the criterion for using "at least three reads matched an OTU in the reference database"? I mean why not at least two or at least four reads? If this was arbitrary please clarify.
We had to correct this number to five (original was incorrect) but the number is indeed arbitrary resulting from experience with other datasets using mBRAVE and the IonTorrent platform. We added text to explain that.
L349-350: Same question as above, why use "a minimum of five reads per cluster"? It would be nice to indicate if any benchmarking was applied a priori or if this was set arbitrarily.
We added text to explain that we used benchmarking (unpublished)
L346-349: Since the authors were mostly interested in arthropods, were reads that matched sequences from bacteria (SYS-CRLBACTERIA), chordates (SYS-CRLCHORDATA) and non-arthropod invertebrates (SYS CRLNONARTHINVERT) discarded or retained? This should be mentioned here and estimates of the number of reads, BINs or OTUs matching each of these categories should be provided.
We added the numbers for each category to table S3 and clarified that all non-arthropod reads were discarded for the remainder of the analysis.

L149-153: These are interesting results. It would be nice to present them graphically, at least in the supplementary. The aim of the study was "to assess spatial and temporal variation in species richness and diversity in arthropod communities from 52 protected areas spanning three Canadian ecoregions" but the temporal aspect of the study was not fully explored. Although it is stated that "trap catches were harvested every second week from early May through September", this information has not be used in the analysis. Should the aim of the study be redefined and restricted to just spatial patterns then?
We generated a new supplementary Figure S2 to show the temporal variation of species richness for all sites. We also added a reference to it in the discussion section that discusses temporal variation.
L152-153: Without any table or figure to support these results, why not provide the actual number or proportion or percentage of BINs for each arthropod order in the text?
We generated a new supplementary Figure S3 to rectify this.
L157-158: Please add some symbols (e.g. asterisks *, **, ***or alphabet a, b, c) to Figure 3b to represent significant differences. Looking at the present figure without referring to the text does not tell the reader if the differences are significant. Besides, the authors only report a single p value ($p < 0.003$) which probably means at least one of the groups is different from the others but failed to report the pairwise multiple comparison tests that tell the reader which pairs or groups (e.g. ECF vs EGL, ECF vs SGL, EGL vs SGL) are significantly different.
We changed the text and added all p values for the three comparisons that were all significant. The figure was amended with a,b,c to indicate the same.
L159: Are the patterns similar if you control for the total number of sites per ecoregion? For example, taking 12 sites per ecoregion and resampling them 100 or 1000 times, similar to the approach used for beta diversity. It could be that one site is driving this pattern, as shown in Figure 2b and reported in L141 "…with more than a third (9,301) found at only one site (Figure 2b)".
Actually, both Figure 2b and L141 refer to the fact that 9301 BINs were only obtained at one site but not all at the same site. It rather means each BIN occurs only at one of all 52 sites.
L164-166: Please provide the full PERMANOVA results in a table in the text or supplementary and reference it here. It is not clear what "decreased site elevation (R2 166 = 0.035, P = 0.03)" means.
We added this as table S5
L168-171: Do these patterns change or remain the same if the same number of sites per ecoregion is used? This needs to be tested given that one site (probably from ECF or EGL?) is disproportionate species-rich and SGL has the lowest number of sites.
Again, there isn't a single site that is disproportionate species-rich. This can be clearly seen in Figure S2. We are using the same number of sites for each ecoregion here. We added a better description to the methods section.
L173-176: What about levels of turnover across time? Were they any temporal trends in alpha and beta diversity? Was the temporal dropped from the study objective and why?
We included a new supplementary figure S2 showing alpha diversity over the season for all sites.
L221-223: Same question as above, were temporal changes in species composition considered? Which results, tables or figures point to this or how did the authors arrive at these statements?
We did not explore temporal differences in species composition. Some parts of the discussion were toned done a bit to reflect this.

Reviewer #4: This manuscript assesses the variation in arthropod communities in three ecoregions in Canada. The study is well done, and the sampling was very thorough with a big sampling effort. I only have minor comments. Specially I consider that the aim can be focused on the ecoregions instead of the feasibility of the method, as this has already been shown. In addition, it would be nice to have more details in certain sections in the data analyses and in the results. I have addressed these comments below.

-I am not sure why the title "Message in a bottle".
That's both a reference to the collecting bottle of a Malaise trap and a famous song of the 1980s. We would like to keep it.
-Line 65- Could you specify which indicator species have been targeted? Or cite studies that target those species?
We included a new reference (a review paper on indicator species selection and use)
- Line 96- Based on the limitations of the ecoregions, it is not clear why ecoregions are an obvious candidate.
We expanded the text to better explain this
-In line 104 seems that your aim is to demonstrate how feasible is to use metabarcoding for large-scale monitoring and that you use the ecoregions to prove that. However, showing the feasibility of this method for large-scale studies has already been done (e.g. Svenningsen et al 2021, Detecting flying

insects using car nets and DNA metabarcoding; Bush et al 2020, DNA metabarcoding reveals metacommunity dynamics in a threatened boreal wetland wilderness). I suggest keeping it focused on the need to apply this method in different ecoregions.
We changed the text to reflect this
-In the Data description section, you mention that you examined phylogenetic diversity, but in the Analyses section you vaguely mention it. The phylogenetic diversity findings are discussed later on, but it is difficult to follow the discussion when the results were not presented previously. In addition, the authors use the findings in phylogenetic diversity to support the idea of a structure in the ecoregions, so I suggest making more emphasis in this in the results section.
We do mention phylogenetic diversity in the Analyses/Results section (158-160 and Figure 3b). In fact. we describe the results of pairwise PD comparisons (Kruskal-Wallis and Dunn's posthoc tests) to show that there are differences in composition of ecoregions.
-Line 189. I agree that the higher number of BINs could be due to eDNA, but couldn't another reason be that the BINs were oversplit during data analysis?
we added a line to include sequence error as another potential source (also requested by reviewer 2)
-Line 215-217. Has this been found previously in other studies using Malaise trap? If so, please reference to those findings.
We don't think that it has been reported as such.
-Line222- This is a brief discussion about temporal turnover. However, these results are not presented previously, or at least not clearly enough.
we have added a new supplementary figure and text to rectify this (see responses to reviewer 3)
-Line 266-267- Yes, you showed compositional shifts using metabarcoding in bulk arthropod samples, but the way this sentence is structured it sounds like you are the first to show this. Compositional shifts in arthropods have been shown previously in other studies using metabarcoding.
we toned down this sentence accordingly
-Line 321- Did you have negative PCR controls? In line 326 you mention negative controls, but I assume you refer to the extraction negative controls.
no, we didn’t use negative PCR controls, the one we refer to is indeed an extraction control, we clarified that in the text
-Line 340- It is not clear why you queried the data against a bacterial library.
we added some text to explain that we screened for contamination by bacterial endosymbionts such as Wolbachia
-Line 348- What was the reason for choosing "at least three reads"? and the same for line 350 where you cluster sequences with a minimum of 5 reads per cluster.
see response to reviewer 3 - we clarified this in the text
-Line 357- If you see tag switching in your negative controls that means that most likely you have it in the rest of the data. How did you ensure that the rest of the data did not have that? You may have tags switching in sequences not found in the negative controls but found in your samples.
That is possible, which is why we said "reduces the effects of tag switching" - the use of negative controls that are sequenced has been recommended. Other options such as the use of matching tags or PCR replicates were not used. We only had technical replicates. Some also list the careful handling of tagged amplicons which is a given for such studies.
-Line 369- As you used the Bray-Curtis index in this metabarcoding data, did you convert your data to presence/absence? It is known that for metabarcoding data the use of read numbers for community analysis is not adequate (see Nichols et al 2018 "Minimizing polymerase biases in metabarcoding").
Yes we did convert to p/a and used the resulting matrices for further analysis. This is has been included in the text.

Close
