# Supplementary material for: Message in a Bottle—Metabarcoding enables biodiversity comparisons across ecoregions
Source: Gigascience. 2022 Apr 28;11:giac040. doi: 10.1093/gigascience/giac040 (PMC9049109; doi:10.1093/gigascience/giac040)
Supplement: giac040_Response_to_Reviewer_Comments_Revision_1 [file giac040_response_to_reviewer_comments_revision_1.pdf]

Author's Response To Reviewer Comments

Close

Reviewer reports:
Reviewer #1: The study is very well designed and written, with good and clear results. The author had considered all my comments from before, just some additional minor comments are below.

Lines 118-119: species (bin) richness is a measure of alpha diversity and change in community composition a measure of beta diversity.
We changed the text to remove this redundancy.

Lines 112-122: Malaise-traps collect some random local no flighting insects, while discuss that it represent local population is ok I miss the part of the random sampling and that the lack of such insects in the samples does not exactly mean the non-presence of these insects.
We modified the text to indicate that random sampling effects might also lead to this pattern.

Lines 243-246: The sentence "Although current metabarcoding protocols cannot estimate the abundance of each species" is not completely right. Currently many metabarcoding studies estimate abundance/biomass of species, some discussion of it is necessary. Some examples (among several others):

Elbrecht, V., & Leese, F. (2015). Can DNA-based ecosystem assessments quantify species abundance? Testing primer bias and biomass—sequence relationships with an innovative metabarcoding protocol. PloS one, 10(7), e0130324.

Thomas, A. C., Deagle, B. E., Eveson, J. P., Harsch, C. H., & Trites, A. W. (2016). Quantitative DNA metabarcoding: improved estimates of species proportional biomass using correction factors derived from control material. Molecular ecology resources, 16(3), 714-726.

Di Muri, C., Lawson Handley, L., Bean, C. W., Li, J., Peirson, G., Sellers, G. S., ... & Hänfling, B. (2020). Read counts from environmental DNA (eDNA) metabarcoding reflect fish abundance and biomass in drained ponds. Metabarcoding and Metagenomics, 4, 97-112.

Ershova, E. A., Wangensteen, O. S., Descoteaux, R., Barth-Jensen, C., & Præbel, K. (2021). Metabarcoding as a quantitative tool for estimating biodiversity and relative biomass of marine zooplankton. ICES Journal of Marine Science, 78(9), 3342-3355.

We added this to the text and integrated the recommended publications in context.

For the figures comparing the ecoregions, as they are just three I would recommend a color blind safe palette, orange, yellow and green is not nice.

We did check our colour palette using Color Oracle which checks for all common forms of colour blindness. In all instances the chosen colours were distinguishable.

Close
