# Supplementary material for: Message in a Bottle—Metabarcoding enables biodiversity comparisons across ecoregions
Source: Gigascience. 2022 Apr 28;11:giac040. doi: 10.1093/gigascience/giac040 (PMC9049109; doi:10.1093/gigascience/giac040)
Supplement: giac040_Reviewer_1_Report_Revision_1 — Camila Duarte Ritter -- 3/1/2022 Reviewed [file giac040_reviewer_1_report_revision_1.pdf]

Reviewer Report

Title: Message in a Bottle â€“ Metabarcoding Enables Biodiversity Comparisons Across Ecoregions

Version: Revision 1 **Date:** 3/1/2022

Reviewer name: Camila Duarte Ritter

Reviewer Comments to Author:

The study is very well designed and written, with good and clear results. The author had considered all my comments from before, just some additional minor comments are below.
Lines 118-119: species (bin) richness is a measure of alpha diversity and change in community composition a measure of beta diversity.
Lines 112-122: Malaise-traps collect some random local no flighting insects, while discuss that it represent local population is ok I miss the part of the random sampling and that the lack of such insects in the samples does not exactly mean the non-presence of these insects.
Lines 243-246: The sentence "Although current metabarcoding protocols cannot estimate the abundance of each species" is not completely right. Currently many metabarcoding studies estimate abundance/biomass of species, some discussion of it is necessary. Some examples (among several others):
Elbrecht, V., & Leese, F. (2015). Can DNA-based ecosystem assessments quantify species abundance? Testing primer bias and biomassâ€”sequence relationships with an innovative metabarcoding protocol. PloS one, 10(7), e0130324.
Thomas, A. C., Deagle, B. E., Eveson, J. P., Harsch, C. H., & Trites, A. W. (2016). Quantitative DNA metabarcoding: improved estimates of species proportional biomass using correction factors derived from control material. Molecular ecology resources, 16(3), 714-726.
Di Muri, C., Lawson Handley, L., Bean, C. W., Li, J., Peirson, G., Sellers, G. S., ... & HÃ¤nfling, B. (2020). Read counts from environmental DNA (eDNA) metabarcoding reflect fish abundance and biomass in drained ponds. Metabarcoding and Metagenomics, 4, 97-112.
Ershova, E. A., Wangensteen, O. S., Descoteaux, R., Barth-Jensen, C., & PrÃ¦bel, K. (2021). Metabarcoding as a quantitative tool for estimating biodiversity and relative biomass of marine zooplankton. ICES Journal of Marine Science, 78(9), 3342-3355.
For the figures comparing the ecoregions, as they are just three I would recommend a color blind safe palette, orange, yellow and green is not nice.

Methods

Are the methods appropriate to the aims of the study, are they well described, and are necessary controls included? Choose an item.

Conclusions

Are the conclusions adequately supported by the data shown? Choose an item.

Reporting Standards

Does the manuscript adhere to the journal's guidelines on [minimum standards of reporting?] Choose an item.

Choose an item.

Statistics

Are you able to assess all statistics in the manuscript, including the appropriateness of statistical tests used? Choose an item.

Quality of Written English

Please indicate the quality of language in the manuscript: Choose an item.

Declaration of Competing Interests

Please complete a declaration of competing interests, considering the following questions:

- Have you in the past five years received reimbursements, fees, funding, or salary from an organisation that may in any way gain or lose financially from the publication of this manuscript, either now or in the future?
- Do you hold any stocks or shares in an organisation that may in any way gain or lose financially from the publication of this manuscript, either now or in the future?
- Do you hold or are you currently applying for any patents relating to the content of the manuscript?
- Have you received reimbursements, fees, funding, or salary from an organization that holds or has applied for patents relating to the content of the manuscript?
- Do you have any other financial competing interests?
- Do you have any non-financial competing interests in relation to this paper?

If you can answer no to all of the above, write 'I declare that I have no competing interests' below. If your reply is yes to any, please give details below.

I declare that I have no competing interests

I agree to the open peer review policy of the journal. I understand that my name will be included on my report to the authors and, if the manuscript is accepted for publication, my named report including any attachments I upload will be posted on the website along with the authors' responses. I agree for my report to be made available under an Open Access Creative Commons CC-BY license (http://creativecommons.org/licenses/by/4.0/). I understand that any comments which I do not wish to be included in my named report can be included as confidential comments to the editors, which will not be published.

Choose an item.

To further support our reviewers, we have joined with Publons, where you can gain additional credit to further highlight your hard work (see: https://publons.com/journal/530/gigascience). On publication of this paper, your review will be automatically added to Publons, you can then choose whether or not to claim your Publons credit. I understand this statement.

Yes Choose an item.
