# Supplementary material for: Message in a Bottle—Metabarcoding enables biodiversity comparisons across ecoregions
Source: Gigascience. 2022 Apr 28;11:giac040. doi: 10.1093/gigascience/giac040 (PMC9049109; doi:10.1093/gigascience/giac040)
Supplement: giac040_Reviewer_2_Report_Original_Submission — Shanlin Liu -- 9/15/2021 Reviewed [file giac040_reviewer_2_report_original_submission.pdf]

Reviewer Report

Title: Message in a Bottle â€“ Metabarcoding Enables Biodiversity Comparisons Across Ecoregions

Version: Original Submission **Date:** 9/15/2021

Reviewer name: Shanlin Liu

Reviewer Comments to Author:

Steinke et al. used a metebarcoding method to investigate the species compositions for 410 insect bulk samples collected in 3 ecoregions. The manuscript is well written, all the materials and methods were clearly described, I think the manuscript should be accepted for publication after addressing several minor issues as follows:
1. Line 126, as Ion torrent is not widely used nowadays, may the authors add some words regarding its sequencing length, error rate, throughput et al.
2. Please unify the format of chao 1 (or chao-1).
3. A rarefaction curve for each sample may need to check whether the species diversity is well represented by its raw reads.
4. Line 187 - 191. This BIN number inflation may also boil down to sequence errors introduced during PCR amplification or sequencing.
5. Please pay attention to the citation format. For example, in line 202, reference # 40 should follow the first author's name.
6. Line 226 - 227, please add some words to better explain the speculation of "passively transported by wind".

Methods

Are the methods appropriate to the aims of the study, are they well described, and are necessary controls included? Choose an item.

Conclusions

Are the conclusions adequately supported by the data shown? Choose an item.

Reporting Standards

Does the manuscript adhere to the journal's guidelines on minimum standards of reporting? Choose an item.

Choose an item.

Statistics

Are you able to assess all statistics in the manuscript, including the appropriateness of statistical tests used? Choose an item.

Quality of Written English

Please indicate the quality of language in the manuscript: Choose an item.

Declaration of Competing Interests

Please complete a declaration of competing interests, considering the following questions:

- Have you in the past five years received reimbursements, fees, funding, or salary from an organisation that may in any way gain or lose financially from the publication of this manuscript, either now or in the future?
- Do you hold any stocks or shares in an organisation that may in any way gain or lose financially from the publication of this manuscript, either now or in the future?
- Do you hold or are you currently applying for any patents relating to the content of the manuscript?
- Have you received reimbursements, fees, funding, or salary from an organization that holds or has applied for patents relating to the content of the manuscript?
- Do you have any other financial competing interests?
- Do you have any non-financial competing interests in relation to this paper?

If you can answer no to all of the above, write 'I declare that I have no competing interests' below. If your reply is yes to any, please give details below.

I declare that I have no competing interests

I agree to the open peer review policy of the journal. I understand that my name will be included on my report to the authors and, if the manuscript is accepted for publication, my named report including any attachments I upload will be posted on the website along with the authors' responses. I agree for my report to be made available under an Open Access Creative Commons CC-BY license (http://creativecommons.org/licenses/by/4.0/). I understand that any comments which I do not wish to be included in my named report can be included as confidential comments to the editors, which will not be published.

Choose an item.

To further support our reviewers, we have joined with Publons, where you can gain additional credit to further highlight your hard work (see: https://publons.com/journal/530/gigascience). On publication of this paper, your review will be automatically added to Publons, you can then choose whether or not to claim your Publons credit. I understand this statement.

Yes Choose an item.
