# Supplementary material for: Message in a Bottle—Metabarcoding enables biodiversity comparisons across ecoregions
Source: Gigascience. 2022 Apr 28;11:giac040. doi: 10.1093/gigascience/giac040 (PMC9049109; doi:10.1093/gigascience/giac040)
Supplement: giac040_Reviewer_3_Report_Original_Submission — Kingsly Beng -- 9/21/2021 Reviewed [file giac040_reviewer_3_report_original_submission.pdf]

Reviewer Report

Title: Message in a Bottle â€“ Metabarcoding Enables Biodiversity Comparisons Across Ecoregions

Version: Original Submission **Date:** 9/21/2021

Reviewer name: Kingsly Beng

Reviewer Comments to Author:

General comments
Steinke et al used DNA metabarcoding of malaise trap samples from 52 protected areas spanning three Canadian ecoregions to assess the spatial patterns of arthropod biodiversity. The research question is relevant and interesting, the study is well designed, data collected are comprehensive, and manuscript is well written and easy to follow. I enjoyed reading it and would like to thank the authors for such a great contribution. My main concern is that the temporal aspect of the study was not explored even though it was mentioned as part of the research objective.
Specific comments
L60-62: These reductions are not only for abundance but also for diversity, at least based on the fourth reference cited here. I would therefore include "diversity" or "richness" in this statement.
L63 & L105: The authors use biosurveillance in some places in the text and bio-surveillance in others. Isn't it better to stick to the same spelling all through, at least for consistency?
L132: I am a bit confused here. Are these "Analyses" or "Results"? The whole subsection from L133-L176 read like results to me.
L329: "of" omitted! Five samples were available from each of the other 22 sites...
L332-334: The first "following" in this sentence can be either omitted or that part of the sentence completed using "manufacturer's instructions"
L345-346: "Reads were trimmed 30 bp from their 5' terminus with a set trim length of 450 bp". Perhaps this needs more clarification. The amplified length was 463 bp, trimming 30 bp gives 433 bp. How then can set trim length be 450 bp?
L348-349: What was the criterion for using "at least three reads matched an OTU in the reference database"? I mean why not at least two or at least four reads? If this was arbitrary please clarify.
L349-350: Same question as above, why use "a minimum of five reads per cluster"? It would be nice to indicate if any benchmarking was applied a priori or if this was set arbitrarily.
L346-349: Since the authors were mostly interested in arthropods, were reads that matched sequences from bacteria (SYS-CRLBACTERIA), chordates (SYS-CRLCHORDATA) and non-arthropod invertebrates (SYS CRLNONARTHINVERT) discarded or retained? This should be mentioned here and estimates of the number of reads, BINs or OTUs matching each of these categories should be provided.
L149-153: These are interesting results. It would be nice to present them graphically, at least in the supplementary. The aim of the study was "to assess spatial and temporal variation in species richness and diversity in arthropod communities from 52 protected areas spanning three Canadian ecoregions" but the temporal aspect of the study was not fully explored. Although it is stated that "trap catches were harvested every second week from early May through September", this information has not be used in

the analysis. Should the aim of the study be redefined and restricted to just spatial patterns then?
L152-153: Without any table or figure to support these results, why not provide the actual number or proportion or percentage of BINs for each arthropod order in the text?
L157-158: Please add some symbols (e.g. asterisks *, **, ***or alphabet a, b, c) to Figure 3b to represent significant differences. Looking at the present figure without referring to the text does not tell the reader if the differences are significant. Besides, the authors only report a single p value ($p < 0.003$) which probably means at least one of the groups is different from the others but failed to report the pairwise multiple comparison tests that tell the reader which pairs or groups (e.g. ECF vs EGL, ECF vs SGL, EGL vs SGL) are significantly different.
L159: Are the patterns similar if you control for the total number of sites per ecoregion? For example, taking 12 sites per ecoregion and resampling them 100 or 1000 times, similar to the approach used for beta diversity. It could be that one site is driving this pattern, as shown in Figure 2b and reported in L141 "…with more than a third (9,301) found at only one site (Figure 2b)".
L164-166: Please provide the full PERMANOVA results in a table in the text or supplementary and reference it here. It is not clear what "decreased site elevation (R2 166 =â€‰0.035, P =â€‰0.03)" means.
L168-171: Do these patterns change or remain the same if the same number of sites per ecoregion is used? This needs to be tested given that one site (probably from ECF or EGL?) is disproportionate species-rich and SGL has the lowest number of sites.
L173-176: What about levels of turnover across time? Were they any temporal trends in alpha and beta diversity? Was the temporal dropped from the study objective and why?
L221-223: Same question as above, were temporal changes in species composition considered? Which results, tables or figures point to this or how did the authors arrive at these statements?

Methods

Are the methods appropriate to the aims of the study, are they well described, and are necessary controls included? Choose an item.

Conclusions

Are the conclusions adequately supported by the data shown? Choose an item.

Reporting Standards

Does the manuscript adhere to the journal's guidelines on minimum standards of reporting? Choose an item.

Choose an item.

Statistics

Are you able to assess all statistics in the manuscript, including the appropriateness of statistical tests used? Choose an item.

Quality of Written English

Please indicate the quality of language in the manuscript: Choose an item.

Declaration of Competing Interests

Please complete a declaration of competing interests, considering the following questions:

- Have you in the past five years received reimbursements, fees, funding, or salary from an organisation that may in any way gain or lose financially from the publication of this manuscript, either now or in the future?
- Do you hold any stocks or shares in an organisation that may in any way gain or lose financially from the publication of this manuscript, either now or in the future?
- Do you hold or are you currently applying for any patents relating to the content of the manuscript?
- Have you received reimbursements, fees, funding, or salary from an organization that holds or has applied for patents relating to the content of the manuscript?
- Do you have any other financial competing interests?
- Do you have any non-financial competing interests in relation to this paper?

If you can answer no to all of the above, write 'I declare that I have no competing interests' below. If your reply is yes to any, please give details below.

I declare that I have no competing interests

I agree to the open peer review policy of the journal. I understand that my name will be included on my report to the authors and, if the manuscript is accepted for publication, my named report including any attachments I upload will be posted on the website along with the authors' responses. I agree for my report to be made available under an Open Access Creative Commons CC-BY license (http://creativecommons.org/licenses/by/4.0/). I understand that any comments which I do not wish to be included in my named report can be included as confidential comments to the editors, which will not be published.

Choose an item.

To further support our reviewers, we have joined with Publons, where you can gain additional credit to further highlight your hard work (see: https://publons.com/journal/530/gigascience). On publication of this paper, your review will be automatically added to Publons, you can then choose whether or not to claim your Publons credit. I understand this statement.

Yes Choose an item.
