# Supplementary material for: Message in a Bottle—Metabarcoding enables biodiversity comparisons across ecoregions
Source: Gigascience. 2022 Apr 28;11:giac040. doi: 10.1093/gigascience/giac040 (PMC9049109; doi:10.1093/gigascience/giac040)
Supplement: giac040_Reviewer_4_Report_Original_Submission — Christina Lynggaard -- 9/24/2021 Reviewed [file giac040_reviewer_4_report_original_submission.pdf]

Reviewer Report

Title: Message in a Bottle â€“ Metabarcoding Enables Biodiversity Comparisons Across Ecoregions

Version: Original Submission **Date:** 9/24/2021

Reviewer name: Christina Lynggaard

Reviewer Comments to Author:

This manuscript assesses the variation in arthropod communities in three ecoregions in Canada. The study is well done, and the sampling was very thorough with a big sampling effort. I only have minor comments. Specially I consider that the aim can be focused on the ecoregions instead of the feasibility of the method, as this has already been shown. In addition, it would be nice to have more details in certain sections in the data analyses and in the results. I have addressed these comments below.

-I am not sure why the title "Message in a bottle".

-Line 65- Could you specify which indicator species have been targeted? Or cite studies that target those species?

- Line 96- Based on the limitations of the ecoregions, it is not clear why ecoregions are an obvious candidate.

-In line 104 seems that your aim is to demonstrate how feasible is to use metabarcoding for large-scale monitoring and that you use the ecoregions to prove that. However, showing the feasibility of this method for large-scale studies has already been done (e.g. Svenningsen et al 2021, Detecting flying insects using car nets and DNA metabarcoding; Bush et al 2020, DNA metabarcoding reveals metacommunity dynamics in a threatened boreal wetland wilderness). I suggest keeping it focused on the need to apply this method in different ecoregions.

-In the Data description section, you mention that you examined phylogenetic diversity, but in the Analyses section you vaguely mention it. The phylogenetic diversity findings are discussed later on, but it is difficult to follow the discussion when the results were not presented previously. In addition, the authors use the findings in phylogenetic diversity to support the idea of a structure in the ecoregions, so I suggest making more emphasis in this in the results section.

-Line 189. I agree that the higher number of BINs could be due to eDNA, but couldn't another reason be that the BINs were oversplit during data analysis?

-Line 215-217. Has this been found previously in other studies using Malaise trap? If so, please reference to those findings.

-Line222- This is a brief discussion about temporal turnover. However, these results are not presented previously, or at least not clearly enough.

-Line 266-267- Yes, you showed compositional shifts using metabarcoding in bulk arthropod samples, but the way this sentence is structured it sounds like you are the first to show this. Compositional shifts in arthropods have been shown previously in other studies using metabarcoding.

-Line 321- Did you have negative PCR controls? In line 326 you mention negative controls, but I assume you refer to the extraction negative controls.

-Line 340- It is not clear why you queried the data against a bacterial library.

-Line 348- What was the reason for choosing "at least three reads"? and the same for line 350 where you cluster sequences with a minimum of 5 reads per cluster.
-Line 357- If you see tag switching in your negative controls that means that most likely you have it in the rest of the data. How did you ensure that the rest of the data did not have that? You may have tags switching in sequences not found in the negative controls but found in your samples.
-Line 369- As you used the Bray-Curtis index in this metabarcoding data, did you convert your data to presence/absence? It is known that for metabarcoding data the use of read numbers for community analysis is not adequate (see Nichols et al 2018 "Minimizing polymerase biases in metabarcoding").

Methods

Are the methods appropriate to the aims of the study, are they well described, and are necessary controls included? Choose an item.

Conclusions

Are the conclusions adequately supported by the data shown? Choose an item.

Reporting Standards

Does the manuscript adhere to the journal's guidelines on minimum standards of reporting? Choose an item.

Choose an item.

Statistics

Are you able to assess all statistics in the manuscript, including the appropriateness of statistical tests used? Choose an item.

Quality of Written English

Please indicate the quality of language in the manuscript: Choose an item.

Declaration of Competing Interests

Please complete a declaration of competing interests, considering the following questions:

- Have you in the past five years received reimbursements, fees, funding, or salary from an organisation that may in any way gain or lose financially from the publication of this manuscript, either now or in the future?
- Do you hold any stocks or shares in an organisation that may in any way gain or lose financially from the publication of this manuscript, either now or in the future?

- Do you hold or are you currently applying for any patents relating to the content of the manuscript?
- Have you received reimbursements, fees, funding, or salary from an organization that holds or has applied for patents relating to the content of the manuscript?
- Do you have any other financial competing interests?
- Do you have any non-financial competing interests in relation to this paper?

If you can answer no to all of the above, write 'I declare that I have no competing interests' below. If your reply is yes to any, please give details below.

I declare that I have no competing interests

I agree to the open peer review policy of the journal. I understand that my name will be included on my report to the authors and, if the manuscript is accepted for publication, my named report including any attachments I upload will be posted on the website along with the authors' responses. I agree for my report to be made available under an Open Access Creative Commons CC-BY license (http://creativecommons.org/licenses/by/4.0/). I understand that any comments which I do not wish to be included in my named report can be included as confidential comments to the editors, which will not be published.

Choose an item.

To further support our reviewers, we have joined with Publons, where you can gain additional credit to further highlight your hard work (see: https://publons.com/journal/530/gigascience). On publication of this paper, your review will be automatically added to Publons, you can then choose whether or not to claim your Publons credit. I understand this statement.

Yes Choose an item.
